# Structure‐Based Discovery of Obeticholic Acid Derivatives as Novel Farnesoid X Receptor Partial Agonists with Improved Selectivity and Reduced Off‐Target Effects

**DOI:** 10.1002/cmdc.202500960

**Published:** 2026-02-04

**Authors:** Daniela Passeri, Bruno Cerra, Andrea Carotti, Francesco Greco, Sara Piermarini, Carolina Colliva, Paride Liscio, Francesca De Franco, Luciano Adorini, Mary Ruth Erickson, Roberto Pellicciari, Antimo Gioiello

**Affiliations:** ^1^ Tes Pharma S.r.l. Via Giovine Italia 1 06073 Solomeo (PG) Italy; ^2^ Laboratory of Medicinal and Advanced Synthetic Chemistry (Lab MASC) Department of Pharmaceutical Sciences University of Perugia Via del Liceo 1 06123 Perugia Italy; ^3^ Intercept Pharmaceuticals Inc. 3636 Nobel Drive San Diego California CA 92122 USA

**Keywords:** bile acid, Farnesoid X receptor, hX4, molecular dynamics, partial agonists, structure–activity relationships

## Abstract

The Farnesoid X receptor (FXR) is a bile acid‐activated nuclear receptor that represents an important therapeutic target for gut‐liver diseases and metabolic disorders. Recently, FXR partial agonists have gained attention for their potential to minimize side effects resulting from receptor over‐activation. In this study, we report the design, synthesis, and biological evaluation of novel obeticholic acid (OCA) derivatives as selective FXR modulators. Structural modifications at the C3*α* position and the side chain of the bile acid scaffold led to the identification of valine derivatives **2** and **16** as metabolically stable and safe FXR modulators with reduced agonist efficacy. Further molecular dynamics simulations revealed that these compounds induce distinct conformational changes within the FXR ligand‐binding domain, consistent with their partial agonist behavior and resulting in moderate modulation of FXR target genes. Unlike OCA, both compounds failed to activate other steroid‐responsive receptors, including MRGPRX4 (hX4), a G‐protein‐coupled receptor implicated in itching in cholestatic patients, supporting their potential as safer FXR modulators with a reduced risk of pruritus‐related side effects. Overall, this study elucidates key structure–activity relationships governing FXR partial agonism and hX4 binding and offers valuable chemical tools for the development of FXR‐targeted therapeutics with improved safety profiles.

## Graphical abstract

Novel obeticholic acid derivatives were designed and synthesized through targeted structural modifications at the C3*α* position and side chain. Valine analogs act as selective FXR partial agonists with favorable metabolic stability and no cytotoxicity, providing safer chemical tools for developing FXR modulators with reduced side effects such as pruritus.

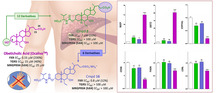

## 1 Introduction

The Farnesoid X receptor (FXR, NR1H4) is a transcription factor that regulates the expression of target genes involved in bile acid (BA), glucose, and lipid homeostasis.^[^ ^1^, ^2^, ^3^ ^]^ Several FXR modulators have been developed and evaluated in clinical trials for the treatment of liver and metabolic conditions, including cholestatic liver diseases, hyperlipidemia, metabolic dysfunction–associated steatohepatitis (MASH), and type 2 diabetes.^[^ ^4^ ^]^ The semisynthetic BA derivative obeticholic acid (OCA, Ocaliva) (**1**) is the first‐in‐class FXR agonist approved by FDA as a second‐line therapy for the treatment of primary biliary cholangitis (PBC).^[^ ^5^ ^,^ ^6^ ^]^ Despite proven efficacy and great potential, results from clinical studies indicate that FXR agonists may induce side effects such as lipid imbalances and pruritus, a common, dose‐dependent symptom observed in PBC patients.^[^ ^7^ ^]^ In addition, elevated cholesterol levels have been reported in clinical trials as the consequence of FXR activation blocking BA biosynthesis and, in turn, cholesterol elimination.^[^ ^8^ ^,^ ^9^ ^]^ Therefore, intense research activity and drug development efforts have been aimed at identifying potent and selective modulators, such as partial agonists, capable of avoiding or minimizing side effects of FXR targeting.^[^ ^10^ ^,^ ^11^ ^]^

Prerequisites for designing partial agonists include understanding the molecular basis allowing modulation of FXR activity, recruitment of coregulators, and the profile of gene expression that, ultimately, determine the compound therapeutic properties.^[^ ^12^ ^]^ While the H12 structure‐function (“mouse‐trap”) model derived from FXR ligand binding domain (LBD) X‐ray structures explains fairly well the relationship between the active state (agonism) and the inactive state (antagonism),^[^ ^13^ ^]^ it does not provide convincing evidences on molecular determinants defining partial agonism and related pharmacological responses.

**Figure 1:**
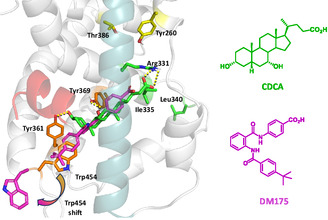
Superposition of CDCA (green sticks) and the anthranilamide‐based partial agonist DM175 (magenta sticks) within the FXR ligand binding domain. The binding poses of CDCA and DM175 have been retrieved from the protein data bank (PDB) code 6HL1,^[^ ^19^ ^]^ and 4QE8 complexes, respectively, after the backbone superposition on the 6HL1 (“inward”) structure. The Trp454 movement upon compound binding is indicated by an arrow, highlighting the shift from the 6HL1 position when FXR is in complex with CDCA to the magenta position (“outward”) induced by the partial agonist. Key residues Trp454, Tyr361, and Tyr369 are shown in orange. Arg331, Ile335 and Leu340 are shown in green sticks, while the S2 binding site residues Tyr260 and Thr386 are shown in yellow sticks. H3 and H12 (AF‐2) cartoons are colored in light blue and red, respectively. H‐bond interactions with CDCA are shown as yellow dashes.

We have previously shown that occupancy of the FXR S2 accessory pocket by a long (hydrophobic) BA side chain produces conformational rearrangements in the receptor that are able to induce partial agonism and gene selective modulation (**Figure** **1**).^[^ ^14^, ^15^, ^16^ ^]^ In particular, Arg331 was identified as a key residue involved in a stable interaction with the C24 tail moiety of BAs, particularly strong in the case of agonists, as confirmed by mutagenesis studies.^[^ ^17^ ^]^ Perturbations at this hot‐spot were likely to stabilize helices H3 and H12 in FXR and to affect ligand efficacy rather than potency.^[^ ^14^, ^15^, ^16^ ^]^ Interestingly, the insertion of the ethyl group at the C6*α* position disrupted the packing of H3, while promoting the packing of H12 and the full, potent activation of the receptor.^[^ ^18^ ^]^ This suggests that, as for PPAR*γ*, FXR partial agonists may act through the stabilization of H3. More recently, Merk and colleagues have demonstrated that a possible way to fine‐tune FXR activation by nonsteroidal molecules (e.g., DM175) involves the partial disruption of the interaction network close to H12 and, in particular, to the activation function‐2 (AF‐2) domain (Figure 1).^[^ ^19^ ^]^ Thus, partial agonism was conferred by an outward movement of Trp454 and a reduced stability of H5‐H6‐loop and H11‐H12‐loop, which are critical for H12 stabilization. These conformational changes were due to the loss of interactions with key residues such as Ty361 and Tyr369 and the positioning of a bulky substituent close to AF‐2 domain. A recent study based on molecular dynamics (MD) simulations and residue‐wise communication network analysis confirmed these findings showing how specific residues and portions of the FXR LBD undergo considerable conformational changes upon binding of an agonist (Tropifexor) or a partial agonist (DM175).^[^ ^20^ ^]^

**Figure 2:**
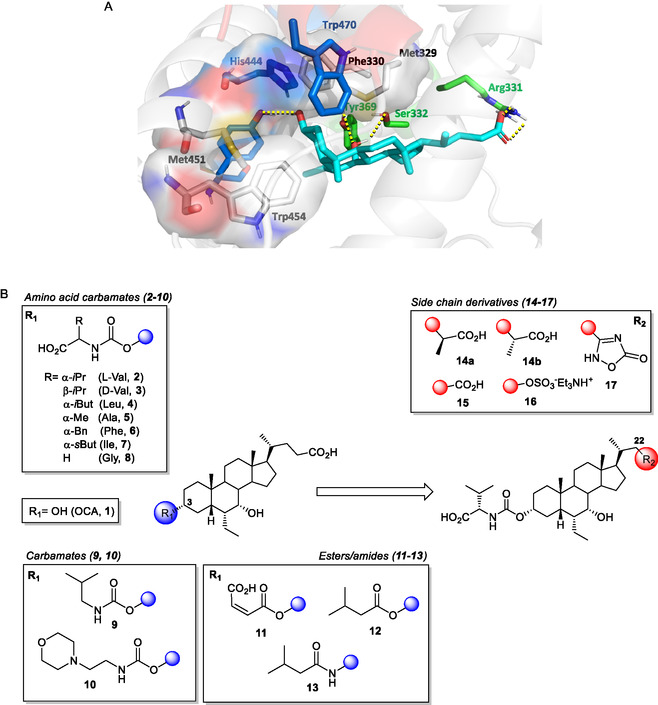
A) Binding mode of OCA (**1**) (cyan sticks) in the FXR LDB (PDB: 6HL1).^[^ ^19^ ^]^ The residues that establish H‐bond interactions (yellow dashed lines) are shown as green sticks and labeled. The residues defining the cavity (white surface) surrounding the C3*α* position are shown as white sticks, while the amino acids of the FXR trigger are shown as blue sticks. B) Structural modifications of OCA (**1**) at the C3*α*‐position (compounds **2**–**13**) and side chain (compounds **14**–**17**).

In this work, we describe the design and synthesis of novel BA derivatives featuring structural modifications at both the C3*α* position and side chain of OCA (**1**) (**Figure** **2**). The goal is to interfere with the AF‐2 domain, thereby enabling an agonist‐to‐partial agonist switch. Notably, none of the OCA derivatives reported to date exhibited FXR partial agonism. As previously mentioned, the presence of the ethyl group at the 6*α*‐position of the chenodeoxycholic acid (CDCA) scaffold provoked a receptor conformational change that results in increased potency and full activation.^[^ ^18^ ^]^ Furthermore, a recent study highlighted the pivotal role of the C3‐OH group of BAs in activating hX4 (also known as MRGPRX4),^[^ ^21^ ^]^ the GPCR implicated in BA‐induced itch symptoms.^[^ ^22^ ^,^ ^23^ ^]^ Based on structural insights into hX4 activation by BAs, we hypothesized that C3 modifications on the OCA scaffold would influence hX4 activation. Therefore, derivatives meeting specific activity and stability criteria were subsequently tested in gene expression experiments to assess target engagement, analyzed by MD simulations to rationalize the molecular basis of partial agonism, evaluated for toxicity and off‐target modulation, including hX4 activation.

## 2 Results and Discussion

### 2.1 Molecular Design

The FXR LBD is composed of twelve helices with the crucial H12 helix enabling the binding and release of coactivator and corepressor proteins at the AF‐2 domain.^[^ ^24^ ^]^ The volume of the ligand binding pocket tends to adapt to the characteristics of the ligands thereby influencing receptor modulation, the dimerization process with the RXR receptor and the recruitment of various cofactor proteins.^[^ ^25^ ^]^ Moreover, FXR ligands can bind distinct regions and engage unique residues within the LBD, leading to specific conformational changes that ultimately determine receptor modulation and gene expression profiles.

Following a visual inspection of OCA (**1**) binding mode in the FXR crystal structure (PDB: 6HL1),^[^ ^19^ ^]^ we hypothesized that derivatization of the C3*α* position with a bulky functional group could lead to FXR partial agonism (Figure 2A). Indeed, structural biology studies have suggested that different degrees of steric hindrance close to AF‐2 domain can fine‐tune FXR activation through a triad of amino acids (Tyr361, His444, Trp470) (the FXR “trigger”), thus providing access to either full, partial, or inverse agonism.^[^ ^26^ ^]^ To prove this hypothesis, a focused library of OCA derivatives (**2**–**13**), bearing diversely substituted carbamate, ester, and amide moieties at the C3‐position of the steroidal nucleus, was synthesized and evaluated as FXR ligands (Figure 2B). Moreover, the most promising partial agonists identified from this first series were further modified at the side chain to improve binding affinity while maintaining a partial agonist profile. These modifications (compounds **14–17**) were inspired by previous findings showing that side‐chain functionalization on the OCA scaffold can enhance FXR potency^[^ ^11^ ^,^ ^18^ ^,^ ^27^ ^]^ and were thus applied here with the aim of reinforcing receptor interactions without promoting full activation.

### 2.2 C3‐Substituted Obeticholic Acid Derivatives

#### 2.2.1 Synthesis

**Scheme 1:**
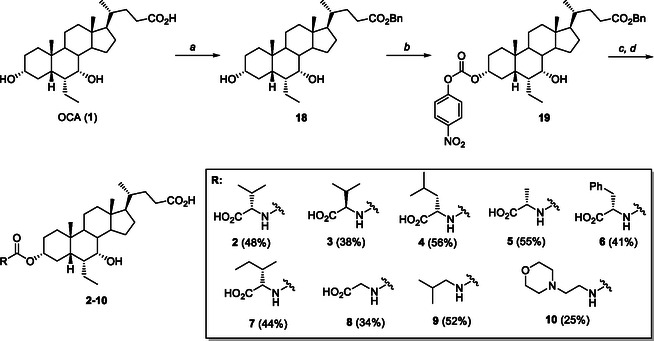
Synthesis of C3‐carbamate derivatives **2**–**10**. *Reagents and conditions*: a) BnBr, Cs_2_CO_3_, MeCN, reflux, 98%; b) 4‐NO_2_PhOC(O)Cl, dry pyridine, 0 °C –> 25 °C, 81%; c) amine or aminoacid, Et_3_N, DMF, 90 °C; d) H_2_, Pd/C, dioxane, 2.5 bar, 25 °C, 60% (**2**), 48% (**3**), 70% (**4**), 69% (**5**), 52% (**6**), 55% (**7**), 43% (**8**), 66% (**9**), and 32% (**10**) from **19**. Product overall yields are reported in brackets.

The synthesis of OCA derivatives **2**–**10** is illustrated in **Scheme** **1**. Thus, the benzyl ester of OCA (**18**) was obtained by reaction with benzyl bromide (BnBr) and cesium carbonate (Cs_2_CO_3_) in refluxing MeCN (98%). Treatment of **18** with *p*‐nitrophenylchloroformate in dry pyridine afforded the carbonate intermediate **19** (81%) that was readily reacted with diverse amino acids and amines. The final reductive debenzylation via hydrogenolysis (Pd/C, H_2_ in dioxane) gave the desired OCA carbamates **2**–**10** in 32–70% isolated yields from **19** after silica gel chromatography (Scheme 1).

**Scheme 2:**
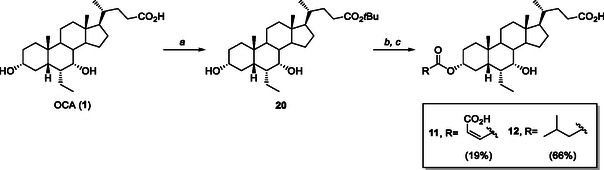
Synthesis of C3‐ester derivatives **11** and **12**. *Reagents and conditions*: a) *t*‐BuOH, H_2_SO_4_, reflux, quantitative; b) maleic anhydride, DMAP, pyridine, reflux, or *i*‐valeryl chloride, pyridine, CH_2_Cl_2_, 25 °C; c) TFA, CH_2_Cl_2_, 0 °C –> 25 °C. Product overall yields are reported in brackets.

The C3‐ester analogs **11** and **12** were obtained from the *t*‐butyl ester of OCA (**20**) by reaction with maleic anhydride or *i‐*valeryl chloride, respectively, in the presence of catalytic *N, N*‐dimethylaminopyridine (DMAP) in pyridine, followed by treatment with trifluoroacetic acid (TFA) in CH_2_Cl_2_. The desired products **11** and **12** were isolated with an overall yield of 19% and 66%, respectively (**Scheme** **2**).

**Scheme 3:**
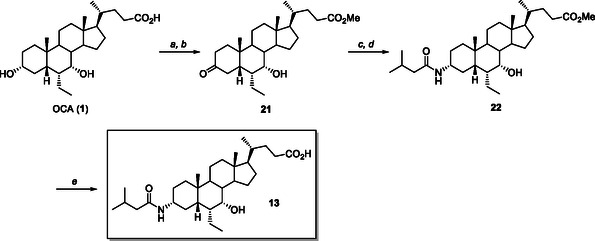
Synthesis of C3‐isobuthylamide derivative **13**. *Reagents and conditions*: a) *p*‐TSA, MeOH, ultrasound; b) Fetizon's reagent, toluene, reflux, 84% from **1**; c) NH_3_ in MeOH, HCO_2_NH_4_, NaCNBH_3_, MeOH, 25 °C; d) *i*‐valeryl chloride, pyridine, CH_2_Cl_2_, 25 °C, 31% from **21**; e) NaOH, MeOH, 87%.

For the preparation of the *i*‐butylamide **13**, the methyl ester of OCA was selectively oxidized at C3 position by treatment with Fetizon's reagent in refluxing toluene (**Scheme** **3**).^[^ ^28^ ^]^ The resulting C3‐oxo intermediate **21** (84% yield) was submitted to a stereoselective reductive amination at C3 position by treatment with a methanolic solution of ammonia, ammonium formate and sodium cyanoborohydride (NaCNBH_3_) used as the reducing agent.^[^ ^29^ ^]^ The following reaction with *i‐*valeryl chloride and pyridine in CH_2_Cl_2_ gave the desired C3*α*‐isobutylamide **22** in 31% isolated yield from OCA (**1**). The final alkaline hydrolysis of the methyl ester group at C24 position under mild conditions (NaOH, MeOH, 25 °C) afforded the desired derivative **13** in 87% yield from **22** (Scheme 3).

#### 2.2.2 AlphaScreen and Metabolic Stability Assays

The newly prepared derivatives **2**–**13** were tested in a biochemical AlphaScreen assay to evaluate their ability to activate FXR, using the endogenous FXR agonist CDCA and OCA (**1**) as reference standards. As shown in **Table** **1**, all synthesized derivatives exhibited reduced efficacy compared to CDCA and OCA (**1**), indicating that they behave as FXR partial agonists in this assay. These results are consistent with our initial hypothesis, which proposed that bulky substituents at C3*α*‐position of BAs introduce steric hindrance that limits full receptor activation. Minor structural changes were generally associated with a decrease in potency, suggesting that the LBD cavity surrounding the C3*α* position is constrained, leaving limited space for productive interactions. In particular, BA carbamates derived from coupling with non‐aromatic amino acids (compounds **2**–**4**, **7**) were able to bind the FXR receptor in a low micromolar potency range (7–25 μM), except for the L‐alanine and glycine derivatives (**5** and **8**), which failed to induce FXR modulation. Interestingly, removal of the carboxylic group (compound **9**) did not significantly affect FXR potency, which was also preserved in the corresponding ester (compound **12**) and the maleic acid derivative (compound **11**), as shown in Table 1.

**Table 1:** FXR activity and metabolic stability in mouse microsomes of C3‐modified obeticholic acid derivatives **2–13**.

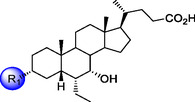						
Cmpd	R	FXR activitya)		Microsomal stability (mouse)b)		
		EC_50_	Efficacy	t_1/2_ [min]b)	CL_int_ c)	Test item % remaining last time point
OCA (**1**)	‐H	0.15 ± 0.05	230 ± 10	>120	<11.59	100
**2**	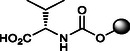	7.0 ± 0.3	20.0 ± 0.1	>120	<11.59	100
**3**	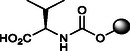	10.0 ± 1.5	10.0 ± 2.3	>120	<11.59	100
**4**	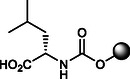	11.0 ± 0.3	20.0 ± 0.1	>120	<11.59	100
**5**	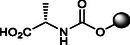	52.0 ± 0.4	55.0 ± 0.1	>120	<11.59	100
**6**	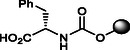	>100		>120	<11.59	100
**7**	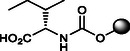	25 ± 1	15.0 ± 0.3	>120	<11.59	100
**8**	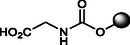	>100		>120	<11.59	100
**9**	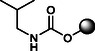	4.5 ± 0.2	45 ± 1	31.36 ± 2.45	44.38 ± 3.47	50
**10**	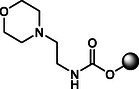	10.0 ± 0.3	70 ± 4	12.6 ± 1	110.35 ± 8.80	21
**11**	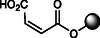	10.0 ± 1.4	13.0 ± 2.5	>120	<11.59	100
**12**	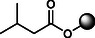	1.4 ± 0.1	24 ± 2	6.9 ± 0.3	201.2 ± 7.49	0
**13**	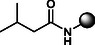	>100		17.3 ± 0.1	80.40 ± 0.64	31

a) Data represents average values of at least three independent experiments of AlphaScreen assay. Units are μM for EC_50_, % of 50 μM CDCA value for efficacy;

b) Half‐life in mouse microsomes expressed in minutes. Values are mean ± SD from duplicates by LC‐MS/MS analysis;

c) Intrinsic clearance expressed in μL/min/mg protein. Values are mean ± SD from duplicates.

All the synthesized BA derivatives were then evaluated in mouse liver microsomes to assess their metabolic stability when exposed to drug‐metabolizing cytochromes (Table 1 and Figure S1–S13, Supporting Information). Most of the tested compounds displayed high metabolic stability, with half‐lives exceeding 120 min and intrinsic clearance (CL_int_) values below 11.59 µL min^−1^ mg^−1^ protein. Among the carbamates, only compounds **9** and **10** showed reduced stability, with half‐lives of ≈31 and 13 min, respectively. Interestingly, compound **10** is likely to undergo oxidative metabolism at the morpholine moiety, while the *i*‐butyl derivative **9** was found to be partially converted into the parent compound OCA (**1**) (50%).^[^ ^30^ ^]^ Similarly, the ester (**12**) and the amide analog (**13**) also exhibited a certain instability in mouse microsomes, leading to the formation of OCA (**1**) in the case of compound **12** (100%) and hydroxylated metabolites (31%) for compound **13**.

#### 2.2.3 Molecular Docking and SAR Analysis

A docking analysis carried out to investigate the molecular basis underlying the FXR activity observed in AlphaScreen assays (Table 1). Given the structural similarity between the co‐crystallized BA with the synthesized compounds, the LBD in complex with the natural FXR agonist CDCA was selected as the template for the docking studies.^[^ ^19^ ^]^ Based on the AlphaScreen data, compounds were classified into three potency levels: active (**2**–**4**, **9**–**12**), moderately active (**5** and **7**) and inactive (**6** and **13**).

**Figure 3:**
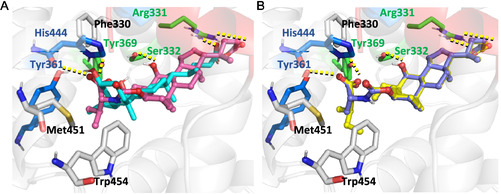
A) Binding modes of OCA (**1**) and compound **2** (cyan and pink ball and sticks, respectively) in the FXR LDB (PDB: 6HL1).^[^ ^19^ ^]^ The interacting residues are shown as white, blue and green sticks and labeled, while the H‐bonds engaged by compound **2** are shown in yellow dashed lines. B) Binding modes of compounds **5** and **7** (yellow and purple ball and sticks, respectively) in the FXR LDB (PDB: 6HL1).^[^ ^19^ ^]^ The interacting residues are shown as white, blue and green sticks and labeled, while H‐bonds engaged are shown in yellow dashed lines.

The comparison between the docking pose of OCA (**1**), used as reference compound, and compound **2,** selected as representative active derivative, revealed four key H‐bonds engaged with LBD residues Arg331, Ser332, Tyr361, and Tyr369 (**Figure** **3**). Notably, the negatively charged C24‐carboxylic group of compound **2** appeared to induce a more folded conformation of the side chain compared to OCA (**1**), enabling a favorable electrostatic interaction with Arg331. Additionally, the carboxylic group of the amino acid moiety at the C3‐position of compound **2** formed H‐bond interactions with Tyr361 and Tyr369. These interactions are analogous to those established by the C3*α*‐ and C7*α*‐hydroxyl groups of OCA (**1**), respectively (Figure 3A). Interestingly, the isopropyl moiety of compound **2** appeared to occupy the hydrophobic pocket typically engaged by the 6*α*‐ethyl substituent of OCA, suggesting a similar role in receptor binding stabilization.

The moderately active compounds **5** and **7** were able to retain the key H‐bond interactions observed for derivative **2** (Figure 3B). However, the lower potency of compound **5** compared with **2** can be attributed to the complete loss of occupancy of the hydrophobic cavity that accommodates the ethyl group of OCA (**1**). Interestingly, derivative **7** was able to partially fill this cavity, but a portion of its butyl group was oriented toward the Trp454, one of the key trigger residues involved in FXR activation. The interaction of the ligand with this critical region of the binding site may interfere with the proper conformational dynamics of the receptor, potentially destabilizing the active conformation and thus accounting for the reduced activity observed for this compound.

**Figure 4:**
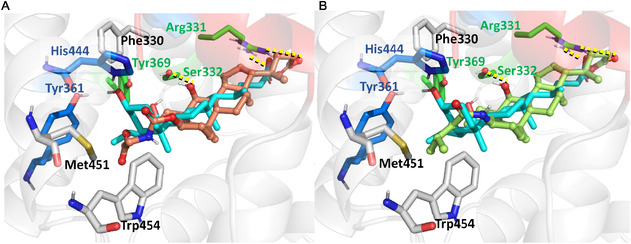
A) Binding modes of OCA (**1**) and compound **8** (cyan and orange ball and sticks, respectively) in the FXR LDB (PDB: 6HL1).^[^ ^19^ ^]^ The interacting residues are shown as white, blue and green sticks and labeled, while the H‐bonds engaged by compound **8** are shown in yellow dashed lines. B) Binding modes of OCA (**1**) and compound **13** (cyan and green ball and sticks, respectively) in the FXR LDB (PDB: 6HL1).^[^ ^19^ ^]^ The interacting residues are shown as white, blue and green sticks and labeled, while the H‐bonds engaged by compound **13** are shown in yellow dashed lines.

Analyses of inactive compounds revealed that no reliable binding pose could be generated for compound **6**, suggesting a lack of compatibility with the FXR binding site. Regarding derivative **8**, its predicted binding mode positioned the C3*α* carboxylic group within the highly hydrophobic pocket typically occupied by the 6*α*‐ethyl group of OCA (**1**) (**Figure** **4**A). In this orientation, the carboxylic acid failed to establish any H bond interactions, further compromising its binding potential. Similarly, the docking pose of the BA **13** showed no H bonding with either Tyr361 or Tyr369. As observed for compound **5**, compound **13** failed to occupy the cavity hosting the 6*α*‐ethyl group. Moreover, like compound **7**, it oriented its *i*‐propyl group toward Trp454, a key residue within the FXR activation trigger. The analysis of these inactive derivatives (i.e., compounds with EC_50_ values > 100 μM) was instrumental to clarify the structural features required for effective FXR activation. Collectively, these findings highlighted three critical aspects: (i) The H‐bond network involving Tyr361 and Tyr369 is essential for compound binding affinity; (ii) the hydrophobic cavity occupied by the C6*α*‐ethyl of OCA (**1**) must be effectively filled to ensure proper ligand anchoring; (iii) occupation of the binding region near Trp454 should be avoided, as it may interfere with receptor activation and reduce ligand activity.

### 2.3 Side Chain‐Modified OCA Derivatives

In view of its overall profile, the L‐valine carbamate derivative **2** was selected for further SAR investigations through modifications of the terminal BA side chain (Figure 2B). The aim was to better understand the role of the side chain in modulating FXR activity. As previously discussed (Figure 3A), the side chain orientation of compound **2** differs from that of OCA (**1**), suggesting distinct interaction patterns within the binding pocket. Inspection of the binding pose of **2** in the FXR binding site led to two key observations: a) to optimize the electrostatic interactions with residues Arg331 and Arg264, *nor* derivatives as well as functional groups with different charge distribution (e.g., sulfates) could potentially improve binding affinity; b) a hydrophobic, partially solvent‐exposed *sub*‐pocket, located near Ile335 and Leu340 (Figure 1), can be occupied by a methyl group inserted at the C23‐position.^[^ ^31^ ^]^

#### 2.3.1 Synthesis

**Scheme 4:**
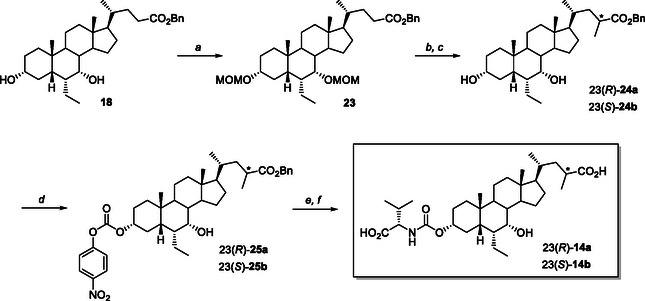
Synthesis of C23‐methyl‐C3‐L‐valyl‐carbamate analogs **14a** and **14b**. *Reagents and conditions*: a) MOMCl, DIPEA, CH_2_Cl_2_, reflux, 84%; b) *n*BuLi, DIPA, MeI, THF, ‐78 °C; c) HCl, THF, 50 °C, 13% for **24a** and 22% for **24b**; d) 4‐NO_2_PhOC(O)Cl, dry pyridine, 0 °C –> 25 °C, 81% for **25a** and 83% for **25b**; e) L‐valine, Et_3_N, DMF, 90 °C; f) H_2_, Pd/C, dioxane, 2.5 bar, 25 °C, 69% for **14a** and 65% for **14b**.

For the preparation of C23‐methyl analogs **14a** and **14b**, we adopted the synthetic strategy illustrated in **Scheme** **4**. OCA benzyl ester **18** was protected as methoxymethyl ether by treatment with methoxymethyl chloride (MOMCl) and diisopropylethylamine (DIPEA) in refluxing CH_2_Cl_2_. The C3, C7‐diprotected intermediate **23** (84% yield) was submitted to the C23‐alkylation reaction by treatment with in‐ situ generated lithium diisopropylamide (LDA) and methyl iodide in freshly distilled THF at −78 °C.^[^ ^31^ ^]^ Hydrolysis of the crude C23‐epimeric mixture with HCl in THF at 50 °C afforded the corresponding C23(*R*)‐methyl and C23(*S*)‐methyl intermediates **24a** and **24b** in 13% and 22% yield from **23**, respectively, after chromatographic purification. Treatment with *p*‐nitrophenyl chloroformate in dry pyridine gave the corresponding C3‐carbonates **25a** (83%) and **25b** (81%), which underwent to C3‐nucleophilic substitution with L‐valine and reductive hydrogenolysis (H_2_, Pd/C). The desired C3‐carbamate‐C23(*R*)‐ and C23(*S*)‐methyl analogs **14a**–**b** were obtained as pure products in 69 and 65% yield, respectively (Scheme 4).

**Scheme 5:**
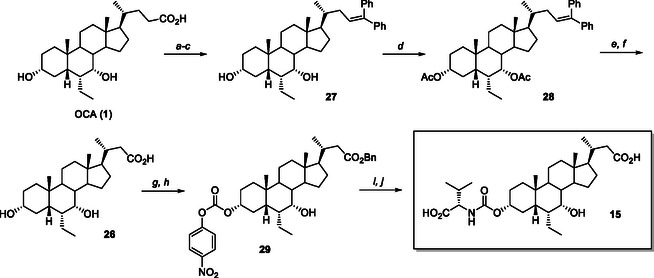
Synthesis of C24‐*nor*‐C3‐L‐valyl‐carbamate analog **15**. *Reagents and conditions*: a) *p*TSA, MeOH, ultrasound; b) PhMgBr, THF, reflux; c) HCl, EtOH, 50 °C, 80% from **1**; *d)* Ac_2_O, Bi(OTf)_3_, CH_2_Cl_2_, 25 °C, quantitative; e) RuCl_3_, NaIO_4_, H_2_SO_4_, AcOEt, MeCN, H_2_O, 0 °C –> 25 °C; f) NaOH, MeOH, reflux, 82% from **28**; g) BnBr, Cs_2_CO_3_, MeCN, reflux; h) 4‐NO_2_PhOC(O)Cl, dry pyridine, 0 °C –> 25 °C, 77% 80% from **26**; i) L‐valine, Et_3_N, DMF, 90 °C; j) H_2_, Pd/C, dioxane, 2.5 bar, 25 °C, 54%.

The synthesis of C24‐*nor*‐C23‐carboxylic acid analog **15** required the preparation of the C24‐*nor*‐OCA (**26**) by Barbier–Wieland degradation via Grignard addition, acid‐promoted dehydration, and ruthenium‐catalyzed oxidative cleavage (6 steps, 65% yield) (**Scheme** **5**).^[^ ^27^ ^]^ C24‐*nor*‐OCA (**26**) thus obtained was converted to the desired C3‐L‐valyl carbamate **15** as previously described for compound **2**.

**Scheme 6:**
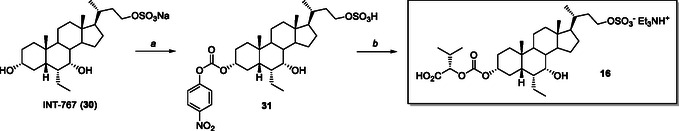
Synthesis of C24‐*nor*‐C23‐sulfate‐C3‐L‐valyl‐carbamate analog **16**. *Reagents and conditions*: a) 4‐NO_2_PhOC(O)Cl, dry pyridine, 0 °C –> 25 °C, 43%; b) L‐valine, Et_3_N, DMF, 90 °C, 54%.

Similarly, compound **16** was prepared from INT‐767 (**30**) in 23% overall yield (**Scheme** **6**).^[^ ^27^ ^]^

**Scheme 7:**
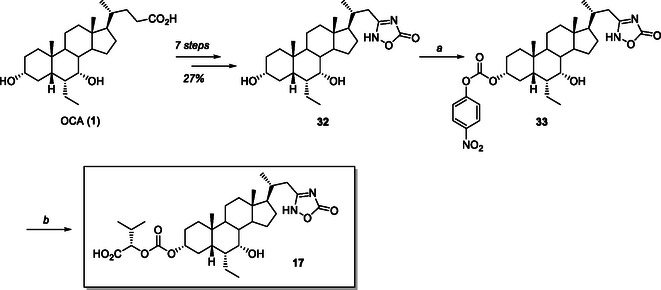
Synthesis of C3‐valyl carbamate of 22,23‐*bisnor*cholan‐oxadiazolone **17**. *Reagents and conditions*: a) 4‐NO_2_PhOC(O)Cl, dry pyridine, 0 °C –> 25 °C, 74%; b) L‐valine, Et_3_N, DMF, 90 °C, 52%.

For the preparation of the C3‐valyl carbamate of 22,23‐*bisnor*‐cholan‐oxadiazolone **17**, OCA (**1**) was converted into the key intermediate **32** in 27% yield over seven steps according to a previously reported synthetic approach (**Scheme** **7**).^[^ ^32^ ^]^ Intermediate **32** thus obtained was activated at C3 position by treatment with *p*‐nitrophenyl chloroformate in dry pyridine to give the corresponding C3‐carbonate intermediate **33** (74% yield), which was then treated with L‐valine and triethylamine in dry DMF at 90 °C to give the desired C3‐L‐valyl‐carbamate analog **17** in 52% yield (Scheme 7).

#### 2.3.2 AlphaScreen Assays

The newly synthesized side chain derivatives of OCA (**14**–**17**) were evaluated as FXR modulators using the AlphaScreen assay (**Table** **2**), with CDCA and OCA (**1**) again employed as the reference standards. The results clearly indicated that most of the compounds exhibited reduced efficacy compared to CDCA and OCA (**1**), consistent with a partial agonist profile. Among the tested compounds, the *nor*‐sulfate derivative **16** showed an improved potency (EC_50_ = 0.83 µM) relative to the parent compound **2**, while retaining a significant reduced efficacy (12%) compared to the parent INT‐767 (EC_50_ = 0.030 µM, efficacy = 280%). Conversely, the oxadiazolone moiety in compound **17** enhanced efficacy (65%) but did not improve potency (EC_50_ = 11 µM). In contrast, shortening of the side chain in the C24‐*nor*‐acid derivative **15,** as well as methyl substitution at the C23 position of **2**, were not tolerated, leading to lack of activity (Table 2).

**Table 2:** FXR activity of obeticholic acid derivatives **14−17**.

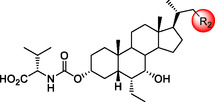			
Cmpd	R_2_	FXR activitya)	
		EC_50_	Efficacy
OCA (**1**)	–	0.15 ± 0.05	230 ± 10
**2**	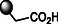	7 ± 1	20.0 ± 0.8
**14a**	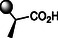	>100	
**14b**	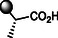	>100	
**15**	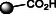	>100	
**16**	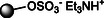	0.83 ± 0.11	12.0 ± 0.9
**17**	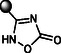	11.0 ± 0.4	65.0 ± 3.2

a) Data represents average values of at least three independent experiments of AlphaScreen assays. Units are μM for EC_50_ and % of 50 μM CDCA value for efficacy.

### 2.4 FXR Molecular Dynamics Simulations

**Figure 5:**
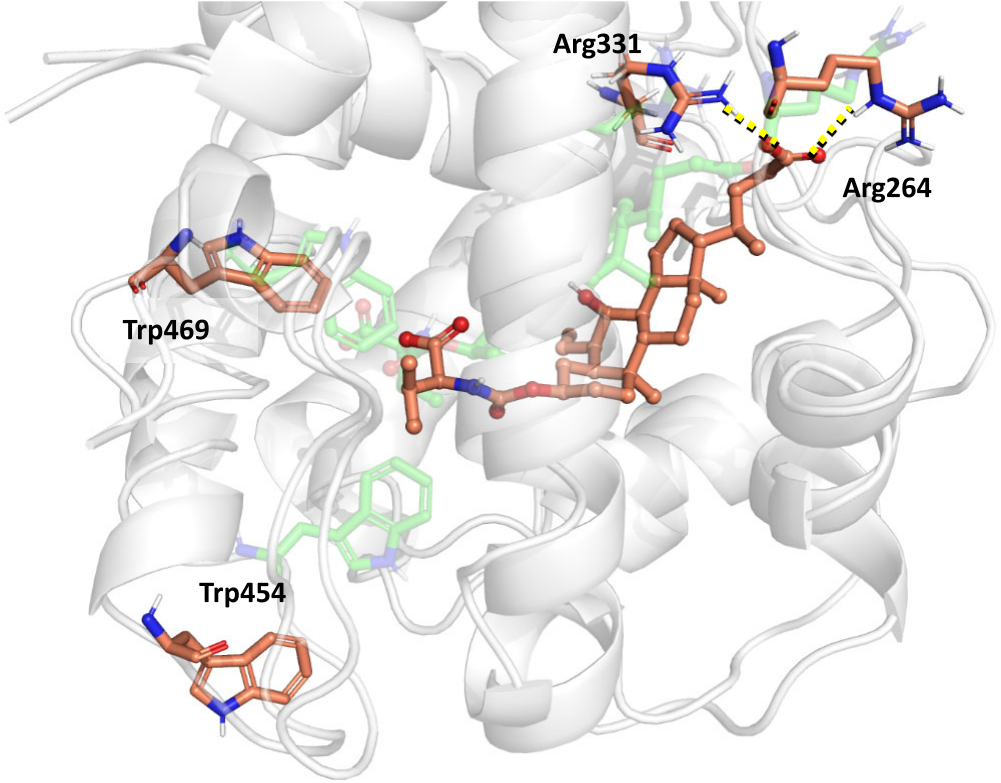
Superposition of starting frame (full agonist ‘inward’ orientation of Trp454, *green* sticks and transparent) and last frame (‘outward’ orientation of Trp454, *orange sticks*) of compound **2** molecular dynamics simulations. The residues are shown as sticks and labeled, while the H‐bonds engaged by compound **2** are shown in yellow dashed lines.

In view of the high plasticity of the FXR LBD, and to further rationalize the activity of compound **2** and related side chain derivatives **14**−**17**, long‐timescale MD simulations (1 µs) were performed. The goal was to assess whether these compounds could induce conformational changes similar to those reported for nonsteroidal FXR partial agonists^[^ ^19^ ^,^ ^33^ ^]^ and to gain deeper insights into their binding mode and key interactions. For clarity and conciseness, the discussion focuses on the analysis of the final MD simulation frame, while additional data, including ligand and receptor RMSD plots and ligand–receptor contact maps, are provided in the Supporting Information (Figure S14–S19, Supporting Information). Compound **2** maintained a binding pose distinct from the full agonist OCA (**1**), confirming the different spatial orientation of its steroidal scaffold within the FXR LBD. Notably, both the valine moiety at the C3*α*‐position and the side chain of **2** occupied alternative regions of the binding pocket. The steric hindrance introduced by the valine substituent appeared to be a key factor driving the displacement of the core scaffold, ultimately resulting in a different orientation of the terminal carboxylic group compared to OCA (**1**) (**Figure** **5**).

Interestingly, in contrast to full agonists, compound **2** was found to induce a conformational shift in helix *α*11, promoting an “outward” orientation of Trp454.^[^ ^19^ ^,^ ^33^ ^]^ This residue and the associated conformational rearrangement were previously reported by Merk and colleagues in the in the crystal structure of FXR bound to the non‐steroidal partial agonist DM175 (PDB ID: 4QE8, Figure 1). To the best of our knowledge, our MD simulations are the first to capture a similar conformational change induced by a BA‐derived FXR partial agonist (Figure 5). This finding was further supported by the recent study of Asthana et al.,^[^ ^20^ ^]^ which also confirmed the role of Trp454 repositioning in FXR modulation. Collectively, the MD results revealed that compound **2** adopts a distinct binding conformation relative to full agonists, both in terms of scaffold orientation and receptor conformational response, characterized in particular by the outward displacement of Trp454.

To provide a direct comparison with a well‐known full agonist, MD simulations were also performed for the OCA‐FXR LBD‐SRC‐1 ternary complex. Additionally, to gain deeper insight into the activity profiles of derivatives **14**−**16**, these compounds were subjected to analogous MD simulations in complex with the FXR LBD and coactivator peptide SRC‐1. For all inactive compounds, the stable H‐bond interactions with Tyr361 or Tyr369 were absent. This finding highlights the crucial role of at least one of these residues in maintaining productive ligand binding to FXR (**Table** **3**). In particular, the loss of activity upon methyl substitution at the C23 position (compounds **14a**−**b**) or the side chain shortening (compound **15**) can be rationalized by the failure to establish these key interactions. The lack of a transferable SAR going from OCA (**1**) to compound **2** further supports the notion that these two ligands adopt fundamentally different orientations within the FXR binding pocket, involving distinct sets of interacting residues and scaffold conformations. Taken together, these data demonstrate that even small structural modifications of the BA side chain, whether in length or steric bulk, can result in complete loss of FXR activity in this class of compounds.

**Table 3:** H‐bond occupancy (%) during MD simulations without steroid receptor coactivator 1 (SRC‐1) for tested FXR full agonists and partial agonists.

Compound	Full/partial agonist	FXR EC_50_ [μM] [% efficacy]	H‐bond occupancy [%]	
			Tyr361	Tyr369
OCA (**1**)	Full agonist	0.15 [230]	15	90
**2**	Partial agonist	7 [20]	80	50
**16**	Partial agonist	0.83 [12]	60	90
**14b**	Partial agonist	>100	5	5
**14a**	Partial agonist	>100	0	10
**15**	Partial agonist	>100	10	0

**Figure 6:**
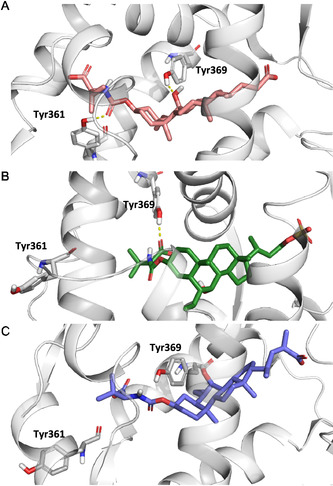
Representative snapshots from MD simulations showing the presence or absence of interactions with Tyr361 and/or Tyr369 of A) 2 (orange sticks), B) 16 (dark green sticks), and one inactive derivative C) 14b (violet sticks). The residues are shown as sticks and labeled, while the hydrogen bonds engaged are shown in yellow dashed lines.

In **Figure** **6**, representative frames from the MD simulations are shown to highlight the interactions between the ligand and residues Tyr361 and Tyr369. Among the tested modifications, the sulfate bioisosteric substitution (compound **16**) emerged as the only side chain alteration that improved FXR binding affinity,^[^ ^27^ ^]^ while preserving partial agonist behavior. During the MD trajectory, compound **16** maintained a stable H‐bond with Tyr369, along with less persistent, though still relevant, interactions with Tyr361. Notably, a conformational shift of the Trp454 side chain, similar to that observed for compound **2**, was also induced by compound **16** during the simulation. To provide a comprehensive overview of the different Trp454 orientations observed across the MD trajectories, a plot of its torsional angles is included in Figure S20, Supporting Information.

### 2.5 Pharmacological Profile of Selected Compounds 2 and 16

#### 2.5.1 FXR Target Gene Expression

In the AlphaScreen assay, compounds **2** and **16** bound to and activated the FXR receptor with potencies of 7 µM and 0.83 µM, and binding efficacies of 25 and 12%, respectively (Figure S21, Supporting Information), displaying dose–response profiles characteristic of partial agonists. To corroborate these findings, compounds **2** and **16** were further assessed in cell‐based transactivation assays employing HEK293T cells transiently transfected with *h*FXR and its canonical responsive element (IR1)‐Luc (Figure S22, Supporting Information). The FXR activation elicited by both compounds was lower than that observed with CDCA, thereby reinforcing their classification as partial FXR agonists.

**Figure 7:**
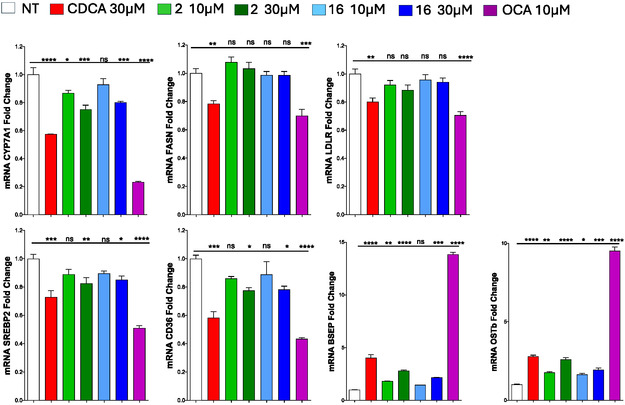
Results of RT‐PCR in HepG2 cell line following overnight treatment with endogenous FXR agonist CDCA (*red*) (30 μM), OCA (*purple*) (10 µM), compound 2 (*green*) (10 μM and 30 μM), or compound 16 (*blue*) (10 μM and 30 μM). Not treated cells (*white*) were stimulated with DMSO 0.1. % Statistical significance was analyzed by One‐way Anova‐GraphPad Prism 5. ns = not significant, *p < 0.05, **p < 0.01, ***p < 0.001 *vs* 0.1% NT = not treated (DMSO).

Both compounds were then tested in human liver cell line (HepG2) for their ability to modulate key FXR target genes, using CDCA and OCA (**1**) as the reference agonist (**Figure** **7**). Activation of FXR by derivatives **2** and **16** determines the repression of CYP7A1 expression, although to a lesser extent than that observed with CDCA and OCA (**1**). Specifically, CYP7A1 was downregulated by ≈30% and 20% with compounds **2** and **16**, respectively, compared with 50% reduction induced by CDCA or 80% reduction after stimulation with OCA (**1**). Moreover, both derivatives moderately upregulated the expression of the BA transporters BSEP and OST*β*, albeit with significantly lower efficacy than CDCA and OCA (**1**), suggesting a reduced impact on BA homeostasis.

Clinical data indicate that full FXR agonists such as CDCA and OCA (**1**) reduce HDL levels and increase LDL concentrations.^[^ ^4^ ^]^ To investigate potential off‐target effects related to lipid metabolism, genes involved in cholesterol homeostasis,^[^ ^34^ ^]^ including CD36, SREBP2, FASN, and LDLR, were analyzed. While CDCA and OCA (**1**) markedly downregulated CD36, compounds **2** and **16** exerted only moderate effects. In contrast, neither compound significantly modulated genes associated with fatty acid synthesis or HDL/LDL regulation at effective concentrations, consistent with the modest suppressive effect exerted by FXR full agonists in vitro.

Overall, these data show that both derivatives are able to modulate all the tested genes but with a lower efficiency compared with the endogenous FXR agonist CDCA or with the full agonist OCA (**1**) (Figure 7), confirming the partial agonist profile of compounds **2** and **16**. This partial activation of FXR may translate into a reduced risk of adverse effects typically associated with full receptor activation.

#### 2.5.2 Cytotoxicity and Steroid Receptor Selectivity

After confirming the metabolic stability in both human and mouse liver microsomes (Table S1, Supporting Information), the toxicity of compounds **2** and **16** was evaluated in two complementary in vitro assays. Cytotoxicity was evaluated by measuring intracellular ATP levels, while cell necrosis was assessed *via* LDH release. As shown in Figure S23 (Supporting Information), neither compound exhibited cytotoxic effects in HepG2 cells at concentrations up to 500 µM, indicating a favorable cellular tolerance.

AlphaScreen assays demonstrated that compounds **2** and **16** selectively modulate FXR, without affecting other steroid nuclear receptors (Figure S24, Supporting Information). In addition, both compounds were tested in enterocyte cells for their ability to activate TGR5, the G protein‐coupled receptor specific for BAs. Neither compound was able to activate this receptor, further supporting their FXR‐selective profile.

#### 2.5.3 Activity and SAR at hX4 Receptor

Pruritus is one of the most debilitating symptoms associated with cholestatic liver diseases, including PBC and primary sclerosing cholangitis (PSC).^[^ ^34^ ^]^ Recent studies have identified hX4 receptor, a G protein‐coupled receptor (GPCR) expressed in dorsal root ganglion (DRG) sensory neurons, as a key mediator of BA‐induced pruritus.^[^ ^35^ ^,^ ^36^ ^]^ In particular, 3‐sulfated BAs have emerged as endogenous ligands of hX4, playing a central role in the pathophysiology of cholestatic itch. A recent cryo‐EM study by Yang et al. elucidated the structural basis of hX4 activation, demonstrating how BAs bind the canonical ligand‐binding pocket of the receptor, with critical interactions mediated by the 3‐OH of the BA.^[^ ^21^ ^]^ Guided by these structural insights, we hypothesized that compound **2** and **16** might retain FXR partial agonist activity, crucial for therapeutic efficacy in liver diseases, while avoiding hX4 activation and the associated pruritic side effects. To test this hypothesis, we evaluated their activity at hX4 using a cell‐based label‐free assay, with the aim of confirming both the absence of hX4 agonism and the preservation of selective FXR modulation.

**Figure 8:**
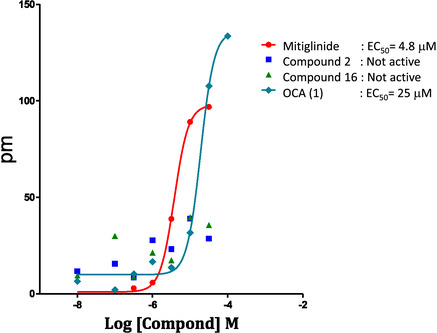
Binding assays of OCA (**1**), compounds **2** and **16** at *h*X4. Compounds were used at concentrations ranging from 0.1 to 50 μM, using mitiglinide as the positive control.

To assess potential activity at MRGPRX4, CHO cells stably expressing the receptor were stimulated with compounds **2** and **16**, and receptor engagement was monitored using dynamic mass redistribution (DMR) technology. As expected, the positive control mitiglinide induced a dose‐dependent increase in picometer shift, consistent with effective MRGPRX4 activation. In contrast, compounds **2** and **16** did not induce any significant DMR signal at the tested concentrations, suggesting that they neither bind to nor activate the receptor (**Figure** **8**). Conversely, OCA (**1**) was confirmed to bind and activate MRGPRX4 at micromolar concentrations, in agreement with previous findings.^[^ ^21^ ^]^

Given the availability of the hX4 cryo‐EM structure in complex with deoxycholic acid 3‐phosphate (DCA‐3P) (PDB: 8KEX),^[^ ^21^ ^]^ we performed an in‐ silico investigation of compounds **2** and **16** to rationalize the observed data. Docking calculations were followed by molecular dynamics (MD) simulations, embedding the receptor–ligand complexes in a POPC phospholipid bilayer. DCA‐3P was included as a reference ligand to enable direct comparison of binding interactions. For each complex, three independent 300 ns MD simulations (totaling 3000 frames per system) were conducted. Consistent with Yang et al.,^[^ ^21^ ^]^ our simulations showed that DCA‐3P formed key H bonds with Arg82, Arg86, and Arg95, supporting its agonist activity. In contrast, these interactions were not formed by compounds **2** and **16**, correlating with their lack of activity at hX4. Interestingly, in our simulations, DCA‐3P did not engage Arg95, and no salt bridge was observed between its carboxylic group and Arg241, previously reported results.^[^ ^21^ ^]^ Conversely, compounds **2** and **16** exhibited transient interactions between their C‐terminal functional groups (carboxylate or sulfate) and Arg241, yet without inducing receptor activation. A summary of the H‐bond occupancies >30% (averaged across replicas) for DCA‐3P, **2**, and **16** is reported in **Table** **4**, while all mean values of the recorded H bond occupancies are reported in Table S2 (Supporting Information).

**Table 4:** Mean h‐bond occupancy (%) calculated on the three replica MD simulations of hX4 in complex with agonist DCA‐3P and compounds **2** and **16**. Only the interactions showing a value higher than 30%, are reported.

Compound	hX4 activity	H‐bond occupancy [%]						
		Arg82	Arg86	Lys96	Arg159	Arg241	Asn245	Tyr254
**DCA‐3P**	Agonist	99.5	97.7	65.5	41.4	–	14.9	62.3
**2**	Not active	10.2	3.7	1.6	6.1	33.3	36.1	23.6
**16**	Not Active	0.4	2.5	57.0	0.2	76.8	15.0	0.8

**Figure 9:**
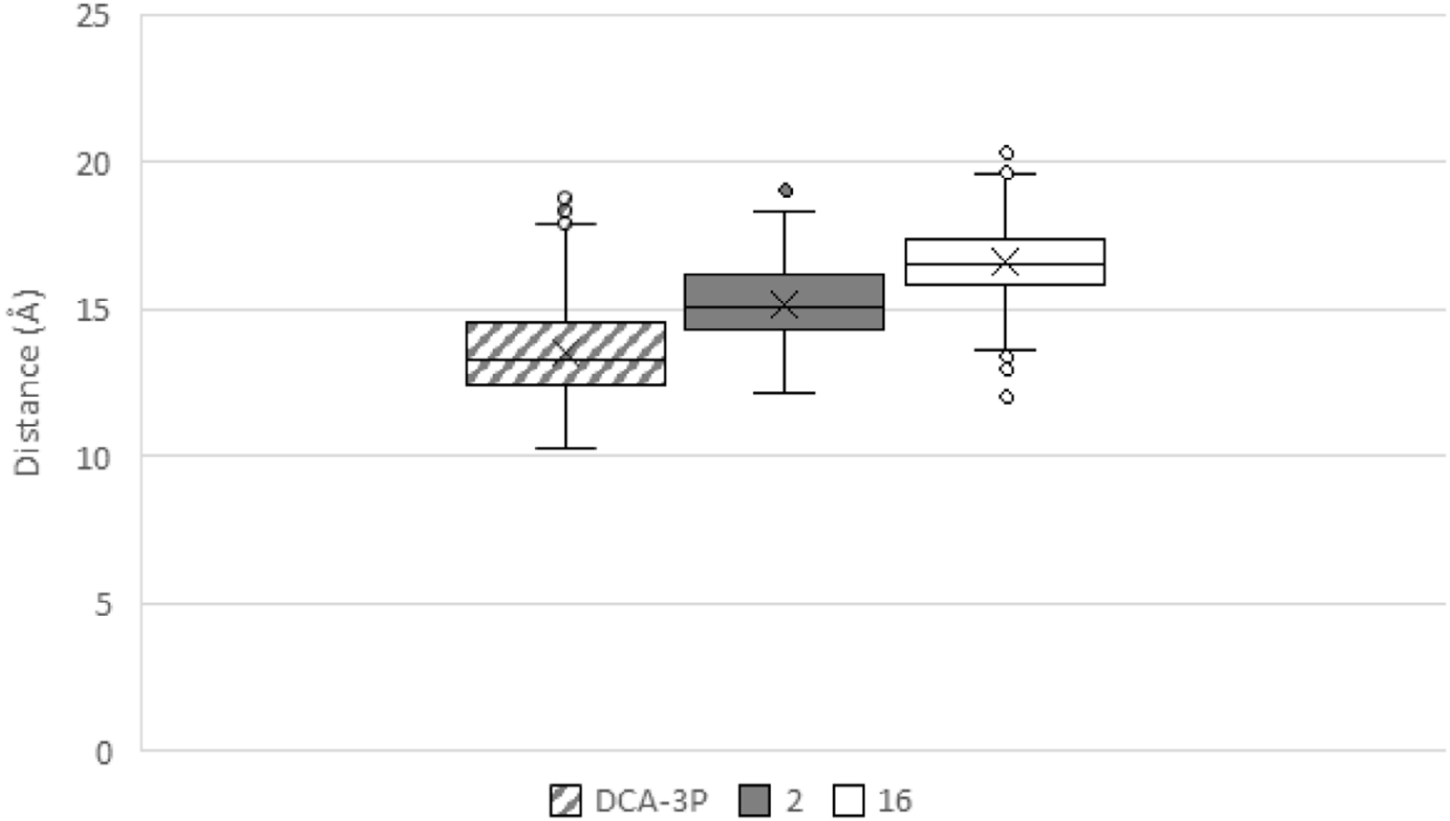
Box plot of the distances between the C*α* carbon of G229 and the carbon atom of the carboxylic group for DCA‐3P and **2**, or the sulfur atom of the sulfate group for **16**.

As highlighted by Yang et al.,^[^ ^21^ ^]^ DCA‐3P, like other GPCR agonists, reaches the toggle switch residue G229 in hX4, which corresponds to W336 in 5‐HT2AR,^[^ ^37^ ^]^ a conserved residue also involved in activation of other class A GPCRs such as A2AR. and *β*2AR.^[^ ^38^ ^,^ ^39^ ^]^ To evaluate this feature in our systems, we monitored the distance between the C*α* atom of G229 and either the carboxylic carbon (for DCA‐3P and compound **2**) or the sulfur atom of the sulfate group (for compound **16**). The resulting box plots (**Figure** **9**) clearly show that compounds **2** and **16** remain at significantly greater distances from G229 compared with DCA‐3P, supporting the conclusion that these compounds are unable to trigger the conformational rearrangements associated with hX4 activation. Additionally, for each ligand–receptor complex, 3D snapshots of the frames exhibiting the highest ligand–receptor interaction energy are provided in Figure S25 (Supporting Information), offering further structural insight into the distinct binding modes of DCA‐3P, **2**, and **16**.

## 3 Conclusions

Recent findings on the FXR receptor have highlighted the potential of partial agonists to elicit graded transcriptional and pharmacological responses.^[^ ^15^ ^,^ ^19^ ^]^ These ligands, by modulating receptor conformational dynamics, influence the interaction of FXR with specific co‐regulators. Unlike full agonists, which promote the binding of co‐activators and release of co‐repressors, partial agonists retain co‐repressor interactions while still recruiting co‐activators. Such selective modulation is governed by distinct molecular determinants that ultimately impact efficacy, specificity, and downstream gene regulation, thereby expanding the therapeutic scope of FXR‐targeting compounds.

In this study, we aimed to mitigate the side effects associated with FXR over‐activation by full agonists, such as cholesterol accumulation. To this end, we synthesized and evaluated a series of novel OCA derivatives bearing structural modifications at both the C3*α*‐position and the side chain. Our data provide for the first time compelling evidences that partial FXR agonism can be achieved through rational modification of the full agonist OCA (**1**). These findings are in line with previous observations, supporting the notion that the FXR LBD can adopt multiple conformations beyond the traditional active/inactive binary model. The structure of the ligand dictates the conformational landscape of the LBD, which in turn influences receptor activity and target gene expression.

A noteworthy implication of our study concerns relationship between C3‐modified BAs and hX4‐mediated cholestatic pruritus. Specifically, compounds **2** and **16** were able to activate FXR without triggering hX4‐mediated signaling, suggesting that structural features responsible for partial agonism may concurrently prevent off‐target pruritic effects. This opens new avenues for combination therapies or the development of dual‐action agents that target both BA and itch signaling.

Overall, this work sheds new light on the SAR of BAs as FXR ligands, leading to the identification of novel partial agonists with high potency and a favorable pharmacological profile. Compounds **2** and **16** demonstrated partial modulation of FXR and its downstream genes, differentiating them from the full endogenous agonists CDCA and OCA (**1**), and potentially reducing FXR‐related adverse effects. Importantly, these compounds were metabolically stable, devoid of activity at steroid receptors, of cytotoxicity at high concentrations, further supporting their translational relevance.

While the in vitro data presented here are promising, the efficacy of compounds **2** and **16** remains to be demonstrated in in vivo models of cholestasis or other chronic liver diseases. This represents an important next step to fully assess their therapeutic potential and safety profile in physiologically relevant conditions. Addressing this aspect in future studies will be crucial to fully validate the translational applicability of these partial FXR agonists. In conclusion, the partial agonist profile of compounds **2** and **16** positions them as promising chemical tool for in vivo studies and as valuable leads for the development of safer FXR modulators. Their ability to fine‐tune FXR activity may offer a promising strategy for treating metabolic disorders while minimizing mechanism‐based side effects commonly associated with full FXR activation.

## 4 Experimental Section

4.1

4.1.1

##### Chemistry: General Methods

Unless otherwise noted, chemicals were obtained from commercial suppliers and used without further purification. Reactions requiring anhydrous conditions were conducted in dried glass apparatus under a positive pressure of argon, using freshly distilled solvent according to reported procedures. NMR spectra were recorded on Brucker Ascend 600 MHz (superconducting magnet) or Bruker AC 400 MHz (Bruker, Madison, WI, USA). Chemical shifts (*δ*) are reported in parts per million (ppm) and are relative to CDCl_3_ (7.26 ppm and 77.0 ppm), CD_3_OD (3.31 ppm and 49.0 ppm) or DMSO‐d_6_ (2.50 ppm and 39.5 ppm). The abbreviations used are as follows: s, singlet; brs, broad singlet; d, doublet; dd, double of doublets; dt, doublet of triplets; t, triplet; q, quartet; qui, quintet; m, multiplet; and brm, broad multiplet. Coupling constants (J) are reported in Hertz (Hz). Thin‐layer chromatography (TLC) was performed on aluminum backed silica plates or RP‐18 glass plates (silica gel 60 F254, Merck, Darmstadt, Germany). Spots were visualized by UV detector (*λ*: 254 nm) and/or by staining and warming with phosphomolybdic acid (5% *w*/*v* solution in EtOH). When required, flash chromatographic purifications were performed using Biotage Isolera (Biotage AB, Uppsala, Sweden). Purity of the synthesized compounds (>95%) was assessed by quantitative NMR analysis (qNMR) in the presence of dimethyl sulfone (standard for quantitative NMR, TraceCERT, Merck KGaA, Darmstadt, Germany, Lot. n^o^.: BCBZ3940, declared purity: 99.82%) as the external standard (acquisition parameters: td, 65.536; ds, 0; ns, 2; sw, 19.8368; o1p, 6.175; o2p, 6.175; rg, 16). High‐resolution mass spectrometry (HRMS) measurements were performed on a UHPLC–MS/MS system consisting of an Agilent 1290 Infinity II module combined with an Agilent 6560 mass spectrometer (Agilent Technologies Inc., Santa Clara, CA, USA). The chromatographic separation was performed using a Zorbax Rrhd Eclipse Plus C18 column (50 mm × 2.1 mm, 1.8 µm, 95 Å, Agilent Technologies Inc.). HPLC eluent A was water (LC‐MS grade, LiChrosolv, Supelco, Merck KGaA, Darmstadt, Germany) with 0.01% (*v*/*v*) of HCO_2_H (LC‐MS grade, LiChropur, Supelco) and 0.1 mM HCO_2_NH_4_ (LC‐MS grade, LiChropur, Supelco), while eluent B was MeOH (LC‐MS grade, LiChrosolv, Supelco) with 0.1 mM HCO_2_NH_4_ (LC‐MS grade, LiChropur, Supelco). The chromatographic method followed the gradient listed below: 0–1 min, 20% B; 1–7 min, 20–97% B; 7–12 min, 97% B; 12–13 min, 97–20% B; 13–15 min, 20–50% B; followed by column reconditioning for 2 min. The column temperature was kept at 25 °C and the flow rate was 0.3 mL min^−1^. The injection volume was 5 µL. Samples were dissolved in MeOH (LC‐MS grade, LiChrosolv, Supelco) at 1 ppm concentration and sonicated for 5 min. For MS detection, the Dual AJS ESI source operated in negative ion mode. The gas temperature was set at 300 °C with a flow of 5 L min^−1^, while the sheath gas temperature was 350 °C with a flow of 11 L min^−1^. The nebulizer pressure was set at 35 psi, and the capillary and fragmentation voltages were 3500 V and 400 V, respectively. The Masshunter workstation data acquisition 10.0 (Agilent Technologies Inc., Santa Clara, CA, USA) program was used for data acquisition while the Masshunter qualitative analysis 10.0 (Agilent Technologies Inc., Santa Clara, CA, USA) software was used for data processing.

##### Synthesis of Compounds 2–17: 3*α*‐[((((*S*)‐1′‐Carboxy‐2′‐methylpropyl)carbamoyl)oxy)]‐7*α*‐hydroxy‐6*α*‐ethyl‐5*β*‐cholan‐24‐oic acid (2)

To a solution of benzyl 3*α*‐[(((4′‐nitrophenoxy)carbonyl)oxy)]‐7*α*‐hydroxy‐6*α*‐ethyl‐5*β*‐cholan‐24‐oate (**19**, 150 mg, 0.22 mmol) in dry DMF (5 mL), L‐valine (39 mg, 0.33 mmol) and Et_3_N (154 μL, 1.1 mmol) were added, and the resulting solution was stirred at 90 °C for 16 h. The mixture was allowed to cool to r.t., diluted with CH_2_Cl_2_ (30 mL) and washed with 3N HCl (30 mL). The aqueous phase was extracted with CH_2_Cl_2_ (2 x 20 mL), and the combined organic extracts were washed with H_2_O (20 mL), brine (20 mL), dried over anhydrous Na_2_SO_4_, and concentrated under reduced pressure. The crude was dissolved in dioxane (10 mL) and hydrogenated with palladium on carbon (10% loading, 30 mg) at 2.5 bar and r.t. for 16 h. The suspension was filtered on Celite, and the filtrate was concentrated under vacuo and purified by flash chromatography (Eluent: CH_2_Cl_2_/MeOH from 100:0 to 80:20, v/*v* + 0.05% AcOH). The title compound (**2**, 78 mg, 0.13 mmol, 60% yield, 97.8% purity by qNMR) was obtained as white solid. ^1^H‐NMR (400 MHz, CD_3_OD): *δ* 0.66 (3H, s, 18‐C*H*_*3*_), 0.90–1.07 (15H, m, 19‐C*H*_*3*_ + 6‐CH_2_C*H*_*3*_ + 21‐C*H*_*3*_ + (C*H*_*3*_)_2_CHCH), 3.70 (1H, s, 7*β*‐C*H*), 4.31 (1H, brs, (C*H*_*3*_)_2_CHC*H*), 4.21–4.23 (1H, m, 3*β*‐C*H*), 5.21–5.23 (m, 1H, N*H*). ^13^C‐NMR (100 MHz, CD_3_OD): *δ* 11.6, 11.7, 17.3, 18.2, 19.0, 20.7, 22.1, 23.1, 23.4, 23.9, 27.4, 28.2, 29.7, 30.7, 30.9, 33.1, 35.2, 35.3, 35.5, 39.9, 40.6, 41.1, 42.7, 45.0, 50.2, 54.7, 58.2, 70.9, 75.7, 155.6, 174.0, 179.4. HRMS *m*/*z* [M ‐ H]^−^ calcd. for C_32_H_53_NO_7_: 563.3822, found: 563.3818, Δppm: −0.78.

##### 3*α*‐[((((*R*)‐1′‐Carboxy‐2′‐methylpropyl)carbamoyl)oxy)]‐7*α*‐hydroxy‐6*α*‐ethyl‐5*β*‐cholan‐24‐oic acid (3):

Synthesized according to the procedure reported for compound **2** using D‐valine in 48% yield from **19** (white solid, 96.8% purity by qNMR). ^1^H‐NMR (400 MHz, CD_3_OD): *δ* 0.70 (3H, s, 18‐C*H*_*3*_), 0.83‐1.02 [15H, m, 19‐C*H*_*3*_ + 6‐CH_2_C*H*_*3*_ + 21‐C*H*_*3*_ + (C*H*_*3*_)_2_CHCH], 3.67 (1H, s, 7*β*‐C*H*), 3.99‐4.08 [1H, m, (C*H*_*3*_)_2_CHC*H*], 4.22‐4.42 (1H, m, 3*β*‐C*H*). ^13^C‐NMR (100 MHz, CD_3_OD): *δ* 10.6, 10.8, 16.8, 17.4, 18.2, 20.6, 22.0, 22.2, 23.1, 26.9, 27.9, 29.7, 30.4, 30.6, 30.9, 33.1, 35.0, 35.3, 35.4, 39.6, 40.1, 41.7, 43.3, 45.4, 50.3, 56.0, 59.3, 69.7, 75.6, 157.5, 174.0, 176.8. HRMS *m*/*z* [M ‐ H]^−^ calcd. for C_32_H_53_NO_7_: 563.3822, found: 563.3820, Δppm: −0.28.

##### 3*α*‐[((((*S*)‐1′‐Carboxy‐3′‐methylbutyl)carbamoyl)oxy)]‐7*α*‐hydroxy‐6*α*‐ethyl‐5*β*‐cholan‐24‐oic acid (4):

Synthesized according to the procedure reported for compound **2** using L‐leucine in 70% yield from **19** (white solid, 95.6% purity by qNMR). ^1^H‐NMR (400 MHz, CDCl_3_): *δ* 0.66 (3H, s, 18‐C*H*_*3*_), 0.87‐1.01 [17H, m, 19‐C*H*_*3*_ + 6‐CH_2_C*H*_*3*_ + 21‐C*H*_*3*_ + (C*H*_*3*_)_2_CHC*H*_*2*_], 3.72 (1H, s, 7*β*‐C*H*), 4.30‐4.55 (1H, m, 3*β*‐C*H*), 5.01‐5.15 [1H, m, (C*H*_*3*_)_2_CHCH_2_C*H*)], 5.79‐6.06 (1H, brs, N*H*). ^13^C‐NMR (100 MHz, CDCl_3_): *δ* 11.7, 11.8, 18.3, 20.8, 21.8, 22.2, 22.9, 23.1, 26.7, 24.8, 26.9, 28.2, 30.8, 30.9, 33.2, 35.2, 35.4, 35.5, 39.6, 40.0, 41.2, 41.7, 42.8, 45.0, 50.5, 52.2, 55.7, 67.1, 70.9, 76.7, 156.2, 177.9, 180.0. HRMS *m*/*z* [M ‐ H]^−^ calcd. for C_33_H_55_NO_7_: 577.3979, found: 577.3977, Δppm: ‐0.25.

##### 3*α*‐[(((*S*)‐1′‐Carboxyethyl)carbamoyl)oxy)]‐7*α*‐hydroxy‐6*α*‐ethyl‐5*β*‐cholan‐24‐oic acid (5):

Synthesized according to the procedure reported for compound **2** using L‐alanine in 69% yield from **19** (98.2% purity by qNMR). ^1^H‐NMR (400 MHz, CD_3_OD): *δ* 0.70 (3H, s, 18‐C*H*_*3*_), 0.89–1.02 (9H, m, 19‐C*H*_*3*_ + 6‐CH_2_C*H*_*3*_ + 21‐C*H*_*3*_), 1.37 (3H, d, *J=* 8.00 Hz, C*H*_*3*_CHCO_2_H), 3.66 (1H, s, 7*β*‐C*H*), 4.15 (1H, q, *J=* 8.00 Hz, CH_3_C*H*CO_2_H), 4.23–4.45 (1H, m, 3*β*‐C*H*). ^13^C‐NMR (100 MHz, CDCl_3_): *δ* 12.0, 12.2, 18.0, 18.8, 22.0, 23.0, 23.4, 23.7, 24.6, 28.2, 29.3, 31.1, 32.3, 34.5, 36.4, 36.6, 36.8, 41.0, 41.5, 43.1, 43.7, 46.8, 50.6, 51.7, 57.3, 71.1, 77.0, 158.4, 176.7, 178.2. HRMS *m*/*z* [M ‐ H]^−^ calcd. for C_30_H_49_NO_7_: 535.3509, found: 535.3507, Δppm: −0.46.

##### 3*α*‐[(((*S*)‐1′‐Carboxy‐2′‐phenyl‐ethyl)carbamoyl)oxy)]‐7*α*‐hydroxy‐6*α*‐ethyl‐5*β*‐cholan‐24‐oic acid (6):

Synthesized according to the procedure reported for compound **2** using L‐phenylalanine in 52% yield from **19** (white solid, 97.6% purity by qNMR). ^1^H‐NMR (400 MHz, CD_3_OD): *δ* 0.71 (3H, s, 18‐C*H*_*3*_), 0.89‐0.93 (6H, m, 19‐C*H*_*3*_ + 6‐CH_2_C*H*_*3*_), 0.98 (3H, d, *J =* 6.42 Hz, 21‐C*H*_*3*_), 2.94 (1H, dd, *J*_*1*_ *=* 8.96 Hz, *J*_*2*_ *=* 13.6 Hz, C*H*_*2(a*)_‐Ph), 3.18 (1H, dd, *J*_*1*_ *=* 9.20 Hz, *J*_*2*_ *=* 13.5 Hz, C*H*_*2(b*)_‐Ph), 3.67 (1H, s, 7*β*‐C*H*), 4.28‐4.36 (2H, m, 3*β*‐C*H +* HO_2_C‐C*H*‐NHCO), 7.21‐7.30 (m, 5H, C_6_ *H*_*5*_). ^13^C‐NMR (100 MHz, CD_3_OD): *δ* 10.6, 10.8, 17.3, 20.5, 21.5, 22.0, 22.2, 23.1, 26.6, 27.8, 29.5, 30.6, 30.9, 32.9, 34.8, 35.1, 35.3, 37.4, 39.5, 40.0, 41.6, 42.2, 45.3, 50.1, 55.8, 69.6, 75.4, 126.7, 127.8, 128.9, 137.4, 156.8, 175.0, 176.9. HRMS *m*/*z* [M ‐ H]^−^ calcd. for C_36_H_53_NO_7_: 611.3822, found: 611.3818, Δppm: ‐0.73.

##### 3*α*‐[((((*S*)‐1’‐Carboxy‐3’‐methyl‐butyl)carbamoyl)oxy)]‐7*α*‐hydroxy‐6*α*‐ethyl‐5*β*‐cholan‐24‐oic acid (7):

Synthesized according to the procedure reported for compound **2** using L‐isoleucine in 55% yield from **19** (white solid, 96.9% purity by qNMR). ^1^H‐NMR (400 MHz, CD_3_OD): *δ* 0.70 (3H, s, 18‐C*H*_*3*_), 0.85‐1.03 (17H, m, 19‐C*H*_*3*_ + 6‐CH_2_C*H*_*3*_ + 21‐C*H*_*3*_ + C*H*_*3*_C*H*_*2*_CHC*H*_*3*_), 3.66 (1H, s, 7*β*‐C*H*), 4.06‐4.10 (1H, m, NHC*H*CO_2_H) 4.24‐4.40 (1H, m, 3*β*‐C*H*). ^13^C‐NMR (100 MHz, CD_3_OD): *δ* 10.5, 10.6, 10.8, 14.5, 17.4, 20.6, 22.0, 22.3, 23.2, 24.7, 26.8, 27.9, 29.7, 30.6, 30.9, 33.10, 35.0, 35.3, 35.4, 37.1, 39.6, 40.1, 41.7, 42.3, 45.4, 50.3, 56.0, 58.4, 69.7, 75.7, 157.4, 174.0, 176.8. HRMS *m*/*z* [M ‐ H]^−^ calcd. for C_33_H_55_NO_7_: 577.3979, found: 577.3976, Δppm: −0.43.

##### 3*α*‐[(((1′‐Carboxymethyl)carbamoyl)oxy)]‐7*α*‐hydroxy‐6*α*‐ethyl‐5*β*‐cholan‐24‐oic acid (8):

Synthesized according to the procedure reported for compound **2** using glycine in 43% yield from **19** (white solid, 95.6% purity by qNMR). ^1^H‐NMR (400 MHz, CD_3_OD): *δ* 0.71 (3H, s, 18‐C*H*_*3*_), 0.90–0.95 (6H, m, 19‐C*H*_*3*_ + 6‐CH_2_C*H*_*3*_), 0.98 (3H, d, *J=* 6.48 Hz, 21‐C*H*_*3*_), 3.67 (3H, s, 7*β*‐C*H* + NHC*H*_*2*_CO_2_H), 4.30–4.34 (1H, m, 3*β*‐C*H*). ^13^C‐NMR (100 MHz, CD_3_OD): *δ* 10.2, 10.4, 17.2, 20.2, 21.7, 21.9, 22.8, 26.4, 27.6, 29.3, 32.3, 32.6, 34.5, 34.6, 34.8, 35.5, 39.2, 39.7, 41.3, 41.9, 43.8, 45.1, 49.8, 55.8, 69.2, 74.9, 156.9, 175.5, 181.9. HRMS *m*/*z* [M ‐ H]^−^ calcd. for C_29_H_47_NO_7_: 521.3353, found: 521.3350, Δppm: −0.52.

##### 3*α*‐[(((*iso*‐Butyl)carbamoyl)oxy)]‐7*α*‐hydroxy‐6*α*‐ethyl‐5*β*‐cholan‐24‐oic acid (9):

Synthesized according to the procedure reported for compound **2** using *iso*‐butylamine in 66% yield from **19** (white solid, 98.8% purity by qNMR). ^1^H‐NMR (400 MHz, CD_3_OD): *δ* 0.71 (3H, s, 18‐C*H*_*3*_), 0.89–0.94 [12H, m, 19‐C*H*_*3*_ + 6‐CH_2_C*H*_*3*_ + (C*H*_*3*_)_2_CHCH_2_], 0.96 (3H, d, *J=* 6.72 Hz, 21‐C*H*_*3*_), 2.89 (2H, d, *J=* 4.00 Hz, (CH_3_)_2_CHC*H*_*2*_], 3.67 (3H, s, 7*β*‐C*H*), 4.26–4.37 (1H, m, 3*β*‐C*H*). ^13^C‐NMR (100 MHz, CD_3_OD): *δ* 12.0, 12.2, 18.8, 20.3 (2x), 22.0, 23.5, 23.7, 24.6, 28.2, 29.3, 30.0, 31.1, 32.0, 32.3, 34.5, 36.4, 36.7, 36.8, 41.0, 41.5, 43.1, 43.7, 46.8, 51.7, 57.4, 71.1, 76.5, 159.2, 178.2. HRMS *m*/*z* [M ‐ H]^−^ calcd. for C_31_H_53_NO_5_: 519.3924, found: 519.3918, Δppm: −1.16.

##### 3*α*‐[(((2′‐Morpholinoethyl)carbamoyl)oxy)]‐7*α*‐hydroxy‐6*α*‐ethyl‐5*β*‐cholan‐24‐oic acid (10):

Synthesized according to the procedure reported for compound **2** using 4‐(2‐ammonioethyl)morpholinium chloride in 32% yield from **19** (white solid, 97.7% purity by qNMR). ^1^H‐NMR (400 MHz, CDCl_3_): *δ* 0.66 (3H, s, 18‐C*H*_*3*_), 0.85–0.94 (9H, m, 19‐C*H*_*3*_ + 6‐CH_2_C*H*_*3*_ + 21‐C*H*_*3*_), 2.45–2.60 (4H, m, ‐CH_2_C*H*_*2*_NC*H*_*2*_CH_2_O‐), 3.19–3.48 (2H, m, C*H*_*2*_NHCO), 3.70–3.74 (5H, m, 7*β*‐C*H* + ‐C*H*_*2*_CH_2_NCH_2_C*H*_*2*_O‐), 4.41–4.46 (1H, m, 3*β*‐C*H*), 5.50 (1H, brs, N*H*). ^13^C‐NMR (100 MHz, CDCl_3_): *δ* 11.6, 11.8, 18.3, 20.7, 22.2, 22.6, 23.0, 23.7, 27.1, 28.2, 29.6, 30.0, 30.9, 31.5, 33.1, 35.2, 35.5 (2x), 39.6, 40.0, 41.2, 42.7 (2x), 45.0, 50.5, 52.9, 56.0, 57.5, 65.9, 70.7, 74.8, 156.4, 178.7. HRMS *m*/*z* [M ‐ H]^−^ calcd. for C_33_H_56_N_2_O_6_: 576.4138, found: 576.4126, Δppm: −2.19.

##### 3*α*‐(Maleyloxy)‐7*α*‐hydroxy‐6*α*‐ethyl‐5*β*‐cholan‐24‐oic acid (11):

A solution of 3*α*, 7*α*‐dihydroxy‐6*α*‐ethyl‐5*β*‐cholan‐24‐oic acid (OCA, **1**) (265 mg, 0.63 mmol) in *t*BuOH (15 mL) and concentrated H_2_SO_4_ (0.2 mL) was refluxed for 18 h. The mixture was allowed to cool to r.t. and concentrated under reduced pressure. The crude was dissolved in CH_2_Cl_2_ (20 mL) and washed with aqueous saturated solution of NaHCO_3_ (2 × 20 mL). The aqueous phase was extracted with CH_2_Cl_2_ (2 × 20 mL) and the combined organic extracts were washed with H_2_O (40 mL), brine (40 mL), dried over anhydrous Na_2_SO_4_, and concentrated under reduced pressure. The crude *t*‐butyl ester **20** (quantitative yield) was dissolved in dry pyridine (15 mL), and maleic anhydride (617 mg, 6.29 mmol) and *N, N*‐dimethyl aminopyridine (7 mg, 0.06 mmol) were added. Stirring was continued at reflux for 18 h, and then the mixture was diluted with CH_2_Cl_2_ (20 mL) and washed with 3 N HCl (40 mL). The aqueous phase was extracted with CH_2_Cl_2_ (2 × 20 mL), and the combined organic extracts were washed with H_2_O (40 mL), brine (40 mL), dried over anhydrous Na_2_SO_4_, and concentrated under reduced pressure. The crude was dissolved in CH_2_Cl_2_ (10 mL), and trifluoroacetic acid (2 mL) was added dropwise at 0 °C. The reaction mixture was allowed to warm to r.t. and stirred for further 4 h. The mixture was diluted with CH_2_Cl_2_ (20 mL) and washed with H_2_O (3 × 20 mL), brine (20 mL), dried over anhydrous Na_2_SO_4_, and concentrated under reduced pressure. The crude was purified by flash chromatography (Eluent: CH_2_Cl_2_/MeOH from 100:0 to 90:10, v/v), and the title compound **11** (63 mg, 0.12 mmol, 19% yield, 98.9% purity by qNMR) was obtained as white solid. ^1^H‐NMR (400 MHz, CDCl_3_): *δ* 0.67 (3H, s, 18‐C*H*_*3*_), 0.85–0.95 (9H, m, 19‐C*H*_*3*_ + 6‐CH_2_C*H*_*3*_ + 21‐C*H*_*3*_), 3.73 (1H, s, 7*β*‐C*H*), 4.69–4.75 (1H, m, 3*β*‐C*H*), 6.30–6.39 (2H, m, C*H* = CH‐CO_2_H). ^13^C‐NMR (100 MHz, CD_3_OD): *δ* 10.8, 11.0, 17.6, 20.7, 22.2, 22.4, 23.3, 26.4, 28.0, 29.3, 31.0, 31.2, 33.2, 35.1, 35.5, 35.6, 39.7, 40.3, 41.8, 42.5, 45.5, 50.4, 56.1, 69.8, 76.0, 123.5, 137.2, 166.0, 171.4, 177.4. HRMS *m*/*z* [M ‐ H]^−^ calcd. for C_30_H_46_O_7_: 518.3244, found: 518.3238, Δppm: −1.03.

##### 3*α*‐((3′‐Methylbutanoyl)oxy)‐7*α*‐hydroxy‐6*α*‐ethyl‐5*β*‐cholan‐24‐oic acid (12):

A solution of 3*α*, 7*α*‐dihydroxy‐6*α*‐ethyl‐5*β*‐cholan‐24‐oic acid (OCA, **1**) (220 mg, 0.52 mmol) in *t*BuOH (15 mL) and concentrated H_2_SO_4_ (0.2 mL) was refluxed for 18 h. The mixture was allowed to cool to r.t. and concentrated under reduced pressure. The crude was dissolved in CH_2_Cl_2_ (20 mL) and washed with aqueous saturated solution of NaHCO_3_ (2 × 20 mL). The aqueous phase was extracted with CH_2_Cl_2_ (2 × 20 mL), and the combined organic extracts were washed with H_2_O (40 mL), brine (40 mL), dried over anhydrous Na_2_SO_4_, and concentrated under reduced pressure. The crude *t*‐butyl ester **20** was dissolved in CH_2_Cl_2_ (15 mL), and dry pyridine (169 μL, 2.10 mmol) and *iso*‐valeryl chloride (192 μL, 1.57 mmol) were added. Stirring was continued at r.t. for 16 h, and then the mixture was diluted with CH_2_Cl_2_ (20 mL) and washed with 3 N HCl (40 mL). The aqueous phase was extracted with CH_2_Cl_2_ (2 × 20 mL), and the combined organic extracts were washed with H_2_O (40 mL), brine (40 mL), dried over anhydrous Na_2_SO_4_, and concentrated under reduced pressure. The crude was dissolved in CH_2_Cl_2_ (15 mL), and trifluoroacetic acid (3 mL) was added dropwise at 0 °C. The reaction mixture was allowed to warm to r.t. and stirred for further 3 h. The mixture was diluted with CH_2_Cl_2_ (20 mL) and washed with H_2_O (2 × 20 mL), brine (20 mL), dried over anhydrous Na_2_SO_4_, and concentrated under reduced pressure. The crude was purified by flash chromatography (Eluent: CH_2_Cl_2_/MeOH from 100:0 to 90:10, v/v), and the title compound **12** (174 mg, 0.35 mmol, 66% yield, 99.6% purity by qNMR) was obtained as white solid. ^1^H‐NMR (400 MHz, CDCl_3_): *δ* 0.67 (3H, s, 18‐C*H*_*3*_), 0.83–0.96 [15H, m, 19‐C*H*_*3*_ + 6‐CH_2_C*H*_*3*_ + 21‐C*H*_*3*_ *+* (C*H*_*3*_)_2_CHCH_2_CO]_,_ 3.73 (1H, s, 7*β*‐C*H*), 4.70–4.75 (1H, m, 3*β*‐C*H*), 6.31–6.40 (2H, m, C*H* = C*H*). ^13^C‐NMR (100 MHz, CDCl_3_): *δ* 11.6, 11.8, 18.2, 20.7, 22.2, 22.4 (2x), 23.1, 23.7, 25.7, 26.7, 28.1, 29.7, 30.8, 31.0, 33.2, 35.2, 35.3, 35.6, 39.6, 40.0, 41.2, 42.8, 43.9, 45.1, 50.5, 55.8, 70.8, 74.4, 172.9, 179.9. HRMS *m*/*z* [M ‐ H]^−^ calcd. for C_31_H_52_O_5_: 504.3815, found: 504.3809, Δppm: −1.17.

##### 3‐(3′‐Methylbutanamido)‐7*α*‐dihydroxy‐6*α*‐ethyl‐5*β*‐cholan‐24‐oic acid (13):

To a solution of methyl 3‐(3′‐methylbutanamido)‐7*α*‐dihydroxy‐6*α*‐ethyl‐5*β*‐cholan‐24‐oate (**22**, 110 mg, 0.21 mmol) in MeOH (2.5 mL), NaOH (50 mg, 1.25 mmol) was added and the resulting solution was stirred at 25 °C for 6 h. The mixture was concentrated under reduced pressure and the residue was redissolved in CH_2_Cl_2_ (20 mL) and acidified with 3 N aqueous solution of HCl. The organic phase was washed with H_2_O (20 mL), brine (20 mL), dried over anhydrous Na_2_SO_4_, and concentrated under reduced pressure. The crude was purified by automated flash chromatography on silica gel (Eluent: CH_2_Cl_2_/acetone from 100:0 to 90:10, v/v) affording the title compound **13** (93 mg, 0.18 mmol, 87% yield, 97.0% purity by qNMR) as whitish solid. ^1^H‐NMR (400 MHz, CDCl_3_): *δ* 0.65 (3H, s, 18‐C*H*_*3*_), 0.86–0.97 [15H, m, 19‐C*H*_*3*_ + 6‐CH_2_C*H*_*3*_ + 21‐C*H*_*3*_ *+* (C*H*_*3*_)_2_CHCH_2_CO)]_,_ 3.69 (1H, brs, 3*β*‐C*H*), 3.72 (1H, s, 7*β*‐C*H*), 5.38 (1H, brs, N*H*). ^13^C‐NMR (100 MHz, CDCl_3_): *δ* 11.5, 11.7, 18.2, 20.9, 22.0, 22.4, 23.6, 23.8, 24.6, 26.2, 27.8, 28.0, 30.9, 31.6, 32.5, 35.2, 35.9, 39.4, 39.9, 40.8, 41.3, 42.6, 44.5, 46.5, 50.3, 51.4, 55.6, 70.6, 171.5, 174.6. HRMS *m*/*z* [M ‐ H]^−^ calcd. for C_31_H_53_NO_4_: 503.3975, found: 503.3967, Δppm: −1.42.

##### 23‐(*R*)‐Methyl 3*α*‐[((((*S*)‐1′‐carboxy‐2′‐methylpropyl)carbamoyl)oxy)]‐7*α*‐hydroxy‐6*α*‐ethyl‐5*β*‐cholan‐24‐oic acid (14a) and 23‐(*S*)‐methyl 3*α*‐[((((*S*)‐1′‐carboxy‐2′‐methylpropyl)carbamoyl)oxy)]‐7*α*‐hydroxy‐6*α*‐ethyl‐5*β*‐cholan‐24‐oic acid (14b):

Synthesized according to the procedure reported for compound **2**. After purification by automated flash chromatography on silica gel (Eluent: CH_2_Cl_2_/MeOH from 100:0 to 90:10, v/*v* + 0.05% AcOH), the title compounds **14a** and **14b** were obtained as white solids in 69 and 65% yield, respectively. The absolute stereochemistry at C23 position was confirmed by comparison of ^1^H‐ and ^13^C‐NMR spectra with those of C23‐methyl derivatives of cholic acid reported in the literature.^[^ ^40^ ^]^

**14a:** 96.4% purity da qNMR. ^1^H‐NMR (600 MHz, DMSO‐d_6_): *δ* 0.63 (3H, s, 18‐C*H*_*3*_), 0.81–0.88 [12H, m, 19‐C*H*_*3*_ + 6‐CH_2_C*H*_*3*_ + (C*H*_*3*_)_2_CH + 21‐C*H*_*3*_], 1.00 (3H, d, *J=* 6.90 Hz, 23*α*‐C*H*_*3*_), 2.30–2.36 (1H, m, 23*β*‐C*H*), 3.53 (1H, s, 7*β*‐C*H*), 3.77–3.79 (1H, brs, O*H*), 4.10–4.11 (1H, m, HNC*H*CO_2_H), 4.20–4.28 (1H, brs, 3*β*‐C*H*), 6.95 (1H, brs, NH), 12.27 (1H, brs CO_2_ *H*). ^13^C‐NMR (150 MHz, DMSO‐d_6_): *δ* 12.1, 12.2, 16.8, 18.5, 18.6, 19.7, 20.8, 22.6, 23.4, 23.5, 27.3, 28.7, 29.5, 30.16, 30.24, 33.0, 34.0, 35.4, 35.6, 36.6, 41.6, 42.6, 45.6, 50.6, 56.8, 60.0, 68.7, 74.6, 156.6, 174.1, 178.7. HRMS *m*/*z* [M ‐ H]^−^ calcd. for C_33_H_55_NO_7_: 577.3979, found: 577.3974, Δppm: −0.77.

**14b:** 94.9% purity by qNMR. ^1^H‐NMR (600 MHz, DMSO‐d_6_): *δ* 0.59 (3H, s, 18‐C*H*_*3*_), 0.81–0.85 [9H, m, 19‐C*H*_*3*_ + 6‐CH_2_C*H*_*3*_ + (C*H*_*3*_)_2_CH], 0.90 (3H, d, *J=* 5.8 Hz, 21‐C*H*_*3*_), 1.06 (3H, d, *J=* 6.70 Hz, 23*β*‐C*H*_*3*_), 2.36–2.45 (1H, m, 23*α*‐C*H*), 3.50 (1H, s, 7*β*‐C*H*), 3.73 (1H, brs, O*H*), 4.08 (1H, brs, HNC*H*CO_2_H), 4.23–4.29 (1H, brs, 3*β*‐C*H*), 6.65 (1H, brs, NH), 12.79 (1H, brs CO_2_ *H*). ^13^C‐NMR (150 MHz, DMSO‐d_6_): *δ* 12.1, 18.4, 19.0, 19.4, 19.9, 20.8, 22.6, 23.4, 23.5, 27.2, 28.4, 30.2, 33.1, 34.7, 35.4, 35.6, 37.2, 41.1, 41.6, 42.5, 45.6, 50.6, 56.8, 60.4, 68.7, 74.5, 156.4, 174.4, 178.3. HRMS *m*/*z* [M ‐ H]^−^ calcd. for C_33_H_55_NO_7_: 577.3979, found: 577.3975, Δppm: −0.56.

##### 3*α*‐[((((*S*)‐1′‐Carboxy‐2′‐methylpropyl)carbamoyl)oxy)]‐7*α*‐hydroxy‐6*α*‐ethyl‐5*β*‐cholan‐24‐*nor*‐23‐oic acid (15):

Synthesized from intermediate **29** according to the two‐step procedure reported for compound **2**. After purification by automated flash chromatography on silica gel (Eluent: CH_2_Cl_2_/MeOH from 100:0 to 90:10, v/*v* + 0.05% AcOH), the title compound **15** was obtained as white solid in 54% yield (97.2% purity by qNMR). ^1^H‐NMR (CD_3_OD, 400 MHz): *δ* 0.74 (3H, s, C*H*_3_), 0.85–1.06 [15H, m, 19‐C*H*_*3*_ + 6‐CH_2_C*H*_*3*_ + 21‐C*H*_*3*_  + (C*H*_*3*_)_2_CH], 2.39–2.46 (1H, m, 22‐C*H*_*2(a)*_), 3.66 (1H, s, 7*β*‐C*H*), 4.01–4.08 (1H, m, HNC*H*CO_2_H), 4.28–4.41 (1H, m, 3*β*‐C*H*). ^13^C‐NMR (CD_3_OD, 100 MHz): *δ* 12.0, 12.2, 18.2, 19.6, 20.0, 22.0, 23.4, 23.7, 24.5, 28.1, 29.4, 31.1, 31.8, 34.5, 35.0, 36.4, 36.7, 40.9, 41.5, 43.1, 43.8, 46.8, 51.7, 57.4, 60.7, 71.1, 77.0, 158.8, 175.6, 177.5. HRMS *m*/*z* [M ‐ H]^−^ calcd. for C_31_H_51_NO_7_: 549.3666, found: 549.3663, Δppm: −0.39.

##### 3*α*‐[((((*S*)‐1′‐Carboxy‐2′‐methylpropyl)carbamoyl)oxy)]‐7*α*‐hydroxy‐6*α*‐ethyl‐5*β*‐cholan‐24‐*nor*‐23‐sulfate triethylammonium salt (16):

Synthesized from intermediate **31** according to the procedure reported for **2**. At the end of the reaction, 3 N aqueous solution of HCl (pH = 5) and acetone were added at ‐10 °C and the pale‐yellow precipitate thus formed (triethylammonium salt) was filtered off, washed with cold acetone, and dried under vacuo. The crude was purified by RP‐18 chromatography (Eluent: H_2_O/MeOH from 90:10 to 20:80, v/v) affording the title compound **16** as pale‐yellow solid (54% yield, 96.0% purity by qNMR). ^1^H‐NMR (CD_3_OD, 400 MHz): *δ* 0.71 (3H, s, C*H*_3_), 0.89–0.94 (9H, m, 19‐C*H*_*3*_ + 6‐CH_2_C*H*_*3*_, 21‐C*H*_*3*_), 0.97 (3H, d, *J=* 7.30 Hz, CH_3_CHC*H*_*3*_), 1.00 (3H, d, *J=* 7.30 Hz, C*H*_*3*_CHCH_3_), 1.32 [9H, t, *J=* 8.00 Hz, (C*H*_*3*_CH_2_)_3_NH^+^], 3.22 [6H, t, *J=* 8.00 Hz, (CH_3_C*H*_*2*_)_3_NH^+^], 3.66 (1H, s, 7*β*‐C*H*), 3.98–4.10 (3H, m, 23‐C*H*_*2*_SO_3_H + HNC*H*CO_2_H), 4.27–4.41 (1H, m, 3*β*‐C*H*). ^13^C‐NMR (CD_3_OD, 100 MHz): *δ* 10.6, 10.8, 16.8, 17.8, 18.2, 20.6, 22.1, 22.3, 22.8, 23.2, 26.8, 28.0, 30.4, 32.8, 33.1, 35.0, 35.1, 35.3, 39.6, 40.1, 41.7, 42.4, 45.4, 46.6, 50.3, 54.7, 56.4, 59.3, 65.9, 69.7, 75.7, 157.5, 174.1. HRMS *m*/*z* [M ‐ H]^−^ calcd. for C_31_H_53_NO_9_S: 615.3441, found: 615.3436, Δppm: −0.78.

##### 3*α*‐[((((*S*)‐1′‐Carboxy‐2′‐methylpropyl)carbamoyl)oxy)]‐7*α*‐hydroxy‐6*α*‐ethyl‐5*β*‐cholan‐22,23‐*bisnor‐*cholan‐(1′, 2′, 4′‐oxadiazol‐5‐one) (17):

Synthesized from intermediate **33** according to the procedure reported for **2**. After purification by automated flash chromatography on silica gel (Eluent: CH_2_Cl_2_/MeOH from 100:0 to 80:20, v/*v* + 0.05% AcOH), the title compound **17** was obtained as white solid in 52% yield (96.5% purity by qNMR). ^1^H‐NMR (400 MHz, CD_3_OD): *δ* 0.75 (3H, s, 18‐C*H*_*3*_), 0.87–1.01 [15H, m, 19‐C*H*_*3*_ + 6‐CH_2_C*H*_*3*_ + 21‐C*H*_*3*_ + CH(C*H*_*3*_)_2_], 2.65–2.70 (1H, m, *H‐*isoxazol‐3‐one), 3.67 (1H, s, 7*β*‐C*H*), 4.04 (1H, d, *J=* 5.45 Hz, HN‐C*H*‐CO_2_H), 4.30–4.36 (m, 1H, 3*β*‐C*H*). ^13^C‐NMR (100 MHz, CD_3_OD): *δ* 10.8, 11.0, 17.0, 18.0, 18.4, 20.7, 22.2, 22.5, 23.4, 27.0, 28.2, 29.9, 30.6, 31.6, 33.3, 34.5, 35.2, 35.5, 39.6, 40.3, 41.9, 42.7, 45.6, 50.5, 56.3, 59.5, 69.8, 75.8, 157.7, 159.5, 161.1, 174.4. HRMS *m*/*z* [M ‐ H]^−^ calcd. for C_32_H_51_N_3_O_7_: 589.3727, found: 589.3718, Δppm: −1.51.

##### Biology: AlphaScreen Assays

Activation of the FXR receptor was determined using a recruitment coactivator assay, namely, AlphaScreen technology. Briefly, assays were conducted in white, low volume, 384‐well ProxyPlate using a final volume of 10 μL containing 10 nM glutathione transferase‐tagged *h*FXR LBD protein and 30 nM biotinylated Src‐1 peptide. The stimulation was carried out with different BA derivatives concentrations for 30 min at 25 °C. Luminescence was read in an EnVision 2103 microplate analyzer (PerkinElmer, USA) after incubation with the detection mix (acceptor and donor beads) for 4 h at 25 °C in the dark. Dose‐response curves were conducted in triplicate.

##### Nuclear Receptor Selectivity

For all nuclear receptors tested, the assay was performed in white low‐volume, 384‐well ProxiPlates (PerkinElmer) using a final volume of 15 µL per well containing the related GST‐NR‐LBD protein and its biotinylated coactivator peptide at specific, previously optimized, concentrations. GST‐NR‐LBD protein and its biotinylated coactivator peptide were incubated with dose response of compounds from 0.001 μM to 100 μM for 1 at room temperature, and then a detection mix of acceptor and donor beads was added, and after 4 h of incubation at room temperature, luminescence was read at EnVision reader.

##### Cell Culture

Hek293T/17 human embryo kidney tissue cells (RRID:CVCL_1926, CRL‐11,268, ATCC) were cultured in DMEM medium supplemented with 1% penicillin/streptomycin, 1% L‐glutamine, and 10% fetal bovine serum (Life Technologies). HepG2 human hepatocellular carcinoma cells (RRID:CVCL_0027, HB‐8065, ATCC) were cultured in E‐MEM medium supplemented with 1% penicillin/streptomycin, 1% L‐glutamine, and 10% fetal bovine serum (Life Technologies). NCI‐H716 human colorectal adenocarcinoma cells (RRID:CVCL_1581, CCL‐271, ATCC) cells were maintained in suspension in RPMI‐1640 medium supplemented with 10% fetal calf serum, 10 mM HEPES, and 1 mM sodium pyruvate. CHO‐K1 cells hamster Chinese ovary cells (RRID:CVCL_0214, CCL‐61, ATCC) were maintained in ATCC‐formulated F‐12K Medium (ATCC 30–2004) supplemented with fetal bovine serum (#ECS0180D, Euroclone) to a final concentration of 10%. Cells were grown at 37 °C in 5% CO_2_. Cells were grown at 37 °C in 5% CO_2_.

##### Detection of Intracellular cAMP Levels

Activation of TGR5 was evaluated by cell based ‐FRET assay using NCI‐H716 cells (RRID:CVCL_1581, CCL‐271, ATCC). Cells were cultured on 96‐well plates coated with Matrigel (BD Biosciences) in DMEM supplemented with 10%FCS, after 24 h, stimulated with increasing concentrations of compounds for 60 min at 37 °C in OptiMEM containing 1 mM 3‐isobutyl‐1‐methylxanthine. The intracellular cAMP level was measured with Lance kit according to the manufacturer's protocol (PerkinElmer) and the plate was read at En Vision reader.

##### Plasmids

pGL4.23, pGL4.74 and pGEM were from Promega. Human FXR full length (NM_001206979.2) was cloned NotI‐Sma into the pCMV sport4 vector. Human RXR*α* full length (NM_002957) was cloned EcoRI‐BglII into pSG5 plasmid. The FXR RE IR1 repeated 3 times has been cloned KpnI‐BglII into pGL4.23, upstream the luciferase gene. pGL4.74 [hRluc/TK] vector encodes the luciferase reporter gene *Renilla reniformis* and it has been used as an expression control.

##### Transient Transfection and Transactivation

Hek293T/17 cells (RRID:CVCL_1926, CRL‐11,268, ATCC) were seeded in a 96‐well plate (Optiplate PerkinElmer) at 15 x 10^3^/well, the day after seeding, the cells were transfected with 100 ng of reporter vector (pGL4.23 IR1X3‐Luc) and 10 ng of both pCMVsport4 hFXR and pSG5 hRXR. As transfection normalization 20 ng of pGL4.74 has been used. The pGEM vector was added to reach the amounts of DNA transfected in each well up to 2000 ng. All transfections were performed using FuGENE HD (Promega) according to the manufacturer's protocol. Increasing concentrations of compounds from 0.01 μM to 300 μM in triplicate were used to stimulate transfected cells for 18 h. After stimulation the cells were lysed with Dual‐Glo luciferase assay system (Promega) and luminescence was read using Victor V instrument (PerkinElmer). Nonlinear regression curve fit analysis has been performed using GraphPad 5.0 software.

##### Quantitative Real‐Time PCR

HepG2 cells (RRID:CVCL_0027, HB‐8065, ATCC) were plated 150,000/well in 24 well plate. The day after plating, HepG2 cells were treated with CDCA (50 μM), OCA (10 μM), compound **16** (10 or 50 μM) and compound **2** (10 or 50 μM) for 16 h. DMSO normalization at 0.1% has been performed. The mRNA expression level of FXR target genes was measured by Real‐Time Polymerase Chain Reaction (Q‐RTPCR). Total RNA was isolated (RNAeasy Kit Qiagen) from HepG2 cells stimulated with one fixed concentration of compounds for 18 h. The RNA was random reverse‐transcribed with SuperScript IV VILO (ThermoFisher) in 10 μL reaction volume. 10 ng template was used in 20 μL final volume reaction of real‐time PCR containing 0.3 μM of each primer and 10 μL of 2X SYBR Green PCR Master MIX (Bio‐Rad). All reactions were performed in triplicate and the thermal cycling conditions were: 3 min at 95 °C, followed by 45 cycles of 95 °C for 10 s, and 60 °C for 30 s in iCycler iQ5 instrument (Bio‐Rad, Hercules, CA). The mean value of the replicates for each sample was calculated and expressed as cycle threshold (CT: cycle number at which each PCR reaction reaches a predetermined fluorescence threshold, set within the linear range of all reactions). The amount of gene expression was then calculated as the difference (ΔCT) between the CT value of the sample for the target gene and the mean CT value of that sample for the endogenous control. Relative expression was calculated as the difference (ΔΔCT) between the ΔCT values of the test sample and of the control sample (WT) for each target gene. The relative quantization value was expressed and shown as 2‐ΔΔCT. All PCR primers were intron spanning designed using the software Beacon Designer on published sequence data from the NCBI database and are indicated in Table S3 (Supporting Information).

##### Cytotoxicity Assays

Cell viability and necrosis were evaluated by measuring ATP and LDH levels respectively. ATP levels were measured using Cell Titer Glo (Promega) whilst LDH levels were measured using Cytotx‐One (Promega), according to the manufacturer's instructions. 10 * 10^4^ HepG2 cells (RRID:CVCL_0027, HB‐8065, ATCC) were stimulated with increasing concentration of compounds in a white/clear 96‐well microplate for 4 h at 37 °C. LCA and tamoxifen were used as positive control of toxicity.

##### Label‐Free hX4 (MRGPRX4) Binding Assay

CHO‐K1 cell lines (RRID:CVCL_0214, CCL‐61, ATCC) were cultured in ATCC‐formulated F‐12K Medium (ATCC 30–2004) supplemented with fetal bovine serum (Euroclone #ECS0180D) to a final concentration of 10%. CHO‐K1 cells were transfected with the pCMV6‐XL5 MRGPRX4 (NM_054032) vector using a 6:1 ratio of FuGENE HD Transfection Reagent (Promega) to DNA content. Cells were plated at a density of 7000 cells per well in an Enspire 384‐well black plate Label‐free microplates with clear bottoms containing a patented optical biosensor integrated into each well (Corning#5444). Briefly, the Enspire with label‐free technology has a dedicated light source for exciting the microplate bottom, and changes in local index of refraction have been measured. These changes result from the ligand induced dynamic mass redistribution within the bottom region of the cell monolayer (cell‐based assays). These changes are detected as a picometer (pm) shift in the wavelength of the reflected light from the microplate. The results are reported as response (pm) showing the picometer shift between final (at the time of compound addition) and baseline (before compound addition) reads. After 48 h, cells were stimulated with test compounds in a 7‐point dose–response assay, performed in triplicate, using the HP‐Tecan instrument. Ligand‐induced MRGPRX4 activation was measured using the Enspire Instrument (PerkinElmer).

##### Metabolic Stability Study

BA derivatives were incubated (final concentration equal to 0.5 μM) with mouse (mouse (CD‐1) pooled microsomes, RRID: Addgene_MSMCPL, Gibco, ThermoFisher Scientific) and human microsomes (human pooled microsomes (50 donors), RRID: Addgene_HMMCPL, Gibco, ThermoFisher Scientific) supplemented with NADPH co‐factors at 37 °C. Aliquots were taken at 6 time‐points (0, 3, 6, 9, 15 and 30 min) in duplicates. At each time‐point, the reactions were terminated by the addition of acetonitrile and mobile phase. The samples were centrifuged, and the parent compound concentration was evaluated by LC‐MS/MS measurements. Dextromethorphan (Merck, Darmstadt, Germany) was used as standards. Zero‐time incubation was used as 100% value. The loss (as percentage) of substrate in incubation was determined to estimate in vitro half‐life and in vitro intrinsic clearance of compounds. Half‐life time (t_1/2_) of tested compound was calculated using the following equation.

(1) $$t_{1/2}\;=\;\ln2/k$$

where k represents the kinetic constant of disappearance and is the slope of linear regression of logarithmic concentration of test item or controls versus time.

The intrinsic clearance (CL_int_) was calculated as follows.

(2) $${\mathrm{CL}}_{\mathrm{int}}\;\left(\mu L\;\min^{-1}\times \mathrm{mg}\;\mathrm{protein}\right)\;=\;V\times 0.693/t_{1/2}$$

where V is the incubation volume ( µL)/microsomal protein ( mg).

Mouse intrinsic clearance classification ranges: CL_int_ < 8.8 μL min^−1^ mg^−1^ = low; 8.8 μL min^−1^ mg^−1^< CL_int_ < 48 μL min^−1^ mg^−1^ = medium; CL_int_ > 48 μL min^−1^ mg^−1^ = high.

The assay is accepted when the control used in the assay (dextromethorphan) has clearance≥ 10 µL min^−1^ mg^−1^ protein. In the assay conditions, dextromethorphan showed a half‐life = 9.62 ± 0.62 min along with a CL_int_ = 144.57 ± 9.22 μL min^−1^ mg^−1^ protein.

##### Computational Chemistry: Docking Analysis

The *Maestro* graphical user interface and the *LigPrep* module (Schrödinger, LLC, New York, NY, 2023) were used to build and assign a protomeric state to the investigated compounds. The PDB code 6HL1 was used as source for the FXR LBD structure in complex with CDCA.^[^ ^19^ ^]^ The complex was treated using the default settings of the *Protein Preparation Wizard* protocol of the Schrödinger Suite. Next, the *Glide SP* docking program was run to obtain the best ranked putative binding poses of all the investigated compounds. The grid box was centered on the co‐crystallized ligand CDCA. A similar protocol was applied to the PDB code 8KEX, the complex of hX4 with DCA‐3P, to be used for the docking of compounds **2** and **16**.

##### Molecular Dynamics Simulations

The docking complexes of compounds **1**, **2**, **14a/b**–**16** were used as input for the *System Builder* module in order to generate the systems to be submitted to the *Desmond Molecular Dynamics* package present in the Schrödinger Suite 2023–1 (Schrödinger, LLC, New York, NY, 2023). A total of 6 cases to be simulated in presence of SRC‐1 chain were set. All the simulations have been performed in order to generate 1000 frames during the 1 μs production run, with an integration time of 2 fs at 298 K. The complexes were firstly solvated with water TIP3P and neutralized with the proper counterions using the *System Builder* module. The box used was orthorhombic, having each side at a minimum distance of 10 Å from any atom of the complex. The force field used was the OPLS4e in an NPT (isothermal–isobaric) ensemble, and the thermostat used was Berendsen type.^[^ ^41^ ^]^ All the other options were set to default values, thus the PBC conditions were applied to the simulated complexes. The dynamic protocol was composed by subsequent steps, with the first being a relaxing minimization, then a progressive increase of temperature to reach 300 K in 140 ps, and finally a production run of 1 μs. The MD trajectories were then analyzed using the *Simulation Interaction Diagram* tool. The hX4 complexes with DCA‐3P (pdb code: 8KEX), **2** and **16** (from docking output) have been used to build the system by placing the GPCR inside the POPC phospholipid bilayer using the *System Builder* module. The complexes were then solvated with water TIP3P and the whole system was neutralized using sodium chloride (NaCl) set to an ionic strength of 0.15 M. Three replicas of 300 ns have been performed. The relaxation protocol was modified to include five steps in the equilibrium or relaxation stage. The first step consisted of Brownian dynamics (NVT, with T = 10) with restraints on solute‐heavy atoms for 1 ns; the second step included T = 100 K, an H_2_O barrier, Brownian NPT, and the membrane restrained in the Z‐direction and protein restrained, for 100 ps. The third step consisted of NPgT, heating from 100 K to 300 K, an H_2_O barrier, and gradual release of restraints over a 300 ps simulation. The fourth step included NVT at T = 300 K with no restraints for 500 ps. The final step was run with NPT at T = 300 K with no restraints for 5 ns. After the equilibration stage, each production run was 300 ns with a time‐step of 2 fs at 300 K.

## Conflict of Interest

A.G. and R.P. are cofounder of Tes Pharma.

## Author Contributions

The manuscript was written through contributions of all authors. All authors have given approval to the final version of the manuscript. **Bruno** **Cerra** and **Andrea Carotti** contributed equally on this work.

## Supporting information

Supplementary Material

## Data Availability

The data that support the findings of this study are available in the supplementary material of this article.
