# Supplementary material for: Structure‐Based Discovery of Obeticholic Acid Derivatives as Novel Farnesoid X Receptor Partial Agonists with Improved Selectivity and Reduced Off‐Target Effects
Source: ChemMedChem. 2026 Feb 4;21(2):e202500960. doi: 10.1002/cmdc.202500960 (PMC12872208; doi:10.1002/cmdc.202500960)
Supplement: Supplementary file 1 — Supplementary Material [file CMDC-21-e202500960-s001.pdf]

Supporting Information

Structure-based Discovery of OCA Derivatives as Novel FXR Partial Agonists with Improved Selectivity and Reduced Off-target Effects

Daniela Passeri,[1] *Bruno Cerra,*[2,‡] *Andrea Carotti*[2,‡] *Francesco Greco,*[1] *Sara Piermarini,*[2] *Carolina Colliva,*[1] *Paride Liscio,*[1] *Francesca De Franco,*[1] *Luciano Adorini,*[3] *Mary Erickson,*[3] *Roberto Pellicciari,*[1] *Antimo Gioiello*[2,]*

[1] Tes Pharma, Via Giovine Italia 1, 06073, Solomeo (PG), Italy.

[2] Department of Pharmaceutical Sciences, University of Perugia, Via del Liceo 1, 06123 Perugia, Italy.

[3] Intercept Pharmaceuticals, Inc., San Diego, CA, USA.

*To whom correspondence should be addressed. Antimo Gioiello, Laboratory of Medicinal and Advanced Synthetic Chemistry, Department of Pharmaceutical Sciences, University of Perugia, Via del Liceo 1, Perugia, Italy. E-mail address: antimo.gioiello@unipg.it. Tel :+39 075 585 2318/5182.

‡These authors contributed equally.

Contents

1. Synthesis of intermediates S1

2. NMR spectra S9

3. Purity data (qNMR in the presence of DMS as the external standard) S60

4. HRMS data S69

5. Metabolic stability study - LC-MS/MS analysis (Figures S1-S13, Table S1) S85

6. FXR molecular dynamics analyses (Figures S14-S20) S92

7. FXR coactivator (SRC-1) recruitment assay (Figure S21) and transactivation assay on Hek293T cells (Figure S22) of compounds **2** and **16** S99

8. *In vitro* cytotoxicity (Figure S23) and nuclear receptor selectivity (Figure S24) of compounds **2** and **16** S102

9. hX4 Molecular dynamics analyses (Table S2 and Figure S25) S103

10. Primers sequences for real-time PCR (Table S3) S104

11. References S105

1. Synthesis of intermediates

Benzyl 3α,7α-dihydroxy-6α-ethyl-5β-cholan-24-oate (18): To a solution of 3α,7α-dihydroxy-6α-ethyl-5β-cholan-24-oic acid (OCA, **1**) (4 g, 9.51 mmol) in MeCN (50 mL), Cs_2CO_3 (4.65 g, 14.26 mmol) and benzyl bromide (5.7 mL, 47.55 mmol) were added. Stirring was continued at reflux for 8 h. The mixture was evaporated to dryness and purified by flash chromatography (Eluent: CH_2Cl_2/MeOH from 100:0 to 95:5, v/v) affording the title intermediate **18** (4.8 g, 9.39 mmol, 98% yield) as white solid. ^{1}H-NMR ($CDCl_3$, 400 MHz): δ 0.63 (3H, s, CH_3), 0.89-0.99 (9H, m, 19-CH_3 + 21-CH_3 + 6-CH_2CH_3), 3.39-3.41 (1H, m, 3β-C*H*), 3.69 (1H, s, 7β-C*H*), 5.05-5.13 (2H, m, CO_2CH_2Ph), 7.32-7.35 (5H, m, Ph).

Benzyl 3α-[(((4'-nitrophenoxy)carbonyl)oxy)]-7α-hydroxy-6α-ethyl-5β-cholan-24-oate (19): To a solution of benzyl 3α,7α-dihydroxy-6α-ethyl-5β-cholan-24-oate (**18**, 4.5 g, 8.81 mmol) in dry pyridine (50 mL) was added *p*-nitrophenylchloroformate (3.5 g, 17.62 mmol) at 0 °C under argon atmosphere. Stirring was continued at r.t. for further 16 h, then the mixture was diluted with CH_2Cl_2 (200 mL) and washed with 1 N HCl (3 x 100 mL). The aqueous phase was extracted with CH_2Cl_2 (2 x 100 mL) and the combined organic extracts were washed with H_2O (200 mL), brine (200 mL), dried over anhydrous Na_2SO_4 and concentrated under reduced pressure. The crude was purified by flash chromatography (Eluent: petroleum ether/EtOAc from 100:0 to 60:40, v/v) affording the title intermediate **19** (4.8 g, 7.10 mmol, 81% yield) was obtained as whitish solid. ^{1}H-NMR ($CDCl_3$, 400 MHz): δ 0.67 (3H, s, CH_3), 0.91-0.95 (9H, m, 19-CH_3 + 21-CH_3 + 6-CH_2CH_3), 3.69 (1H, s, 7β-C*H*), 4.55-4.58 (1H, m, 3β-C*H*), 5.13-5.14 (2H, m, CO_2CH_2Ph), 7.29-7.41 (7H, m, Ph), 8.29 (2H, d, *J*= 9.06 Hz, Ph).

Methyl 3-oxo-7α-dihydroxy-6α-ethyl-5β-cholan-24-oate (21): To a solution of 3α,7α-dihydroxy-6α-ethyl-5β-cholan-24-oic acid (OCA, **1**) (400 mg, 0.95 mmol) in MeOH (10 mL), *p*TSA*H_2O (20 mg, 0.1 mmol) was added and the resulting mixture was irradiated at 25 °C under ultrasounds conditions. The mixture was concentrated under reduced pressure and the residue was dissolved in CH_2Cl_2 (25 mL) and washed with aqueous saturated solution of $NaHCO_3$ (25 mL) and H_2O (25 mL). The organic phase was concentrated under reduced pressure and the residue was dissolved in dry toluene and refluxed for 24 h in the presence of Fetizon's reagent (Ag_2CO_3 on Celite, 1.07 mmol/g, 2 g) using a Dean–Stark apparatus.[1] The suspension was filtered on a pad of Celite and concentrated under reduced pressure. The crude was filtered on a short pad of silica (Eluent: petroleum ether/EtOAc from 100:0 to 60:40, v/v) affording the desired intermediate **21** (346 mg, 0.80 mmol, 84% yield) as whitish solid. ^{1}H-NMR ($CDCl_3$, 400 MHz): δ 0.63 (3H, s, C*H*3), 0.89-0.99 (9H, m, 19-C*H*3 + 21-C*H*3 + 6-CH_2C*H*3), 2.71 (1H, t, *J*= 13.92 Hz), 3.69 (1H, s, 7β-C*H*).

Methyl 3-(3'-methylbutanamido)-7α-dihydroxy-6α-ethyl-5β-cholan-24-oate (22): To a solution of methyl 3-oxo-7α-dihydroxy-6α-ethyl-5β-cholan-24-oate (**21**, 300 mg, 0.69 mmol) in dry MeOH (15 mL), HCO_2NH_4 (535 mg, 6.93 mmol) and 7 M solution of NH_3 in MeOH (2 drops) were sequentially added at 25 °C. The mixture was stirred for 1 h at 25 °C and then NaCN•BH_3 (44 mg, 0.69 mmol) was added in one portion. The mixture was stirred at 25 °C for further 24 h. The volatiles were removed under reduced pressure and the residue was dissolved in CH_2Cl_2 (15 mL) and washed with H_2O (3 x 15 mL) and brine (15 mL). The organic phase was concentrated under reduced pressure and the crude was dissolved in CH_2Cl_2 (10 mL) and treated with dry pyridine (168 µL, 2.08 mmol) and *iso*-valeryl chloride (130 µL, 1.04 mmol) at 25 °C for 16 h. The mixture was diluted with CH_2Cl_2 (20 mL) and washed with 3 N HCl (20 mL). The aqueous phase

was extracted with CH_2Cl_2 (2 x 10 mL) and the combined organic extracts were washed with H_2O (25 mL), brine (25 mL), dried over anhydrous Na_2SO_4 and concentrated under reduced pressure. The crude was purified by automated flash chromatography on silica (Eluent: petroleum ether/EtOAc from 100:0 to 70:30, v/v) affording the title intermediate **22** (110 mg, 0.21 mmol, 31% yield) as whitish solid. ^{1}H-NMR ($CDCl_3$, 400 MHz): δ 0.65 (3H, s, C*H*$_3$), 0.86-0.97 [15H, m, 19-C*H*$_3$ + 21-C*H*$_3$ + 6-CH$_2$C*H*$_3$ + (C*H*$_3$)$_2$CHCH$_2$], 3.60-3.62 (1H, m, 3β-C*H*), 3.65 (3H, s, CO$_2$C*H*$_3$), 3.70 (1H, s, 7β-C*H*), 5.29-5.31 (1H, brs, N*H*).

Benzyl 3α,7α-dimethoxymethyloxy-6α-ethyl-5β-cholan-24-oate (23): To a solution of benzyl 3α,7α-dihydroxy-6α-ethyl-5β-cholan-24-oate (**2**, 3.5 g, 6.85 mmol) in CH_2Cl_2 (100 mL), *N,N*-diisopropylethylamine (14.1 mL, 82.23 mmol) and DMAP (42 mg, 0.34 mmol) were added at 25 °C. The mixture was stirred at 25 °C and methoxymethylchloride (4.2 mL, 54.82 mmol) was added dropwise over 30 min. The resulting dark mixture was refluxed for 16 h. The mixture was allowed to cool to r.t. and washed with H_2O (100 mL), 0.5 N aqueous solution of HCl (100 mL), brine (100 mL), dried over anhydrous Na_2SO_4 and concentrated under reduced pressure. The crude was filtered on a short pad of silica eluting with CH_2Cl_2. The title intermediate **23** (3.4 g, 5.68 mmol, 84% yield) was obtained as colorless oil. ^{1}H-NMR (400 MHz, $CDCl_3$): δ 0.62 (3H, s, 18-C*H*$_3$), 0.90-0.99 (9H, m, 19-C*H*$_3$ + 6-CH$_2$C*H*$_3$ + 21-C*H*$_3$), 3.29-3.33 (1H, brs, 3β-C*H*), 3.37 (6H, s, 2 x OCH$_2$OC*H*$_3$), 3.49 (1H, s, 7β-C*H*), 4.59-4.64 (2H, *pseudo*-q, OC*H*$_2$OCH$_3$), 4.70 (2H, s, OC*H*$_2$OCH$_3$), 5.11 (2H, brs, C*H*$_2$Ph), 7.33-7.42 (5H, m, Ph).

Benzyl 23-(*R*)-methyl 3α,7α-dihydroxy-6α-ethyl-5β-cholan-24-oate (24a) and benzyl 23-(*S*)-methyl 3α,7α-dihydroxy-6α-ethyl-5β-cholan-24-oate (24b): Into a flame-dried three necks round bottom flask, to a solution of freshly distilled *N,N*-diisopropylamine (3.1 mL, 22.38 mmol) in freshly distilled tetrahydrofuran (50 mL), a 2.5 N titrated solution of *n*-butyllithium in hexane

(8.6 mL, 21.37 mmol) was added dropwise, at -78 °C and under argon atmosphere, over 30 min. The mixture was stirred at -78 °C and under argon atmosphere for further 40 min and then a solution of benzyl 3α,7α-dimethoxymethyloxy-6α-ethyl-5β-cholan-24-oate (**23**, 2 g, 3.34 mmol) in freshly distilled tetrahydrofuran (30 mL) was added dropwise over 30 min. The mixture was stirred for further 1.5 h and then iodomethane (3.1 mL, 50.09 mmol) was added dropwise over 15 min at -78 °C and under argon atmosphere. After further 1 h of stirring, the mixture was allowed to warm to r.t. and stirred for further 16 h. The mixture was diluted with EtOAc (100 mL) and washed with H_2O (100 mL). The aqueous phase was extracted with EtOAc (2 x 50 mL) and the combined organic extracts were washed with 0.5 N aqueous solution of HCl (100 mL), aqueous saturated solution of $NaHCO_3$ (100 mL), H_2O (100 mL), brine (100 mL), dried over anhydrous Na_2SO_4 and concentrated under reduced pressure. The crude was dissolved in MeOH/THF (1:1, v/v, 40 mL) and treated with 3 N aqueous solution of HCl (20 mL) at 50 °C for 24 h. The mixture was concentrated under reduced pressure and the residue was dissolved in EtOAc (100 mL) and washed with aqueous saturated solution of $NaHCO_3$ (100 mL), H_2O (100 mL), brine (100 mL), dried over anhydrous Na_2SO_4 and concentrated under reduced pressure. The crude was purified by automated flash chromatography on spherical silica (Eluent: CH_2Cl_2/acetone 90:10, v/v). affording the title intermediates **24a** (220 mg, 0.49 mmol, 13% yield) and **24b** (380 mg, 0.72 mmol, 22% yield) as white solids. The absolute stereochemistry at C23 position was confirmed by comparison of ^{1}H- and ^{13}C-NMR spectra with those of C23-methyl derivatives of cholic acid reported in the literature.[2]

24a: ^{1}H-NMR (400 MHz, $CDCl_3$): δ 0.65 (3H, s, 18-CH_3), 0.80-0.93 (9H, m, 19-CH_3 + 6-CH_2CH_3 + 21-CH_3), 1.11 (3H, d, J= 6.90 Hz, 23α-CH_3), 2.46-2.58 (1H, m, 23β-CH), 3.31-3.45 (1H, brs, 3β-CH), 3.68 (1H, s, 7β-CH), 5.04-5.13 (2H, brs, CH_2Ph), 7.27-7.37 (5H, m, Ph). ^{13}C-NMR (100

MHz, $CDCl_3$): *δ* 11.6, 11.7, 16.2, 18.1, 20.7, 22.2, 23.1, 23.6, 28.5, 30.5, 33.1, 33.7, 33.8, 35.4, 35.5, 36.8, 39.5, 39.6, 39.9, 41.1, 42.8, 45.1, 50.4, 56.5, 66.0, 70.8, 72.2, 128.0, 128.1, 128.4, 136.2, 177.3.

24b: ^{1}H-NMR (400 MHz, $CDCl_3$): *δ* 0.55 (3H, s, 18-C*H₃*), 0.91-0.93 (9H, m, 19-CH_3 + 6-CH_2CH_3 + 21-CH_3), 1.16 (3H, d, *J*= 6.70 Hz, 23β-CH_3), 2.59-2.69 (1H, m, 23α-C*H*), 3.32-3.46 (1H, brs, 3β-C*H*), 3.70 (1H, s, 7β-C*H*), 5.04-5.13 (2H, brs, CH_2Ph), 7.27-7.36 (5H, m, Ph). ^{13}C-NMR (100 MHz, $CDCl_3$): *δ* 11.65, 11.69, 18.5, 18.9, 20.7, 22.2, 23.1, 23.7, 28.2, 30.6, 33.2, 34.0, 34.6, 35.48, 35.51, 37.3, 39.6, 40.0, 41.0, 41.1, 42.7, 45.2, 50.5, 56.6, 65.9, 70.9, 72.3, 128.0, 128.3, 128.5, 136.2, 176.8.

Benzyl 23-(*R*)-methyl 3α-[(((4'-nitrophenoxy)carbonyl)oxy)]-7α-dihydroxy-6α-ethyl-5β-cholan-24-oate (25a) and benzyl 23-(*S*)-methyl 3α-[(((4'-nitrophenoxy)carbonyl)oxy)]-7α-dihydroxy-6α-ethyl-5β-cholan-24-oate (25b): Synthesized according to the procedure reported for compound **19**. After purification by automated flash chromatography on silica gel (Eluent: petroleum ether/Et_2O from 100:0 to 70:30, v/v), the title intermediates **25a** and **25b** were obtained as white solids in 83% and 81% yield, respectively.

25a: ^{1}H-NMR (400 MHz, $CDCl_3$): *δ* 0.67 (3H, s, 18-CH_3), 0.80-0.93 (9H, m, 19-CH_3 + 6-CH_2CH_3 + 21-CH_3), 1.13 (3H, d, *J*= 6.90 Hz, 23α-CH_3), 2.51-2.54 (1H, m, 23β-C*H*), 3.72 (1H, s, 7β-C*H*), 4.45-4.60 (1H, brs, 3β-C*H*), 5.11 (2H, brs, CH_2Ph), 7.27-7.38 (7H, m, Ph), 8.27 (2H, d, *J*= 8.0 Hz, Ph).

25b: ^{1}H-NMR (400 MHz, $CDCl_3$): *δ* 0.55 (3H, s, 18-CH_3), 0.89-0.92 (9H, m, 19-CH_3 + 6-CH_2CH_3 + 21-CH_3), 1.16 (3H, d, *J*= 6.70 Hz, 23β-CH_3), 2.56-2.71 (1H, m, 23α-C*H*), 3.72 (1H, s, 7β-C*H*), 4.47-4.61 (1H, brs, 3β-C*H*), 5.12 (2H, brs, CH_2Ph), 7.27-7.36 (5H, m, Ph), 8.26 (2H, d, *J*= 8.0 Hz, Ph).

24,24-Diphenyl 3α,7α-dihydroxy-6α-ethyl-5β-cholan-23-ene (27): Synthesized from OCA (**1**) in 80% isolated yield over three steps according to the literature.[3] Analytical and spectroscopical data were in agreement with the literature.

24,24-Diphenyl 3α,7α-diacetoxy-6α-ethyl-5β-cholan-23-ene (28): To a solution of 24,24-diphenyl 3α,7α-dihydroxy-6α-ethyl-5β-cholan-23-ene (**27**, 2.64 g, 4.89 mmol) in CH_2Cl_2 (25 mL), acetic anhydride (4.7 mL, 48.9 mmol) and $Bi(OTf)_3$ (160 mg, 0.25 mmol) were added and the resulting mixture was stirred at 25 °C for 2 h. The reaction mixture was slowly quenched with aqueous saturated solution of $NaHCO_3$ (100 mL) and extracted with CH_2Cl_2 (2 x 20 mL). The combined organic extracts were washed with H_2O (100 mL), brine (100 mL), dried over anhydrous Na_2SO_4 and concentrated under reduced pressure. The crude **28** (white solid, quantitative recovery) was used for the next step without further purification. ^{1}H-NMR ($CDCl_3$, 400 MHz): *δ* 0.65 (3H, s, 18-CH_3), 0.90 (3H, t, *J*= 7.34 Hz, 6-CH_2CH_3), 0.95 (3H, s, 19-CH_3), 0.97 (3H, d, *J*= 6.56 Hz, 21-CH_3), 2.05 (3H, s, 3α-OCOCH_3), 2.07 (3H, s, 7α-OCOCH_3), 2.23-2.32 (1H, brm, 22-C$H_{2(a)}$), 4.55-4.61 (1H, m, 3β-C*H*), 5.10 (1H, s, 7β-C*H*), 6.09-6.13 (1H, m, 23-C*H*), 7.11-7.39 (brm, 10H, 2 x C_6H_5).

3α,7α-Dihydroxy-6α-ethyl-5β-cholan-24-*nor*-23-oic acid (26): $NaIO_4$ (9.41 g, 44.0 mmol) was suspended in H_2O (9 mL) and 2 N aqueous solution of H_2SO_4 (1.3 mL) and the resulting suspension was stirred at room temperature for 30 min. The suspension was then cooled to 0 °C and $RuCl_3$*H_2O (51 mg, 0.25 mmol) was added in one portion. The resulting mixture was stirred for 1 h and a bright yellow colour appears. MeCN (13 mL) was added and after stirring for further 5 min, a solution of 24,24-diphenyl 3α,7α-diacetoxy-6α-ethyl-5β-cholan-23-ene (**28**, 3.06 g, 4.89 mmol) in EtOAc (20 mL) was added dropwise at 0 °C, over 10 min. After further 10 min, the mixture was allowed to warm to r.t. and it was filtered off washing with EtOAc. The filtrate was

washed with H_2O (50 mL), brine (50 mL), dried over anhydrous Na_2SO_4 and concentrated under reduced pressure. The crude was dissolved in MeOH/H_2O (20 mL, 9:1, v/v) and refluxed in the presence of NaOH (2 g, 50 mmol) for 48 h. The mixture was allowed to cool to r.t., acidified by adding 3 N aqueous solution of HCl (200 mL) and extracted with CH_2Cl_2 (3 x 100 mL). The combined organic extracts were washed with H_2O (50 mL), brine (50 mL), dried over anhydrous Na_2SO_4 and concentrated under reduced pressure. The crude was purified by automated flash chromatography on silica (Eluent: CH_2Cl_2/MeOH from 100:0 to 90:10, v/v + 0.05% AcOH) affording the title intermediate **26** (1.63 g, 4.0 mmol, 82% yield from **27**) as white solid. ^{1}H-NMR (CD_3OD, 400 MHz): δ 0.75 (3H, s, 18-CH_3), 0.89-0.92 (6H, m, 19-CH_3 + 6-CH_2CH_3), 1.02 (3H, d, J= 6.07 Hz, 21-CH_3), 2.39-2.44 (1H, brm, 22-C$H_{2(a)}$), 3.31-3.35 (1H, m, 3β-CH), 3.66 (1H, s, 7β-CH). ^{13}C-NMR (CD_3OD, 100 MHz): δ 12.0, 12.2, 20.0, 22.0, 23.5, 23.8, 24.5, 29.4, 31.3, 34.4, 34.5, 35.1, 36.6, 36.8, 40.9, 41.6, 42.6, 43.1, 43.8, 47.0, 51.7, 57.4, 71.1, 73.2, 177.6.

Benzyl 3α-[(((4'-nitrophenoxy)carbonyl)oxy)]-7α-dihydroxy-6α-ethyl-5β-cholan-24-*nor*-23-oate (29): Synthesized from intermediate **26** according to the two-step procedure reported for compound **19**. After purification by automated flash chromatography on silica gel (Eluent: petroleum ether/Et_2O from 100:0 to 60:40, v/v), the title intermediate **29** was obtained as white solid in 77% yield. ^{1}H-NMR ($CDCl_3$, 400 MHz): δ0.68 (3H, s, CH_3), 0.87-0.92 (6H, m, 19-CH_3 + 6-CH_2CH_3), 0.98 (3H, d, J= 6.08 Hz, 21-CH_3), 2.49 (1H, dd, J_1= 4.0 Hz, J_2= 16.0 Hz, 22-C$H_{2(a)}$), 3.74 (1H, s, 7β-CH), 4.46-4.61 (1H, m, 3β-CH), 5.10-5.17 (2H, m, CO_2CH_2Ph), 7.29-7.35 (7H, m, Ph), 8.13 (2H, d, J= 8.07 Hz, Ph).

3α,7α-Dihydroxy-6α-ethyl-24-*nor*-5β-cholan-23-sulfate sodium salt (INT-767, 30): Synthesized from OCA (**1**) in 41% yield over 7 steps according to the literature.[3] Analytical and spectroscopical data were in agreement with the literature.

3α-[(((4'-Nitrophenoxy)carbonyl)oxy)]-7α-hydroxy-6α-ethyl-24-*nor*-5β-cholan-23-sulfate (31): Synthesized from INT-767 (**30**) according to the procedure reported for **19**. After purification by automated flash chromatography on silica gel (Eluent: CH_2Cl_2/MeOH from 100:0 to 80:20, v/v + 0.05% AcOH), the title intermediate **31** was obtained as white solid in 43% yield. ^{1}H-NMR (CD_3OD, 400 MHz): δ 0.71 (3H, s, CH_3), 0.89-0.95 (6H, m, 19-CH_3 + 6-CH_2CH_3), 1.00 (3H, d, *J*= 5.98 Hz, 21-CH_3), 3.67 (1H, s, 7β-C*H*), 4.01-4.12 (2H, m, 23-C$H_2$$SO_3$H), 4.46-4.55 (1H, m, 3β-C*H*), 7.44 (2H, d, *J*= 8.30 Hz, Ph), 8.30 (2H, d, *J*= 8.30 Hz, Ph).

3α,7α-Dihydroxy-6α-ethyl-5β-cholan-22,23-*bisnor*-cholan-(1',2',4'-oxadiazol-5-one) (32): Synthesized from OCA (**1**) in 27% yield over 7 steps according to the literature.[4] Analytical and spectroscopical data were in agreement with the literature.

3-[(((4'-Nitrophenoxy)carbonyl)oxy)]-7α-hydroxy-6α-ethyl-5β-cholan-22,23-*bisnor*-cholan-(1',2',4'-oxadiazol-5-one) (33): Synthesized from OCA (**1**) according to the procedure reported for **19**. After purification by automated flash chromatography on silica gel (Eluent: petroleum ether/EtOAc from 100:0 to 65:35, v/v), the title intermediate **33** was obtained as white solid in 74% yield. ^{1}H-NMR ($CDCl_3$, 400 MHz): δ 0.71 (3H, s, CH_3), 0.84-0.92 (6H, m, 19-CH_3 + 6-CH_2CH_3), 1.02 (3H, d, *J*= 6.23 Hz, 21-CH_3), 2.27-2.34 (1H, m, 22-C$H_{2(a)}$), 2.66-2.70 (1H, m, 22-C$H_{2(b)}$), 3.73 (1H, s, 7β-C*H*), 4.01-4.12 (2H, m, 23-C$H_2$$SO_3$H), 4.46-4.54 (1H, m, 3β-C*H*), 7.37 (2H, d, *J*= 9.02 Hz, Ph), 8.26 (2H, d, *J*= 8.90 Hz, Ph), 11.2-11.6 (1H, brs, N*H*).

2. NMR spectra

^{1}H-NMR (400 MHz, $CDCl_3$) of benzyl 3α,7α-dihydroxy-6α-ethyl-5β-cholan-24-oate (18)

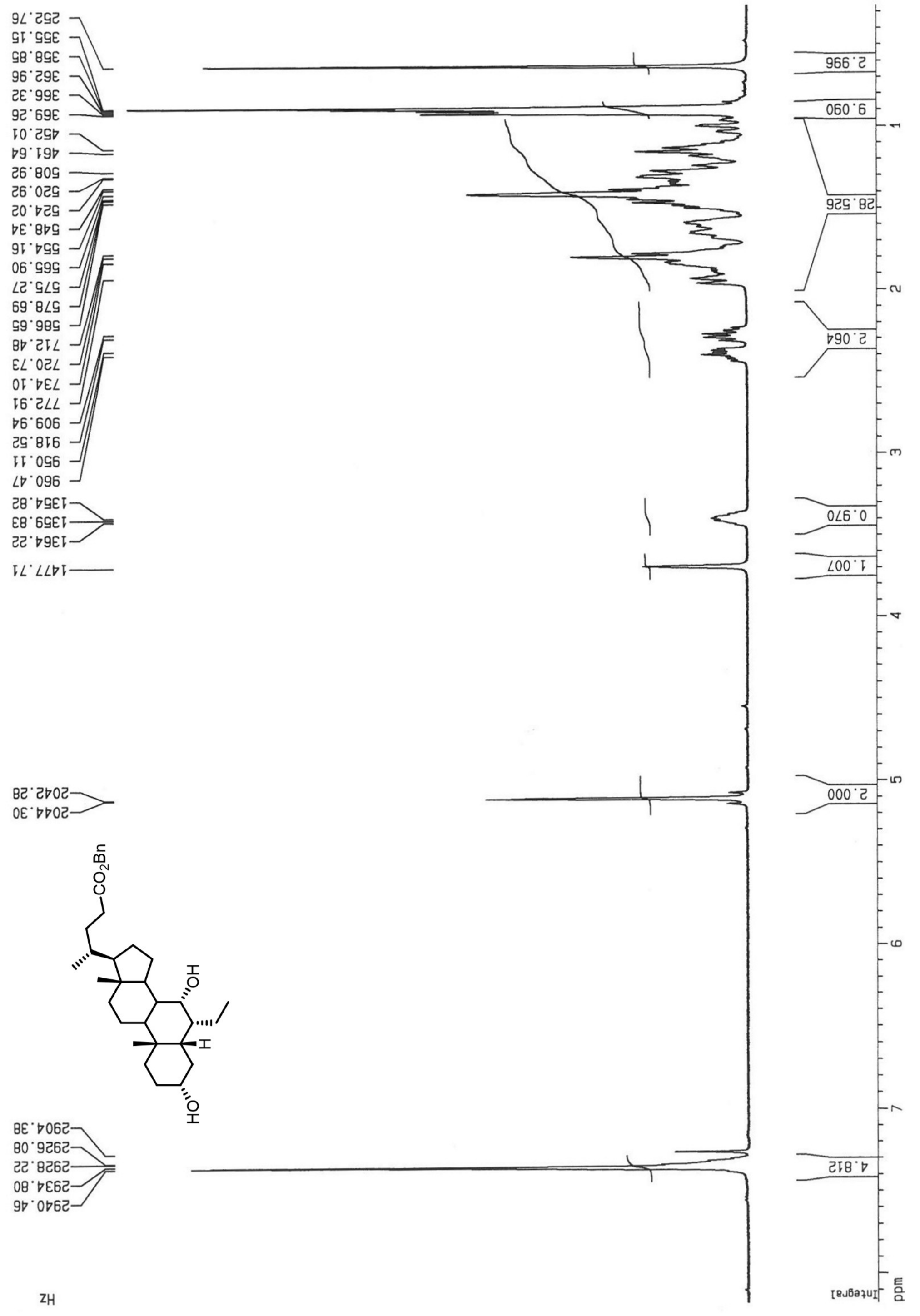

^{1}H-NMR (400 MHz, CDCl$_3$) of benzyl 3α-[(((4'-nitrophenoxy)carbonyl)oxy)]-7α-hydroxy-6α-ethyl-5β-cholan-24-oate (19)

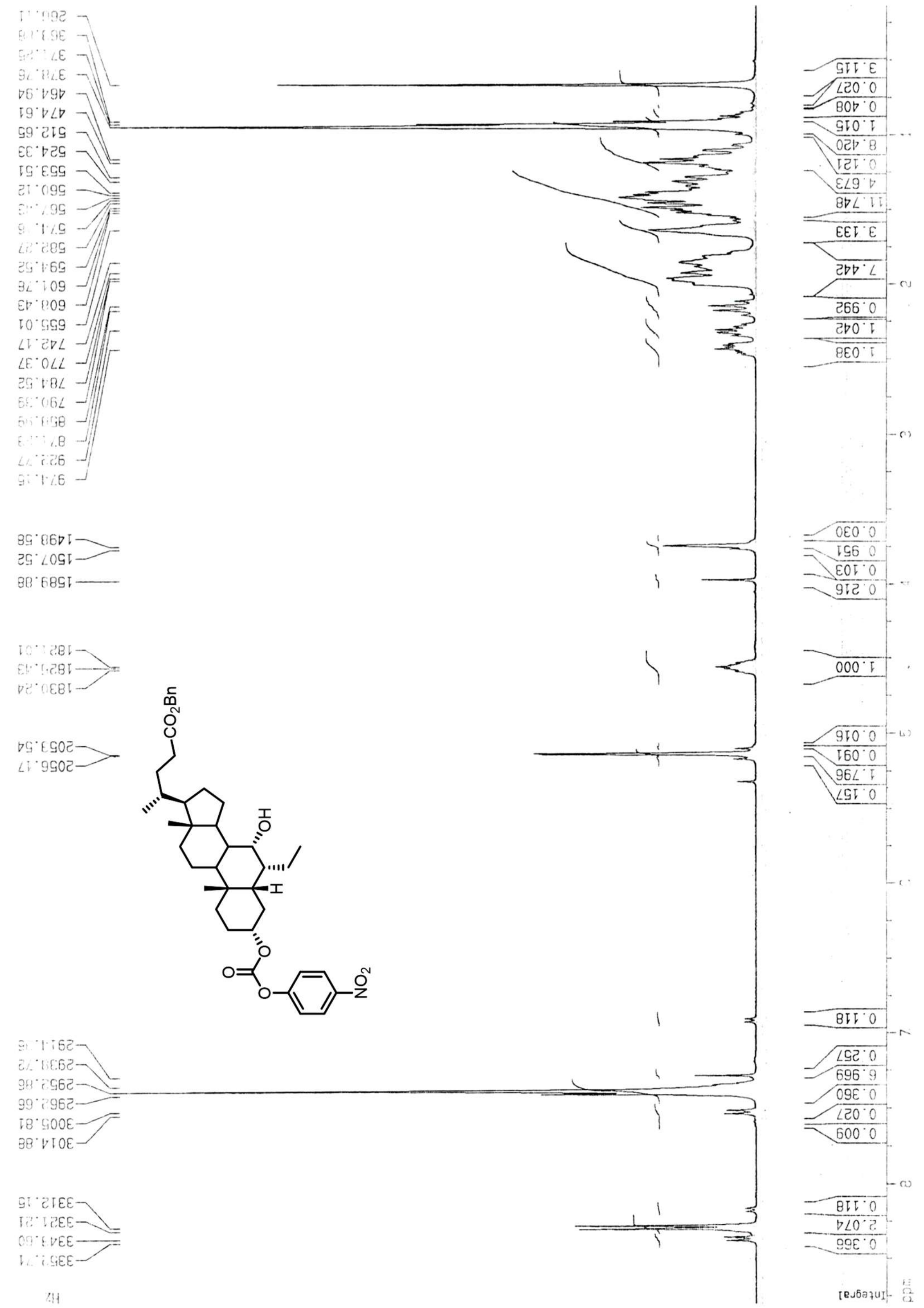

^{1}H-NMR (400 MHz, CD₃OD) of 3α-[((((*S*)-1’-carboxy-2’-methylpropyl)carbamoyl)oxy)]-7α-hydroxy-6α-ethyl-5β-cholan-24-oic acid (2)

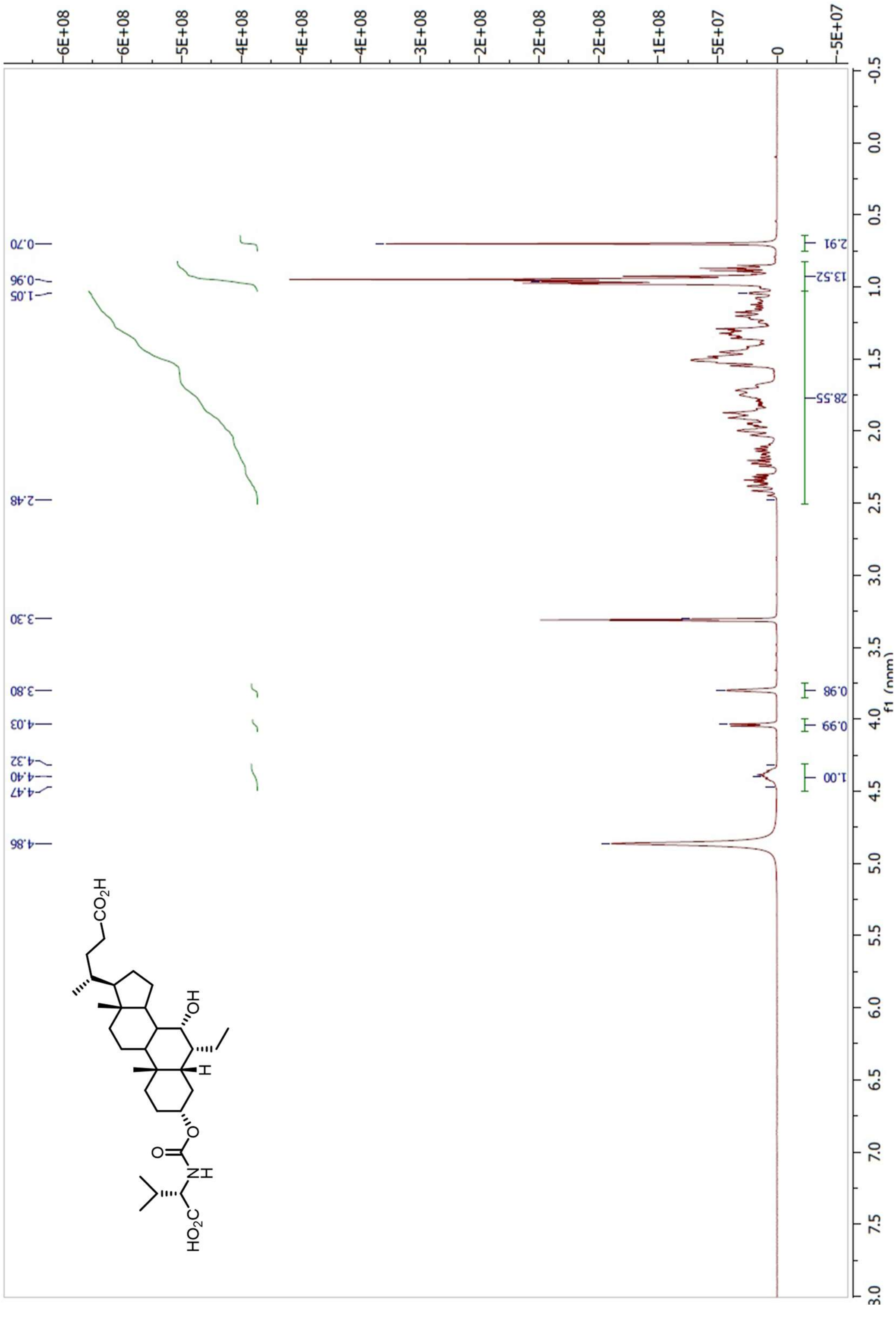

^{13}C-NMR (100 MHz, CD₃OD) of 3α-[((((*S*)-1’-carboxy-2’-methylpropyl)carbamoyl)oxy)]-7α-hydroxy-6α-ethyl-5β-cholan-24-oic acid (2)

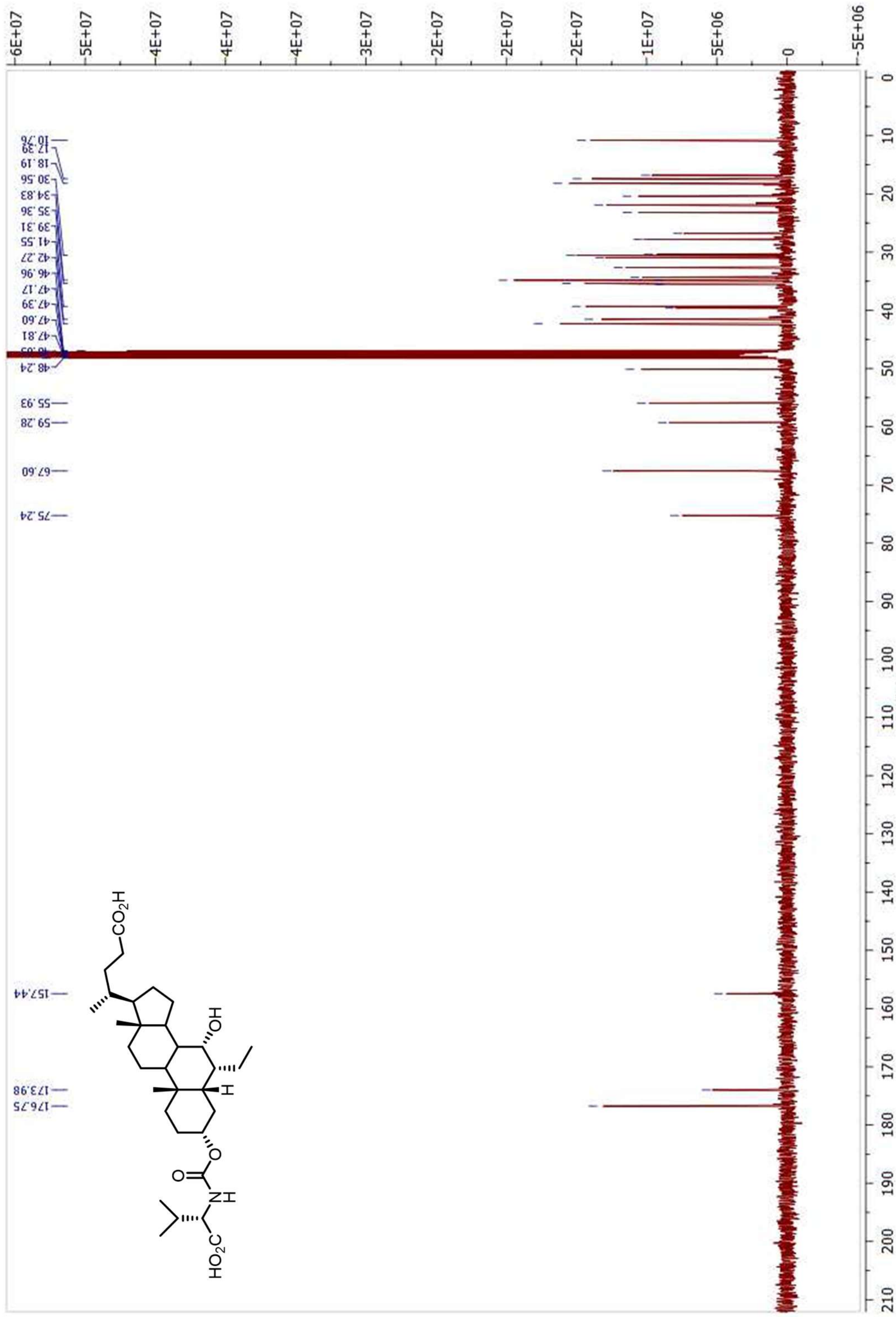

^{1}H-NMR (400 MHz, CD3OD) of 3α-[((((*R*)-1'-carboxy-2'-methylpropyl)carbamoyl)oxy)]-7α-hydroxy-6α-ethyl-5β-cholan-24-oic acid (3)

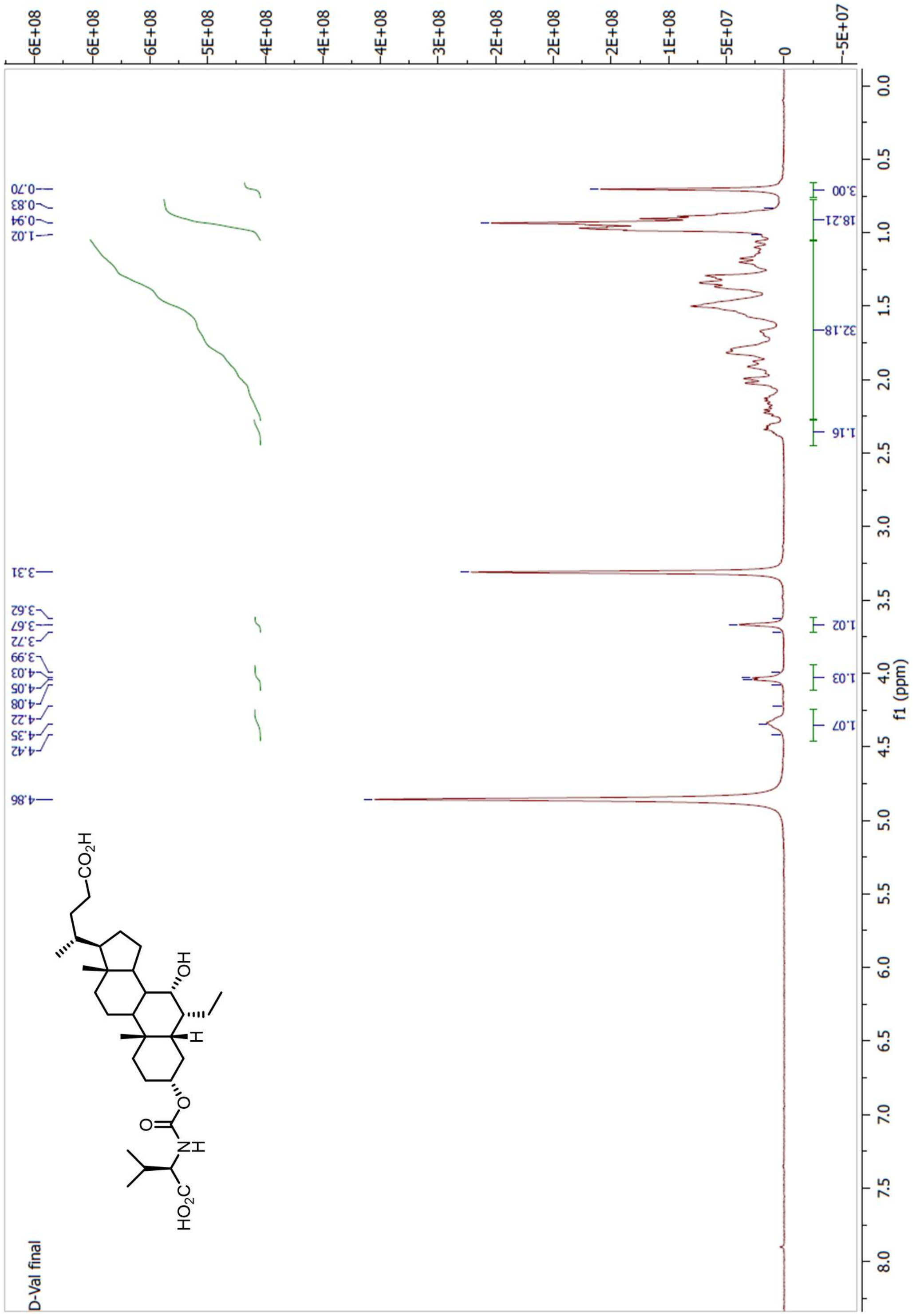

^{13}C-NMR (100 MHz, CD_3OD) of 3α-[((((*R*)-1'-carboxy-2'-methylpropyl)carbamoyl)oxy)]-7α-hydroxy-6α-ethyl-5β-cholan-24-oic acid (3)

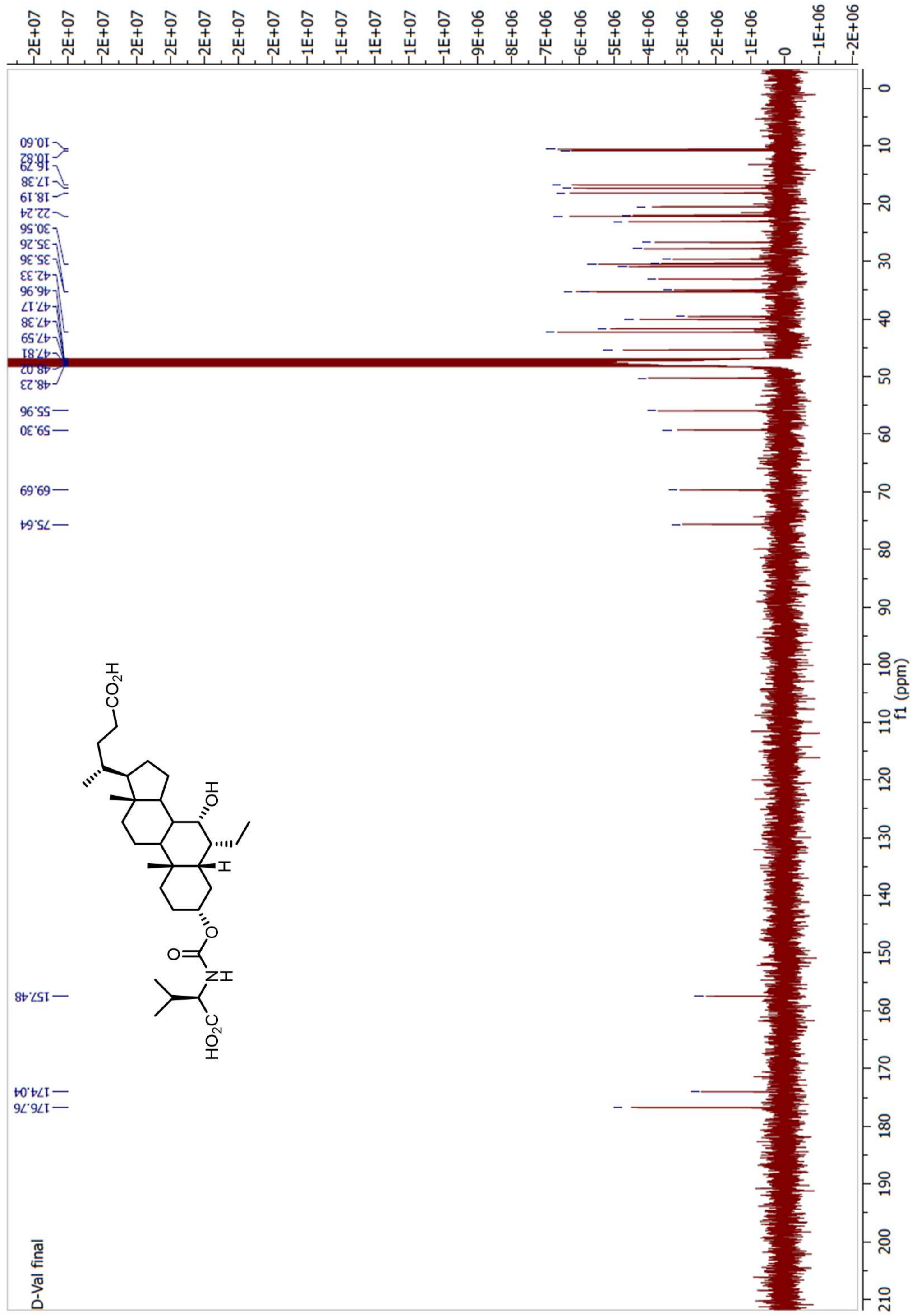

^{1}H-NMR (400 MHz, CDCl$_3$) of 3α-[((((*S*)-1'-carboxy-3'-methylbutyl)carbamoyl)oxy)]-7α-hydroxy-6α-ethyl-5β-cholan-24-oic acid (4)

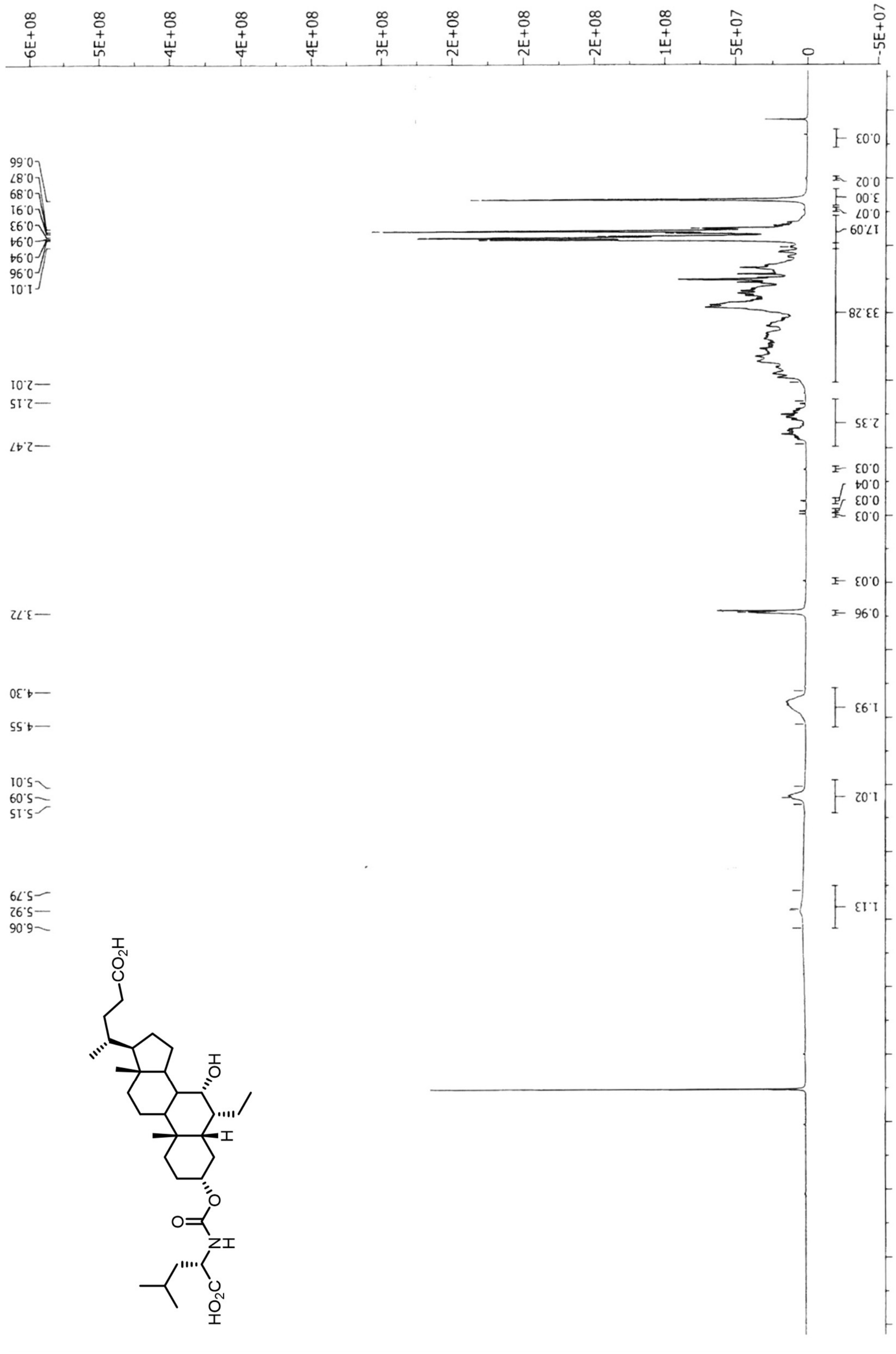

^{13}C-NMR (100 MHz, $CDCl_3$) of 3α-[((((*S*)-1'-carboxy-3'-methylbutyl)carbamoyl)oxy)]-7α-hydroxy-6α-ethyl-5β-cholan-24-oic acid (4)

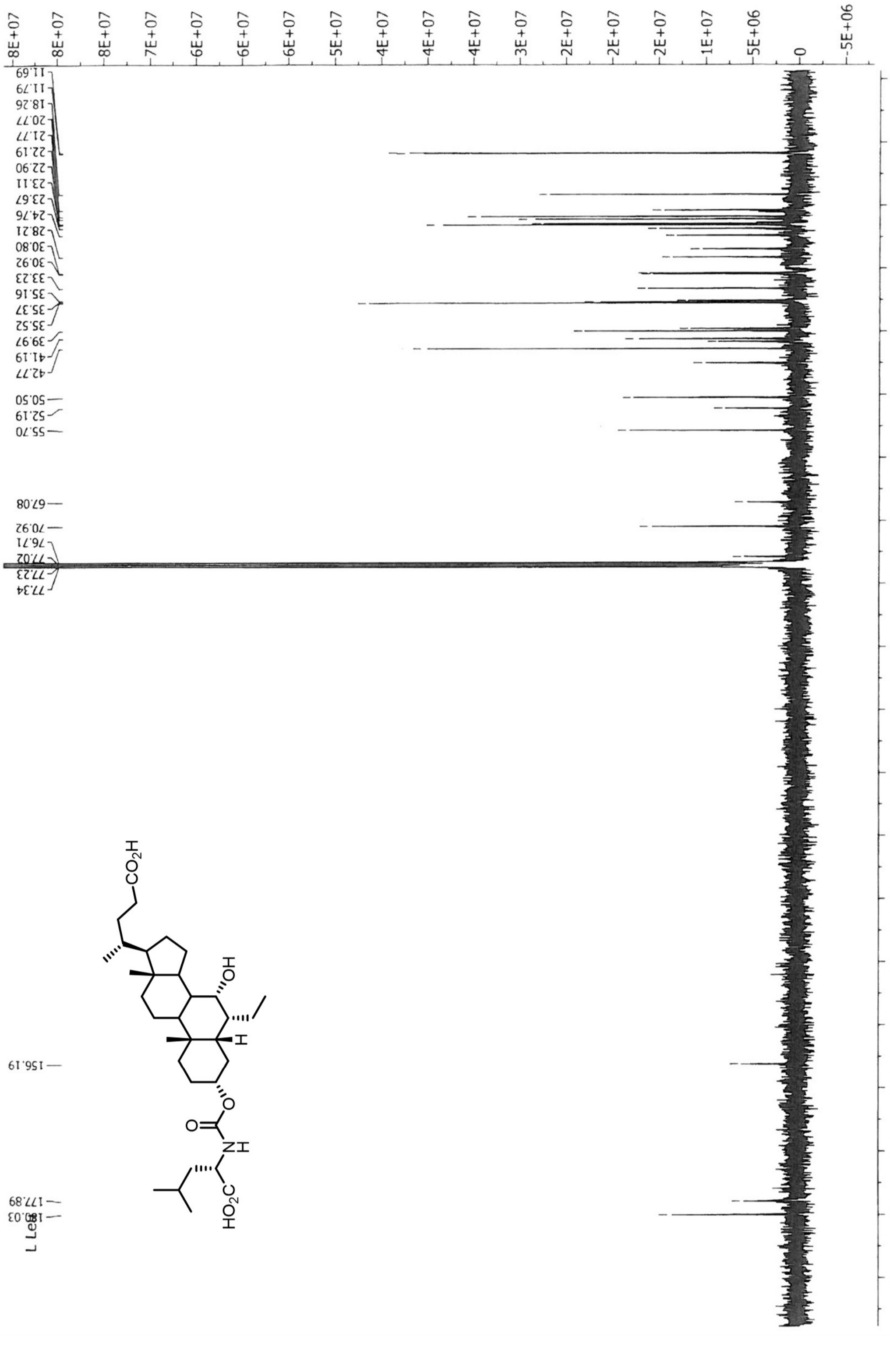

^{1}H-NMR (400 MHz, CD_3OD) of 3α-[(((*S*)-1'-carboxyethyl)carbamoyl)oxy)]-7α-hydroxy-6α-ethyl-5β-cholan-24-oic acid (5)

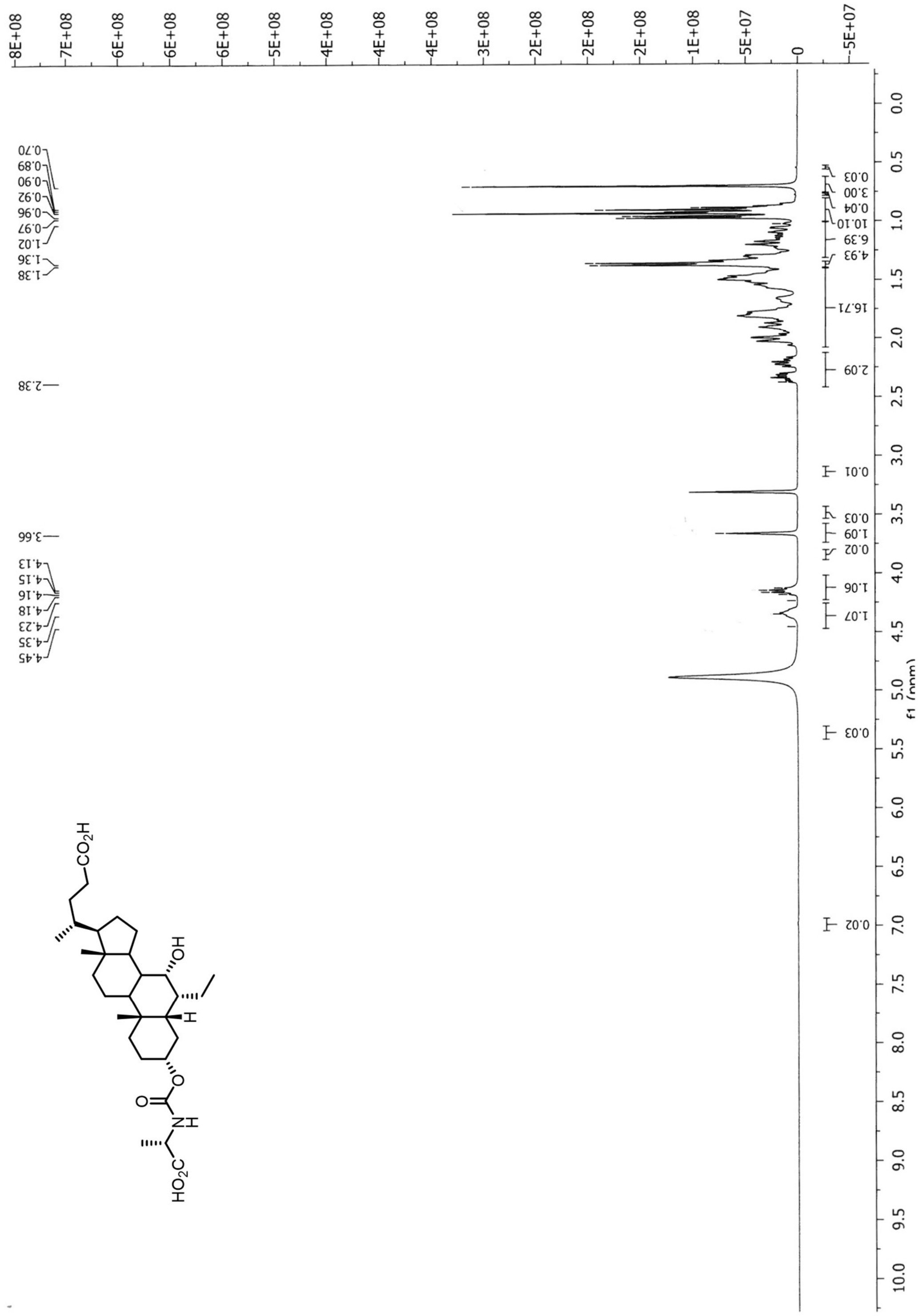

^{13}C-NMR (100 MHz, CD3OD) of 3α-[(((*S*)-1'-carboxyethyl)carbamoyl)oxy)]-7α-hydroxy-6α-ethyl-5β-cholan-24-oic acid (5)

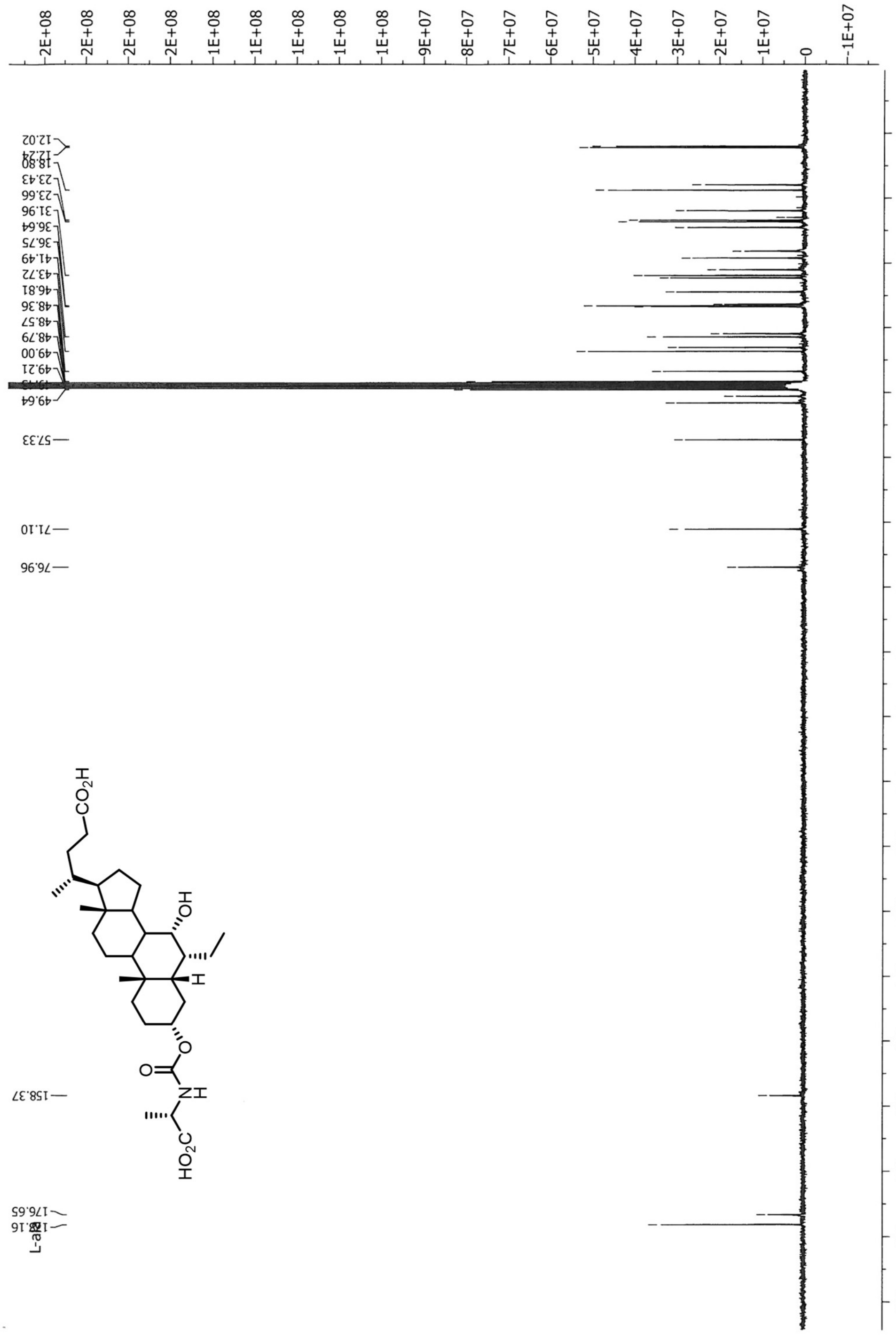

^{1}H-NMR (400 MHz, CD_3OD) of 3α-[(((*S*)-1'-carboxy-2'-phenyl-ethyl)carbamoyl)oxy)]-7α-hydroxy-6α-ethyl-5β-cholan-24-oic acid (6)

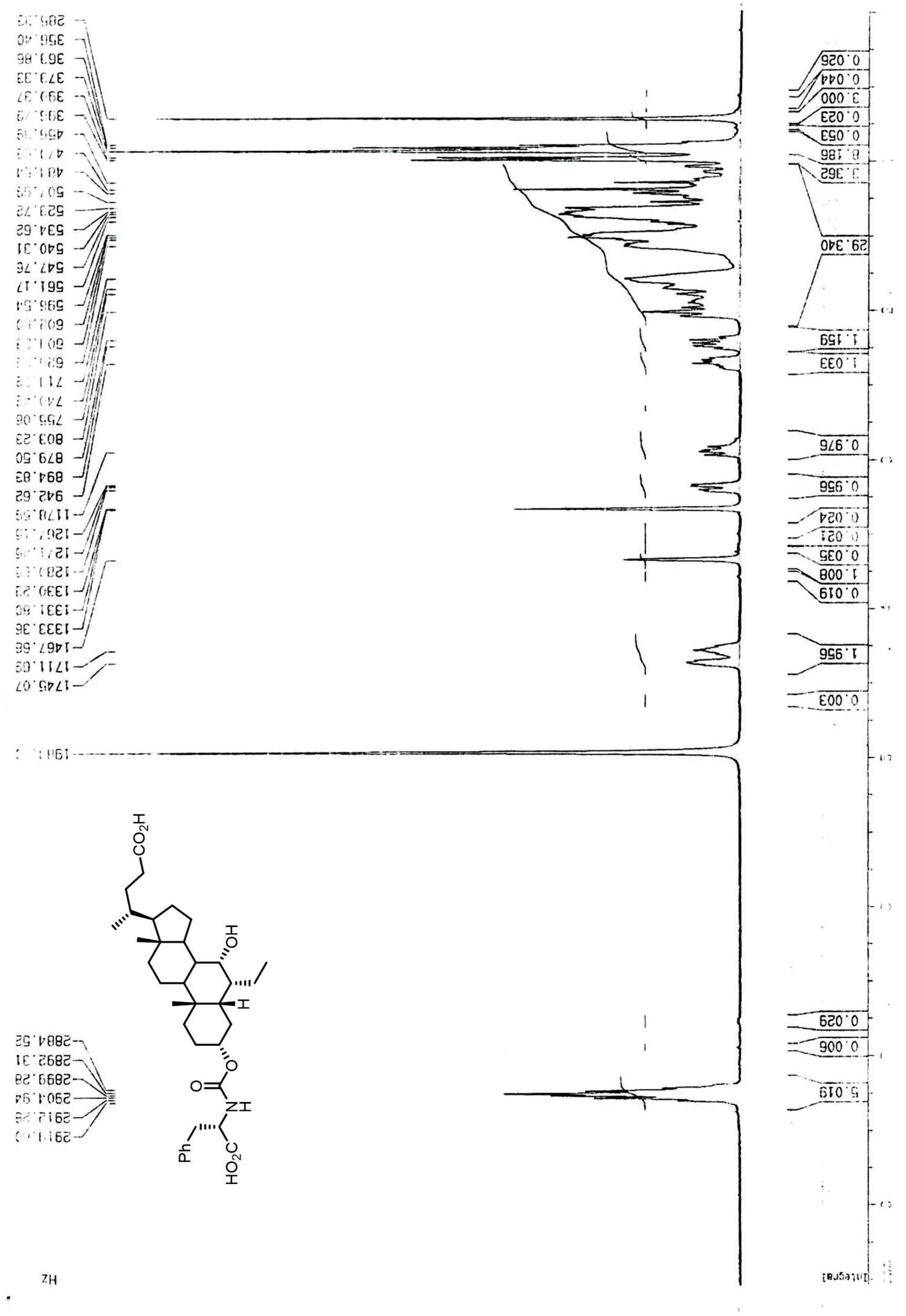

^{13}C-NMR (100 MHz, CD_3OD) of 3α-[(((*S*)-1'-carboxy-2'-phenyl-ethyl)carbamoyl)oxy)]-7α-hydroxy-6α-ethyl-5β-cholan-24-oic acid (6)

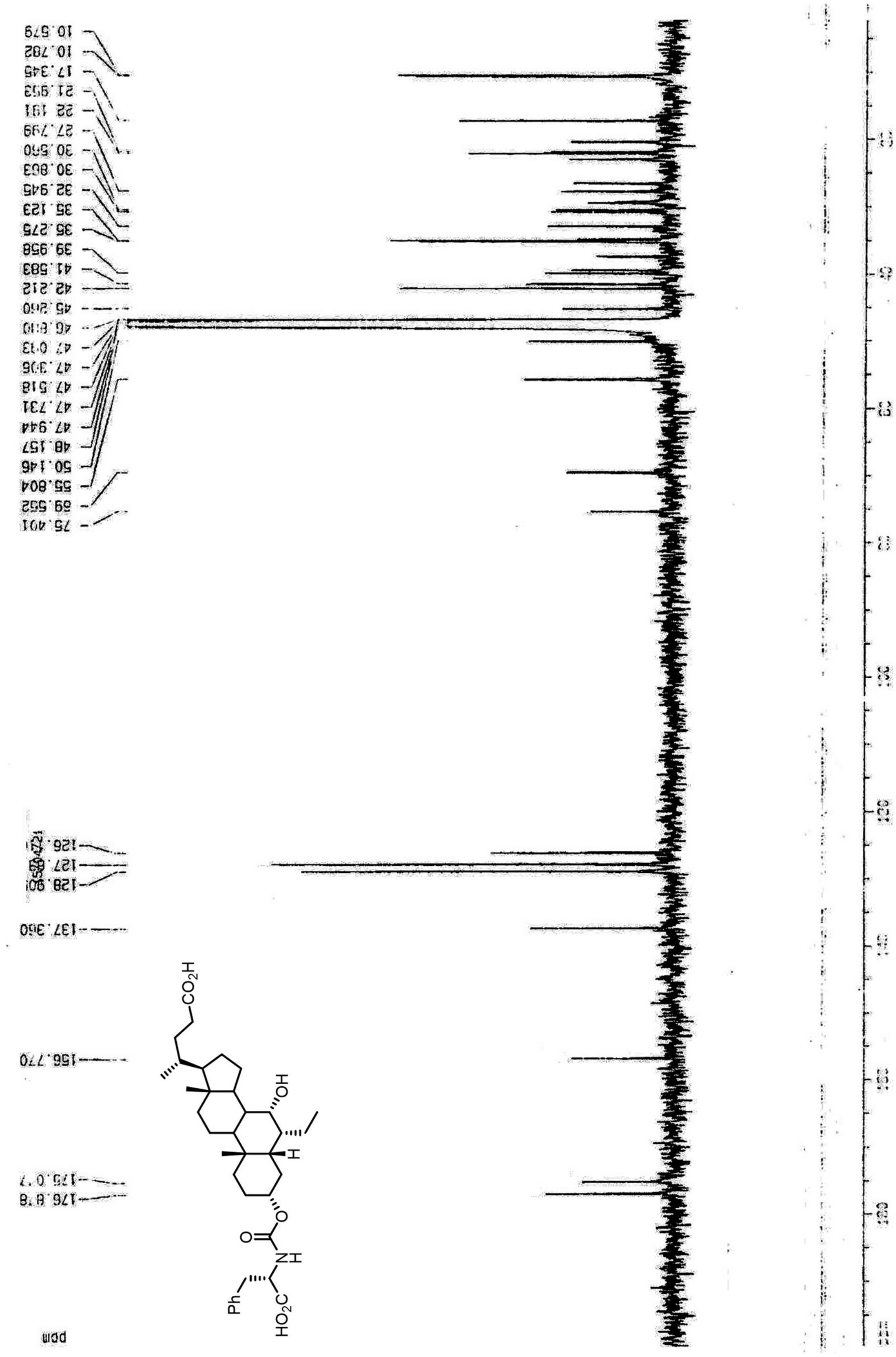

^{1}H-NMR (400 MHz, CD_3OD) of 3α-[((((*S*)-1'-carboxy-3'-methyl-butyl)carbamoyl)oxy)]-7α-hydroxy-6α-ethyl-5β-cholan-24-oic acid (7)

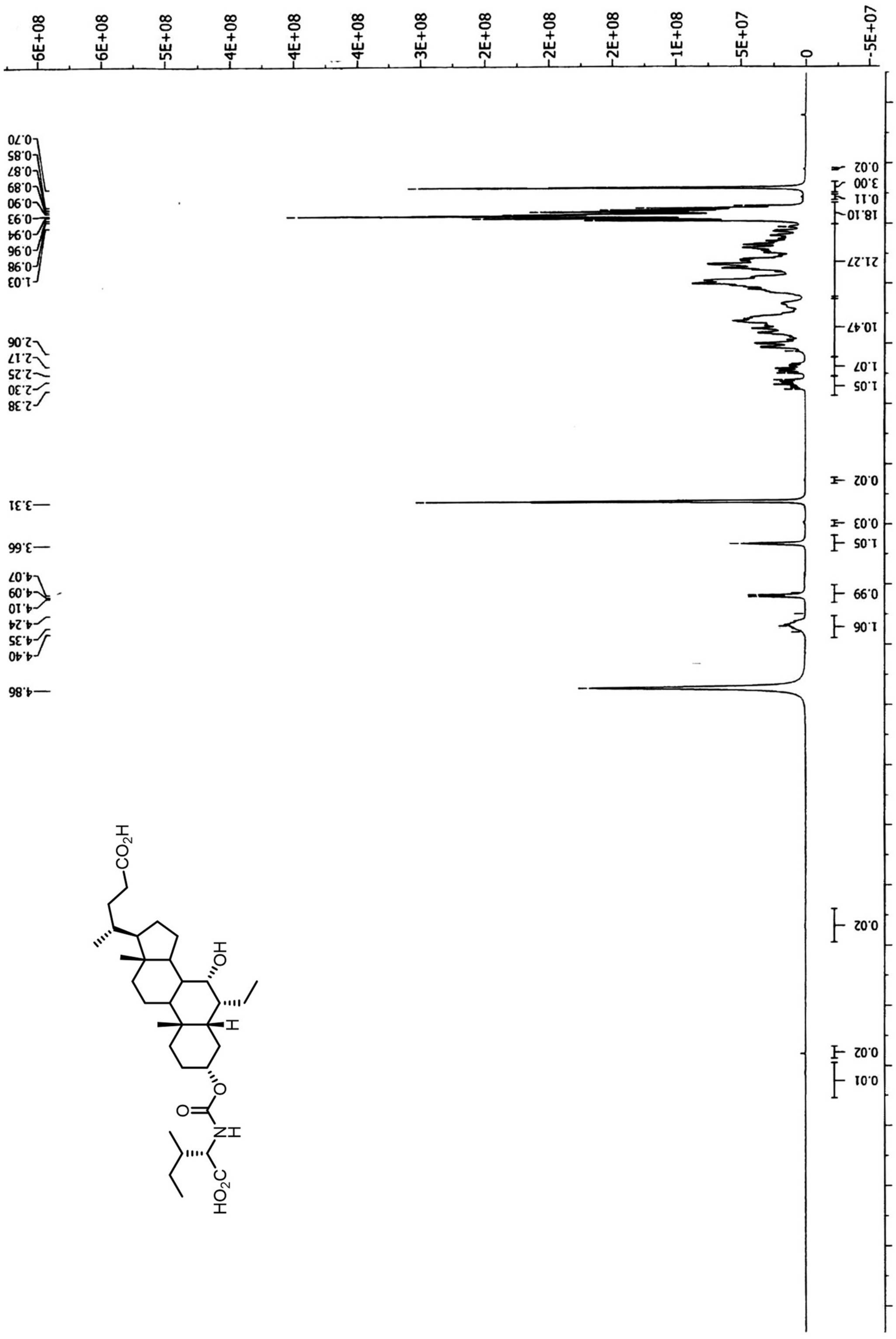

^{13}C-NMR (100 MHz, CD3OD) of 3α-[((((*S*)-1'-carboxy-3'-methyl-butyl)carbamoyl)oxy)]-7α-hydroxy-6α-ethyl-5β-cholan-24-oic acid (7)

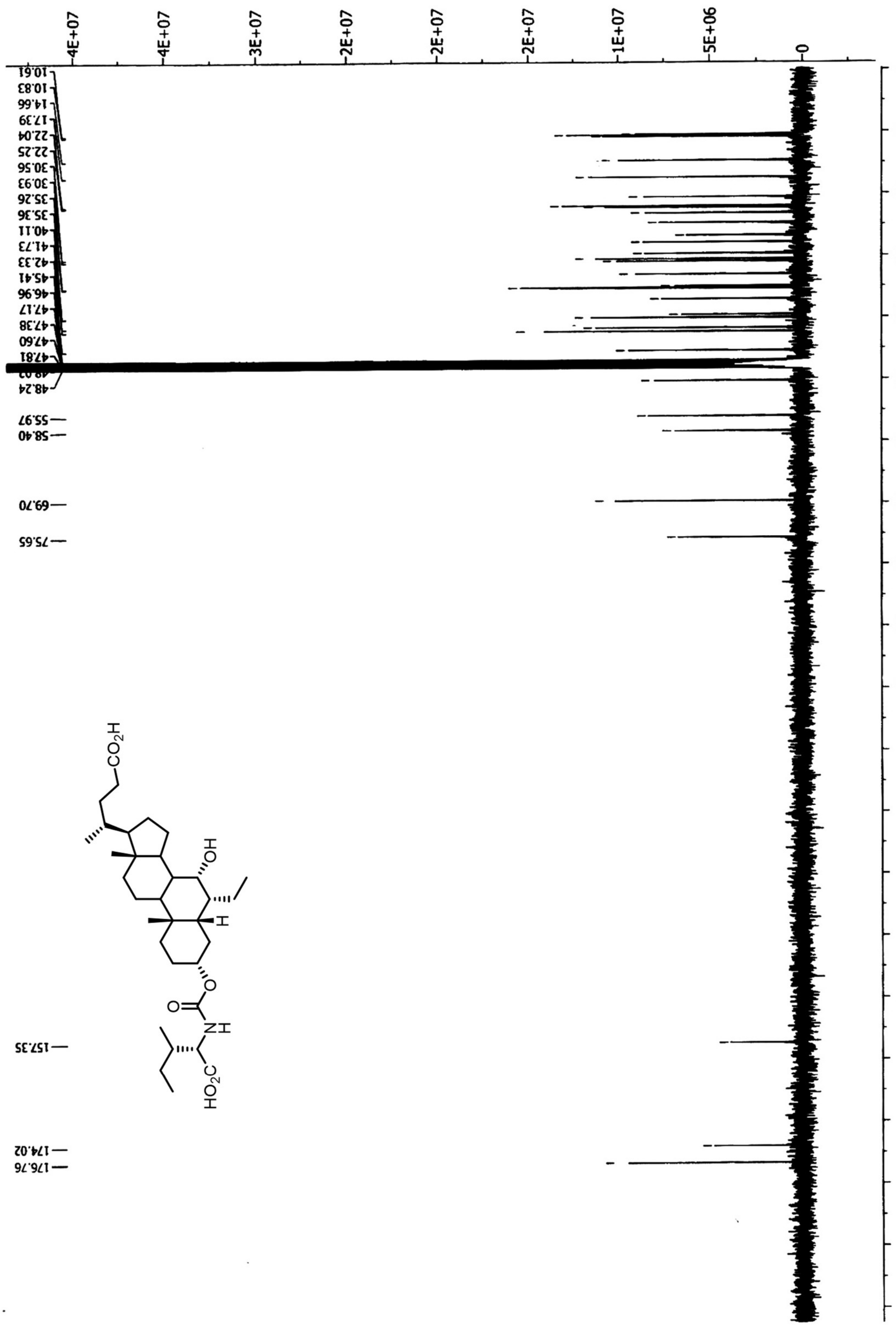

^{1}H-NMR (400 MHz, CD_3OD) of 3α-[(((1'-carboxymethyl)carbamoyl)oxy)]-7α-hydroxy-6α-ethyl-5β-cholan-24-oic acid (8)

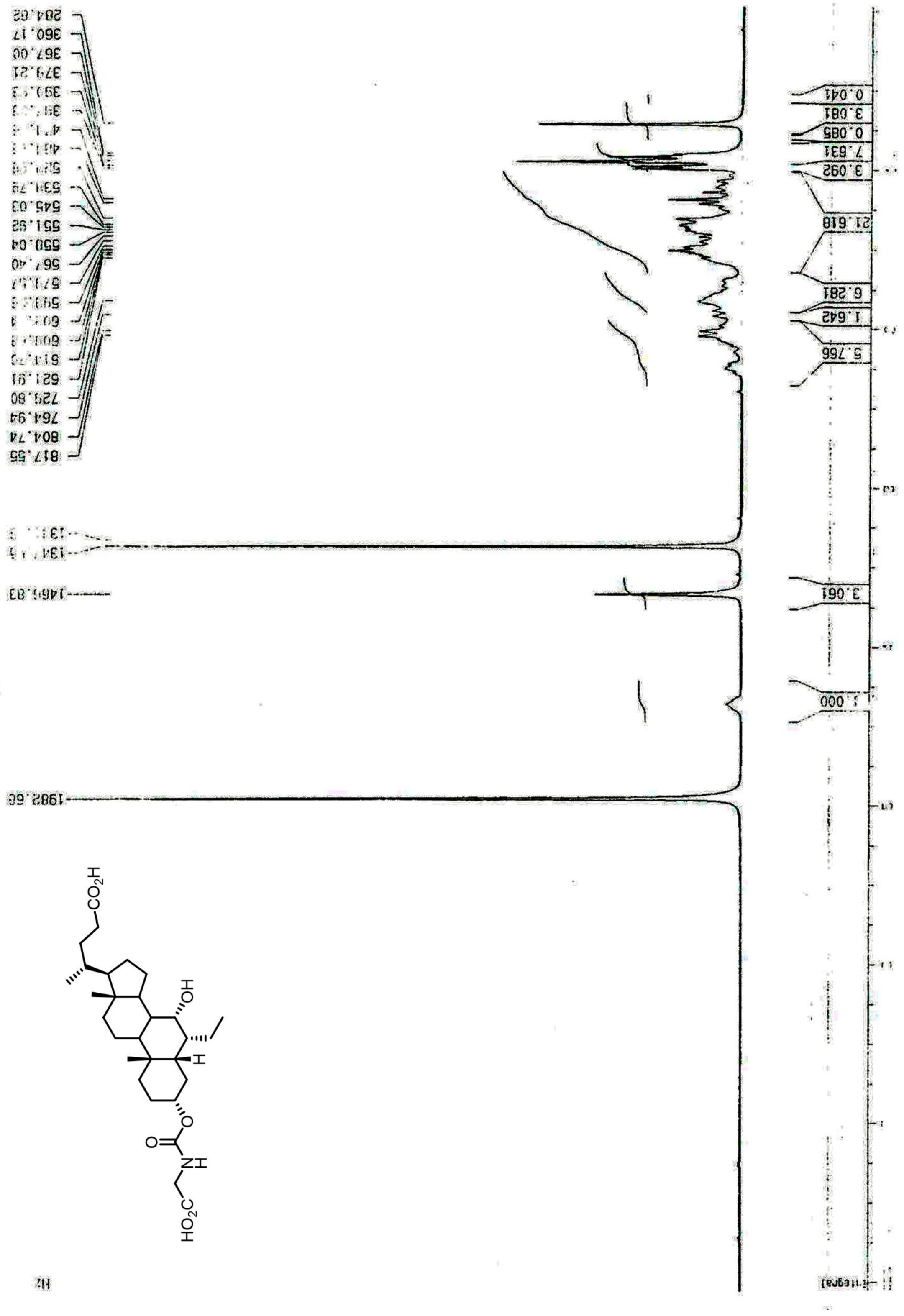

^{13}C-NMR (100 MHz, CD$_3$OD) of 3α-[(((1'-carboxymethyl)carbamoyl)oxy)]-7α-hydroxy-6α-ethyl-5β-cholan-24-oic acid (8)

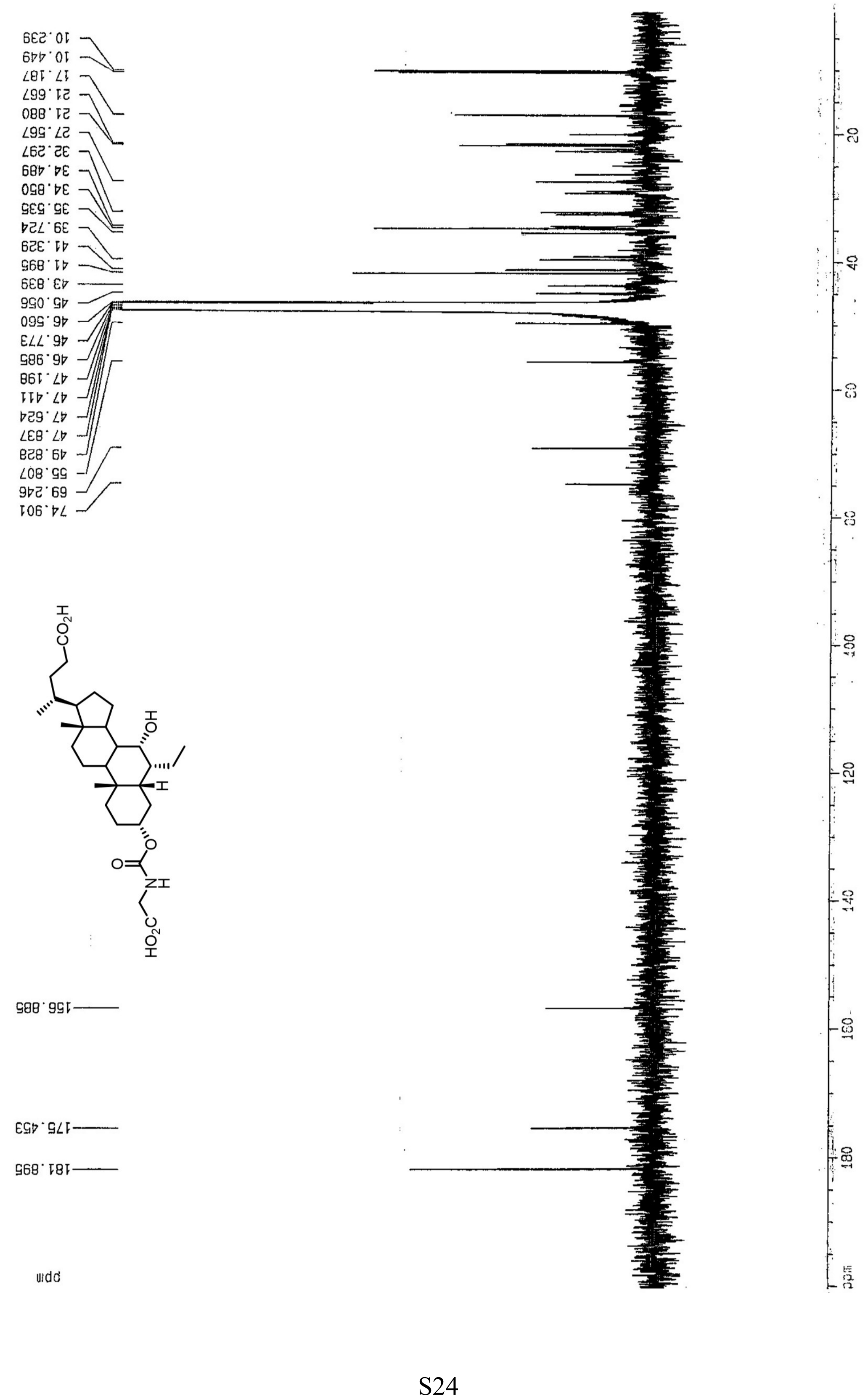

^{1}H-NMR (400 MHz, CD_3OD) of 3α-[(((*iso*-butyl)carbamoyl)oxy)]-7α-hydroxy-6α-ethyl-5β-cholan-24-oic acid (9)

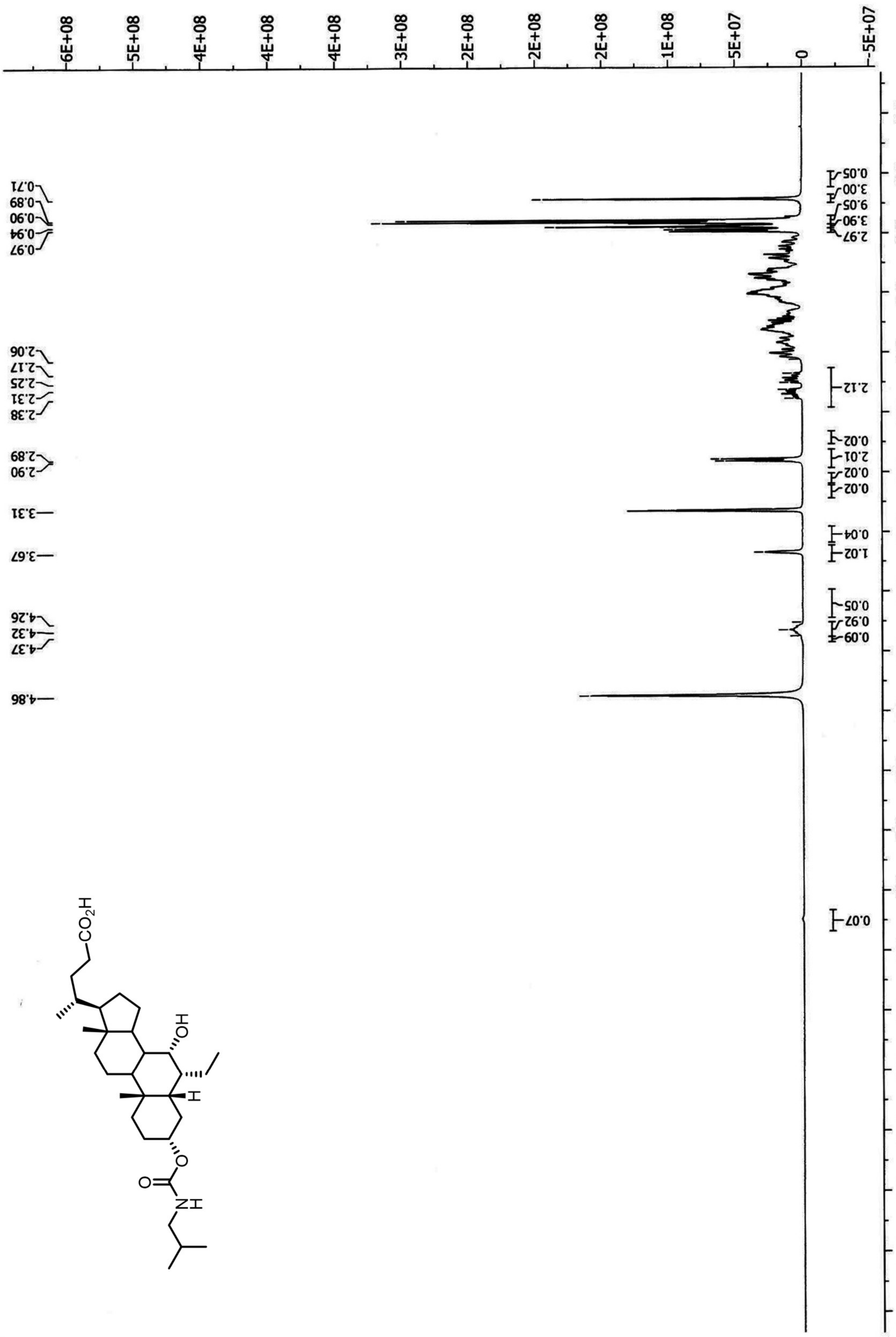

^{13}C-NMR (100 MHz, CD_3OD) of 3α-[(((*iso*-butyl)carbamoyl)oxy)]-7α-hydroxy-6α-ethyl-5β-cholan-24-oic acid (9)

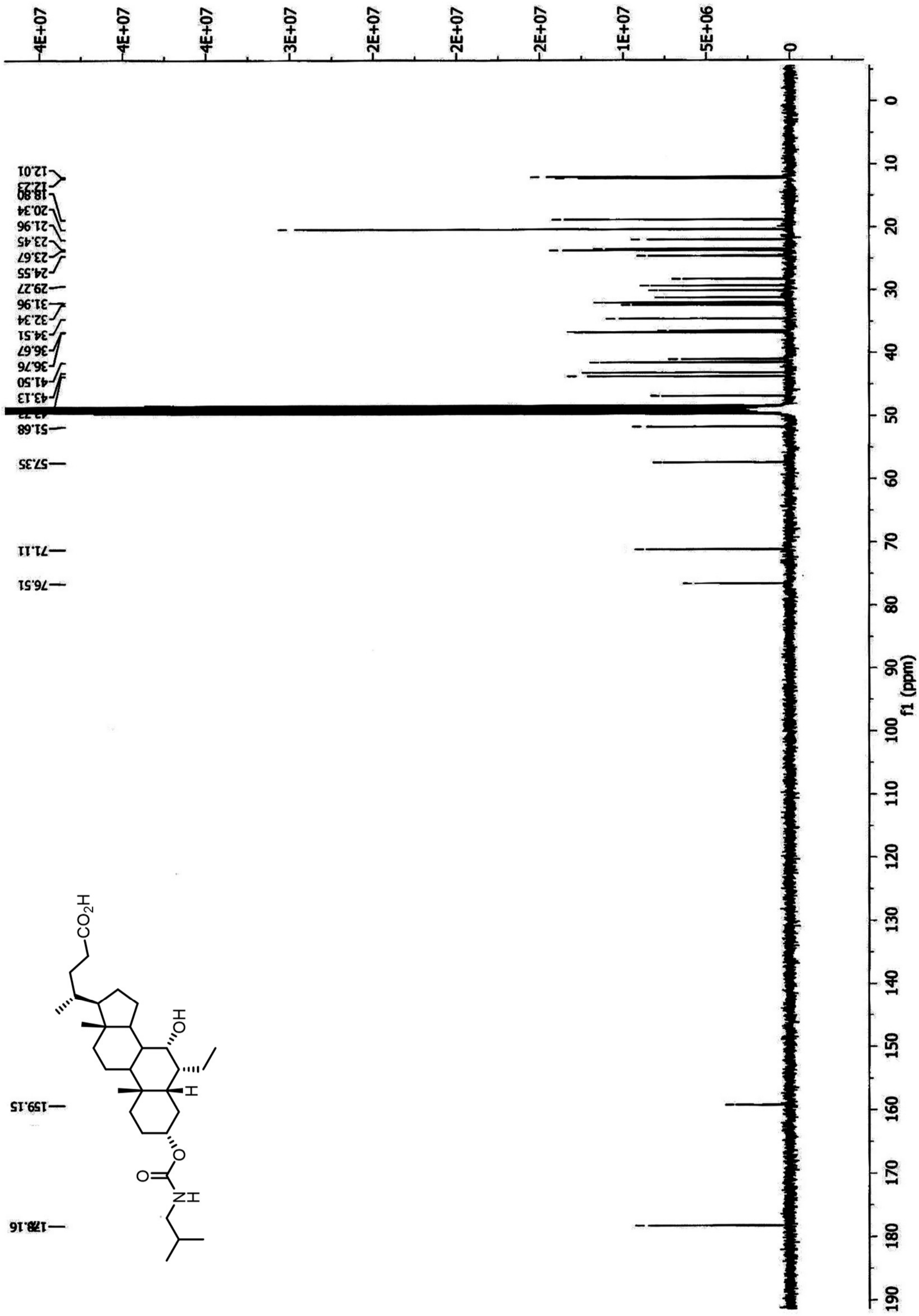

^{1}H-NMR (400 MHz, CDCl3) of 3α-[(((2′-morpholinoethyl)carbamoyl)oxy)]-7α-hydroxy-6α-ethyl-5β-cholan-24-oic acid (10)

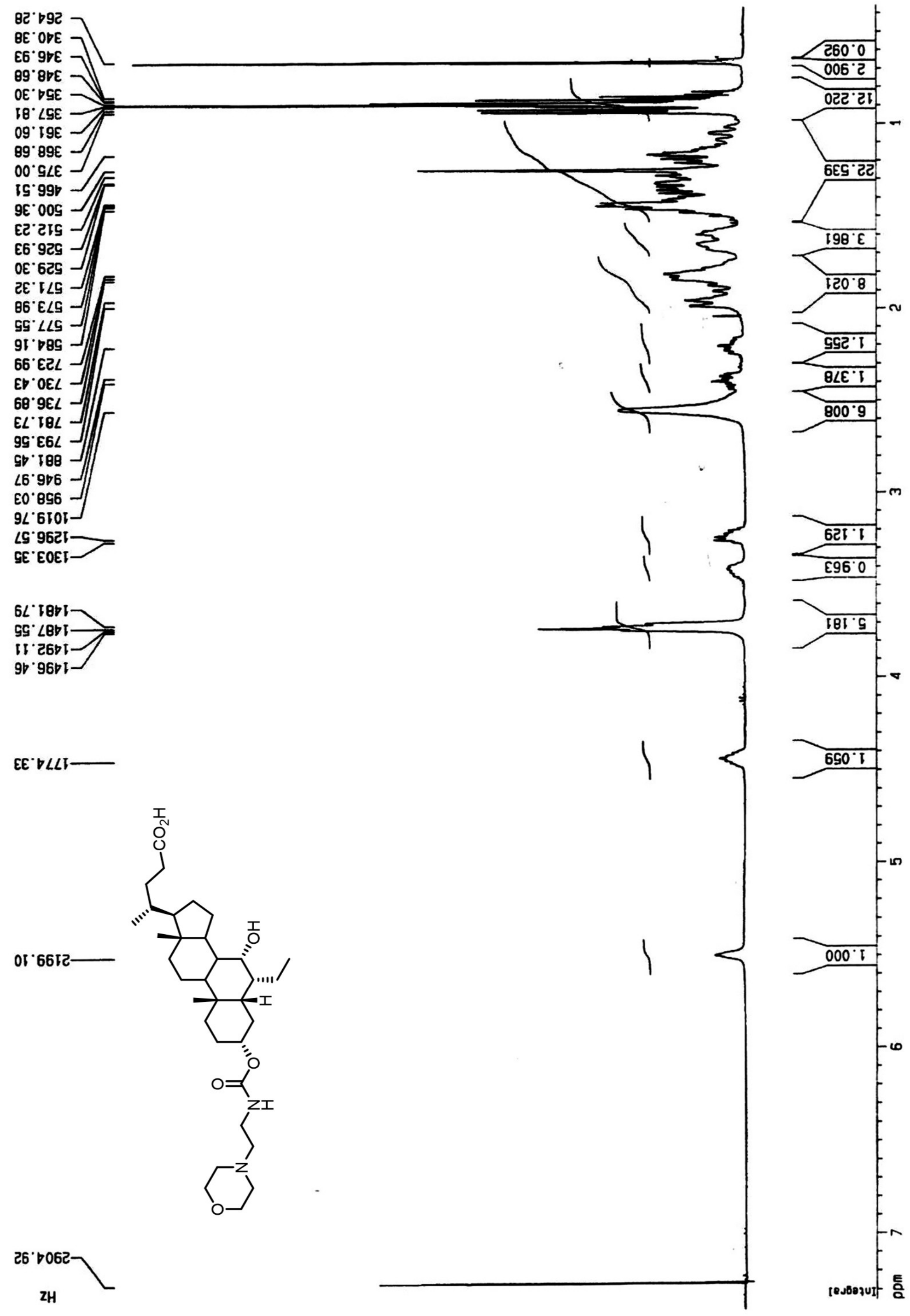

^{13}C-NMR (100 MHz, CDCl3) of 3α-[(((2'-morpholinoethyl)carbamoyl)oxy)]-7α-hydroxy-6α-ethyl-5β-cholan-24-oic acid (10)

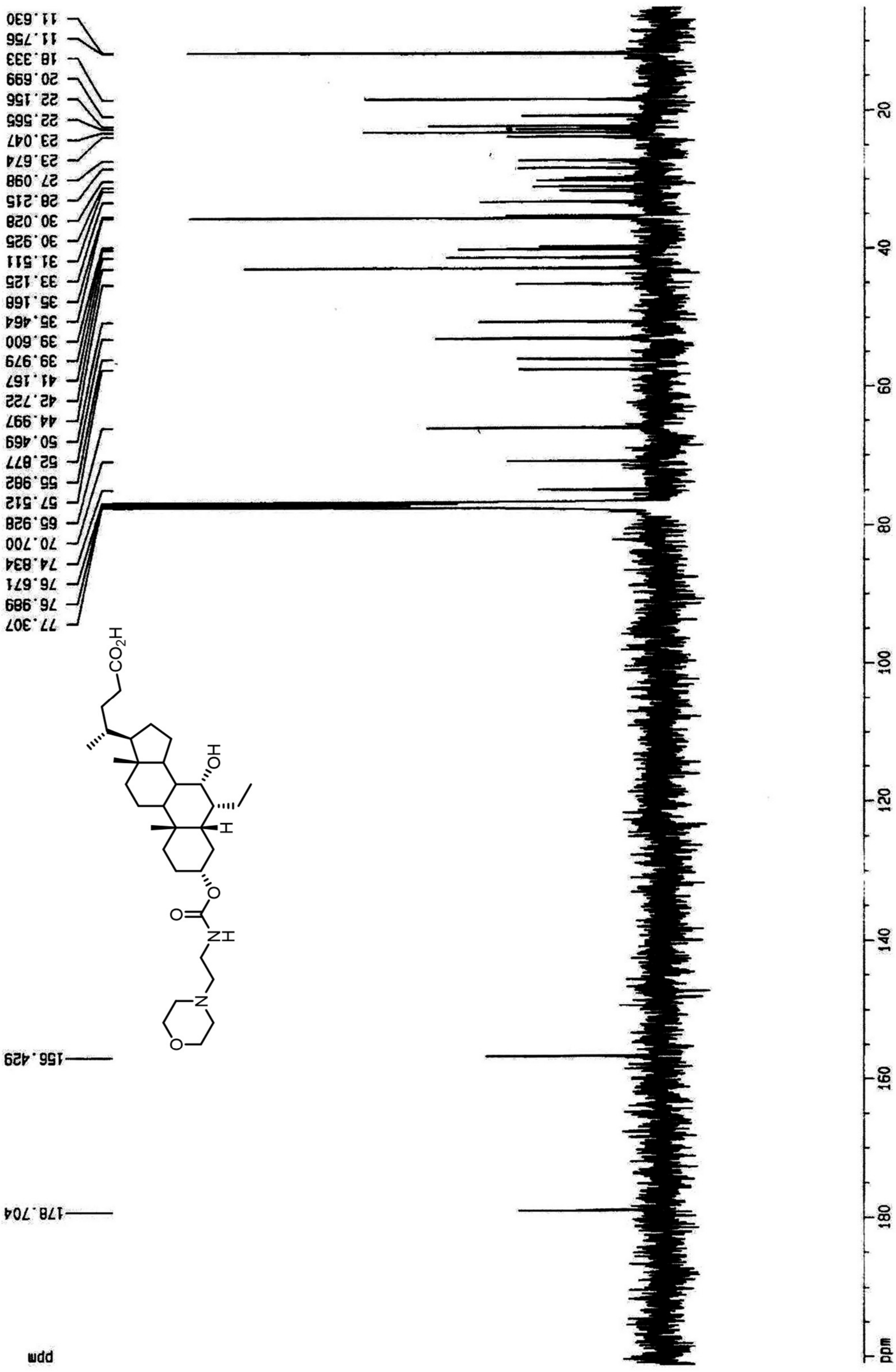

^{1}H-NMR (400 MHz, $CDCl_3$) of 3α-(maleyloxy)-7α-hydroxy-6α-ethyl-5β-cholan-24-oic acid (11)

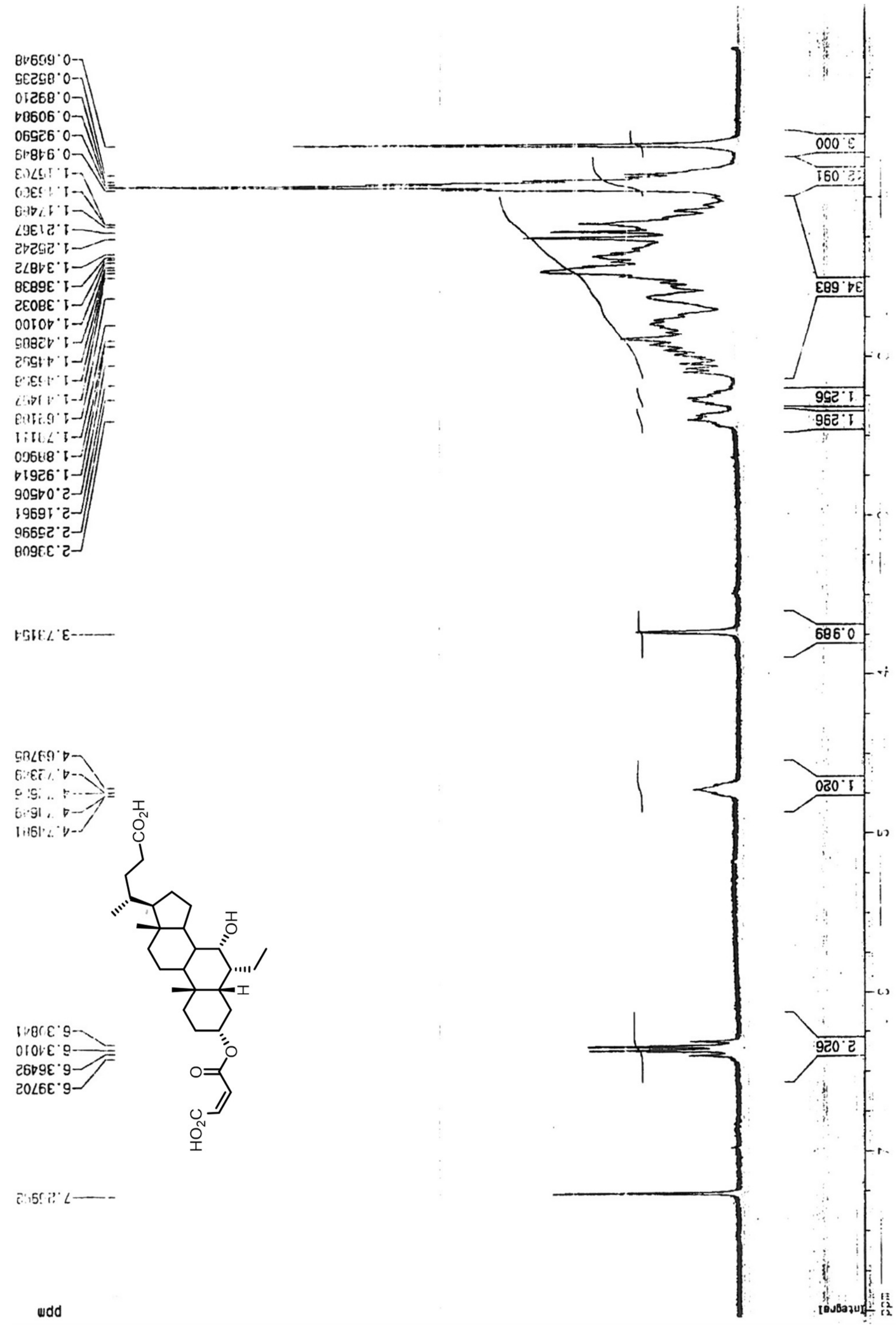

^{13}C-NMR (100 MHz, CD_3OD) of 3α-(maleyloxy)-7α-hydroxy-6α-ethyl-5β-cholan-24-oic acid (11)

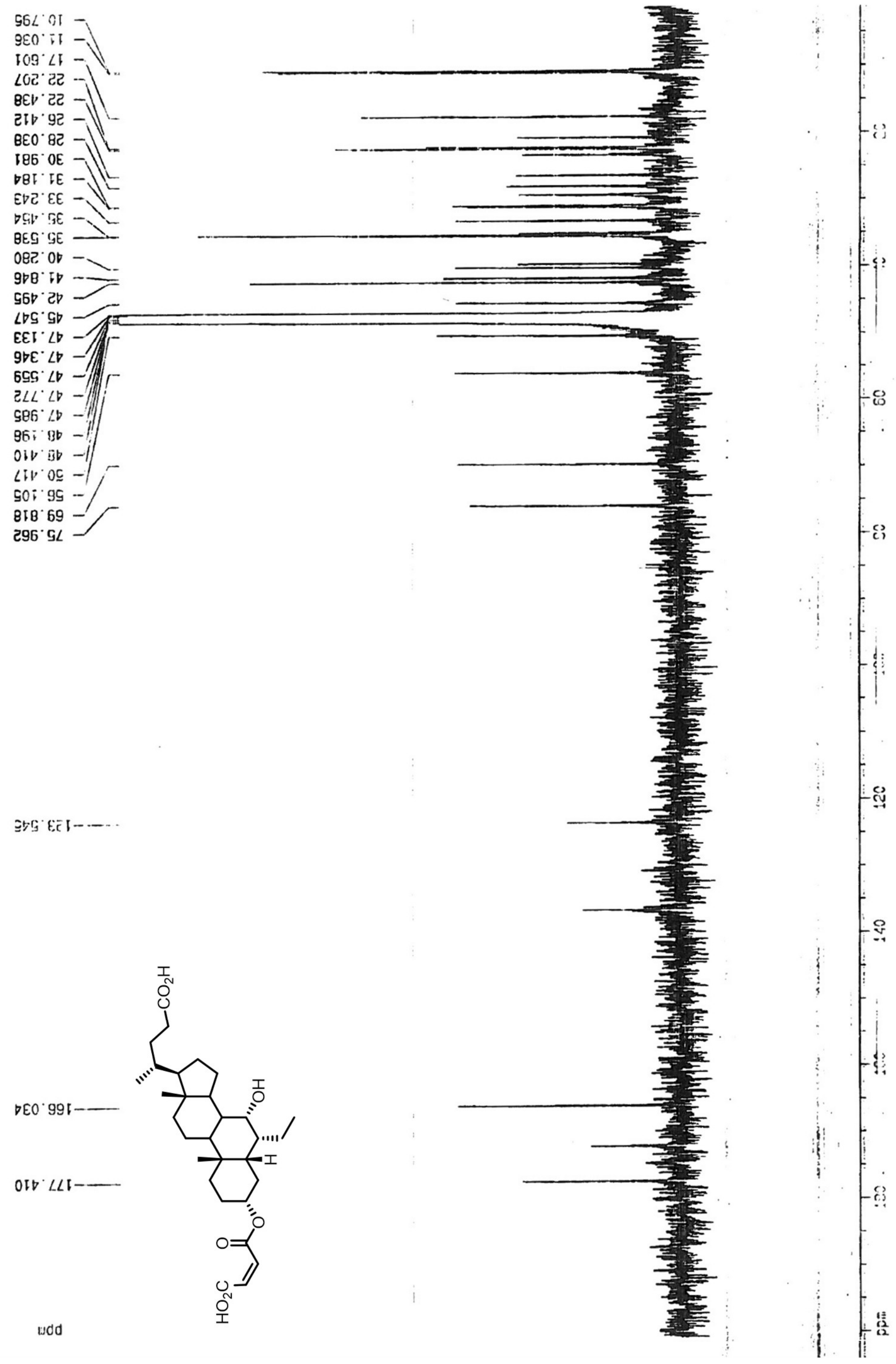

^{1}H-NMR (400 MHz, $CDCl_3$) of 3α-((3'-methylbutanoyl)oxy)-7α-hydroxy-6α-ethyl-5β-cholan-24-oic acid (12)

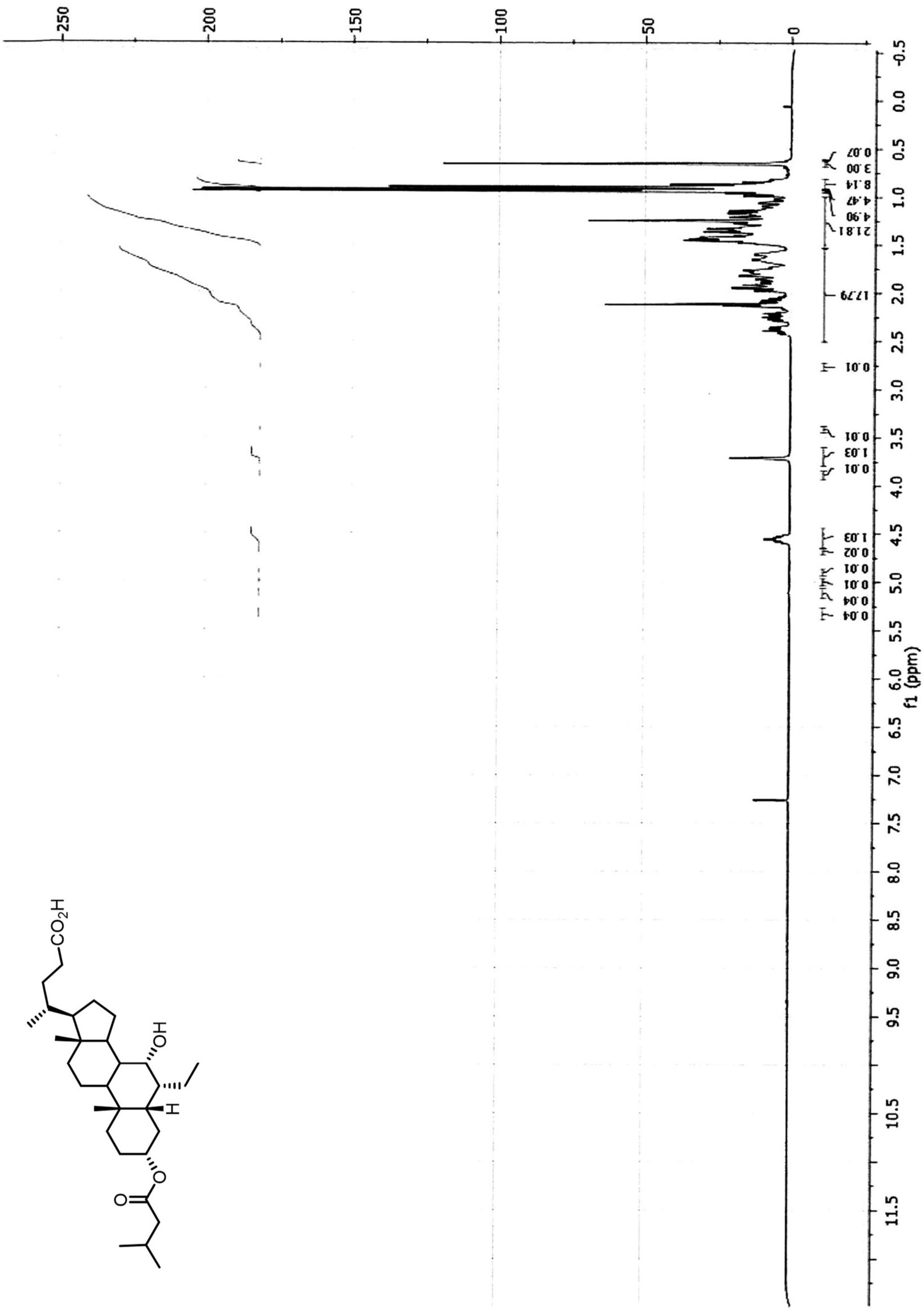

^{13}C-NMR (100 MHz, $CDCl_3$) of 3α-((3'-methylbutanoyl)oxy)-7α-hydroxy-6α-ethyl-5β-cholan-24-oic acid (12)

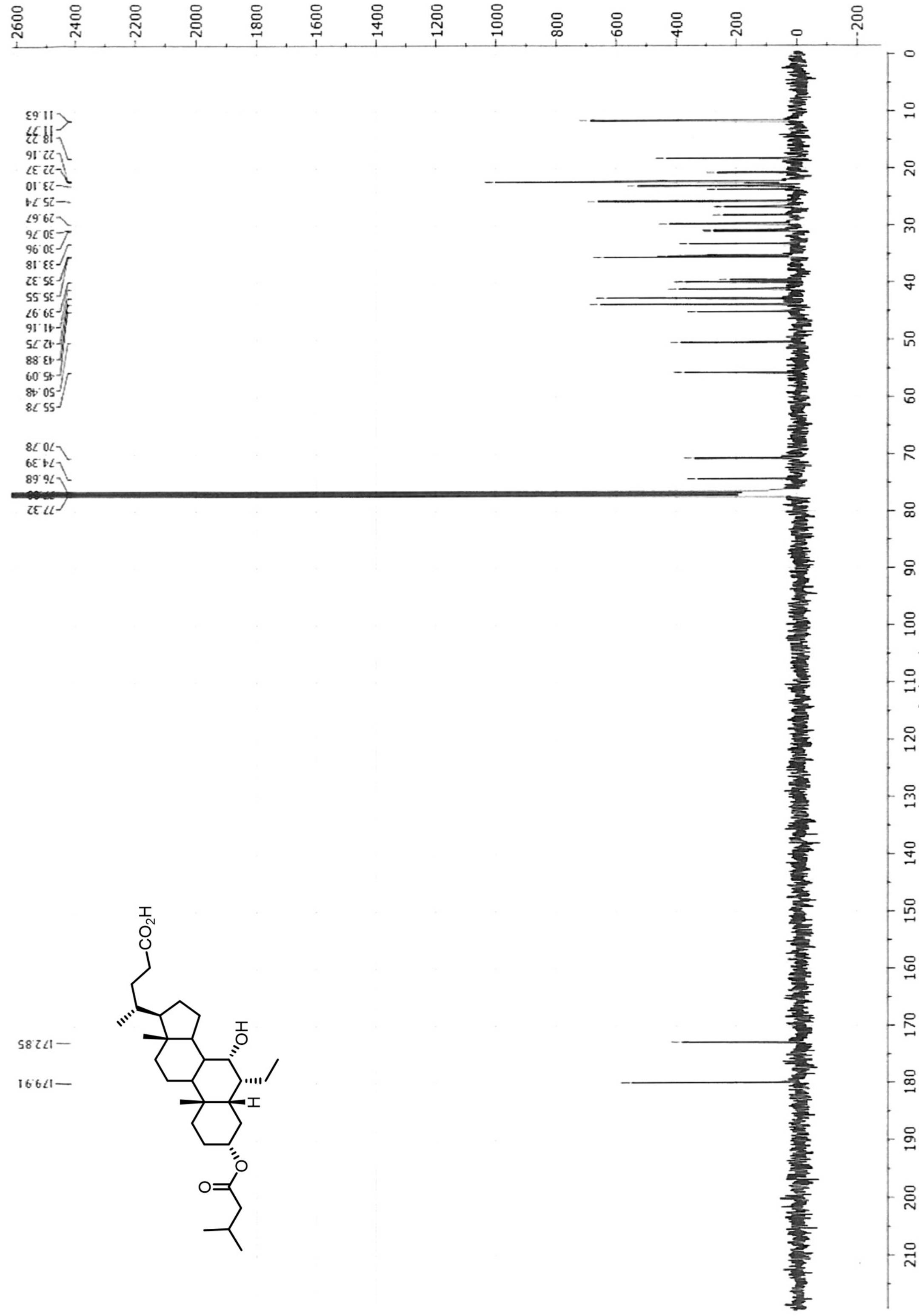

^{1}H-NMR (400 MHz, CDCl3) of methyl 3-oxo-7α-dihydroxy-6α-ethyl-5β-cholan-24-oate (21)

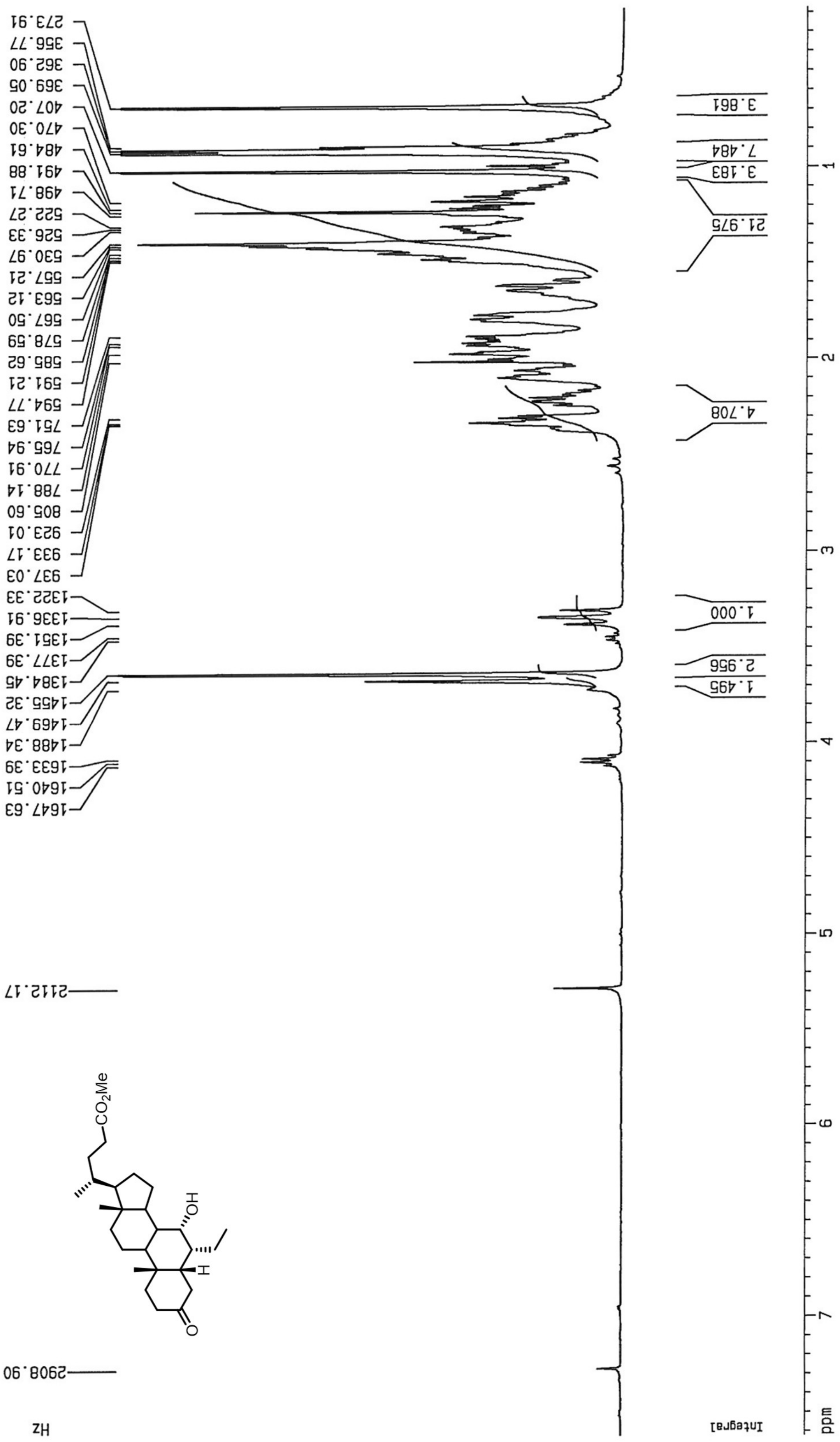

^{1}H-NMR (400 MHz, CDCl$_3$) of methyl 3-(3'-methylbutanamido)-7α-dihydroxy-6α-ethyl-5β-cholan-24-oate (22)

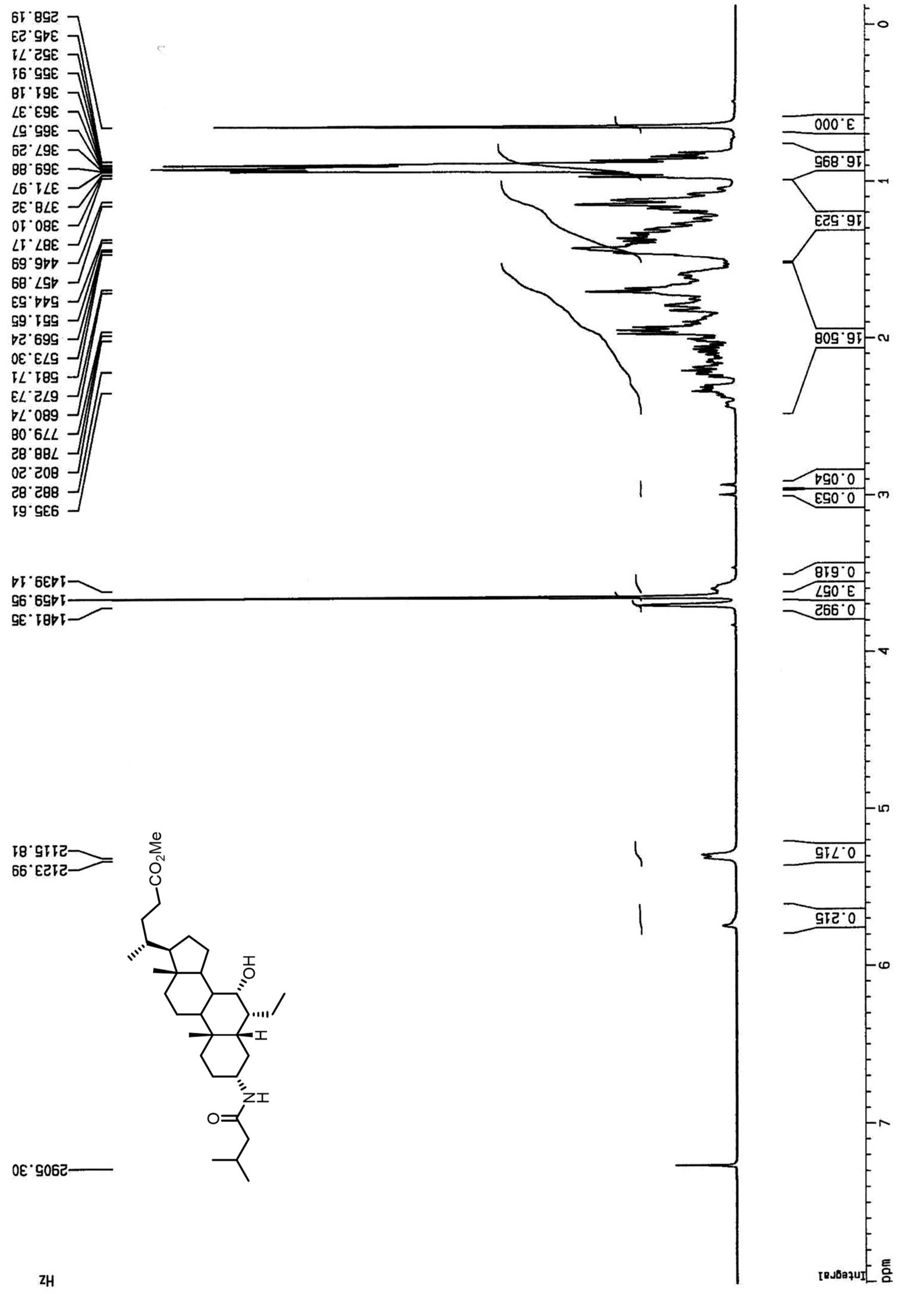

^{1}H-NMR (400 MHz, $CDCl_3$) of 3-(3'-methylbutanamido)-7α-dihydroxy-6α-ethyl-5β-cholan-24-oic acid (13)

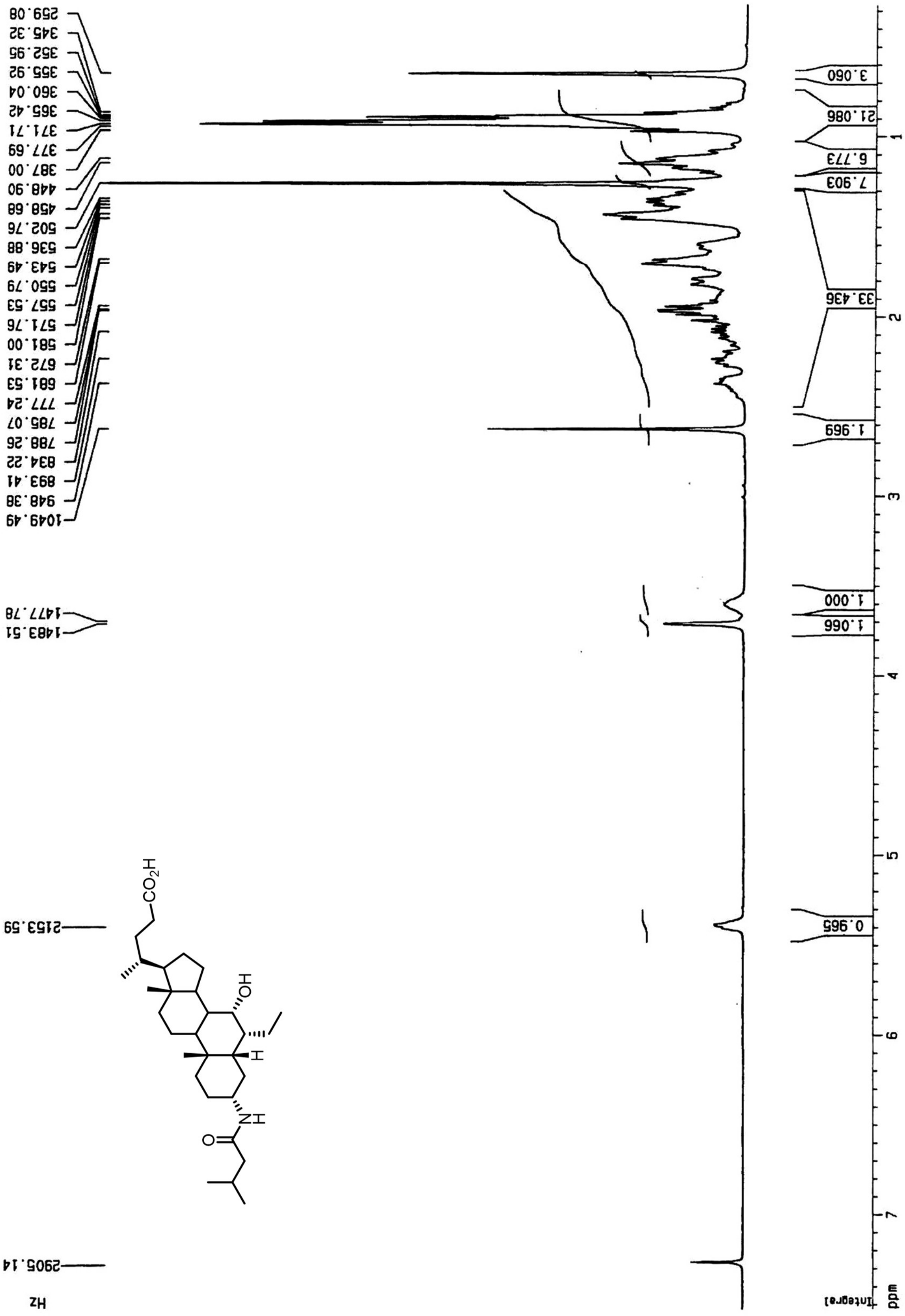

^{13}C-NMR (100 MHz, $CDCl_3$) of 3-(3'-methylbutanamido)-7α-dihydroxy-6α-ethyl-5β-cholan-24-oic acid (13)

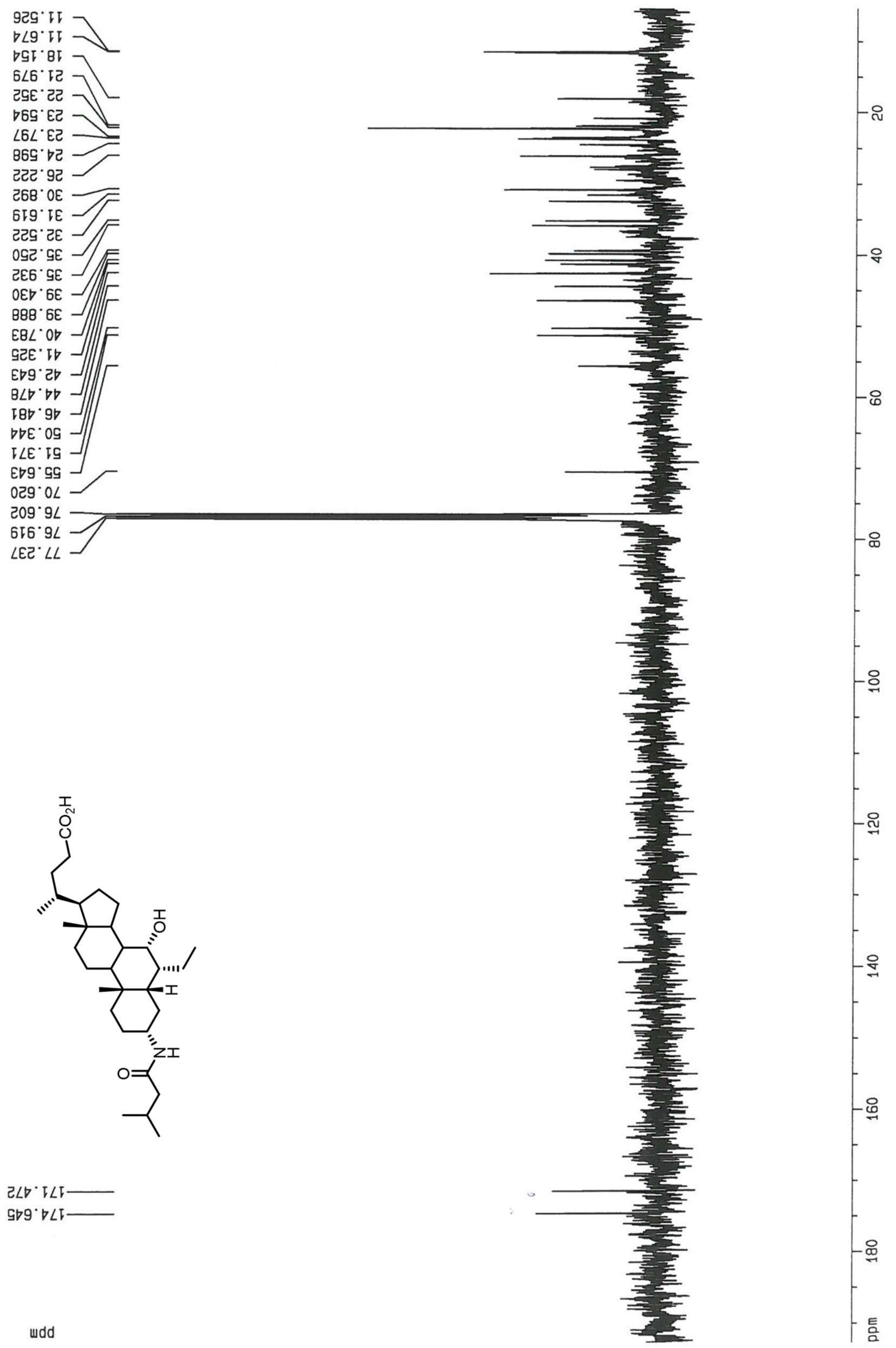

^{1}H-NMR (400 MHz, CDCl$_3$) of benzyl 3α,7α-dimethoxymethyloxy-6α-ethyl-5β-cholan-24-oate (23)

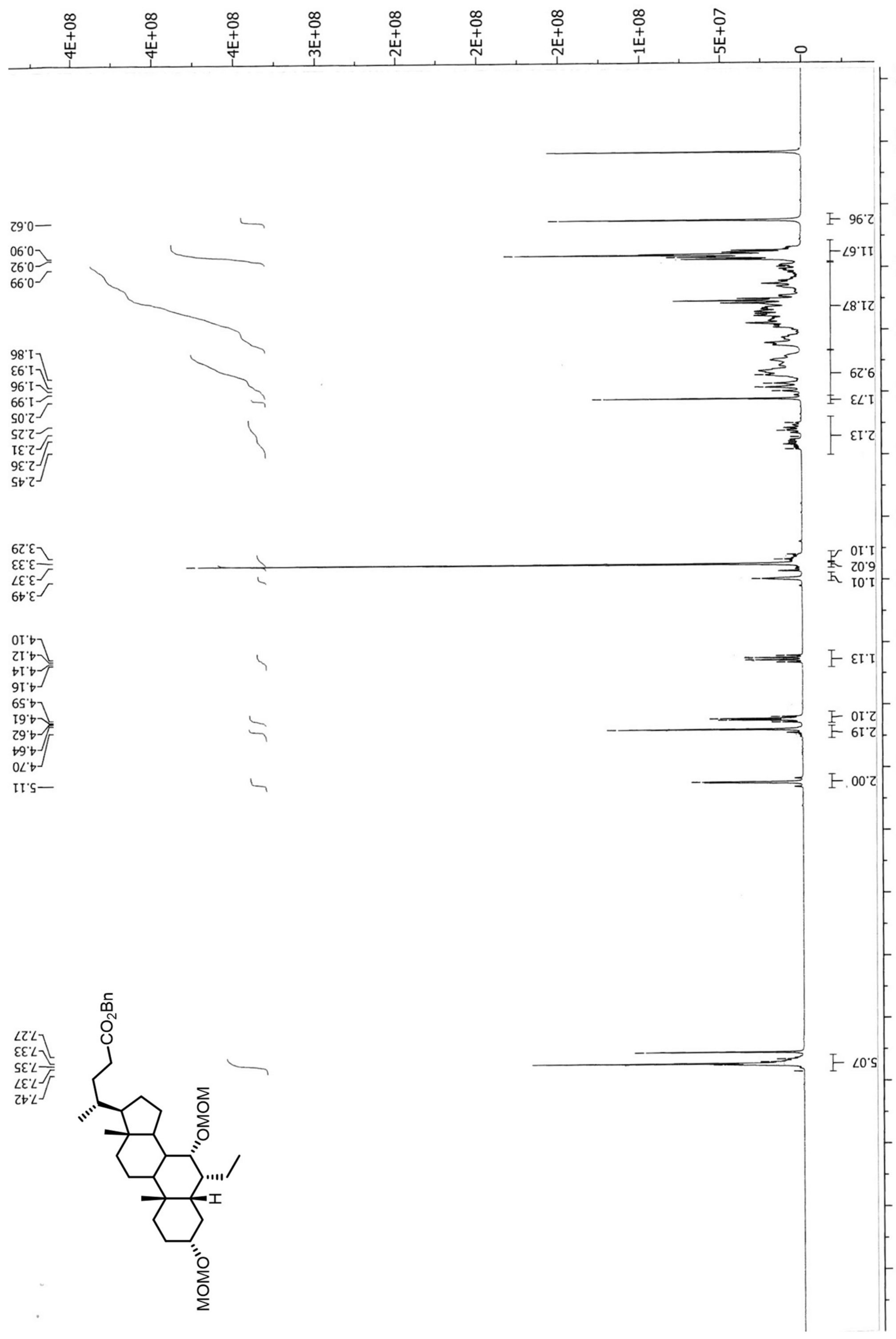

^{1}H-NMR (400 MHz, CDCl$_3$) of benzyl 23-(*R*)-methyl 3α,7α-dihydroxy-6α-ethyl-5β-cholan-24-oate (24a)

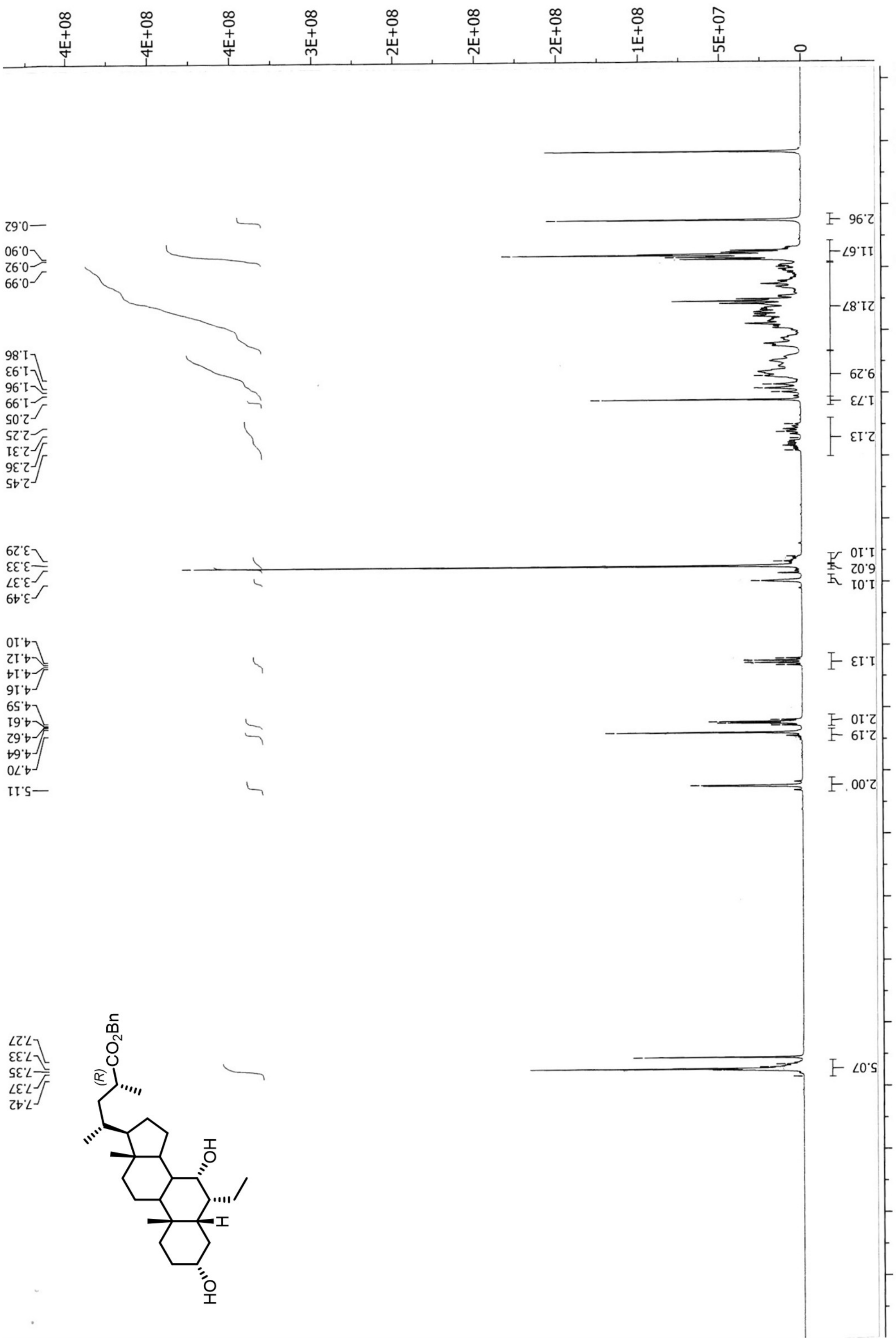

^{13}C-NMR (100 MHz, $CDCl_3$) of benzyl 23-(*R*)-methyl 3α,7α-dihydroxy-6α-ethyl-5β-cholan-24-oate (24a)

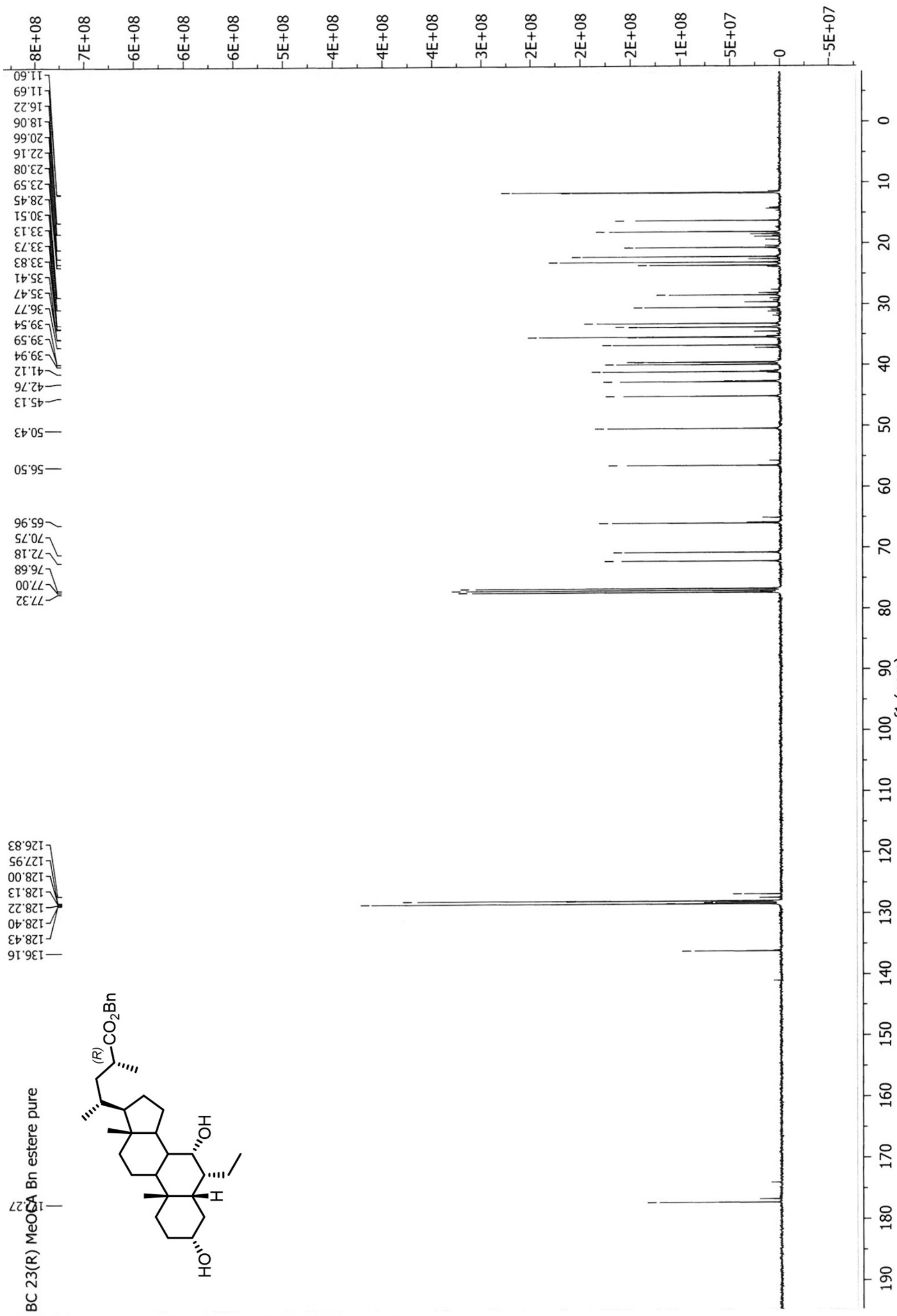

^{1}H-NMR (400 MHz, $CDCl_3$) of benzyl 23-(*S*)-methyl 3α,7α-dihydroxy-6α-ethyl-5β-cholan-24-oate (24b)

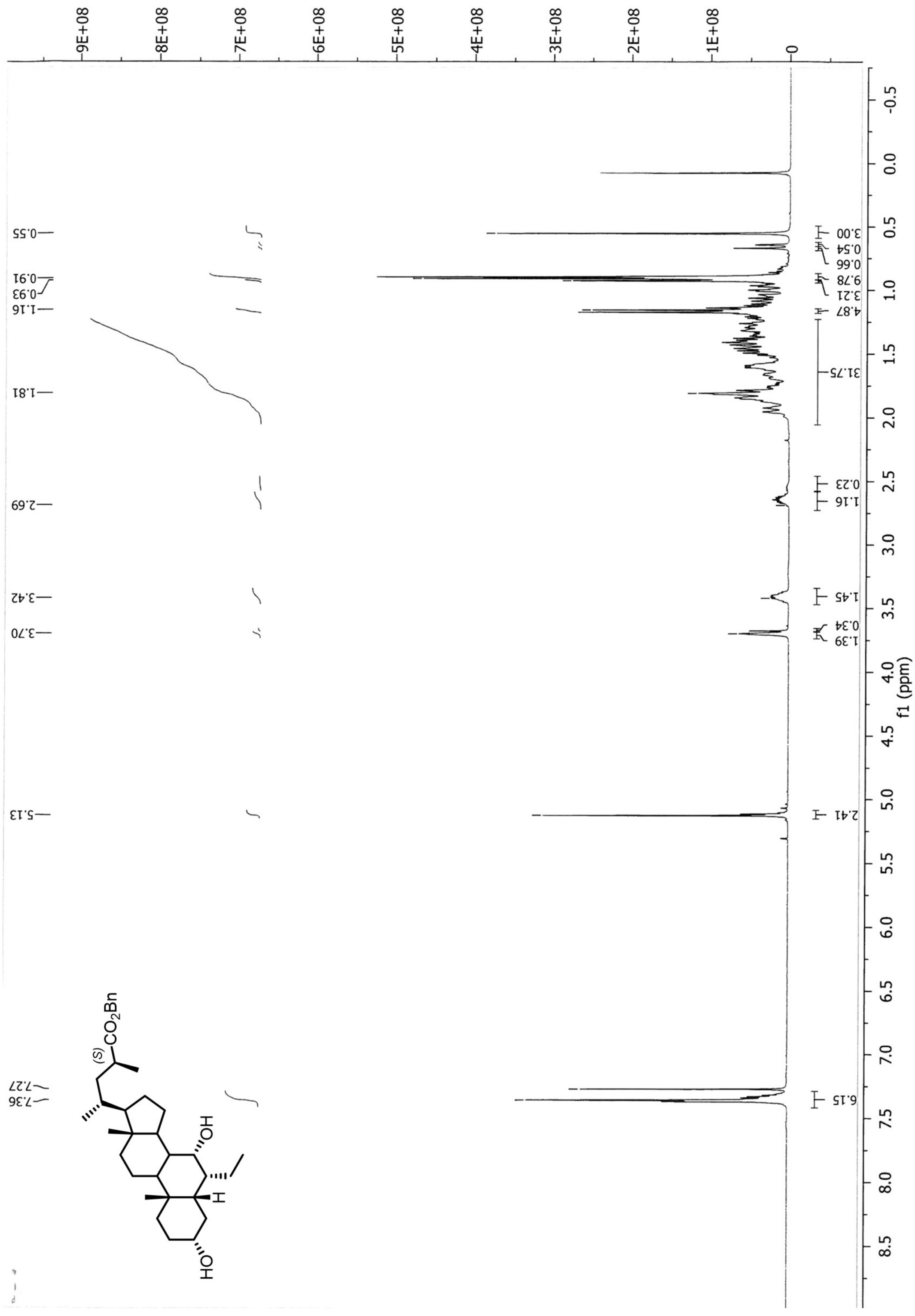

^{13}C-NMR (100 MHz, CDCl$_3$) of benzyl 23-(*S*)-methyl 3α,7α-dihydroxy-6α-ethyl-5β-cholan-24-oate (24b)

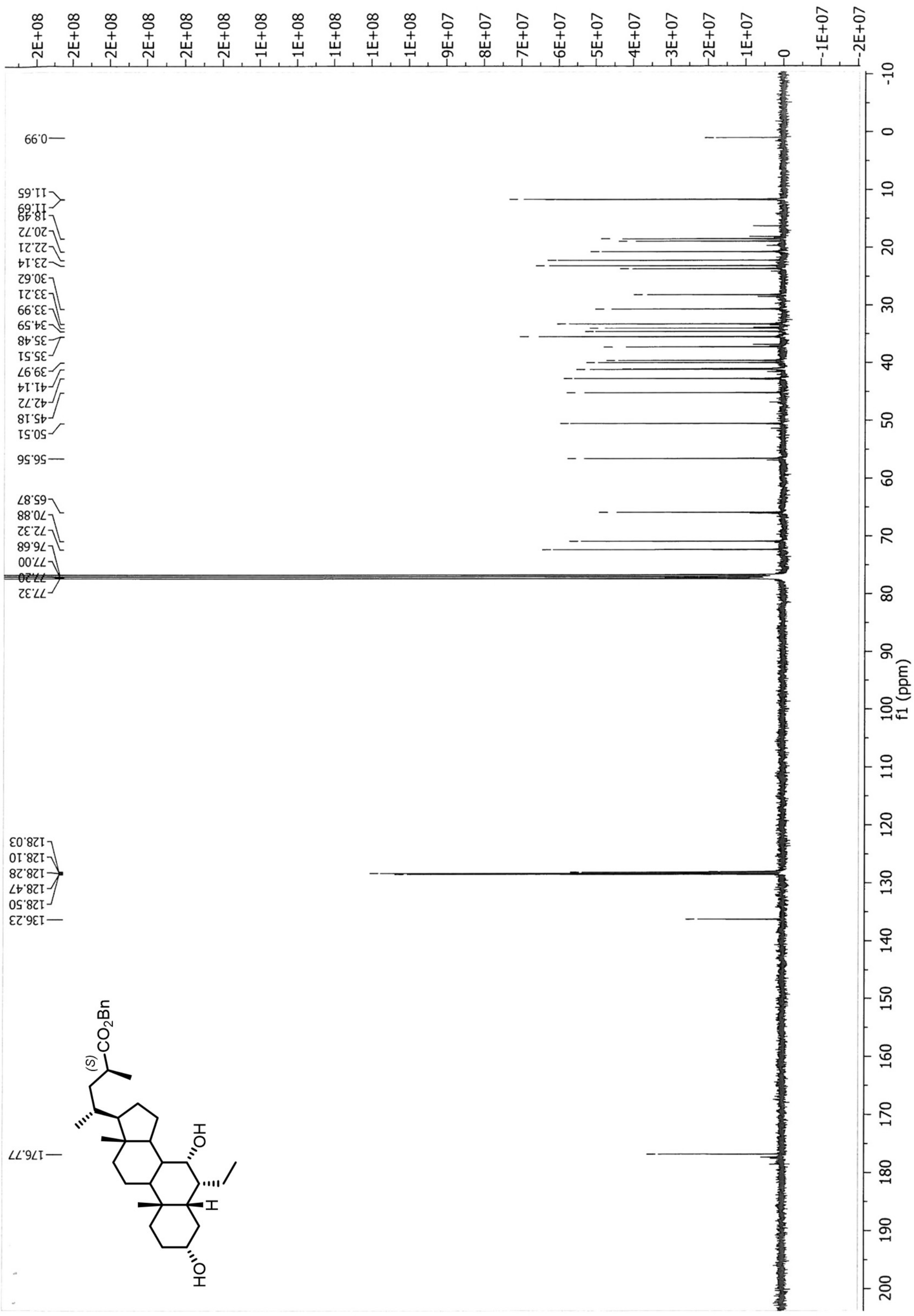

^{1}H-NMR (400 MHz, $CDCl_3$) of benzyl 23-(*R*)-methyl 3α-[(((4'-nitrophenoxy)carbonyl)oxy)]-7α-dihydroxy-6α-ethyl-5β-cholan-24-oate (25a)

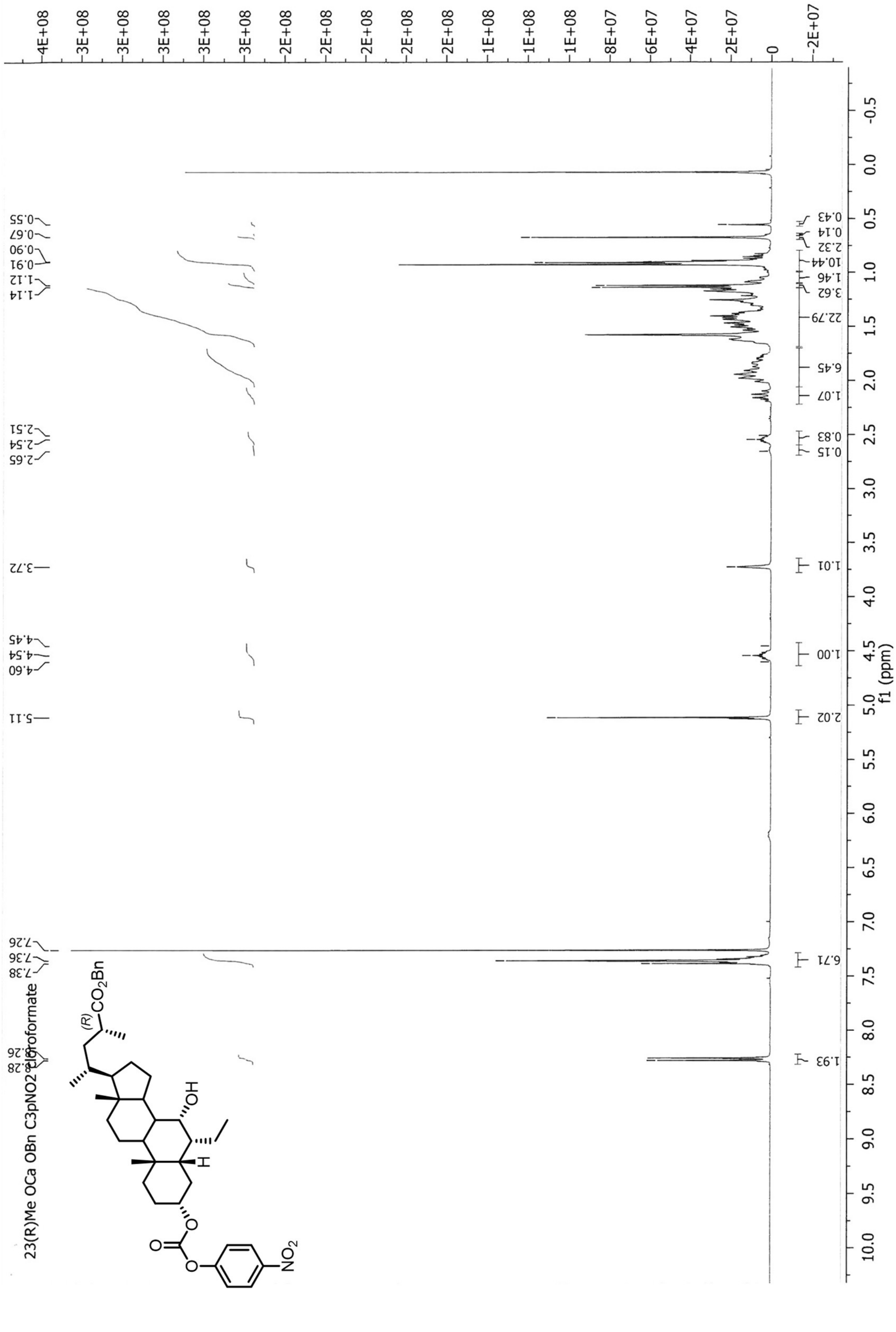

^{1}H-NMR (400 MHz, CDCl$_3$) of benzyl 23-(*S*)-methyl 3α-[(((4'-nitrophenoxy)carbonyl)oxy)]-7α-dihydroxy-6α-ethyl-5β-cholan-24-oate (25b)

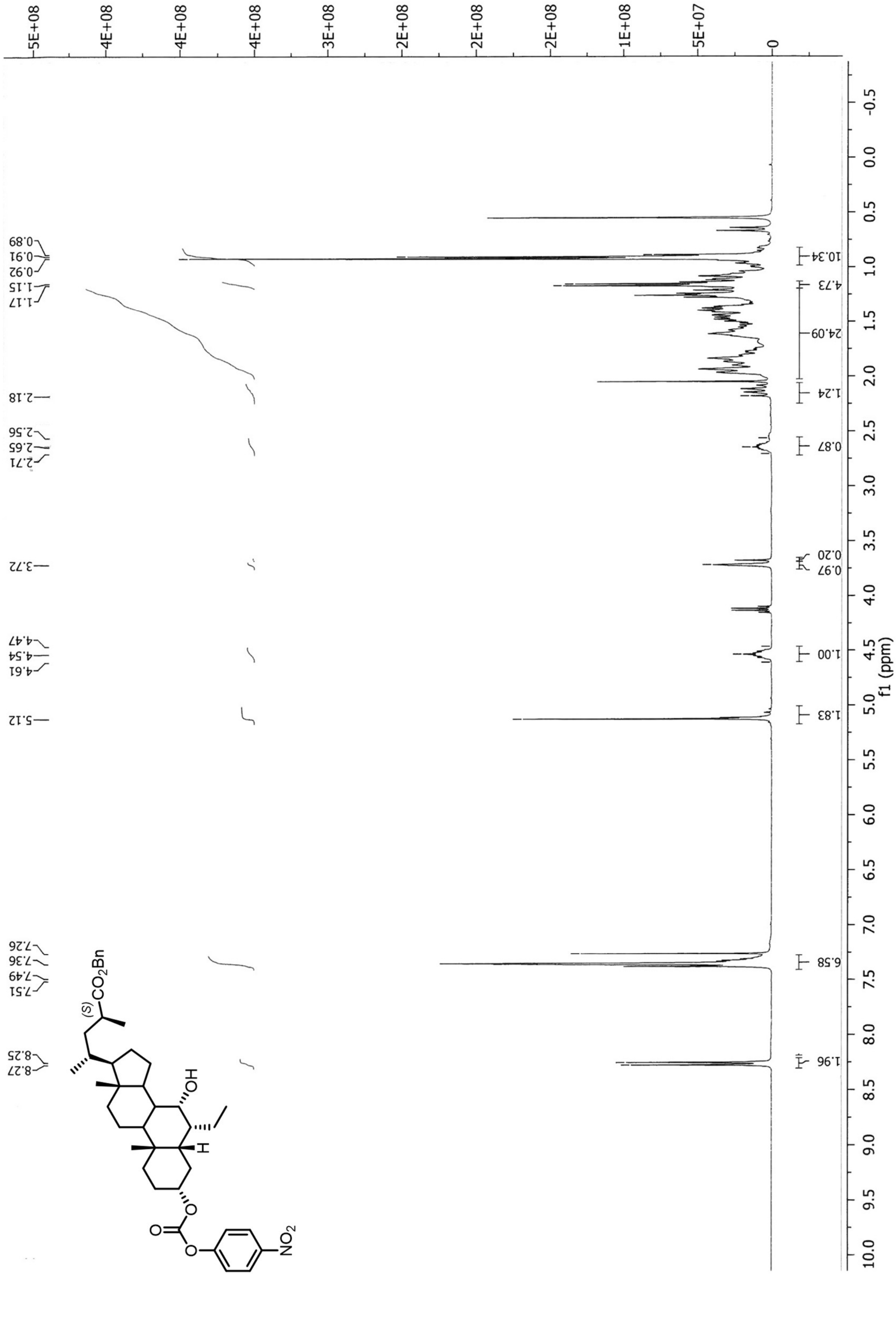

^{1}H-NMR (600 MHz, DMSO-d$_6$) of 23-(*R*)-methyl 3α-[((((*S*)-1'-carboxy-2'-methylpropyl)carbamoyl)oxy)]-7α-hydroxy-6α-ethyl-5β-cholan-24-oic acid (14a)

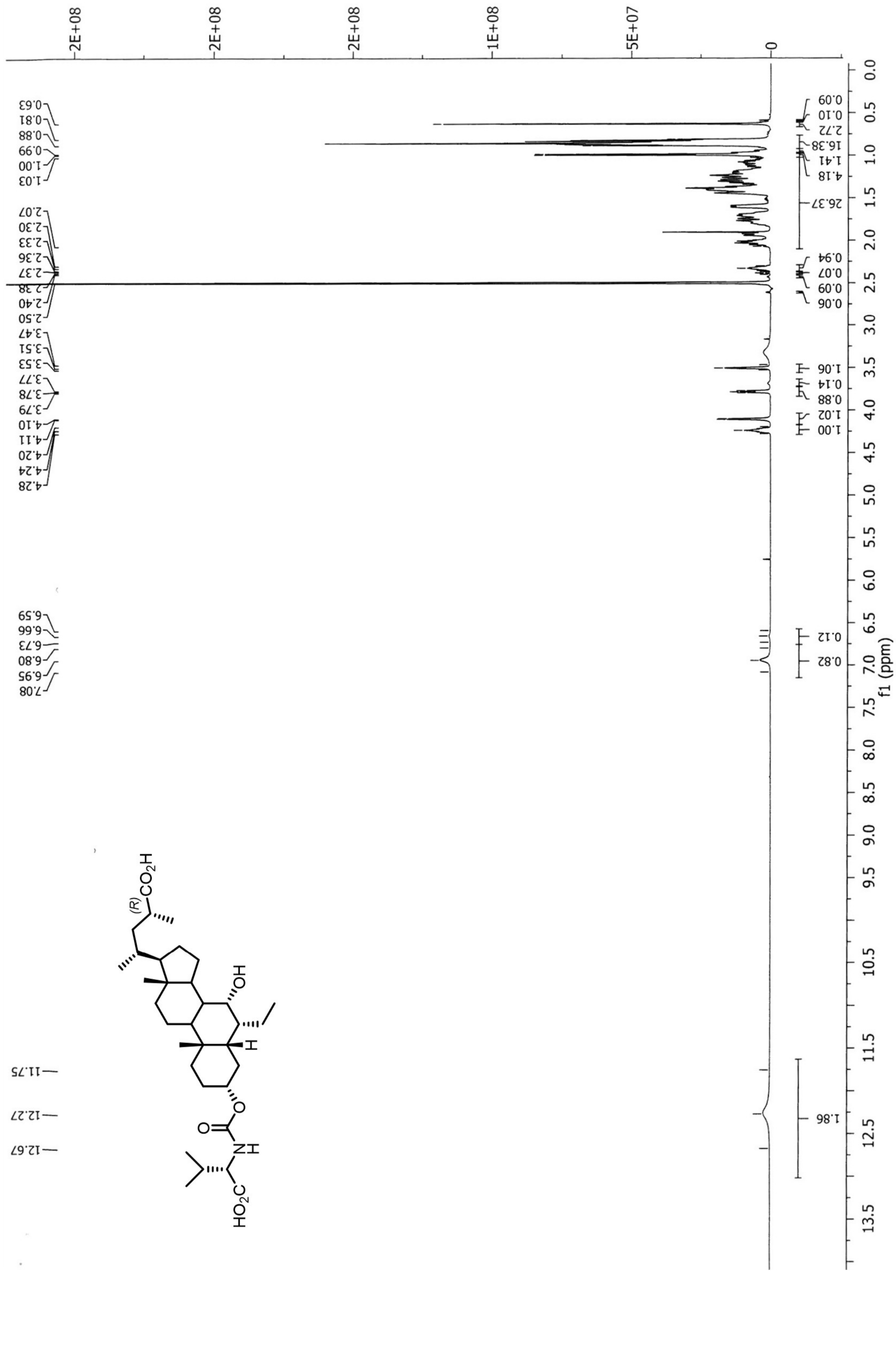

^{13}C-NMR (150 MHz, DMSO-d$_6$) of 23-(*R*)-methyl 3α-[((((*S*)-1'-carboxy-2'-methylpropyl)carbamoyl)oxy)]-7α-hydroxy-6α-ethyl-5β-cholan-24-oic acid (14a)

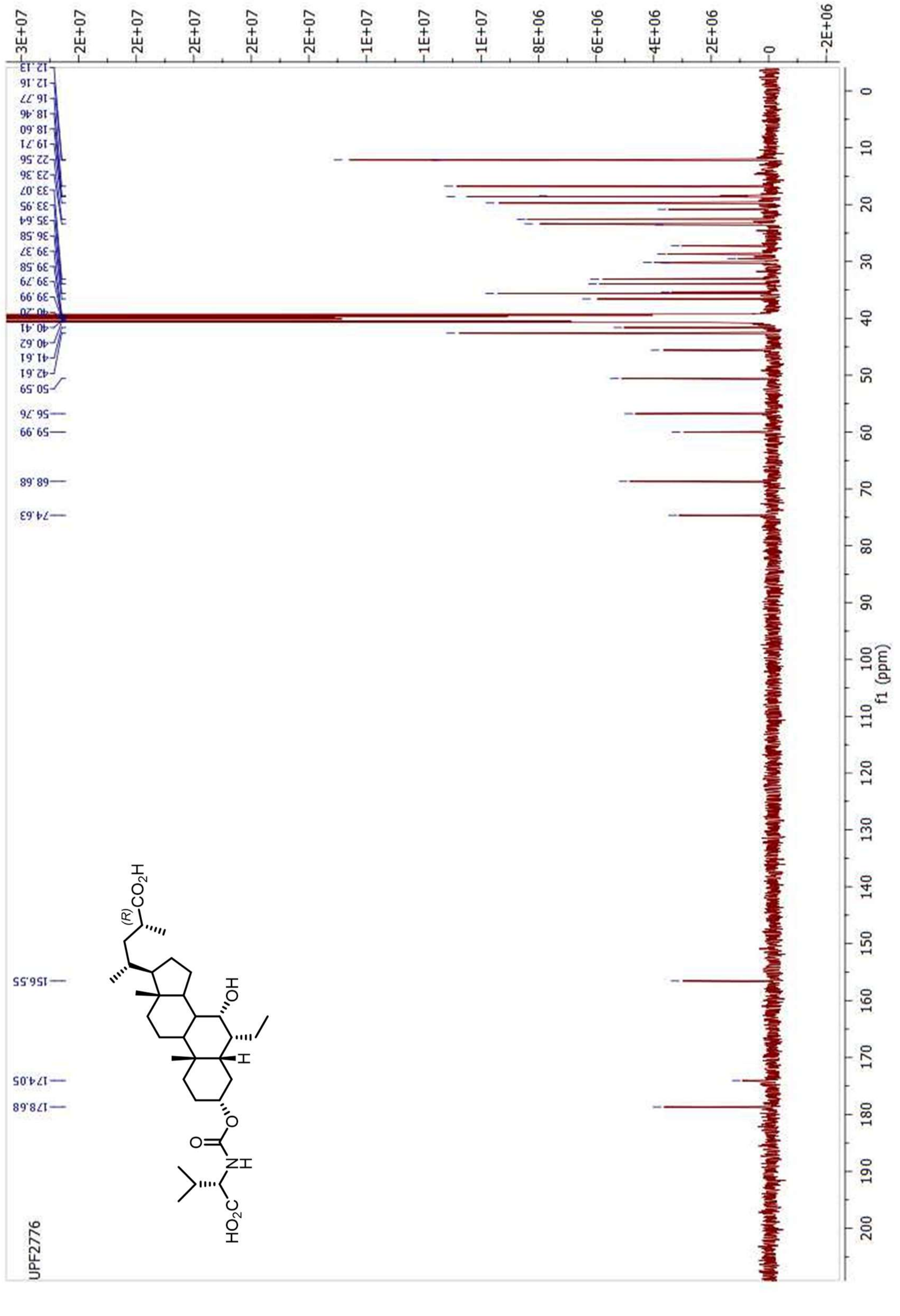

^{1}H-NMR (600 MHz, DMSO-d$_6$) of 23-(*S*)-methyl 3α-[((((*S*)-1’-carboxy-2’-methylpropyl)carbamoyl)oxy)]-7α-hydroxy-6α-ethyl-5β-cholan-24-oic acid (14b)

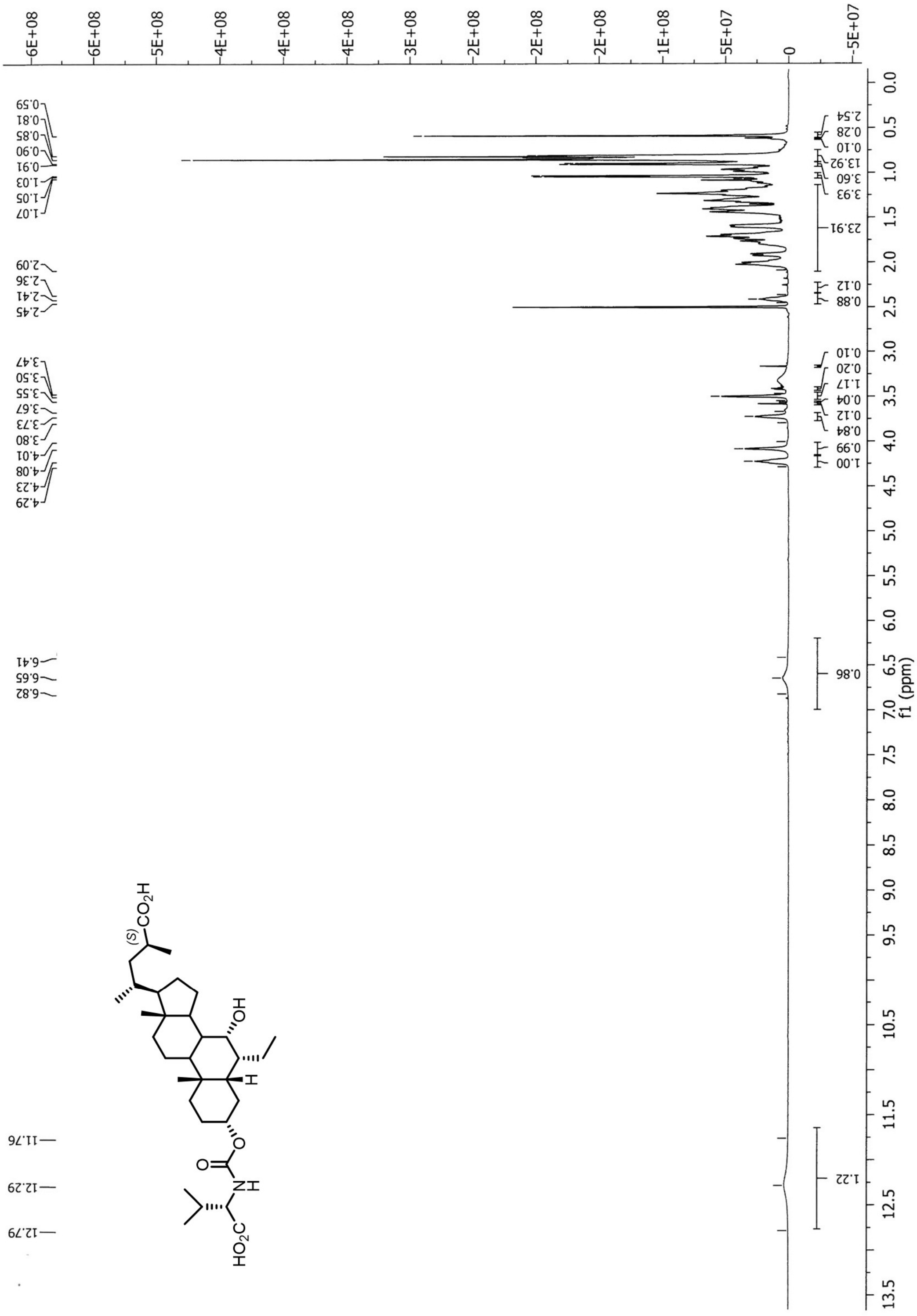

^{13}C-NMR (150 MHz, DMSO-d$_6$) of 23-(*S*)-methyl 3α-[((((*S*)-1'-carboxy-2'-methylpropyl)carbamoyl)oxy)]-7α-hydroxy-6α-ethyl-5β-cholan-24-oic acid (14b)

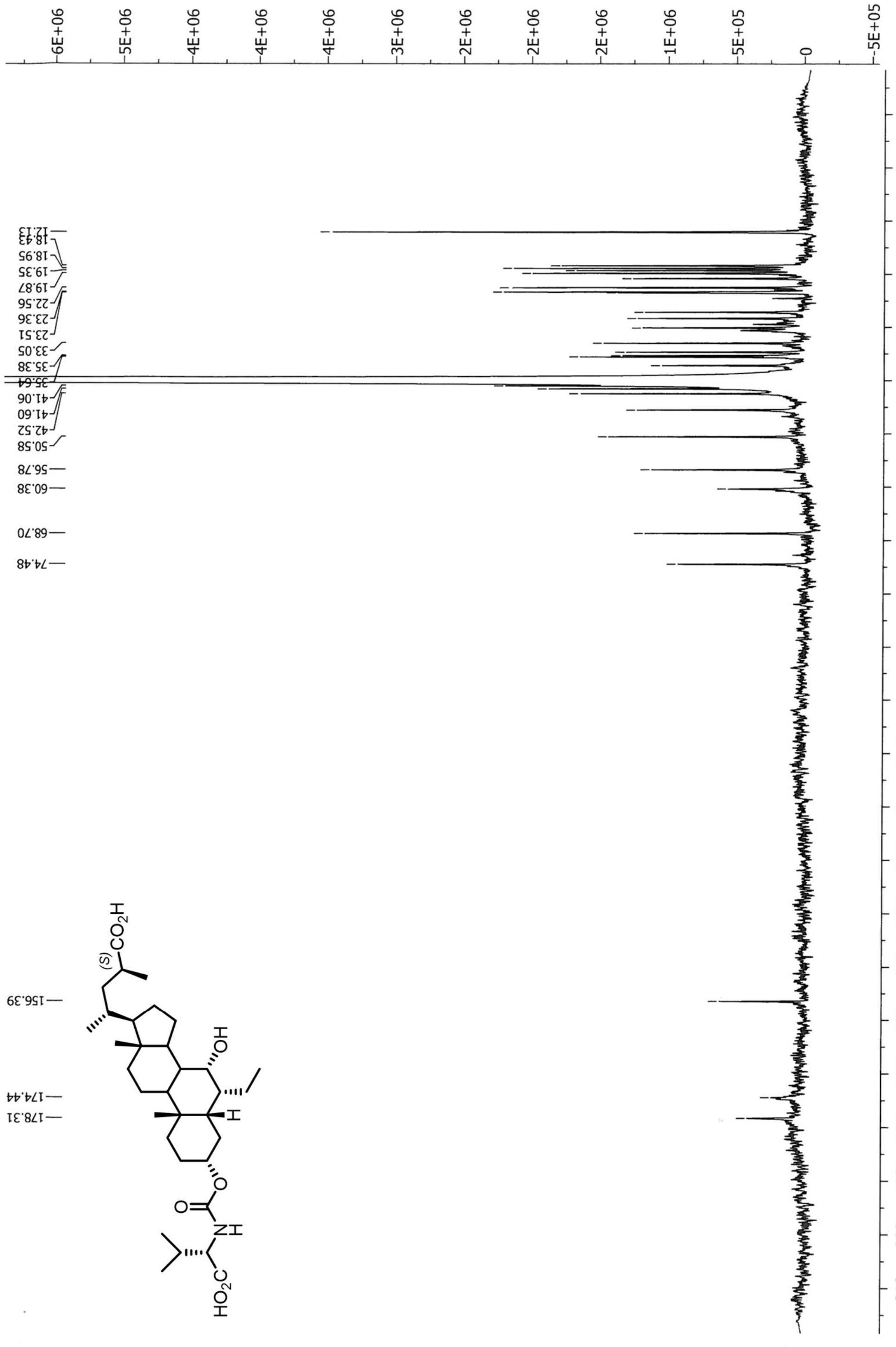

^{1}H-NMR (400 MHz, $CDCl_3$) of 24,24-diphenyl 3α,7α-diacetoxy-6α-ethyl-5β-cholan-23-ene (28)

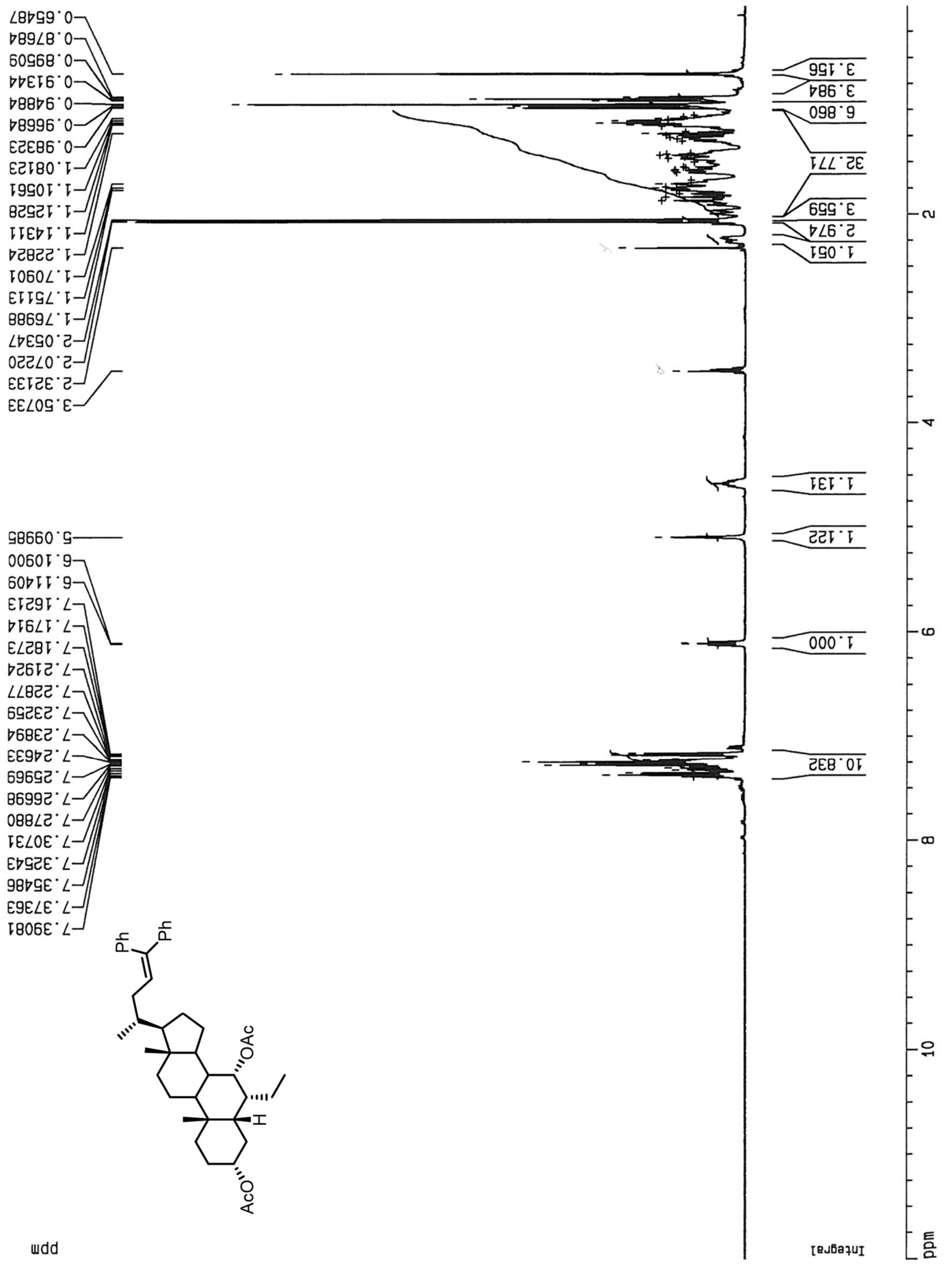

^{1}H-NMR (400 MHz, CD_3OD) of 3α,7α-dihydroxy-6α-ethyl-5β-cholan-24-*nor*-23-oic acid (26)

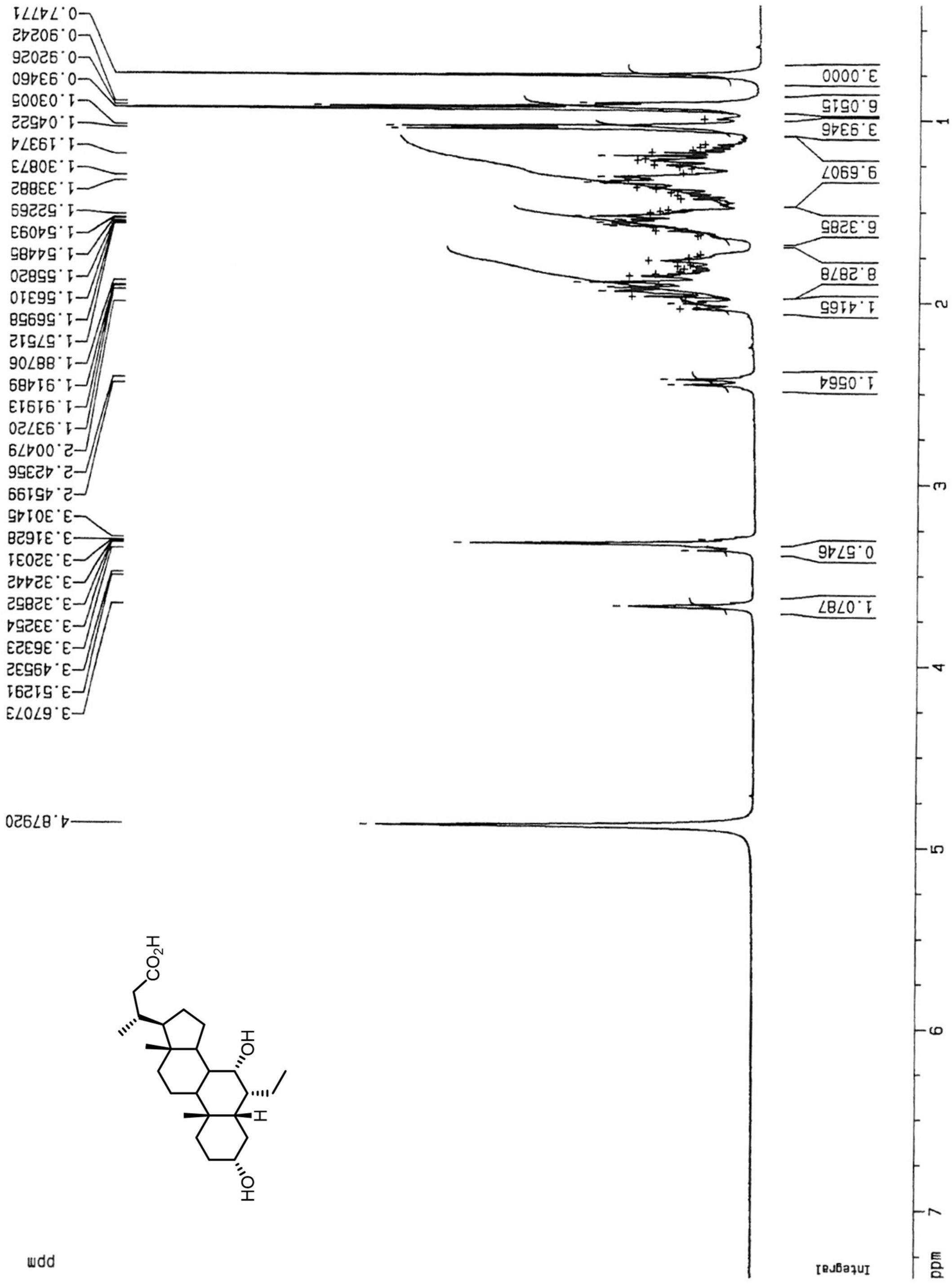

^{13}C-NMR (100 MHz, CD$_3$OD) of 3α,7α-cihydroxy-6α-ethyl-5β-cholan-24-*nor*-23-oic acid (26)

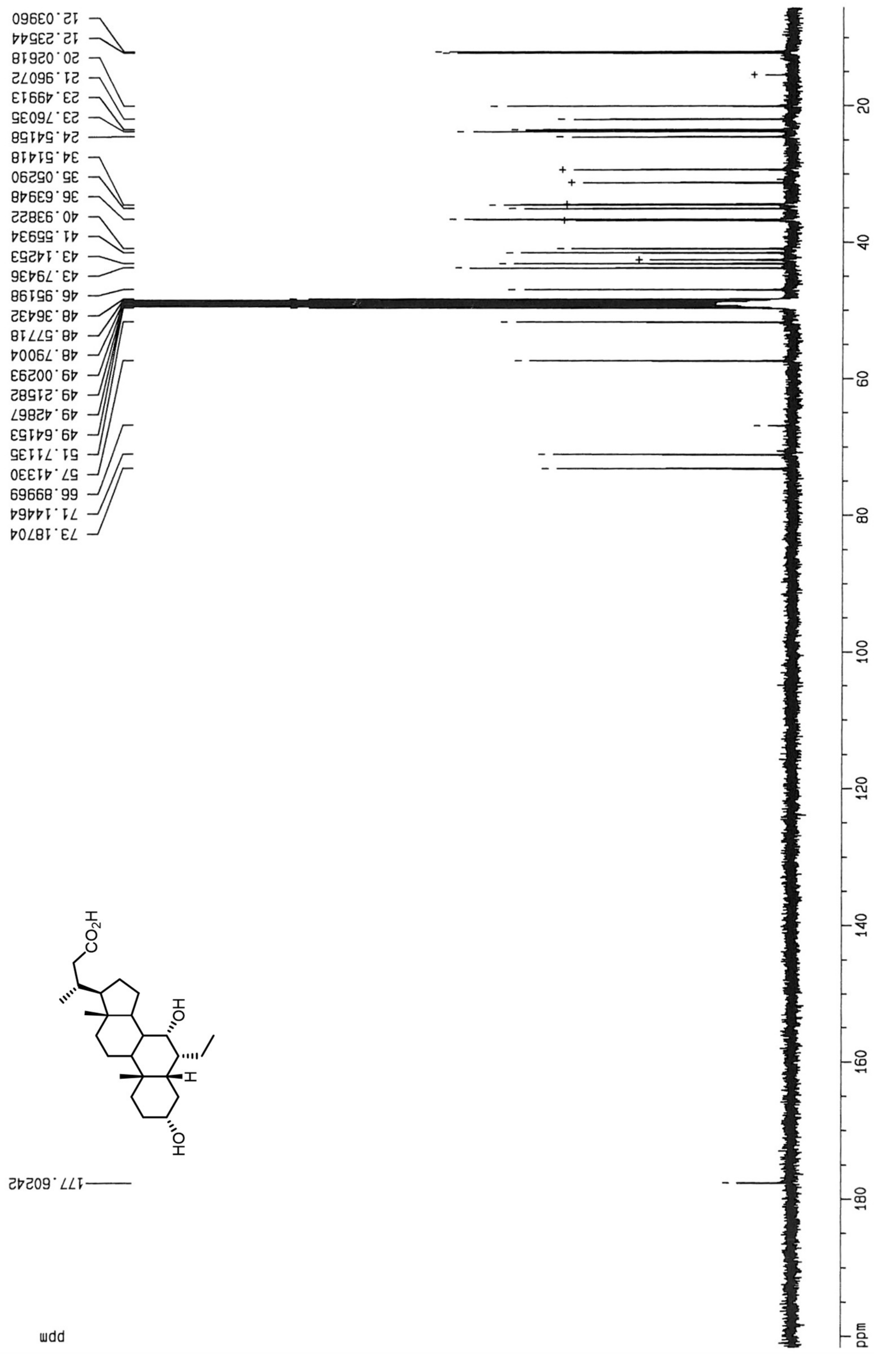

^{1}H-NMR (400 MHz, $CDCl_3$) of benzyl 3α-[(((4'-nitrophenoxy)carbonyl)oxy)]-7α-dihydroxy-6α-ethyl-5β-cholan-24-*nor*-23-oate (29)

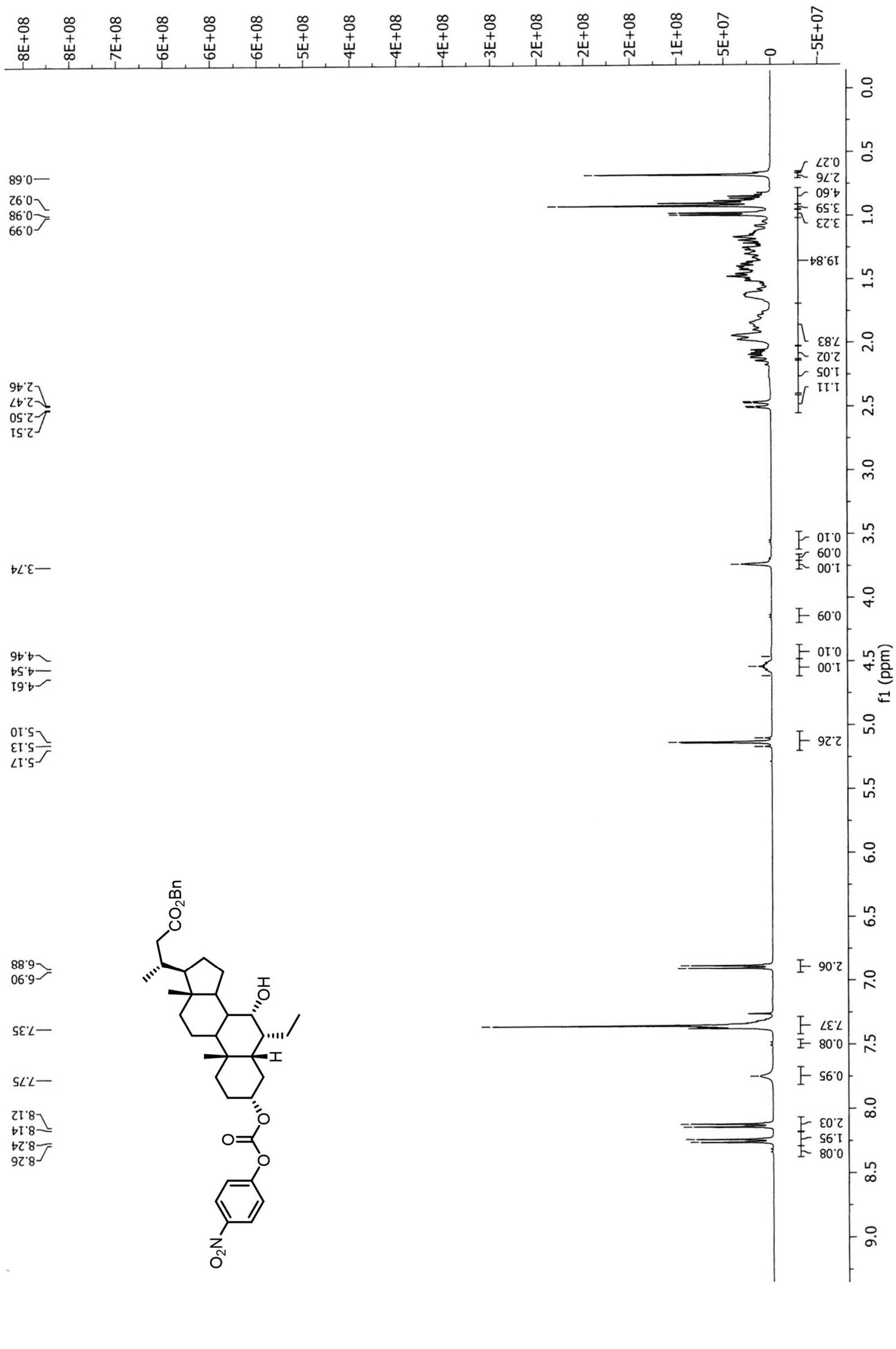

^{1}H-NMR (400 MHz, CD_3OD) of 3α-[((((*S*)-1'-carboxy-2'-methylpropyl)carbamoyl)oxy)]-7α-hydroxy-6α-ethyl-5β-cholan-24-*nor*-23-oic acid (15)

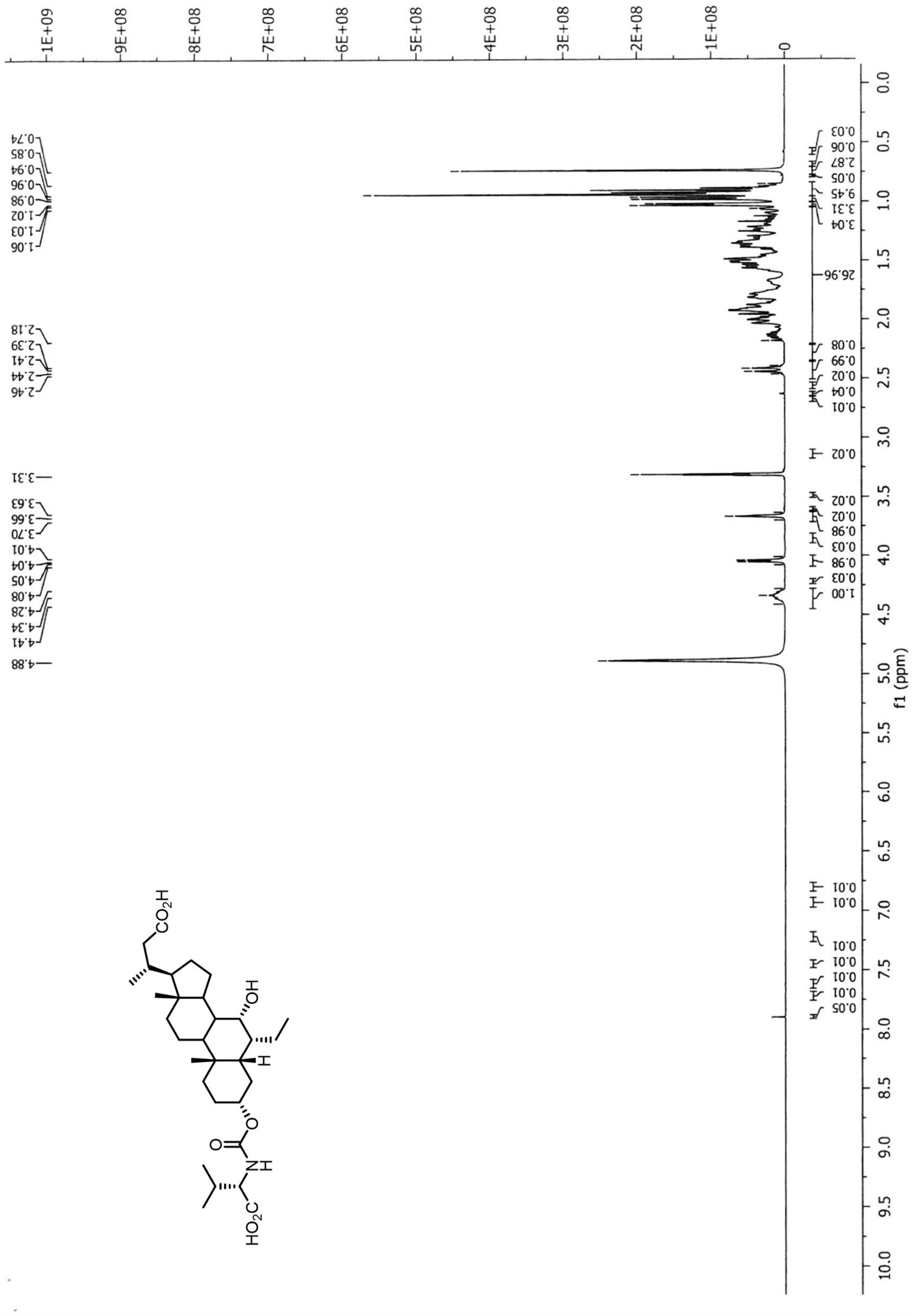

^{13}C-NMR (100 MHz, CD₃OD) of 3α-[((((*S*)-1'-carboxy-2'-methylpropyl)carbamoyl)oxy)]-7α-hydroxy-6α-ethyl-5β-cholan-24-*nor*-23-oic acid (15)

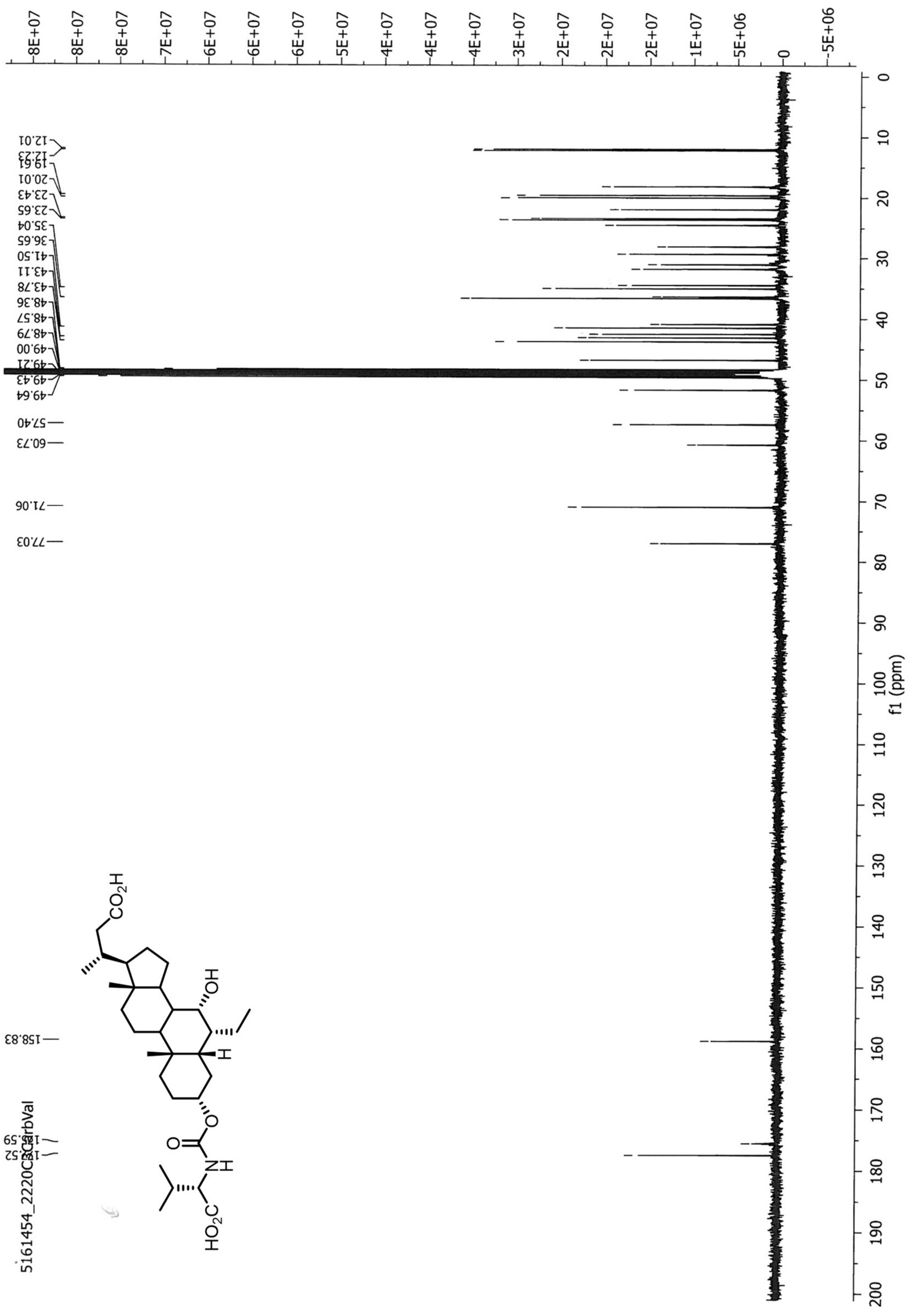

^{1}H-NMR (400 MHz, CD_3OD) of 3α-[(((4'-nitrophenoxy)carbonyl)oxy)]-7α-hydroxy-6α-ethyl-24-*nor*-5β-cholan-23-sulfate (31)

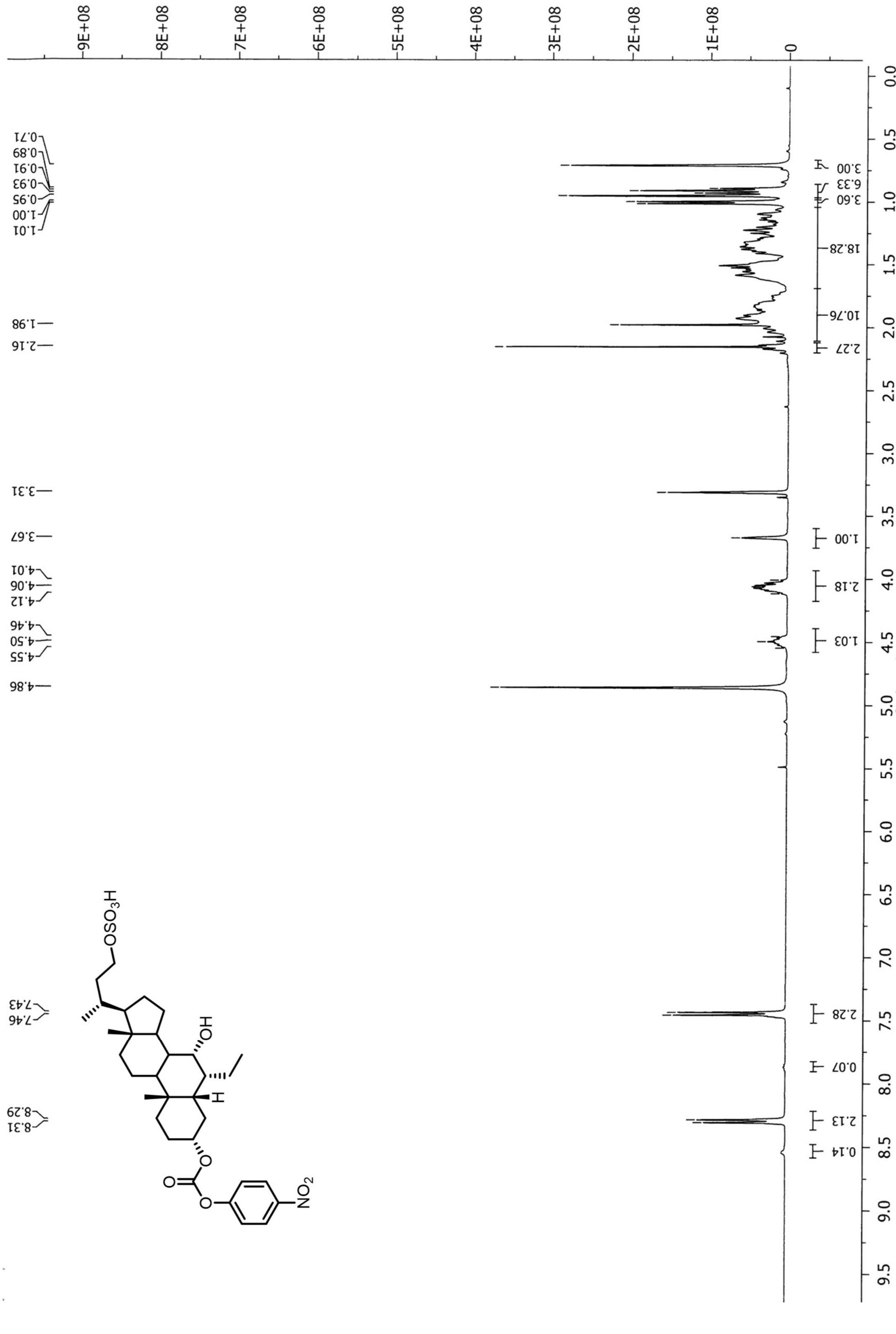

^{1}H-NMR (400 MHz, CD_3OD) of 3α-[((((*S*)-1'-carboxy-2'-methylpropyl)carbamoyl)oxy)]-7α-hydroxy-6α-ethyl-5β-cholan-24-*nor*-23-sulfate triethylammonium salt (16)

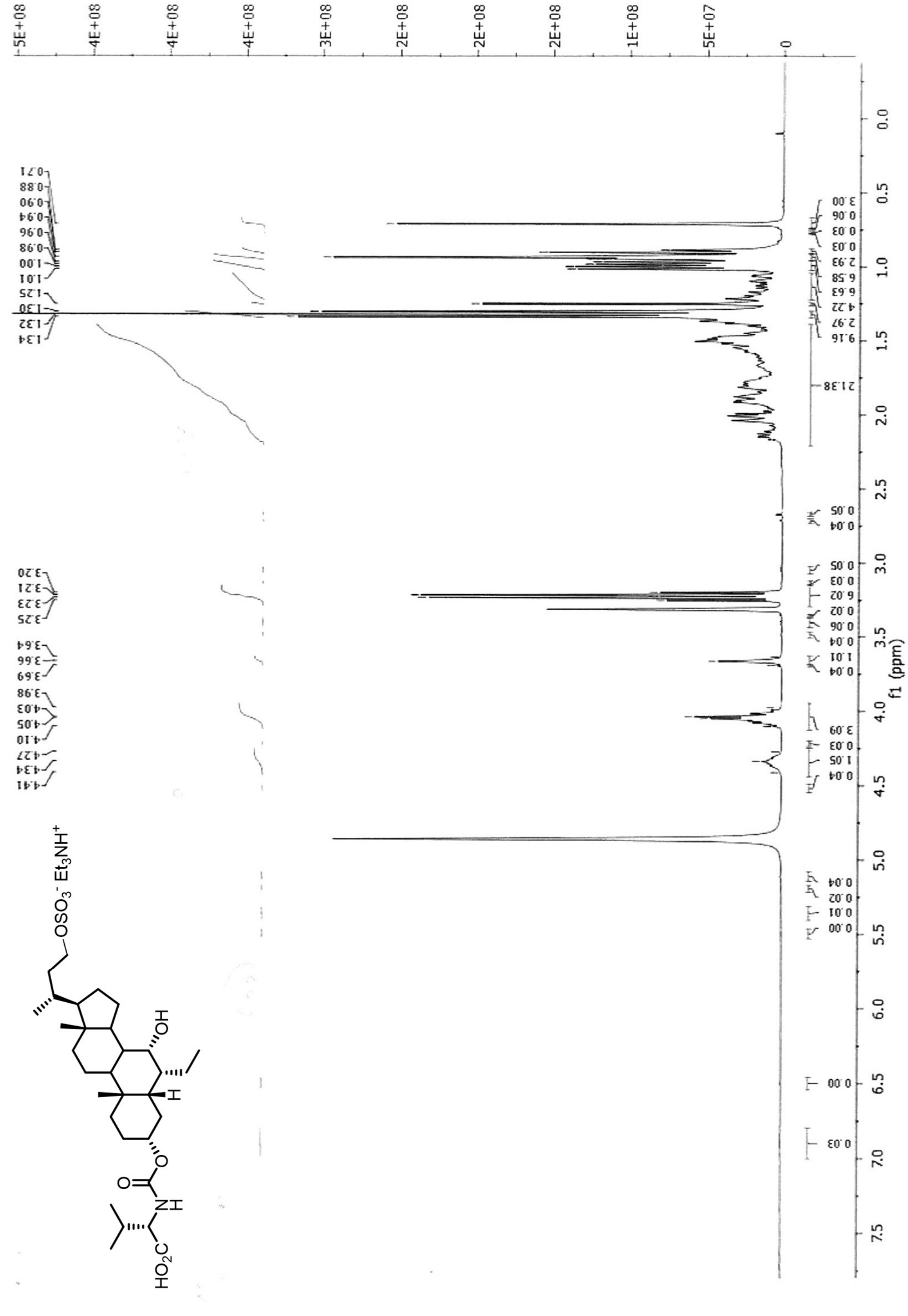

^{13}C-NMR (100 MHz, CD3OD) of 3α-[((((*S*)-1'-carboxy-2'-methylpropyl)carbamoyl)oxy)]-7α-hydroxy-6α-ethyl-5β-cholan-24-*nor*-23-sulfate triethylammonium salt (16)

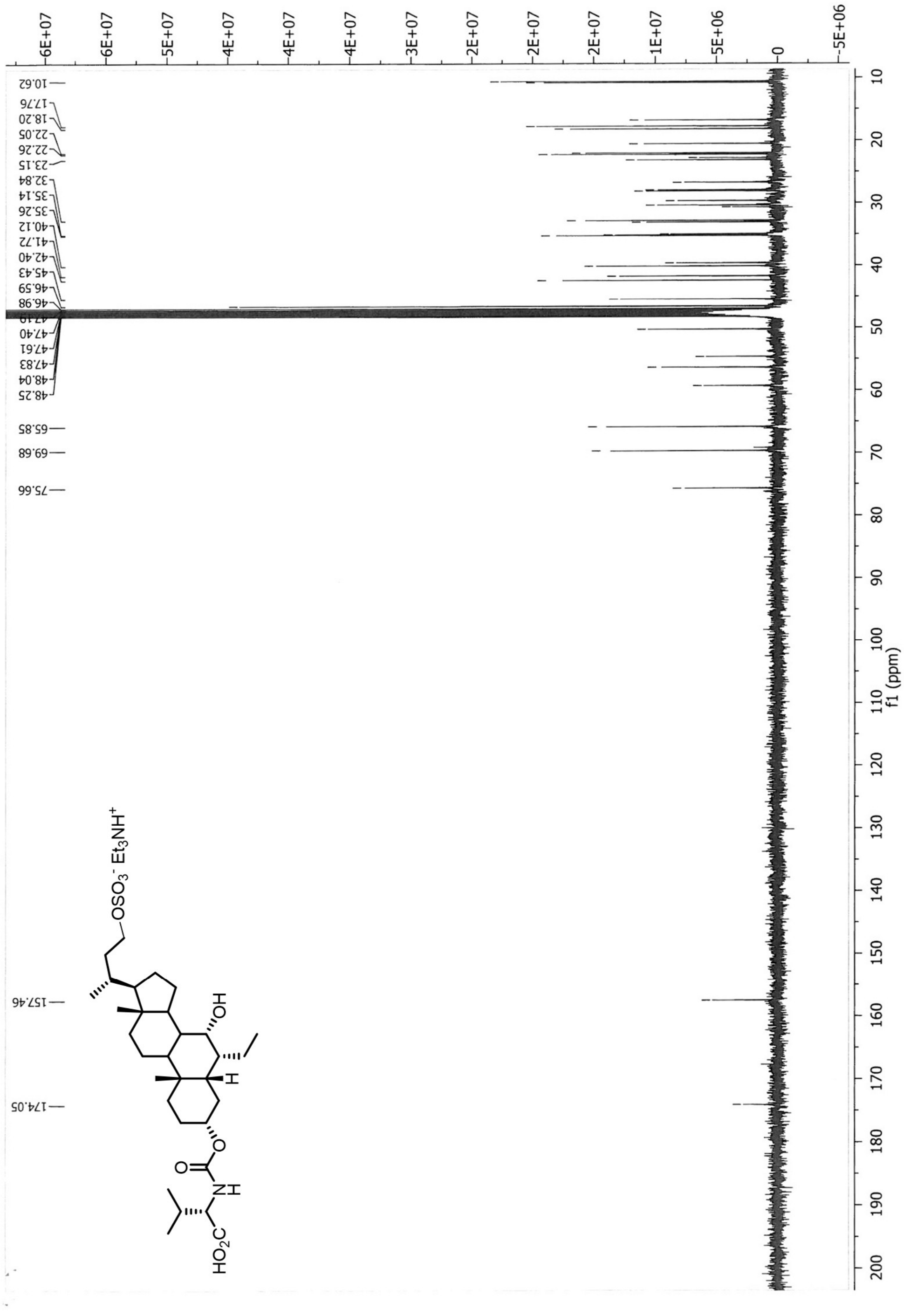

^{1}H-NMR (400 MHz, $CDCl_3$) of 3-[(((4'-nitrophenoxy)carbonyl)oxy)]-7α-hydroxy-6α-ethyl-5β-cholan-22,23-*bisnor* cholan -(1',2',4'-oxadiazol-5-one) (33)

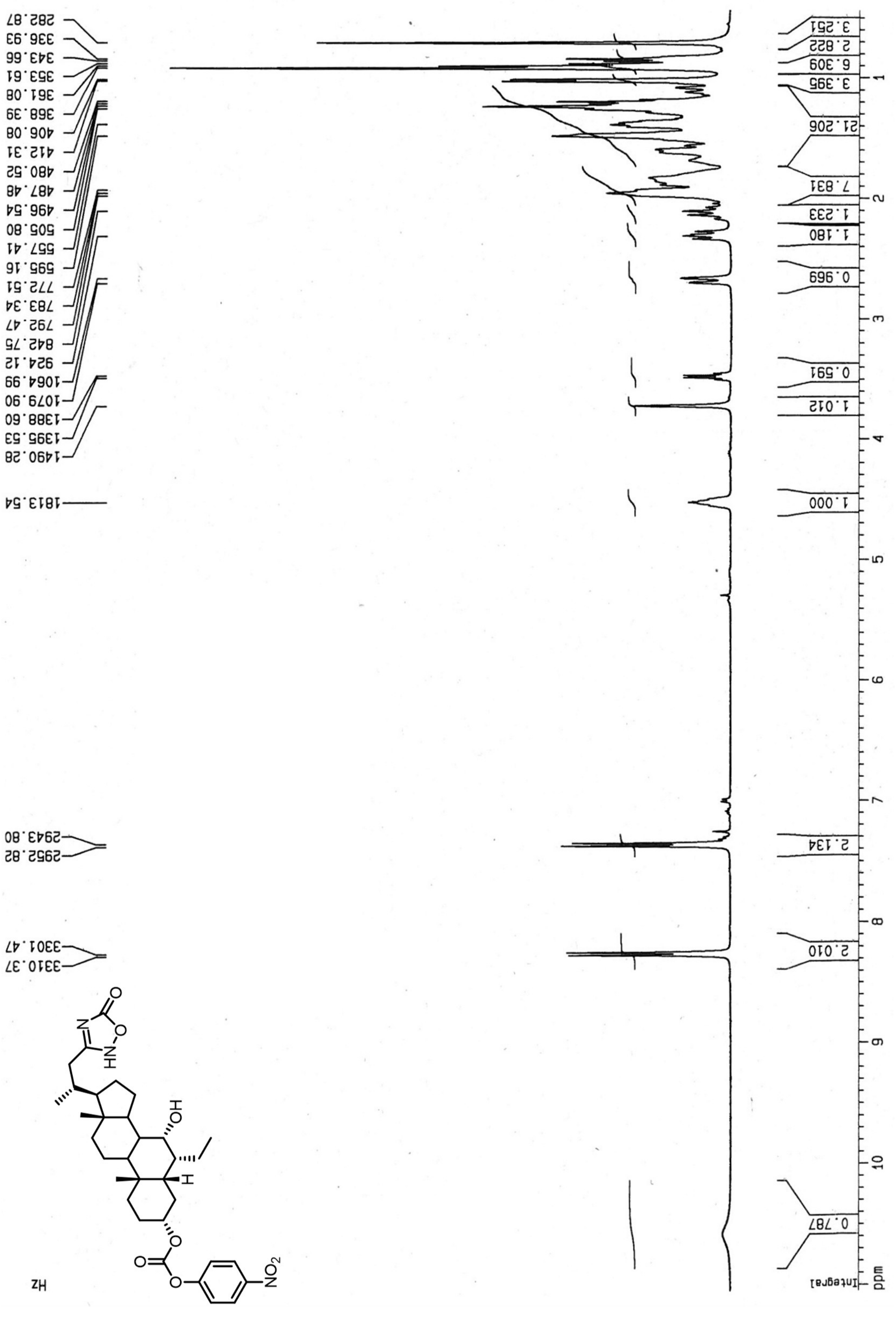

^{1}H-NMR (400 MHz, CD_3OD) of 3α-[((((*S*)-1'-carboxy-2'-methylpropyl)carbamoyl)oxy)]-7α-hydroxy-6α-ethyl-5β-cholan-22,23-*bisnor* cholan -(1',2',4'-oxadiazol-5-one) (17)

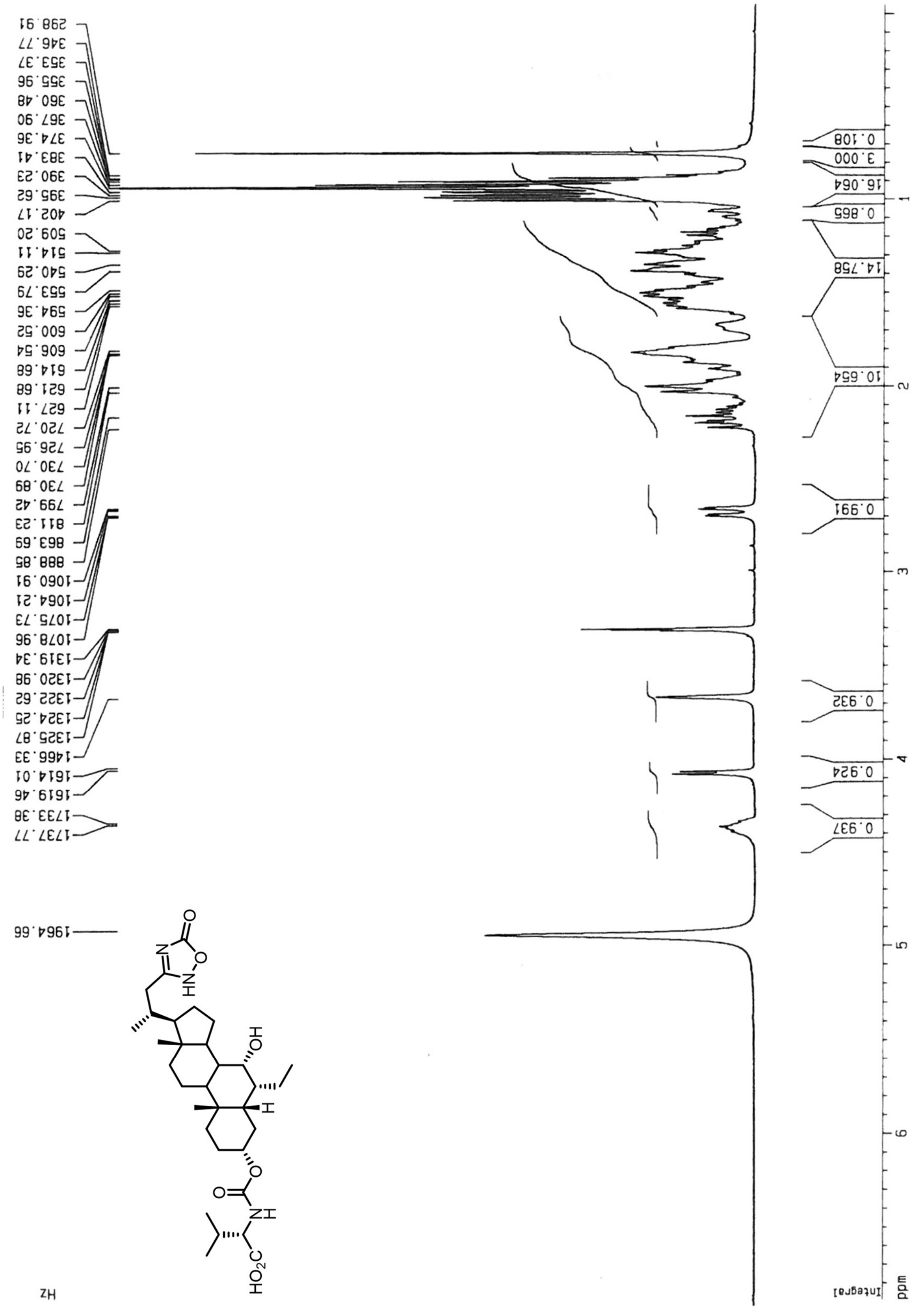

^{13}C-NMR (100 MHz, CD$_3$OD) of 3α-[((((*S*)-1'-carboxy-2'-methylpropyl)carbamoyl)oxy)]-7α-hydroxy-6α-ethyl-5β-cholan-22,23-*bisnor* cholan -(1',2',4'-oxadiazol-5-one) (17)

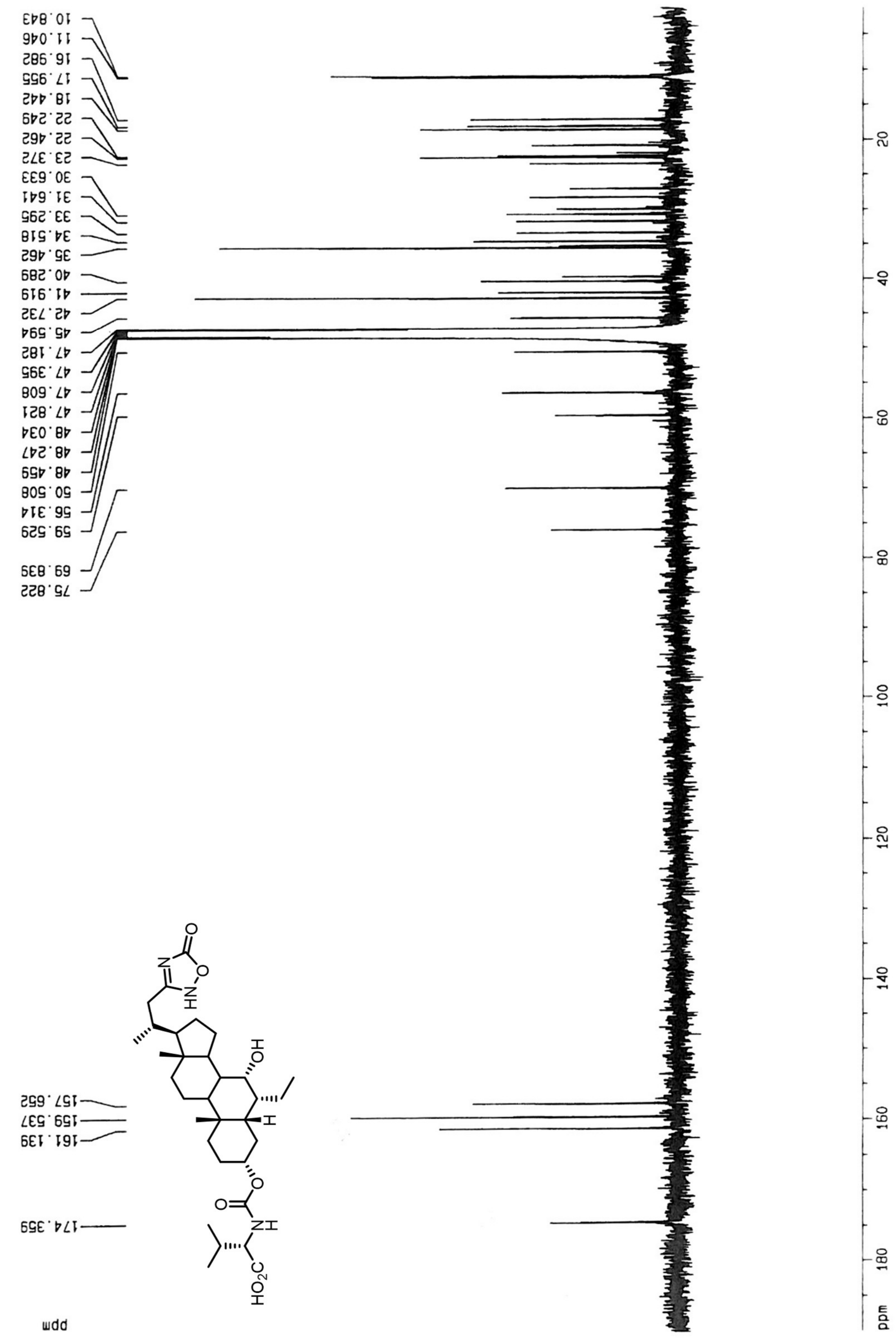

3. Purity data (qNMR in the presence of DMS as the external standard)

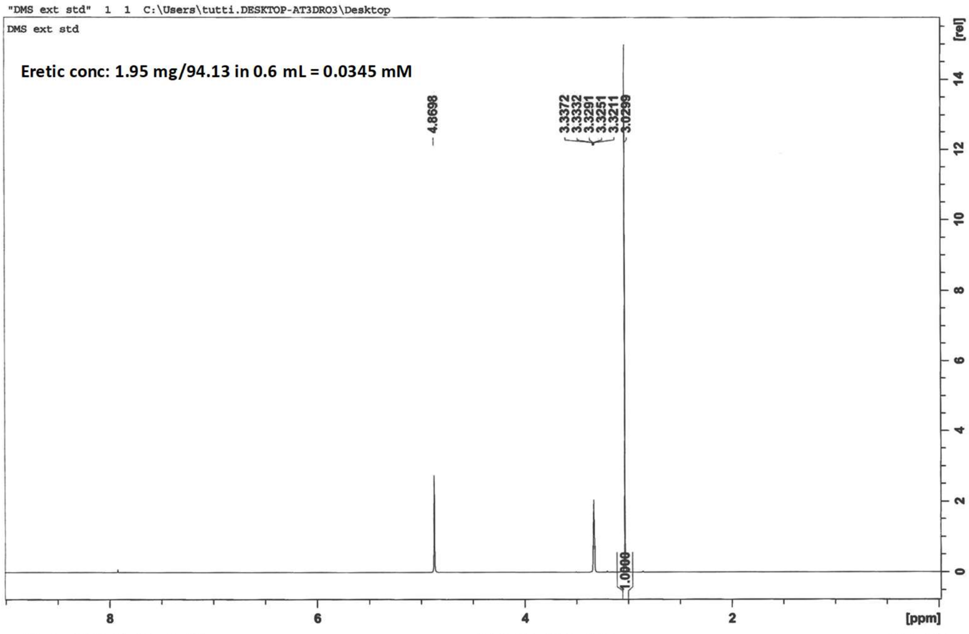

Object	Integral [abs]	Integral [rel]	Peaks	ν(F1) [ppm]	Concentration (Eretic)	Atoms (Eretic)
Integral 1	1298615034368.00	1.0000	1	3.0257	0.0346	6

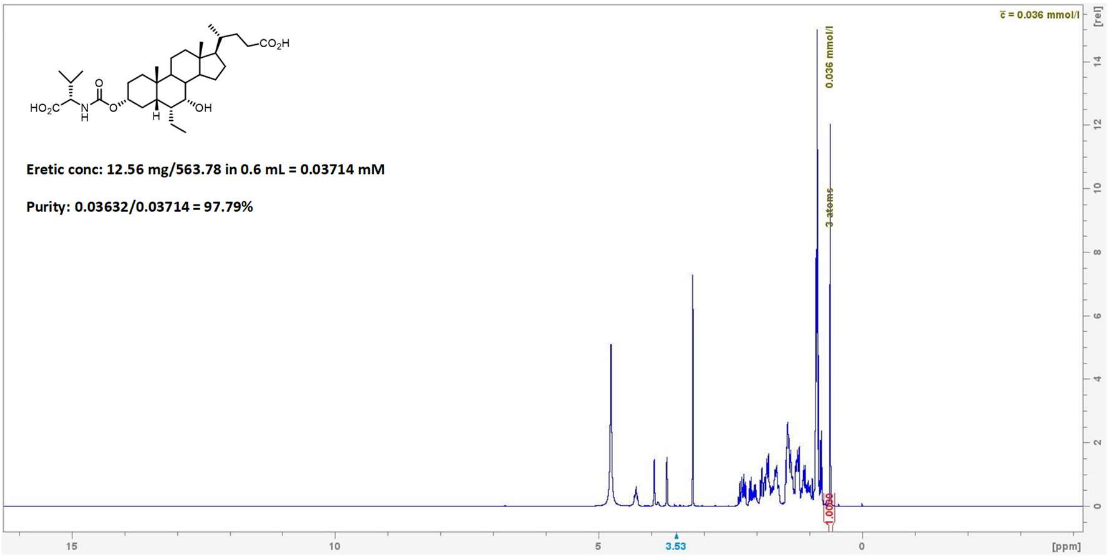

Object	Integral [abs]	Integral [rel]	Peaks	Range (F1) from	Range (F1) to	(F1) [ppm]	Concentration (Eretic)	Atoms (Eretic)	Intensity [abs]
Integral 1	8466723478528.00	1.0000	1	0.633	0.570	0.6015	0.0363161448	3	

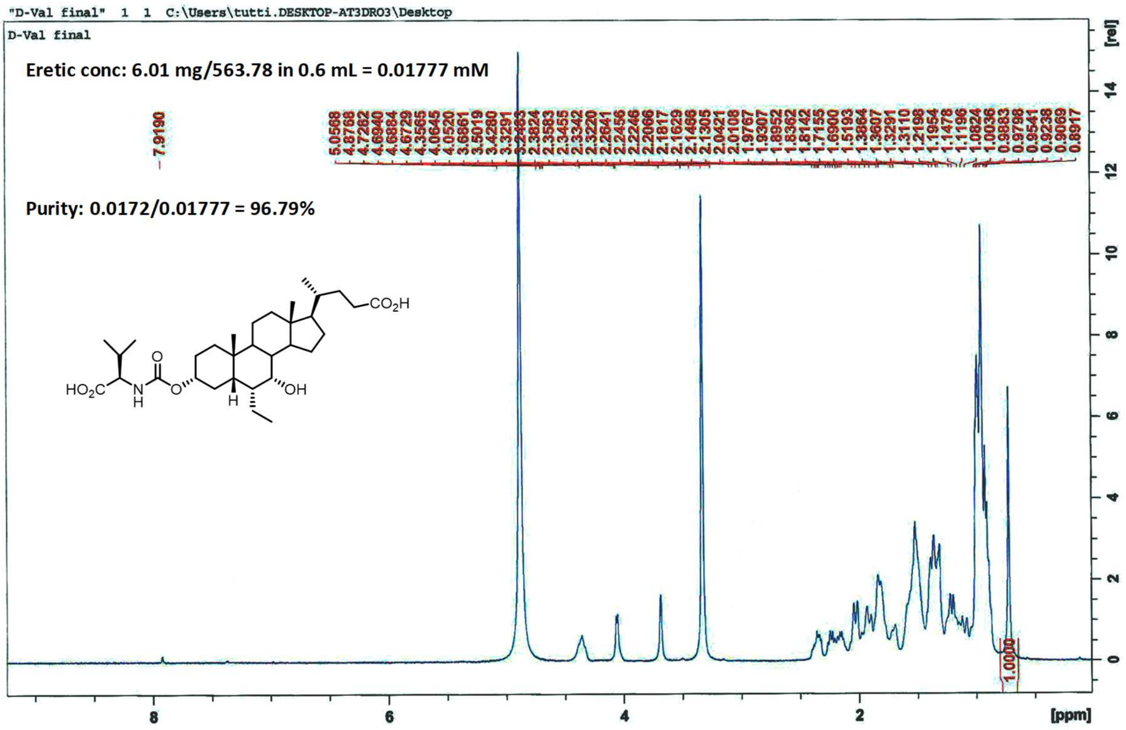

Jun 1, 2023 (4:04:21 PM) Integral list data Page 1/1

Object	Integral [abs]	Integral [rel]	Peaks	v(F1) [ppm]	Concentration (Eretic)	Atoms (Eretic)
Integral 1	323418247840.00	1.0000	1	0.7166	0.0172	3

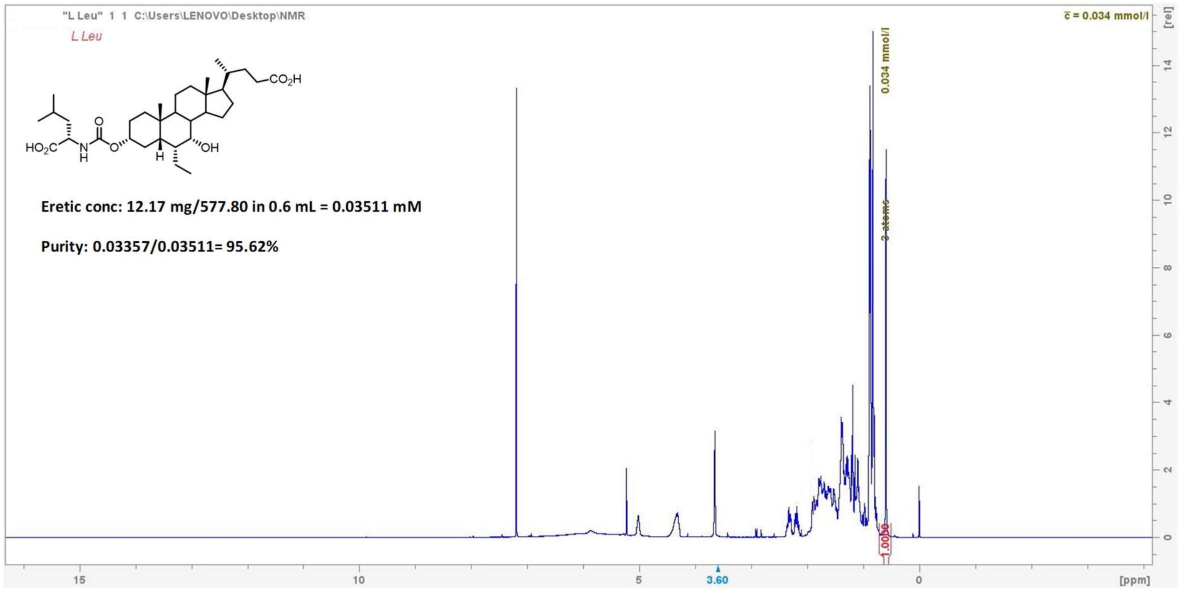

Object	Integral [abs]	Integral [rel]	Peaks	Range (F1) from	Range (F1) to	(F1) [ppm]	Concentration (Eretic)	Atoms (Eretic)	Intensity [abs]
Integral 1	7826000749568.00	1.0000	1	0.640	0.559	0.5994	0.0335680748	3	

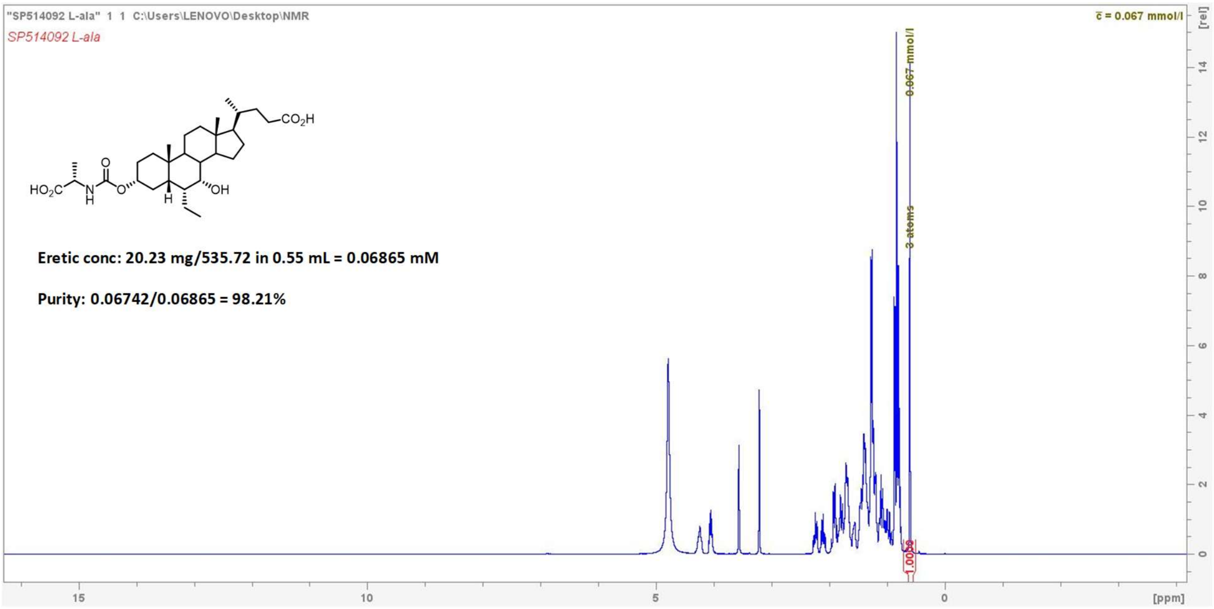

Object	Integral [abs]	Integral [rel]	Peaks	Range (F1) from	Range (F1) to	(F1) [ppm]	Concentration (Eretic)	Atoms (Eretic)	Intensity [abs]
Integral 1	1245019717248.00	1.0000	1	0.644	0.557	0.6000	0.0674201623	3	

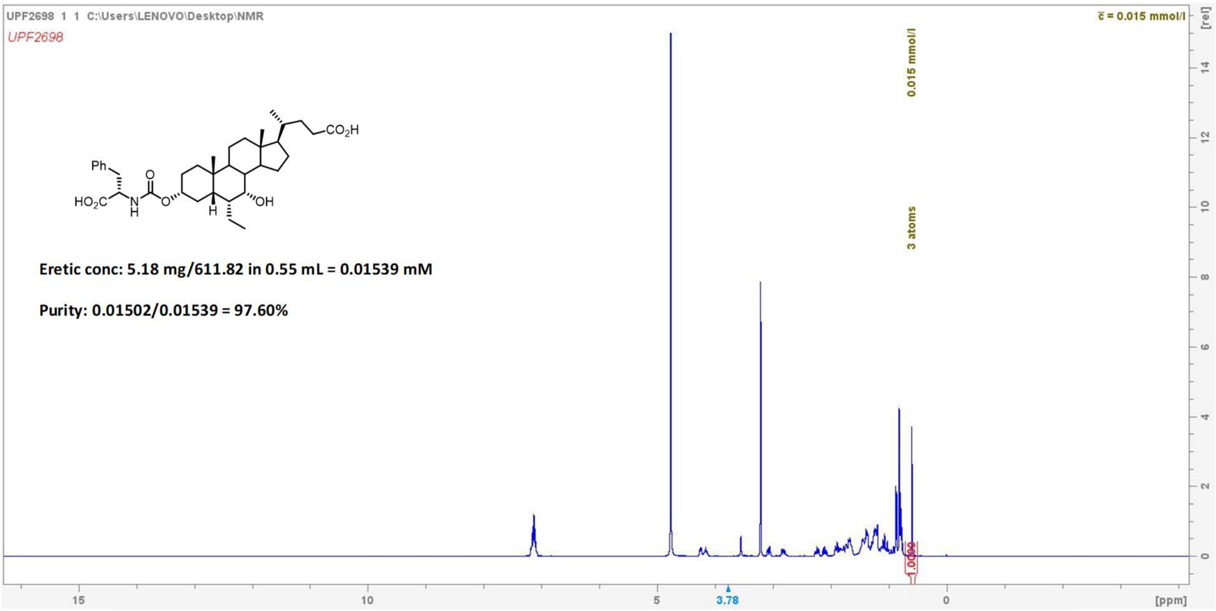

Object	Integral [abs]	Integral [rel]	Peaks	Range (F1) from	Range (F1) to	(F1) [ppm]	Concentration (Eretic)	Atoms (Eretic)	Intensity [abs]
Integral 1	277279806848.00	1.0000	1	0.630	0.555	0.5927	0.0150153547	3	

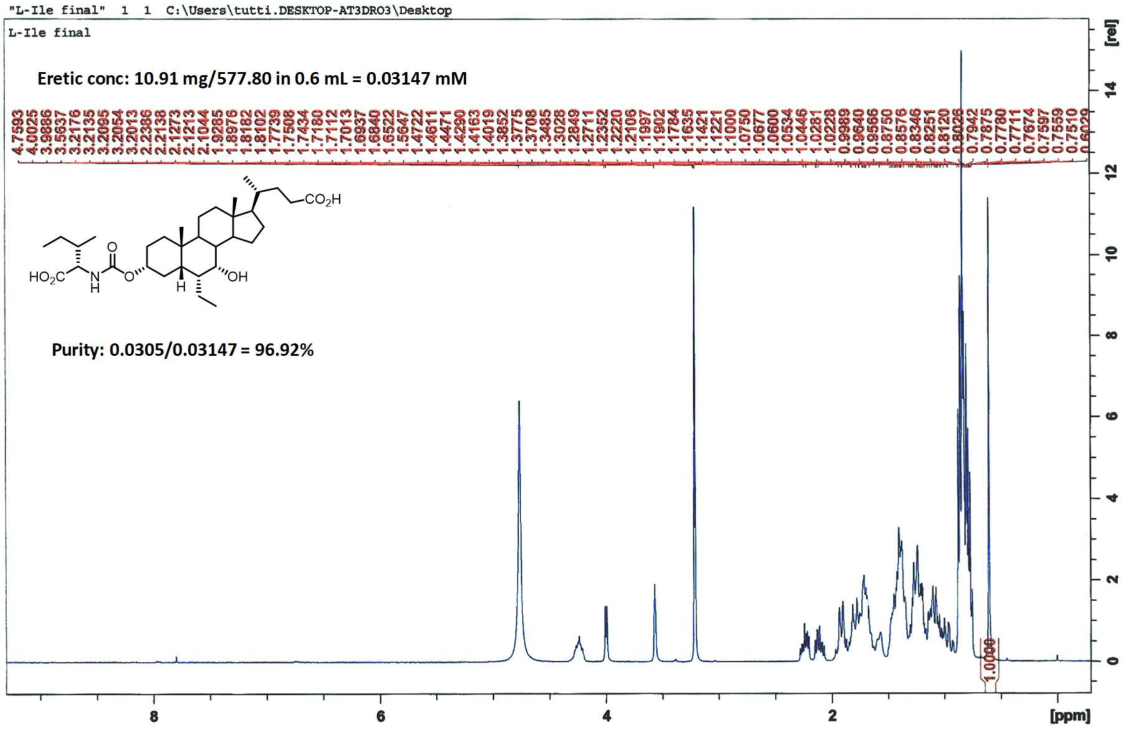

Jun 1, 2023 (4:00:18 PM)

Integral list data

Page 1/1

Object	Integral [abs]	Integral [rel]	Peaks	ν(F1) [ppm]	Concentration (Eretic)	Atoms (Eretic)
Integral 1	572156276672.00	1.0000	1	0.5939	0.0305	3

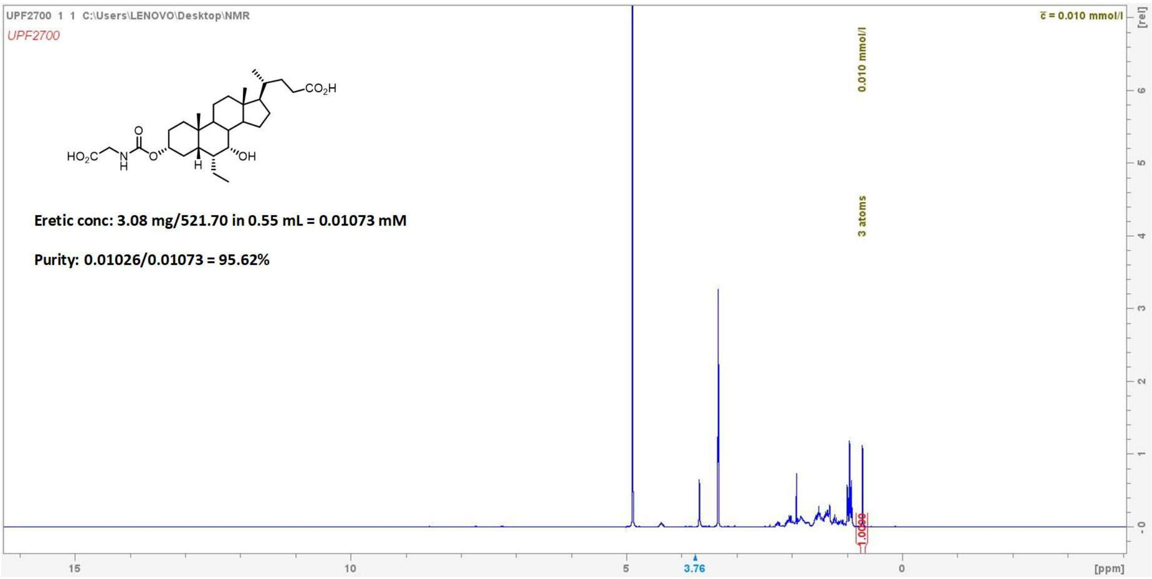

Object	Integral [abs]	Integral [rel]	Peaks	Range (F1) from	Range (F1) to	(F1) [ppm]	Concentration (Eretic)	Atoms (Eretic)	Intensity [abs]
Integral 1	189400869632.00	1.0000	1	0.766	0.679	0.7227	0.0102564310	3	

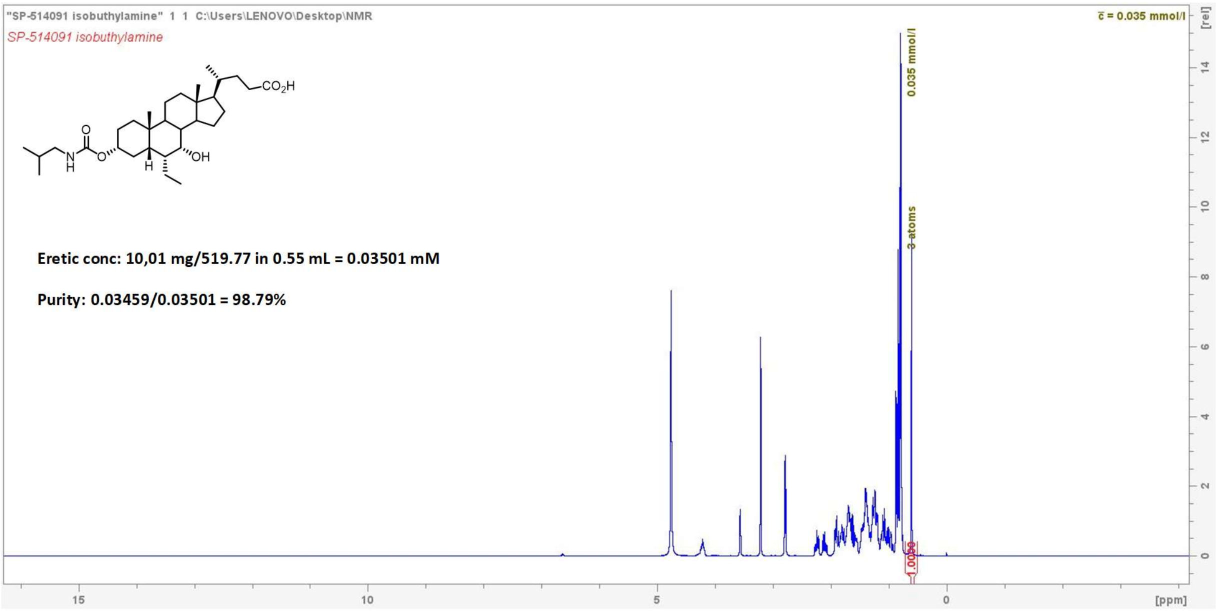

Object	Integral [abs]	Integral [rel]	Peaks	Range (F1) from	Range (F1) to	(F1) [ppm]	Concentration (Eretic)	Atoms (Eretic)	Intensity [abs]
Integral 1	638819439872.00	1.0000	1	0.627	0.576	0.6013	0.0345934498	3	

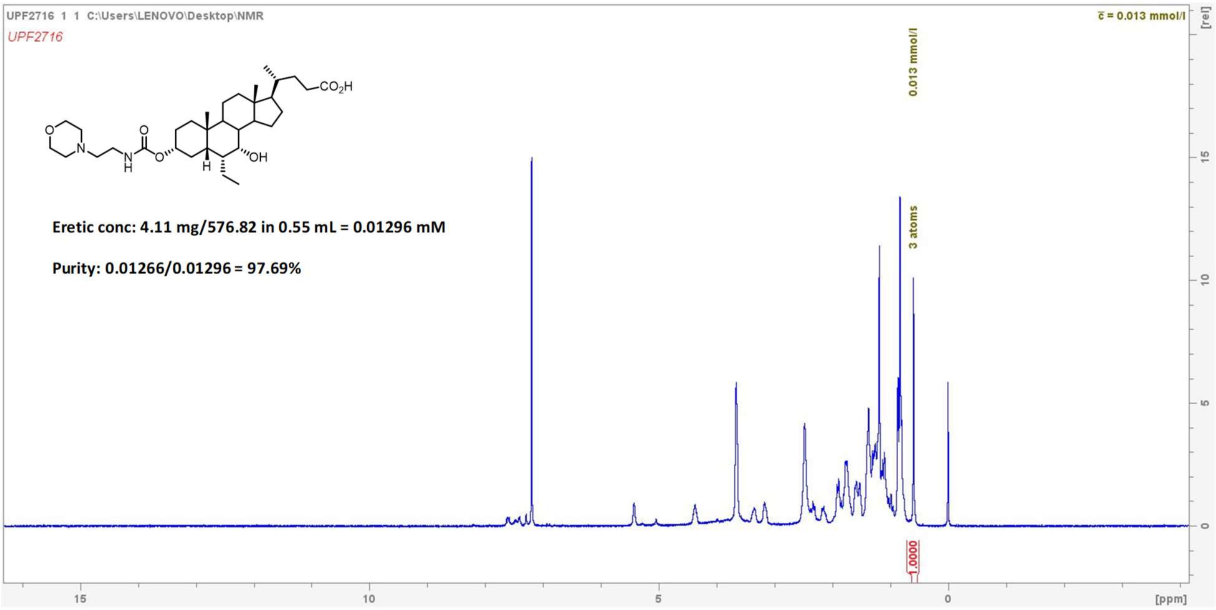

Object	Integral [abs]	Integral [rel]	Peaks	Range (F1) from	Range (F1) to	(F1) [ppm]	Concentration (Eretic)	Atoms (Eretic)	Intensity [abs]
Integral 1	233806824560.00	1.0000	1	0.636	0.546	0.5909	0.0126610756	3	

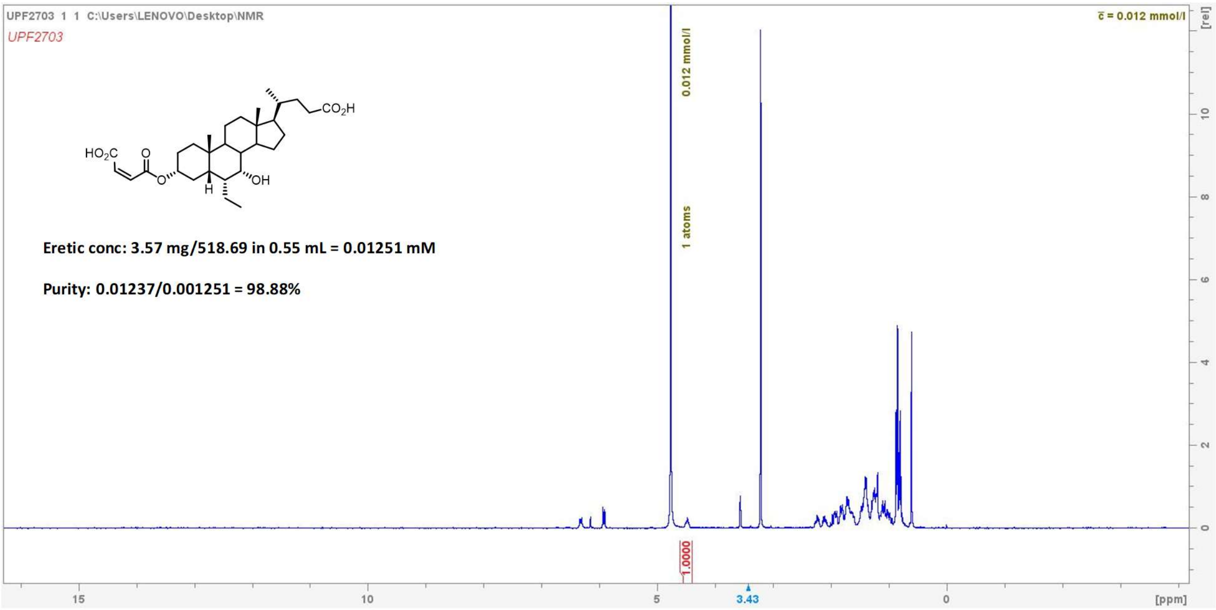

Object	Integral [abs]	Integral [rel]	Peaks	Range (F1) from	Range (F1) to	(F1) [ppm]	Concentration (Eretic)	Atoms (Eretic)	Intensity [abs]
Integral 1	76139385536.00	1.0000	4	4.554	4.403	4.4786	0.0123694269	1	

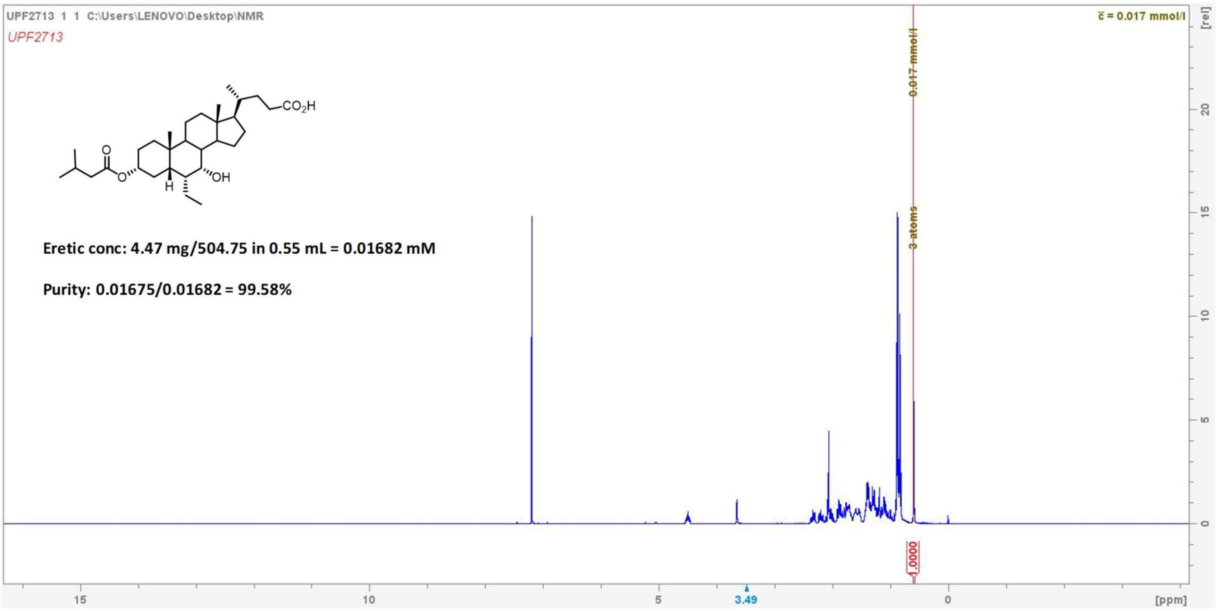

Object	Integral [abs]	Integral [rel]	Peaks	Range (F1) from	Range (F1) to	(F1) [ppm]	Concentration (Eretic)	Atoms (Eretic)	Intensity [abs]
Integral 1	309237899136.00	1.0000	1	0.613	0.582	0.5977	0.0167459663	3	

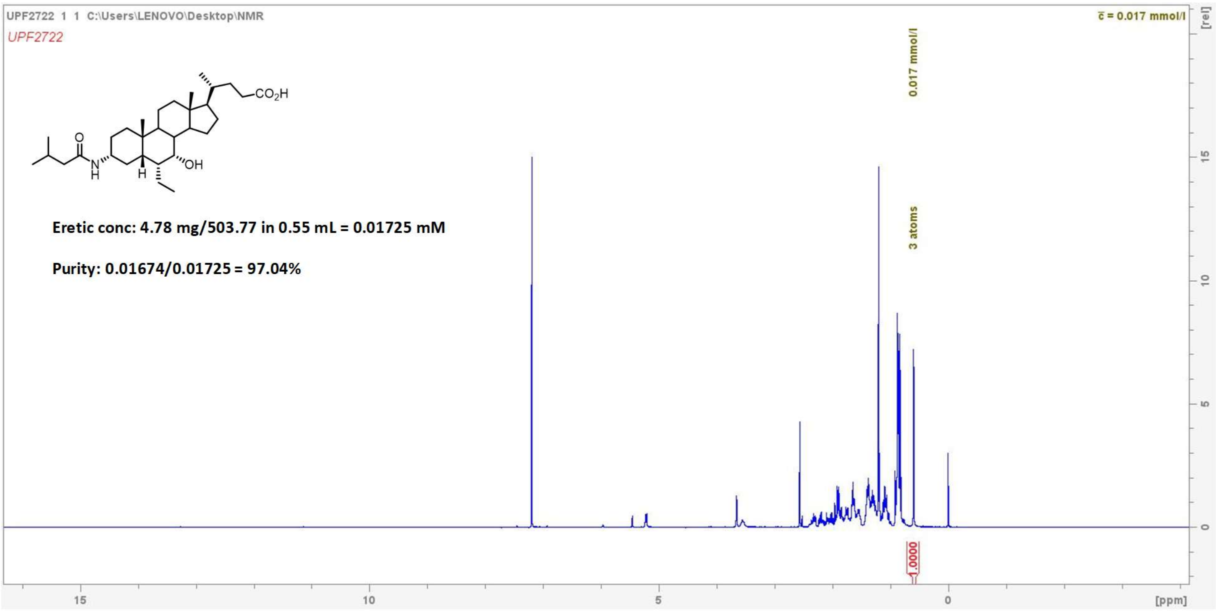

Object	Integral [abs]	Integral [rel]	Peaks	Range (F1) from	Range (F1) to	(F1) [ppm]	Concentration (Eretic)	Atoms (Eretic)	Intensity [abs]
Integral 1	309113847360.00	1.0000	1	0.619	0.568	0.5934	0.0167390576	3	

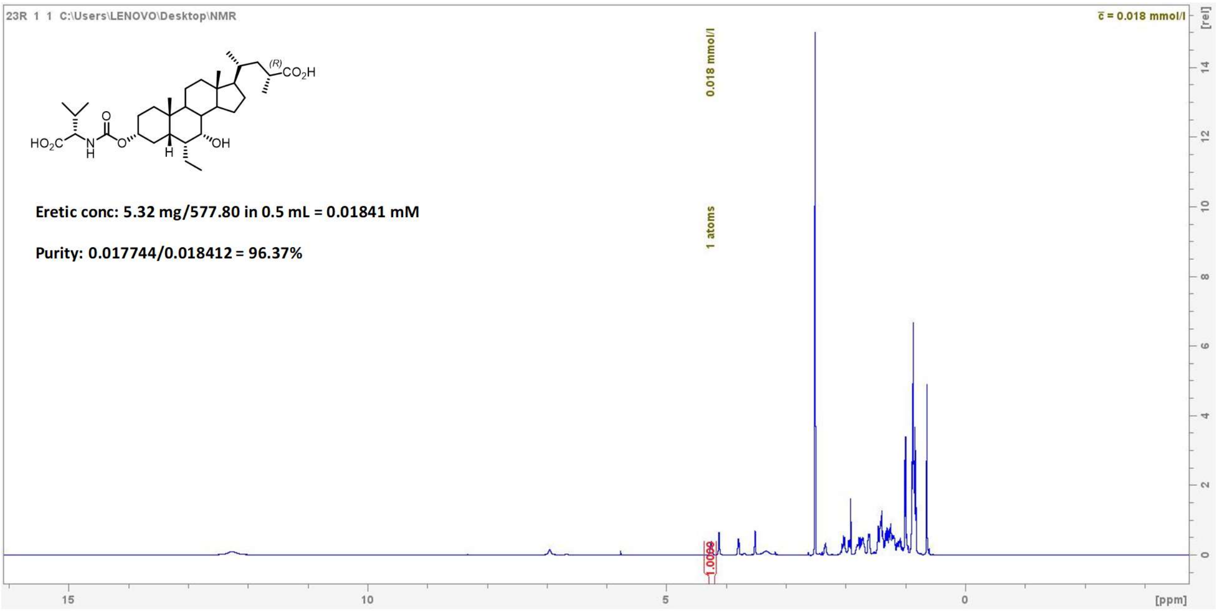

Object	Integral [abs]	Integral [rel]	Peaks	Range (F1) from	Range (F1) to	(F1) [ppm]	Concentration (Eretic)	Atoms (Eretic)	Intensity [abs]
Integral 1	66050555008.00	1.0000	5	4.299	4.194	4.2465	0.0177436941	1	

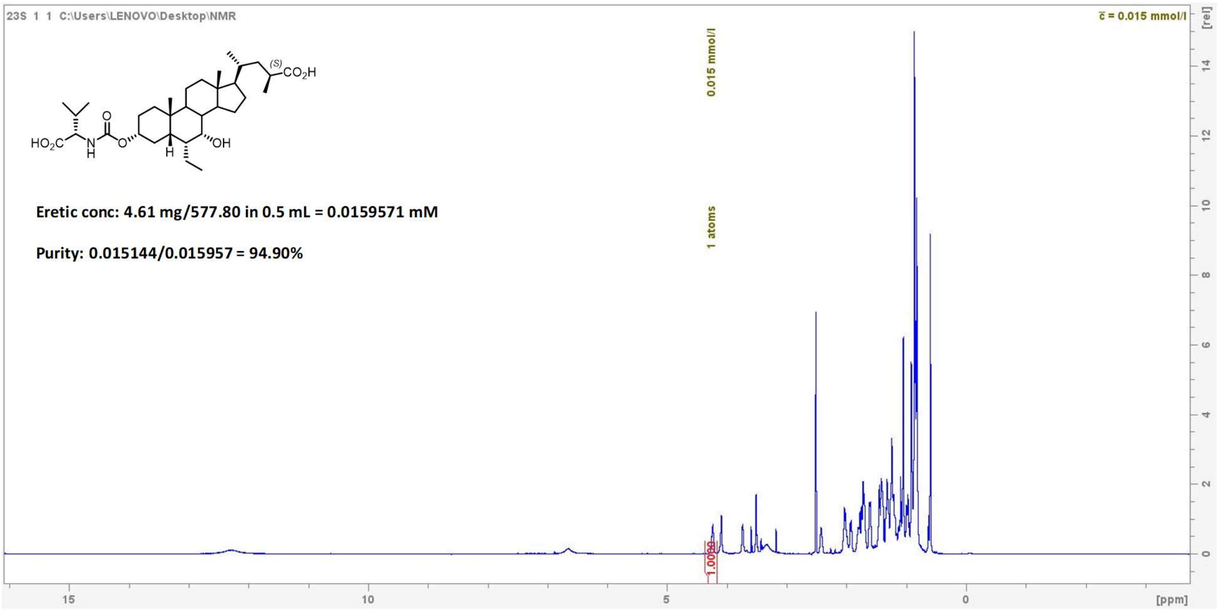

Object	Integral [abs]	Integral [rel]	Peaks	Range (F1) from	Range (F1) to	(F1) [ppm]	Concentration (Eretic)	Atoms (Eretic)	Intensity [abs]
Integral 1	5637321091 2.00	1.0000	3	4.315	4.172	4.2435	0.0151441231	1	

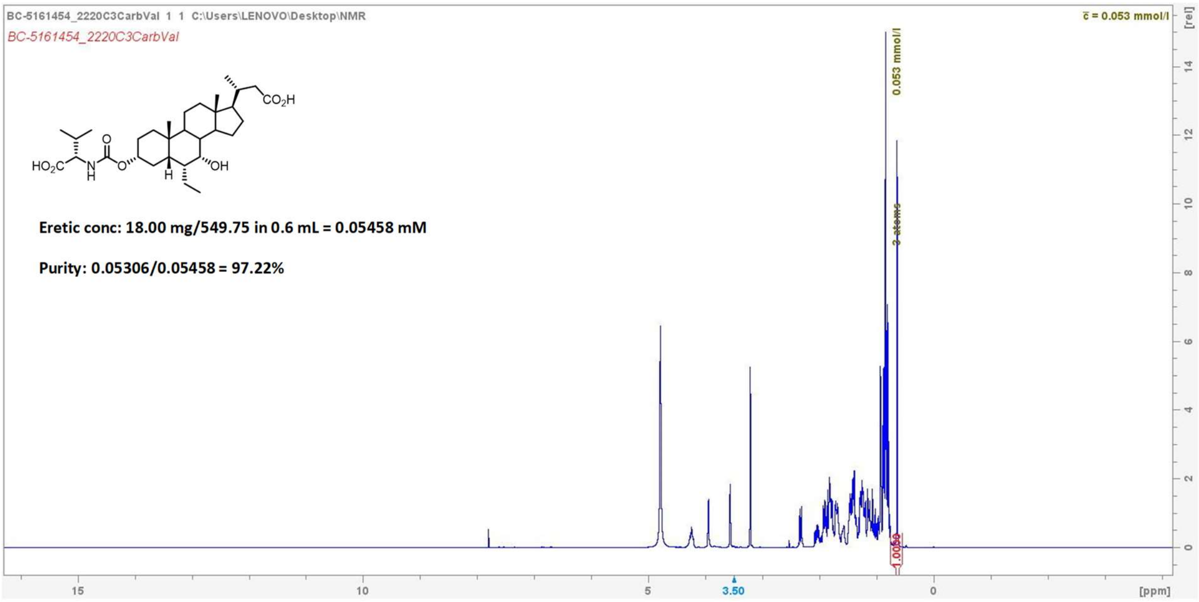

Object	Integral [abs]	Integral [rel]	Peaks	Range (F1) from	Range (F1) to	(F1) [ppm]	Concentration (Eretic)	Atoms (Eretic)	Intensity [abs]
Integral 1	1108380477 9520.00	1.0000	1	0.663	0.606	0.6347	0.0530578170	3	

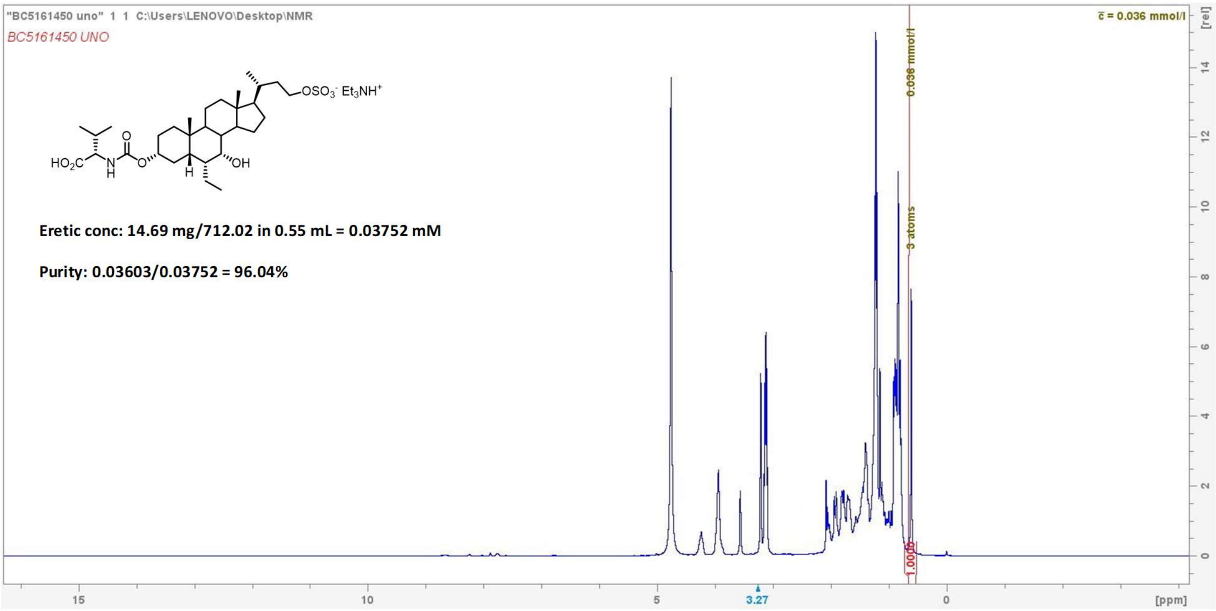

Object	Integral [abs]	Integral [rel]	Peaks	Range (F1) from	Range (F1) to	(F1) [ppm]	Concentration (Eretic)	Atoms (Eretic)	Intensity [abs]
Integral 1	7526421173760.00	1.0000	1	0.670	0.538	0.6038	0.0360283976	3	

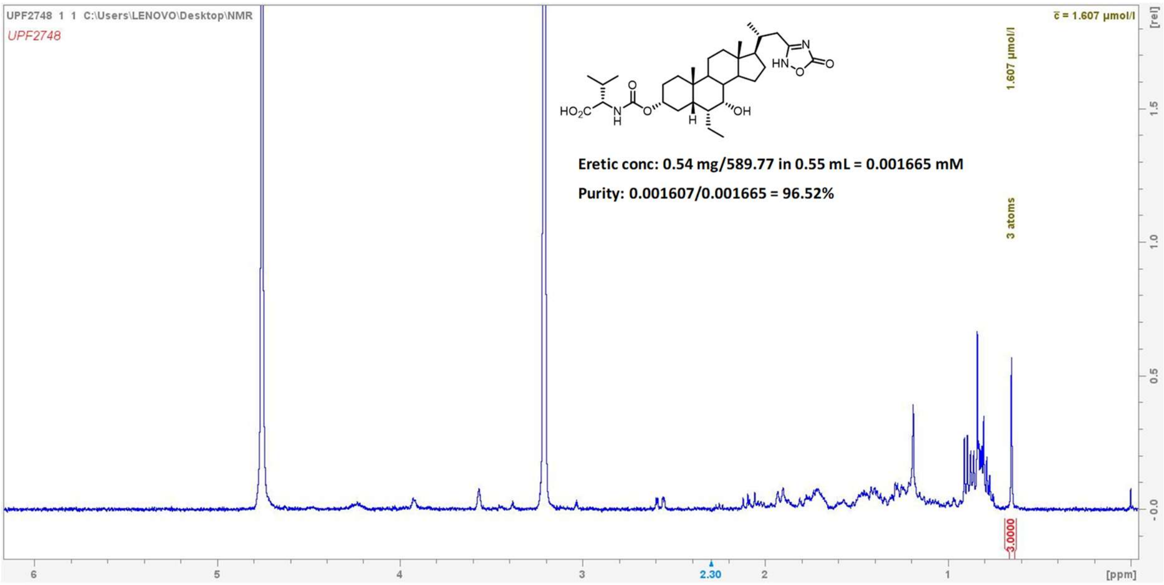

Object	Integral [abs]	Integral [rel]	Peaks	Range (F1) from	Range (F1) to	(F1) [ppm]	Concentration (Eretic)	Atoms (Eretic)	Intensity [abs]
Integral 1	28781039232.00	3.0000	1	0.667	0.635	0.6513	0.0016071416	3	

4. HRMS data

Target Screening Report

Compound Chromatograms (overlaid)

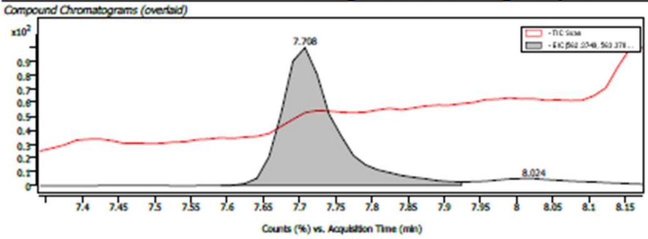

Structure

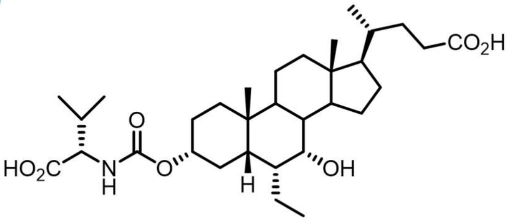

Compound Spectra (overlaid)

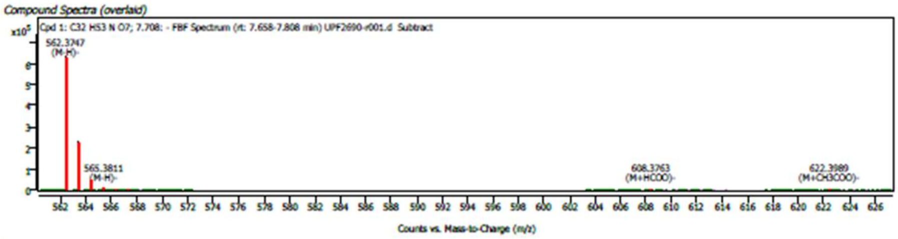

Compound ID Table

Name	Formula	Species	RT	RT Diff	Mass	CAS	ID Source	Score	Score (Lib)	Score (Tgt)
	C32 H53 N O7	(M-H)- (M+HCOO)- (M+CH3COO)-	7.708		563.3818		FBF	99.02		99.02

Cpd. 2: C32 H53 N O7

Name	Formula	RT	RI	Mass	Diff (Tgt, ppm)	CAS	ID Source	Score	Algorithm
	C32 H53 N O7	8.024		563.3806	-2.90		FBF	93.07	FBF

Formula	Height	Area
C32 H53 N O7	90012.1822770508	1020455.77242709

Species	m/z	Score (Tgt)	Score (Lib)	Score (DB)	Score (MFG)	Score (RT)
(M-H)-	562.3735	93.07				

Compound Chromatograms (overlaid)

Structure

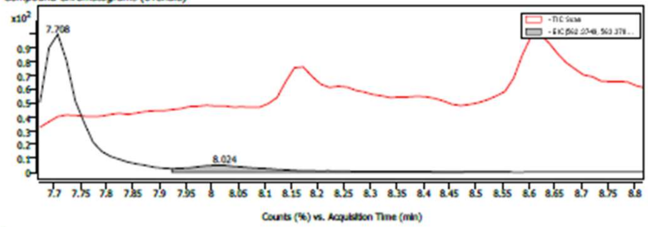

MassHunter Qualitative Analysis | Page 2 of 3 | Generated at 11:17 AM on 11/21/2025

Target Screening Report

Compound Chromatograms (overlaid)

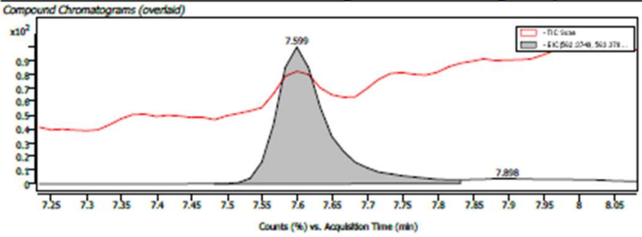

Structure

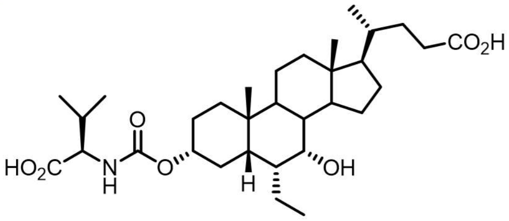

Compound Spectra (overlaid)

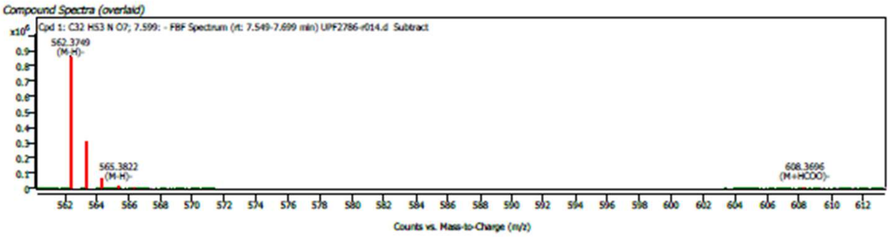

Compound ID Table

Name	Formula	Species	RT	RT Diff	Mass	CAS	ID Source	Score	Score (Lib)	Score (Tgt)
	C32 H53 N O7	(M-H)- (M+HCOO)-	7.599		563.3820		FBF	99.42		99.42

Cpd. 2: C32 H53 N O7

Name	Formula	RT	RI	Mass	Diff (Tgt, ppm)	CAS	ID Source	Score	Algorithm
	C32 H53 N O7	7.898		563.3807	-2.65		FBF	94.79	FBF

Formula	Height	Area
C32 H53 N O7	94129.3642737057	1389310.24458102

Species	m/z	Score (Tgt)	Score (Lib)	Score (DB)	Score (MFG)	Score (RT)
(M-H)-	562.3736	94.79				

Compound Chromatograms (overlaid)

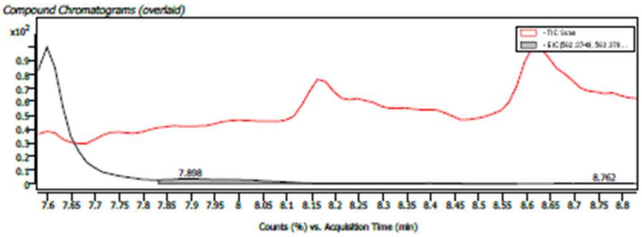

Structure

Target Screening Report

Agilent

Species	m/z	Score (Tgt)	Score (Lib)	Score (DB)	Score (MFG)	Score (RT)
(M-H)- (M+CH3COO)-	576.3906 636.4016	99.54				

Compound Chromatograms (overlaid)

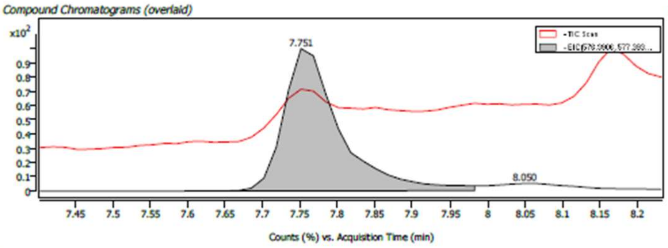

Structure

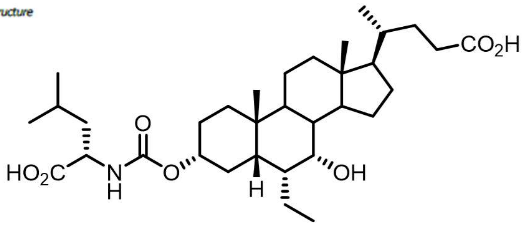

Compound Spectra (overlaid)

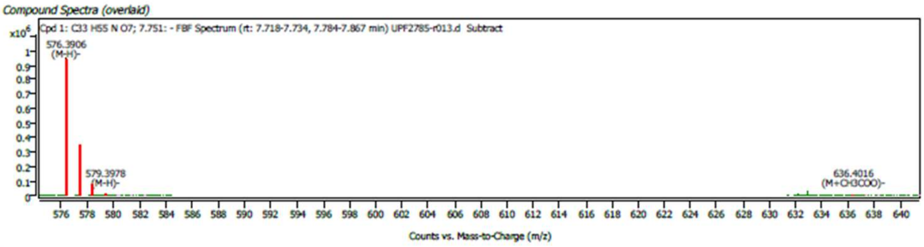

Compound ID Table

Name	Formula	Species	RT	RT Diff	Mass	CAS	ID Source	Score	Score (Lib)	Score (Tgt)
	C33 H55 N O7	(M-H)- (M+CH3COO)-	7.751		577.3977		FBF	99.54		99.54

Cpd. 2: C33 H55 N O7

Name	Formula	RT	RI	Mass	Diff (Tgt, ppm)	CAS	ID Source	Score	Algorithm
	C33 H55 N O7	8.050		577.3965	-2.35		FBF	95.67	FBF

Formula	Height	Area
C33 H55 N O7	166724.872832314	1502618.25822486

Species	m/z	Score (Tgt)	Score (Lib)	Score (DB)	Score (MFG)	Score (RT)
(M-H)- (M+HCOO)-	576.3893 622.4091	95.67				

Compound Chromatograms (overlaid)

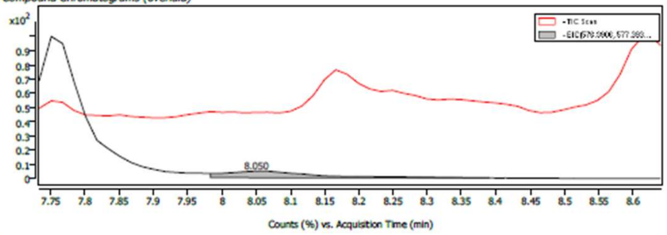

Structure

Target Screening Report

Compound Chromatograms (overlaid)

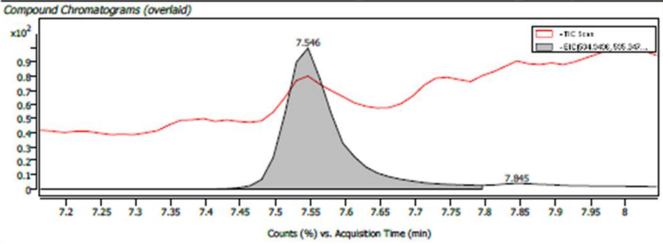

Structure

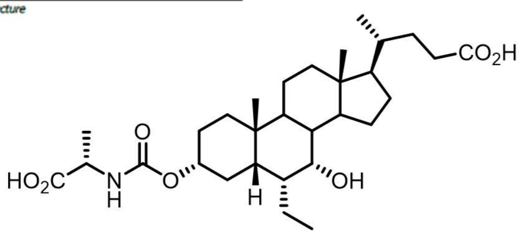

Compound Spectra (overlaid)

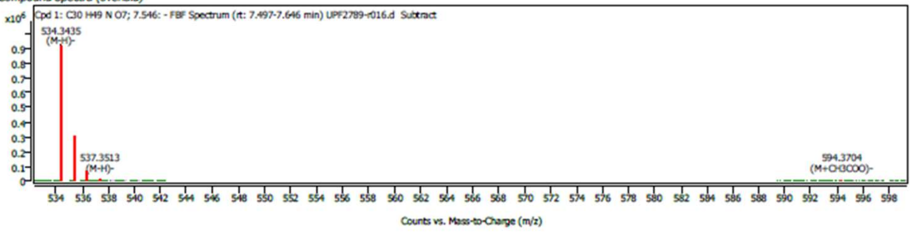

Compound ID Table

Name	Formula	Species	RT	RT Diff	Mass	CAS	ID Source	Score	Score (Lib)	Score (Tgt)
	C30 H49 N O7	(M-H)- (M+CH3COO)-	7.546		535.3507		FBF	99.53		99.53

Cpd. 2: C30 H49 N O7

Name	Formula	RT	RI	Mass	Diff (Tgt, ppm)	CAS	ID Source	Score	Algorithm
	C30 H49 N O7	7.845		535.3491	-3.33		FBF	88.87	FBF

Formula	Height	Area
C30 H49 N O7	102687.026178935	1317710.06821766

Species	m/z	Score (Tgt)	Score (Lib)	Score (DB)	Score (MFG)	Score (RT)
(M-H)-	534.3423	88.87				

Compound Chromatograms (overlaid)

Structure

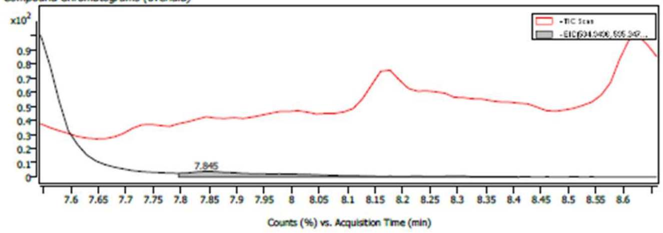

Target Screening Report

Species	m/z	Score (Tgt)	Score (Lib)	Score (DB)	Score (MFG)	Score (RT)
(M-H)- (M+HCOO)-	610.3748 656.3823	98.80				

Compound Chromatograms (overlaid)

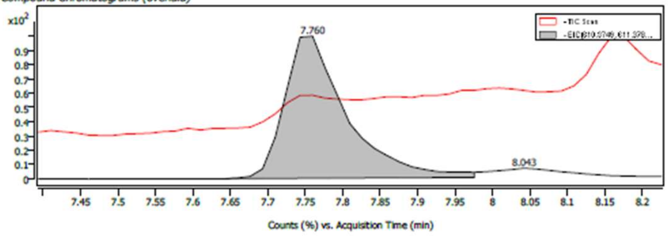

Structure

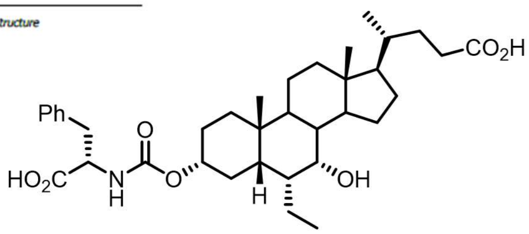

Compound Spectra (overlaid)

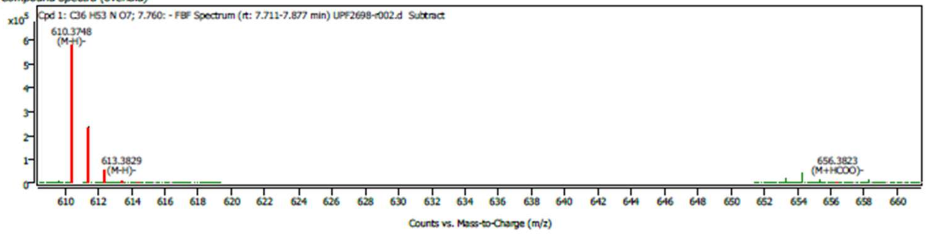

Compound ID Table

Name	Formula	Species	RT	RT Diff	Mass	CAS	ID Source	Score	Score (Lib)	Score (Tgt)
	C36 H53 N O7	(M-H)- (M+HCOO)-	7.760		611.3818		FBF	98.80		98.80

Cpd. 2: C36 H53 N O7

Name	Formula	RT	RI	Mass	Diff (Tgt, ppm)	CAS	ID Source	Score	Algorithm
	C36 H53 N O7	8.043		611.3808	-2.37		FBF	95.47	FBF

Formula	Height	Area
C36 H53 N O7	91072.7453542283	622362.802542668

Species	m/z	Score (Tgt)	Score (Lib)	Score (DB)	Score (MFG)	Score (RT)
(M-H)-	610.3735	95.47				

Compound Chromatograms (overlaid)

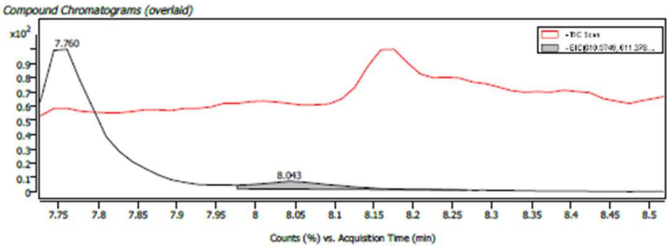

Structure

Target Screening Report

Species	m/z	Score (Tgt)	Score (Lib)	Score (DB)	Score (MFG)	Score (RT)
(M-H)- (M+HCOO)- (M+CH3COO)-	576.3905 622.3824 636.4104	99.34				

Compound Chromatograms (overlaid)

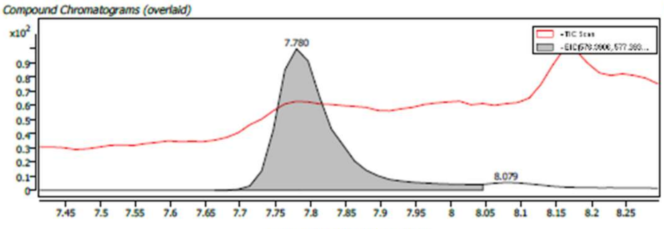

Structure

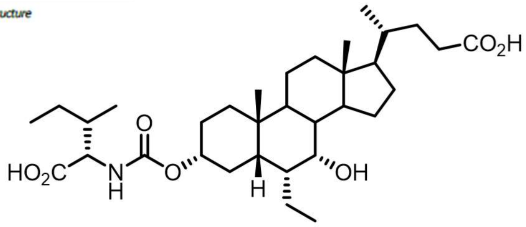

Compound Spectra (overlaid)

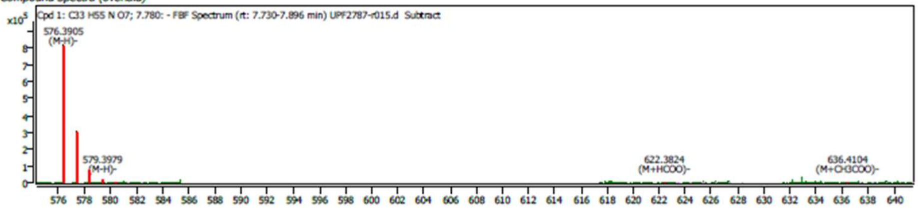

Compound ID Table

Name	Formula	Species	RT	RT Diff	Mass	CAS	ID Source	Score	Score (Lib)	Score (Tgt)
	C33 H55 N O7	(M-H)- (M+HCOO)- (M+CH3COO)-	7.780		577.3976		FBF	99.34		99.34

Cpd. 2: C33 H55 N O7

Name	Formula	RT	RI	Mass	Diff (Tgt, ppm)	CAS	ID Source	Score	Algorithm
	C33 H55 N O7	8.079		577.3963	-2.70		FBF	92.97	FBF

Formula	Height	Area
C33 H55 N O7	123516.992640166	1057704.13325412

Species	m/z	Score (Tgt)	Score (Lib)	Score (DB)	Score (MFG)	Score (RT)
(M-H)- (M+CH3COO)-	576.3891 636.4171	92.97				

Compound Chromatograms (overlaid)

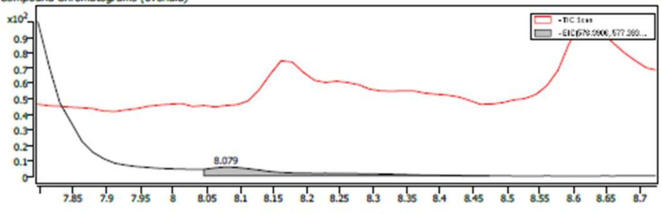

Structure

Target Screening Report

Species	m/z	Score (Tgt)	Score (Lib)	Score (DB)	Score (MFG)	Score (RT)
(M-H)- (M+CH3COO)-	520.3279 580.3374	99.52				

Compound Chromatograms (overlaid)

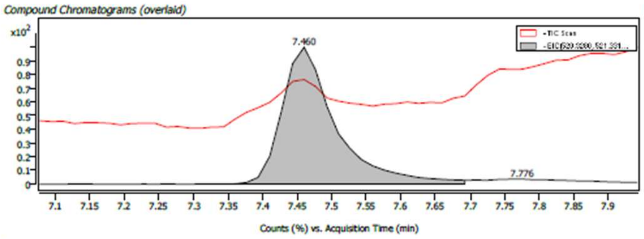

Structure

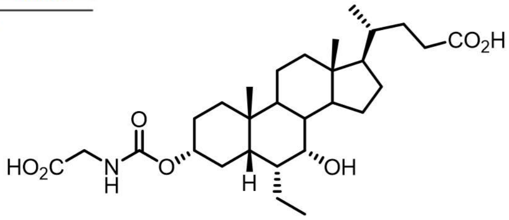

Compound Spectra (overlaid)

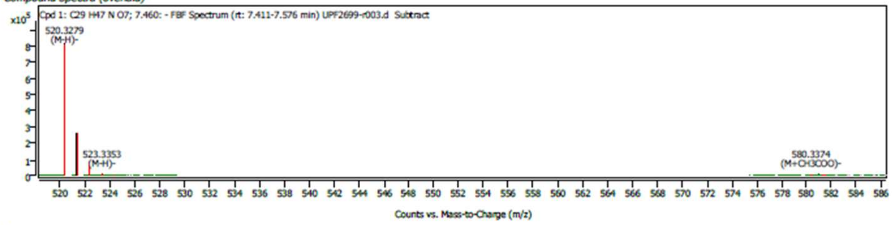

Compound ID Table

Name	Formula	Species	RT	RT Diff	Mass	CAS	ID Source	Score	Score (Lib)	Score (Tgt)
	C29 H47 N O7	(M-H)- (M+CH3COO)-	7.460		521.3350		FBF	99.52		99.52

Cpd. 2: C29 H47 N O7

Name	Formula	RT	RI	Mass	Diff (Tgt, ppm)	CAS	ID Source	Score	Algorithm
	C29 H47 N O7	7.776		521.3339	-2.63		FBF	90.80	FBF

Formula	Height	Area
C29 H47 N O7	85603.0984083508	1170803.73393727

Species	m/z	Score (Tgt)	Score (Lib)	Score (DB)	Score (MFG)	Score (RT)
(M-H)-	520.3268	90.80				

Compound Chromatograms (overlaid)

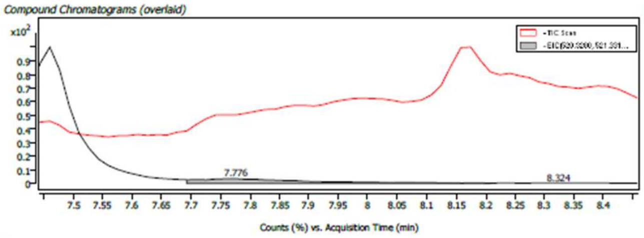

Structure

Target Screening Report

Agilent Trusted Answers

Compound Chromatograms (overlaid)

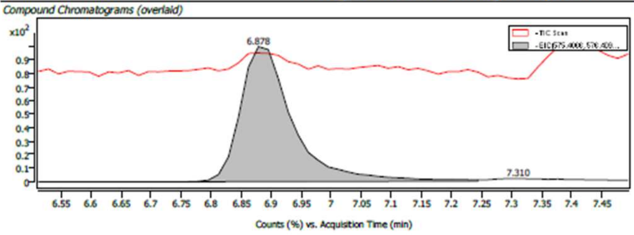

Structure

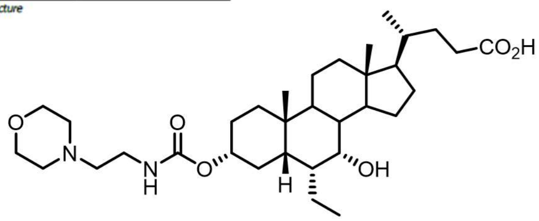

Compound Spectra (overlaid)

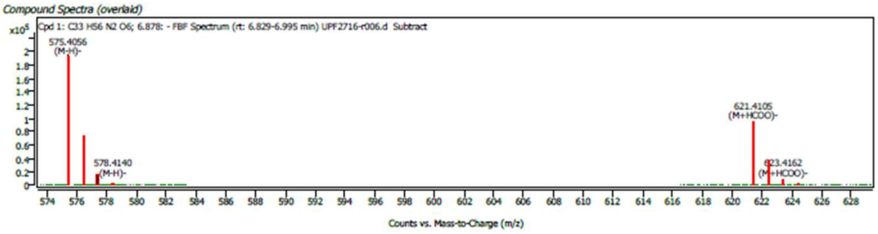

Compound ID Table

Name	Formula	Species	RT	RT Diff	Mass	CAS	ID Source	Score	Score (Lib)	Score (Tgt)
	C33 H56 N2 O6	(M-H)- (M+HCOO)-	6.878		576.4126		FBF	97.33		97.33

Cpd. 2: C33 H56 N2 O6

Name	Formula	RT	RI	Mass	Diff (Tgt, ppm)	CAS	ID Source	Score	Algorithm
	C33 H56 N2 O6	7.310		576.4115	-4.02		FBF	89.19	FBF

Formula	Height	Area
C33 H56 N2 O6	7548.11374018903	50806.1183128162

Species	m/z	Score (Tgt)	Score (Lib)	Score (DB)	Score (MFG)	Score (RT)
(M-H)- (M+HCOO)- (M+CH3COO)-	575.4048 621.4096 635.4230	89.19				

Compound Chromatograms (overlaid)

Structure

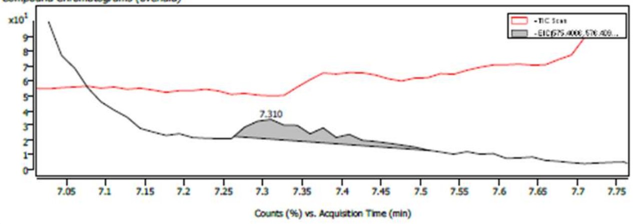

Target Screening Report

Compound Chromatograms (overlaid)

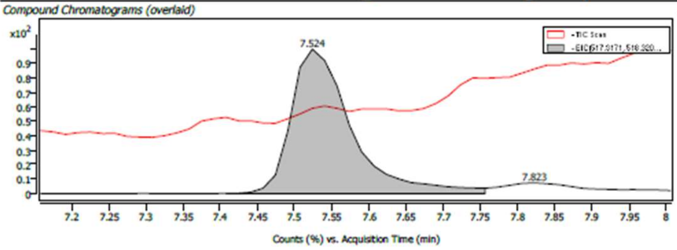

Structure

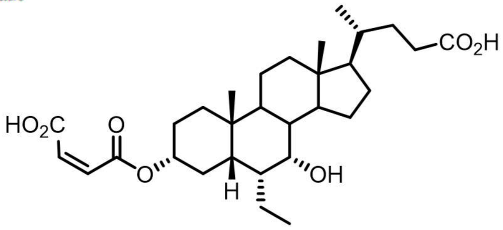

Compound Spectra (overlaid)

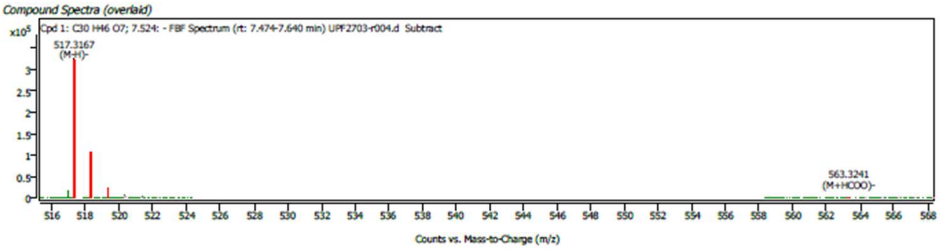

Compound ID Table

Name	Formula	Species	RT	RT Diff	Mass	CAS	ID Source	Score	Score (Lib)	Score (Tgt)
	C30 H46 O7	(M-H)-<br>(M+HCOO)-	7.524		518.3238		FBF	98.74		98.74

Cpd. 2: C30 H46 O7

Name	Formula	RT	RI	Mass	Diff (Tgt, ppm)	CAS	ID Source	Score	Algorithm
	C30 H46 O7	7.823		518.3229	-2.71		FBF	93.72	FBF

Formula	Height	Area
C30 H46 O7	65686.185863153	668867.908827021

Species	m/z	Score (Tgt)	Score (Lib)	Score (DB)	Score (MFG)	Score (RT)
(M-H)-	517.3156	93.72				

Compound Chromatograms (overlaid)

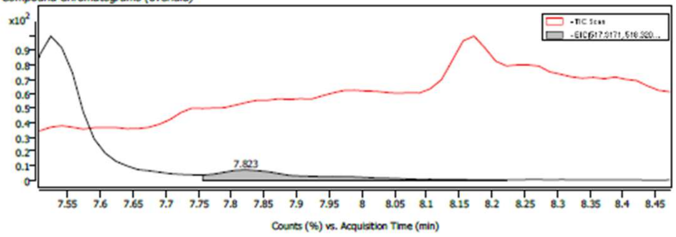

Structure

Target Screening Report

Compound Chromatograms (overlaid)

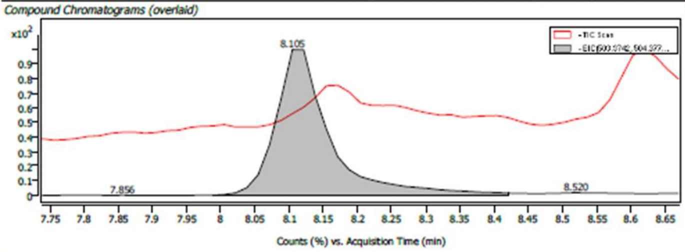

Structure

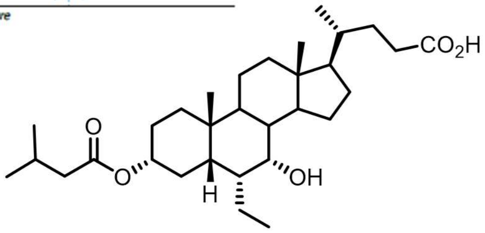

Compound Spectra (overlaid)

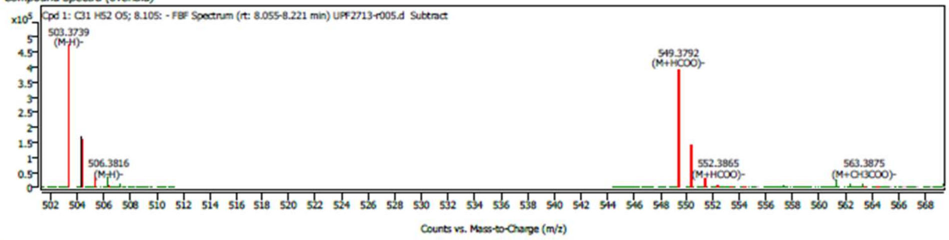

Compound ID Table

Name	Formula	Species	RT	RT Diff	Mass	CAS	ID Source	Score	Score (Lib)	Score (Tgt)
	C31 H52 O5	(M-H)- (M+HCOO)- (M+CH3COO)-	8.105		504.3809		FBF	98.46		98.46

Cpd. 2: C31 H52 O5

Name	Formula	RT	RI	Mass	Diff (Tgt, ppm)	CAS	ID Source	Score	Algorithm
	C31 H52 O5	8.520		504.3798	-3.25		FBF	75.16	FBF

Formula	Height	Area
C31 H52 O5	48163.7221235574	906368.7649352

Species	m/z	Score (Tgt)	Score (Lib)	Score (DB)	Score (MFG)	Score (RT)
(M-H)- (M+HCOO)-	503.3730 549.3783	75.16				

Compound Chromatograms (overlaid)

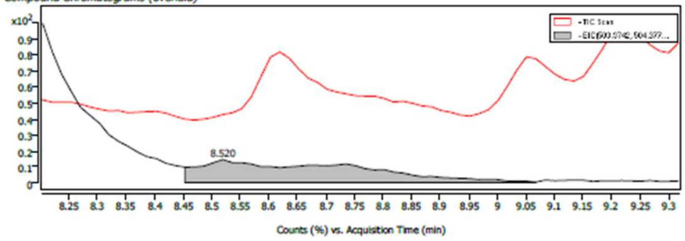

Structure

Target Screening Report

Agilent Trusted Answers

Species	m/z	Score (Tgt)	Score (Lib)	Score (DB)	Score (MFG)	Score (RT)
(M-H)- (M+HCOO)- (M+CH3COO)-	502.3891 548.3953 562.4074	98.77				

Compound Chromatograms (overlaid)

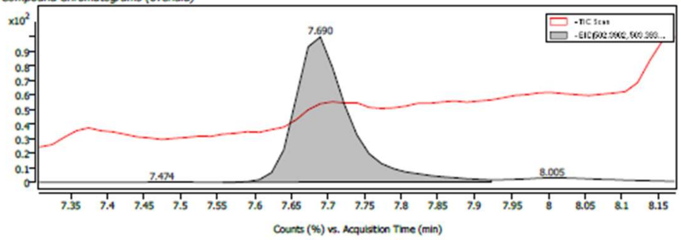

Structure

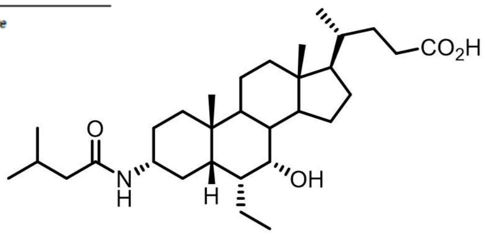

Compound Spectra (overlaid)

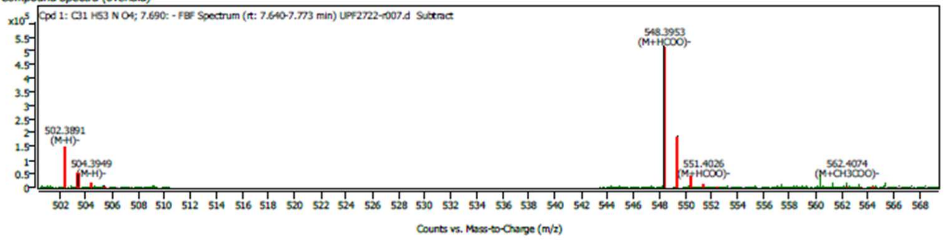

Compound ID Table

Name	Formula	Species	RT	RT Diff	Mass	CAS	ID Source	Score	Score (Lib)	Score (Tgt)
	C31 H53 N O4	(M-H)- (M+HCOO)- (M+CH3COO)-	7.690		503.3967		FBF	98.77		98.77

Cpd. 2: C31 H53 N O4

Name	Formula	RT	RI	Mass	Diff (Tgt, ppm)	CAS	ID Source	Score	Algorithm
	C31 H53 N O4	8.005		503.3951	-4.72		FBF	90.79	FBF

Formula	Height	Area
C31 H53 N O4	41926.1161840814	385200.614949836

Species	m/z	Score (Tgt)	Score (Lib)	Score (DB)	Score (MFG)	Score (RT)
(M-H)- (M+HCOO)- (M+CH3COO)-	502.3881 548.3940 562.4043	90.79				

Compound Chromatograms (overlaid)

Structure

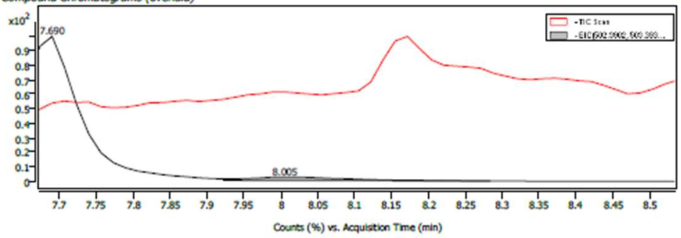

Target Screening Report

Compound Chromatograms (overlaid)

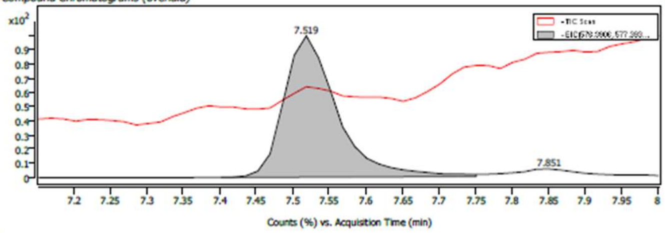

Structure

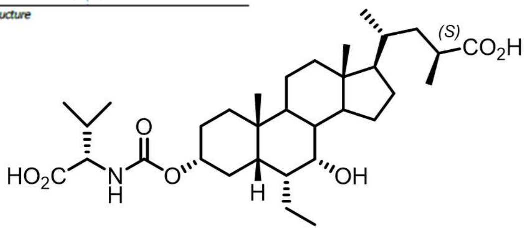

Compound Spectra (overlaid)

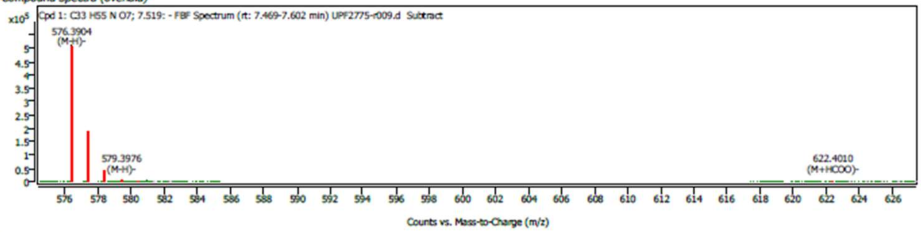

Compound ID Table

Name	Formula	Species	RT	RT Diff	Mass	CAS	ID Source	Score	Score (Lib)	Score (Tgt)
	C33 H55 N O7	(M-H)- (M+HCOO)-	7.519		577.3974		FBF	99.02		99.02

Cpd. 2: C33 H55 N O7

Name	Formula	RT	RI	Mass	Diff (Tgt, ppm)	CAS	ID Source	Score	Algorithm
	C33 H55 N O7	7.851		577.3961	-3.02		FBF	92.47	FBF

Formula	Height	Area
C33 H55 N O7	60363.9197242294	349938.239465245

Species	m/z	Score (Tgt)	Score (Lib)	Score (DB)	Score (MFG)	Score (RT)
(M-H)- (M+CH3COO)-	576.3890 636.4010	92.47				

Compound Chromatograms (overlaid)

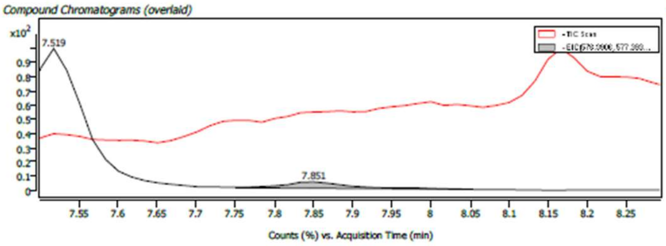

Structure

Target Screening Report

Compound Chromatograms (overlaid)

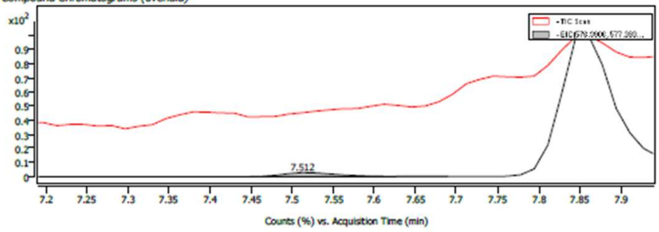

Structure

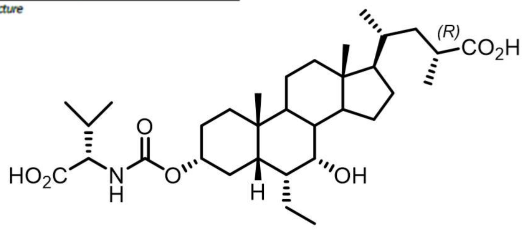

Compound Spectra (overlaid)

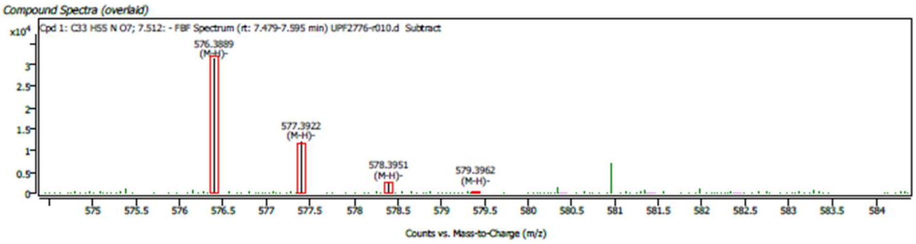

Compound ID Table

Name	Formula	Species	RT	RT Diff	Mass	CAS	ID Source	Score	Score (Lib)	Score (Tgt)
	C33 H55 N O7	(M-H)-	7.512		577.3962		FBF	95.26		95.26

Cpd. 2: C33 H55 N O7

Name	Formula	RT	RI	Mass	Diff (Tgt, ppm)	CAS	ID Source	Score	Algorithm
	C33 H55 N O7	7.861		577.3975	-0.56		FBF	99.45	FBF

Formula	Height	Area
C33 H55 N O7	2546366.52675158	13547648.4887542

Species	m/z	Score (Tgt)	Score (Lib)	Score (DB)	Score (MFG)	Score (RT)
(M-H)- (M+HCOO)- (M+CH3COO)-	576.3903 622.3912 636.4078	99.45				

Compound Chromatograms (overlaid)

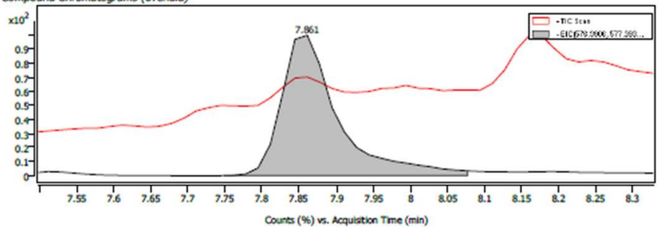

Structure

Target Screening Report

Agilent

Compound Chromatograms (overlaid)

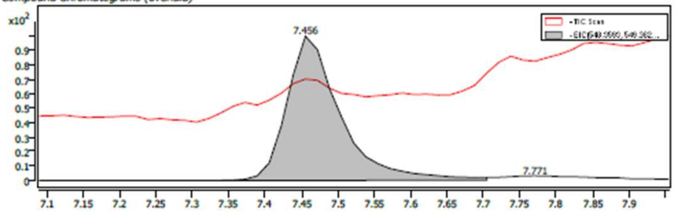

Structure

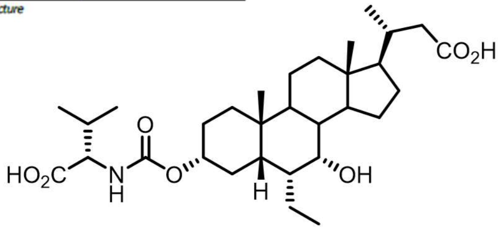

Compound Spectra (overlaid)

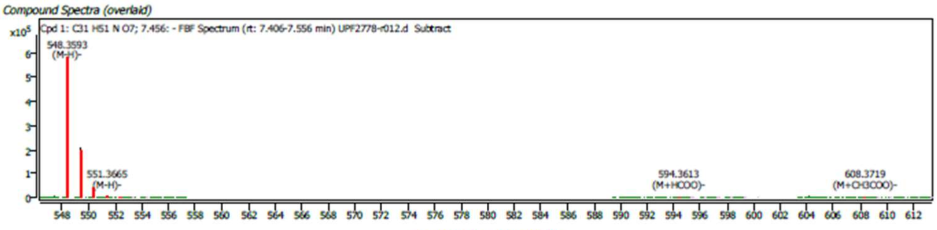

Compound ID Table

Name	Formula	Species	RT	RT Diff	Mass	CAS	ID Source	Score	Score (Lib)	Score (Tgt)
	C31 H51 N O7	(M-H)- (M+HCOO)- (M+CH3COO)-	7.456		549.3663		FBF	99.03		99.03

Cpd. 2: C31 H51 N O7

Name	Formula	RT	RI	Mass	Diff (Tgt, ppm)	CAS	ID Source	Score	Algorithm
	C31 H51 N O7	7.771		549.3651	-2.57		FBF	89.38	FBF

Formula	Height	Area
C31 H51 N O7	50075.8821308297	562435.14236977

Species	m/z	Score (Tgt)	Score (Lib)	Score (DB)	Score (MFG)	Score (RT)
(M-H)- (M+HCOO)- (M+CH3COO)-	548.3577 594.3751 608.3984	89.38				

Compound Chromatograms (overlaid)

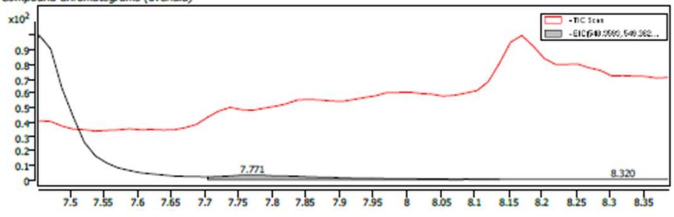

Structure

Compound Summary

Cpd	Name	Formula	CAS	RT	Mass	Mass (Tgt)	Diff (Tgt, ppm)	Score	Algorithm
1		C31 H53 N O9 S		7.446	615.3436	615.3441	-0.78	98.06	FBF

Compound Details

Cpd. 1: C31 H53 N O9 S

Name	Formula	RT	RI	Mass	Diff (Tgt, ppm)	CAS	ID Source	Score	Algorithm
	C31 H53 N O9 S	7.446		615.3436	-0.78		FBF	98.06	FBF

Formula	Height	Area
C31 H53 N O9 S	893059.569417001	5516462.38324573

Species	m/z	Score (Tgt)	Score (Lib)	Score (DB)	Score (MFG)	Score (RT)
(M-H)-	614.3366	98.06				

Target Screening Report

Compound Chromatograms (overlaid)

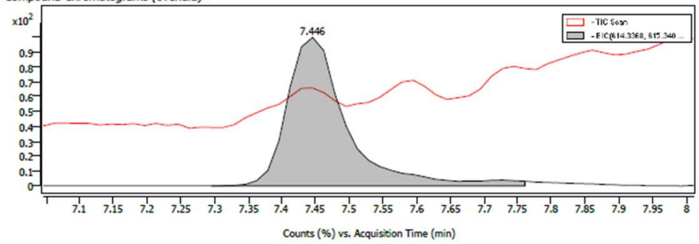

Structure

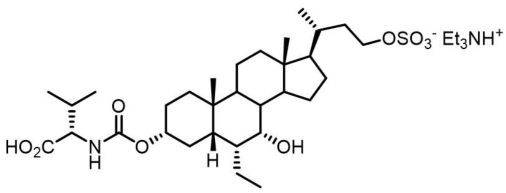

Compound Spectra (overlaid)

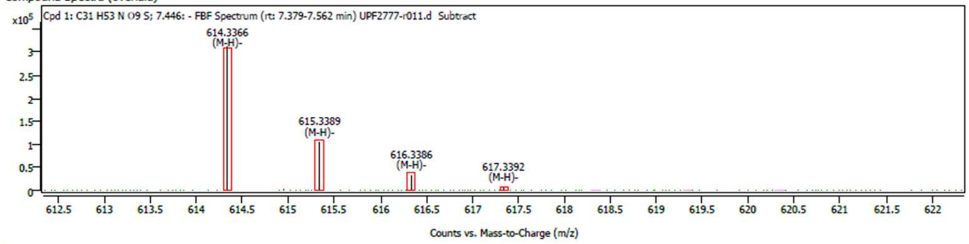

Compound ID Table

Name	Formula	Species	RT	RT Diff	Mass	CAS	ID Source	Score	Score (Lib)	Score (Tgt)
	C31 H53 N O9 S	(M-H)-	7.446		615.3436		FBF	98.06		98.06

MassHunter Qual 10.0
(End of Report)

Compound Summary

Cpd	Name	Formula	CAS	RT	Mass	Mass (Tgt)	Diff (Tgt, ppm)	Score	Algorithm
1		C32 H51 N3 O7		7.354	589.3718	589.3727	-1.51	97.81	FBF

Compound Details

Cpd. 1: C32 H51 N3 O7

Name	Formula	RT	RI	Mass	Diff (Tgt, ppm)	CAS	ID Source	Score	Algorithm
	C32 H51 N3 O7	7.354		589.3718	-1.51		FBF	97.81	FBF

Formula	Height	Area
C32 H51 N3 O7	654486.293655322	3359191.20959954

Species	m/z	Score (Tgt)	Score (Lib)	Score (DB)	Score (MFG)	Score (RT)
(M-H)- (M+CH3COO)-	588.3647 648.3987	97.81				

Target Screening Report

Agilent Trusted Answers

Compound Chromatograms (overlaid)

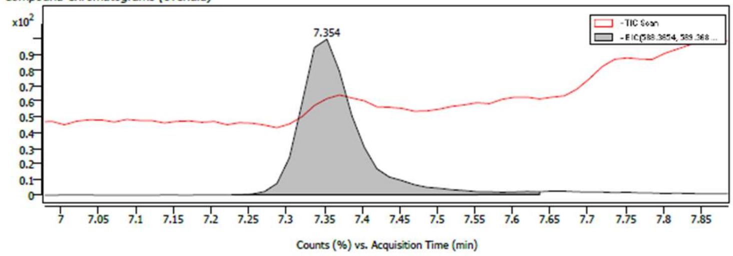

Structure

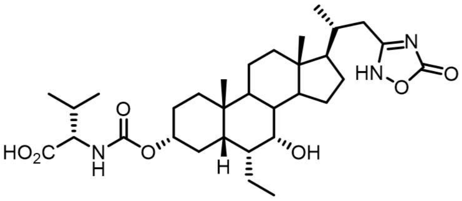

Compound Spectra (overlaid)

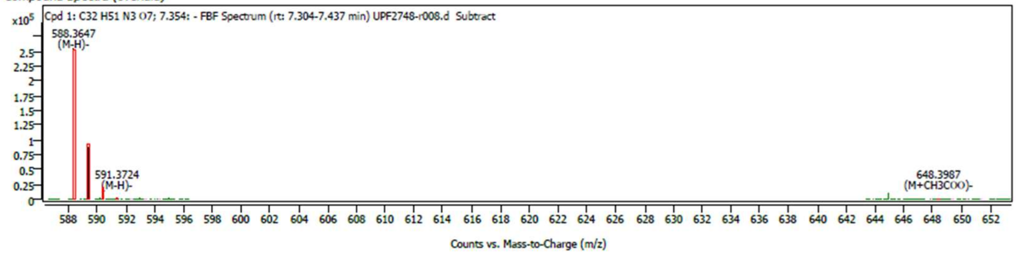

Compound ID Table

Name	Formula	Species	RT	RT Diff	Mass	CAS	ID Source	Score	Score (Lib)	Score (Tgt)
	C32 H51 N3 O7	(M-H)- (M+CH3COO)-	7.354		589.3718		FBF	97.81		97.81

MassHunter Qual 10.0
(End of Report)

5. Metabolic stability study - LC-MS/MS analysis

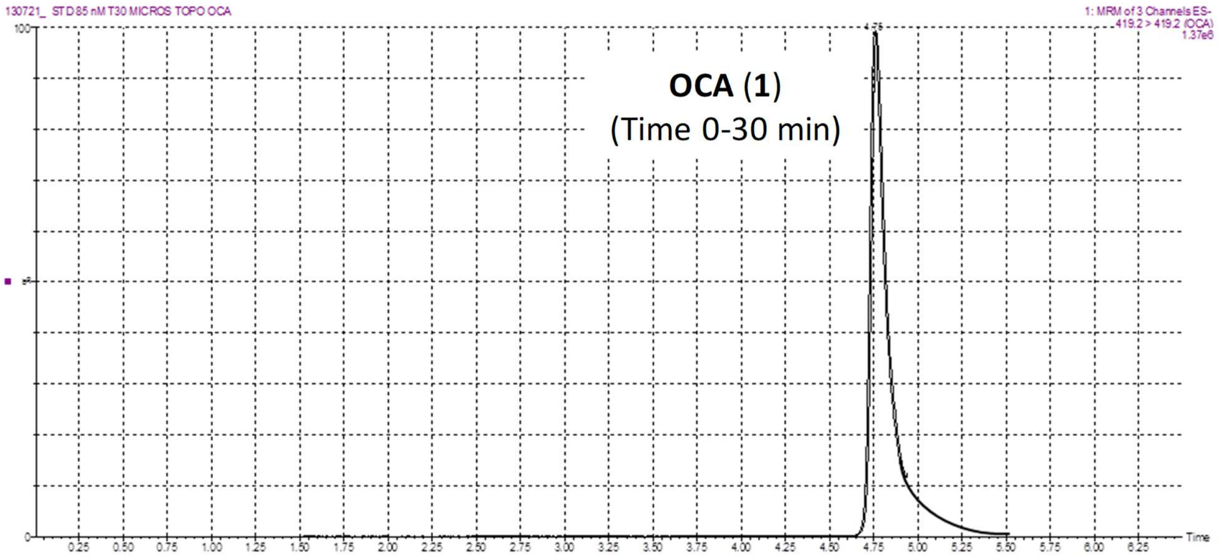

Figure S1. Chromatogram from time 0 minute to 30 minute (acquisition window, MRM) of OCA (**1**) in mouse microsome sample.

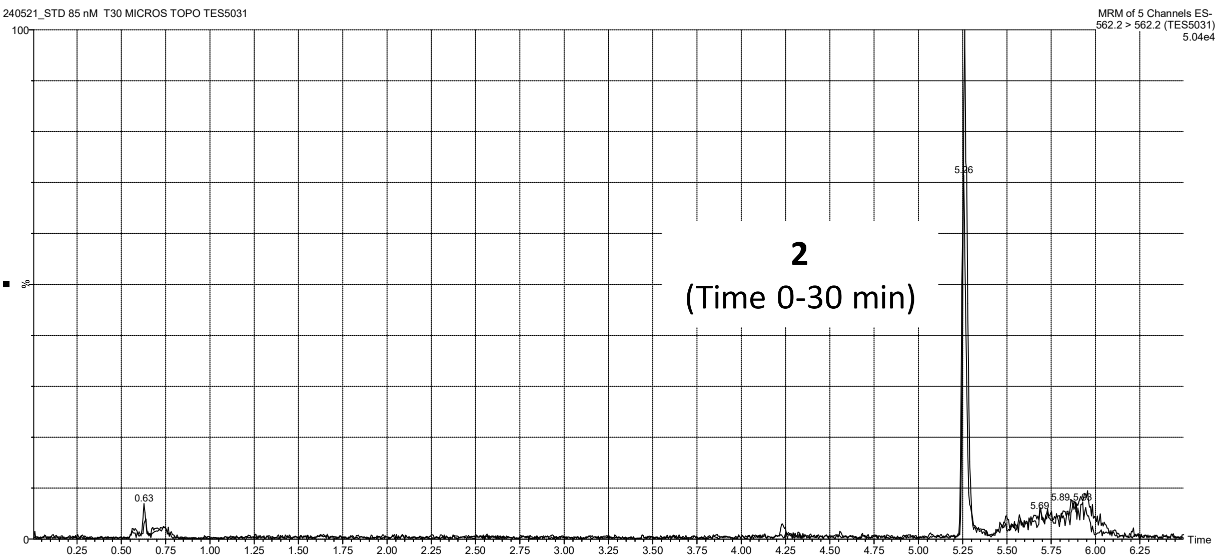

Figure S2. Chromatogram from time 0 minute to 30 minute (acquisition window, MRM) of **2** in mouse microsome sample.

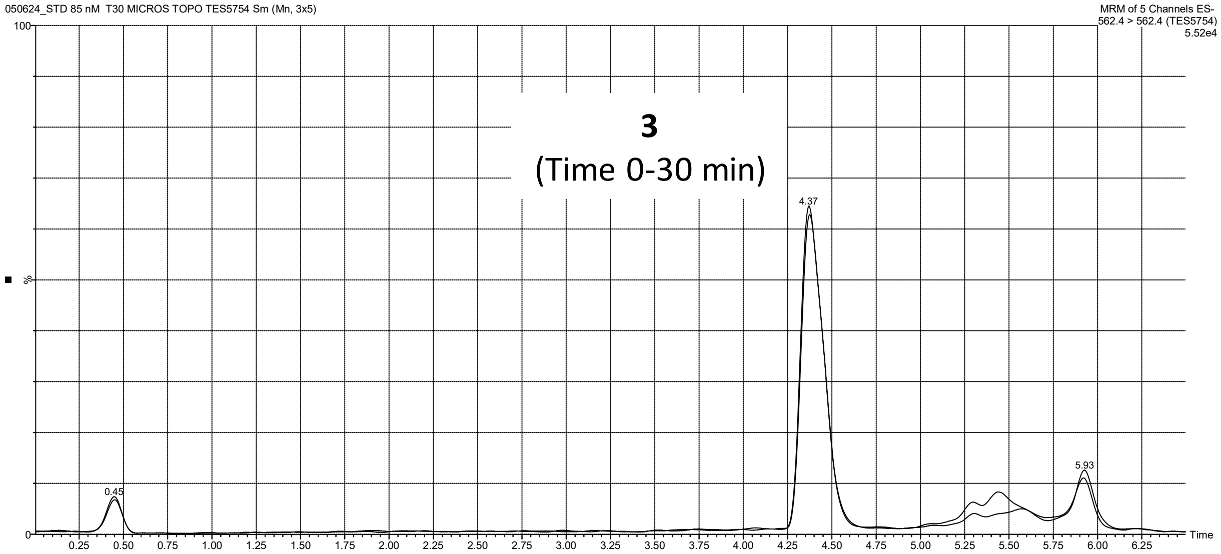

Figure S3. Chromatogram from time 0 minute to 30 minute (acquisition window, MRM) of **3** in mouse microsome sample.

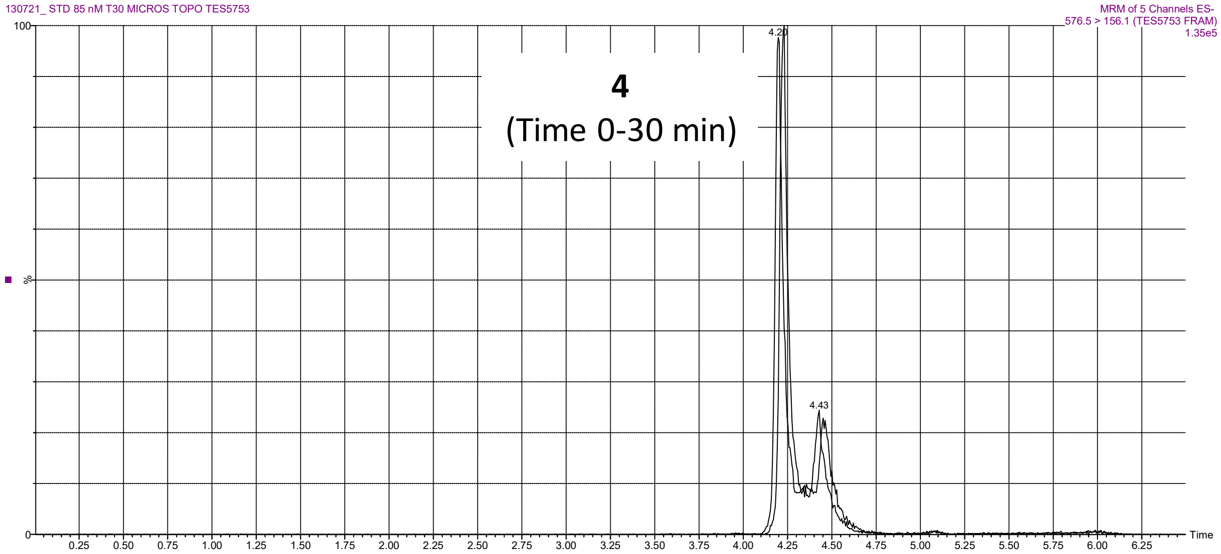

Figure S4. Chromatogram from time 0 minute to 30 minute (acquisition window, MRM) of **4** in mouse microsome sample.

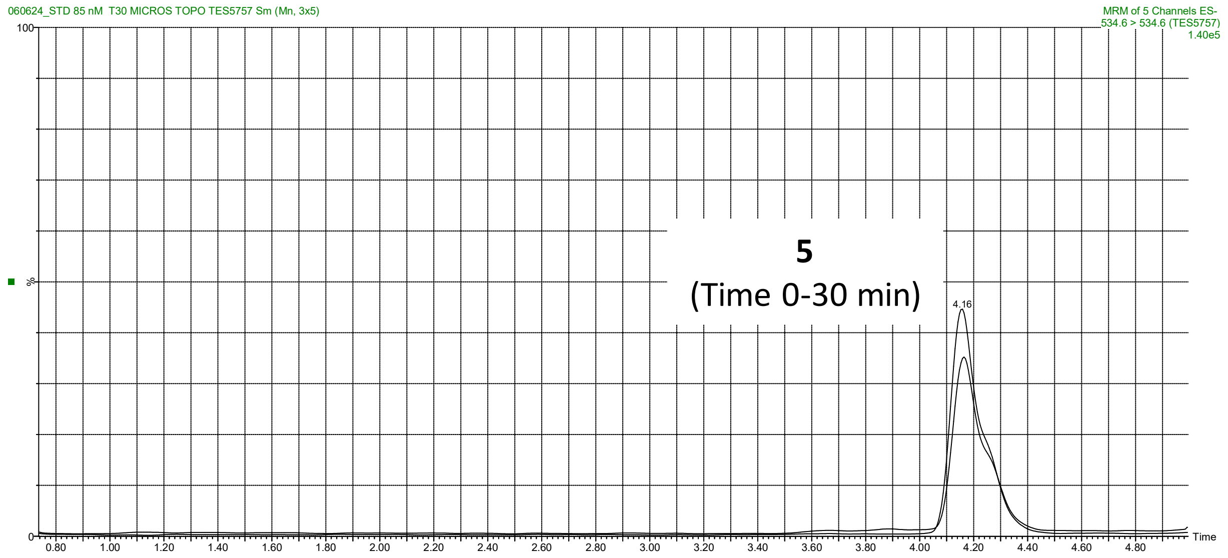

Figure S5. Chromatogram from time 0 minute to 30 minute (acquisition window, MRM) of **5** in mouse microsome sample.

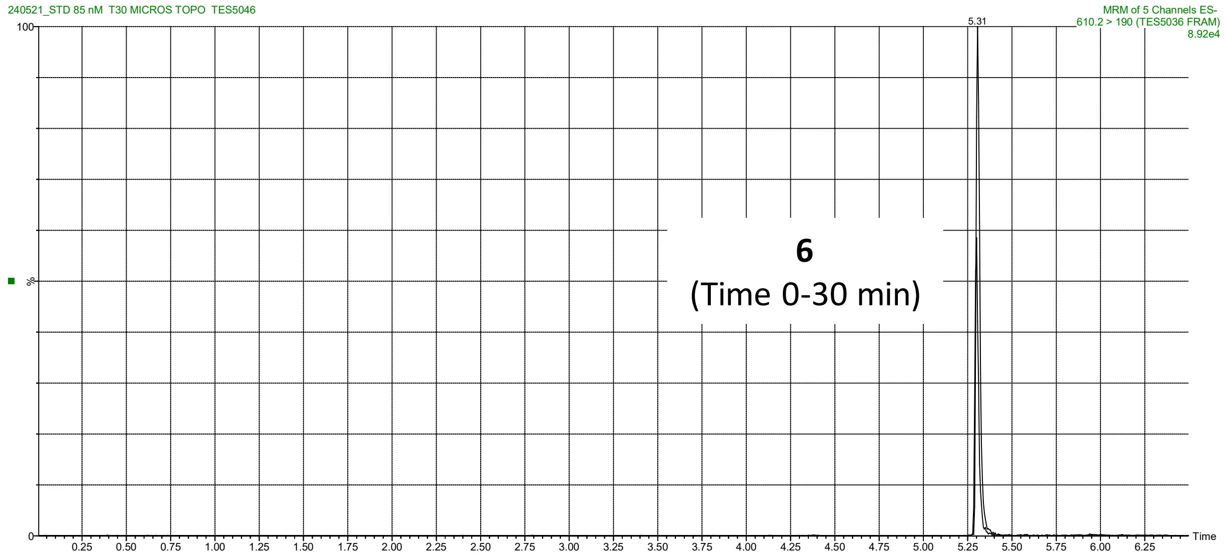

Figure S6. Chromatogram from time 0 minute to 30 minute (acquisition window, MRM) of **6** in mouse microsome sample.

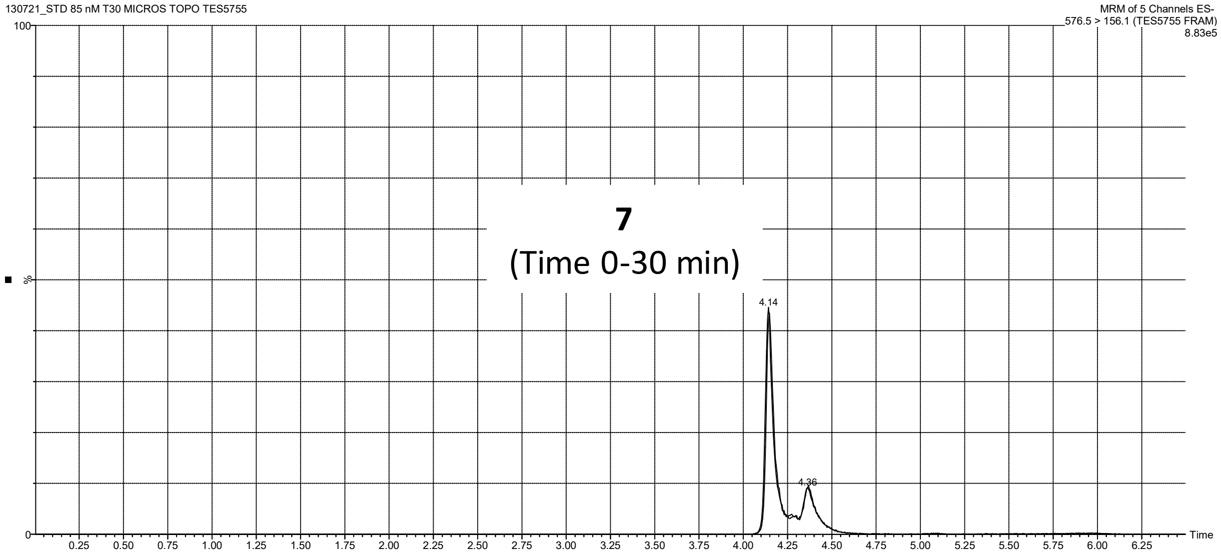

Figure S7. Chromatogram from time 0 minute to 30 minute (acquisition window, MRM) of **7** in mouse microsome sample.

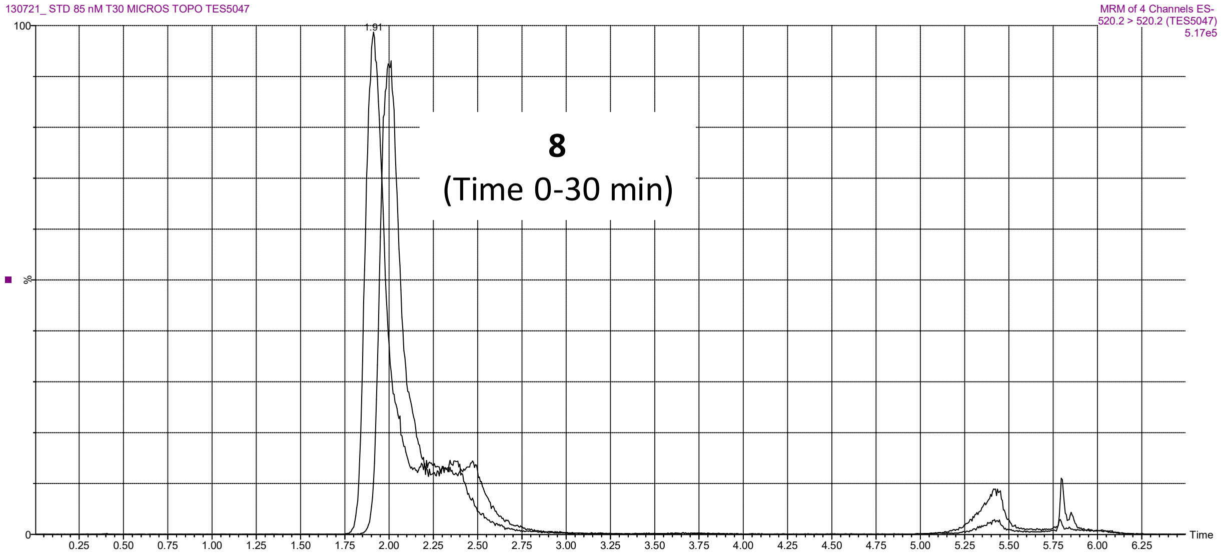

Figure S8. Chromatogram from time 0 minute to 30 minute (acquisition window, MRM) of **8** in mouse microsome sample.

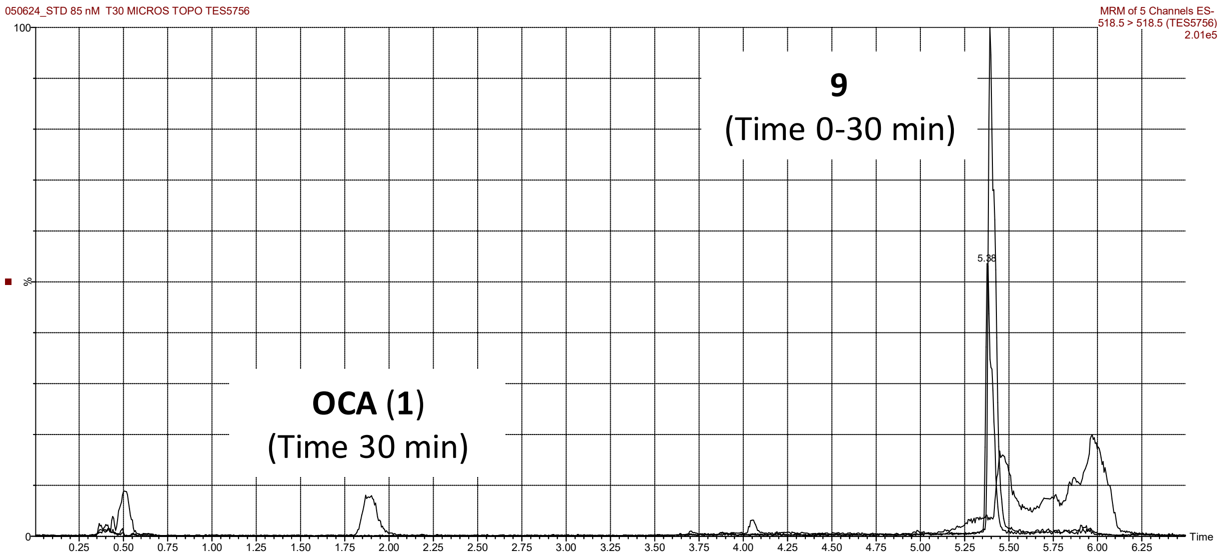

Figure S9. Chromatogram from time 0 minute to 30 minute (acquisition window, MRM) of **9** and OCA (**1**) in mouse microsome sample.

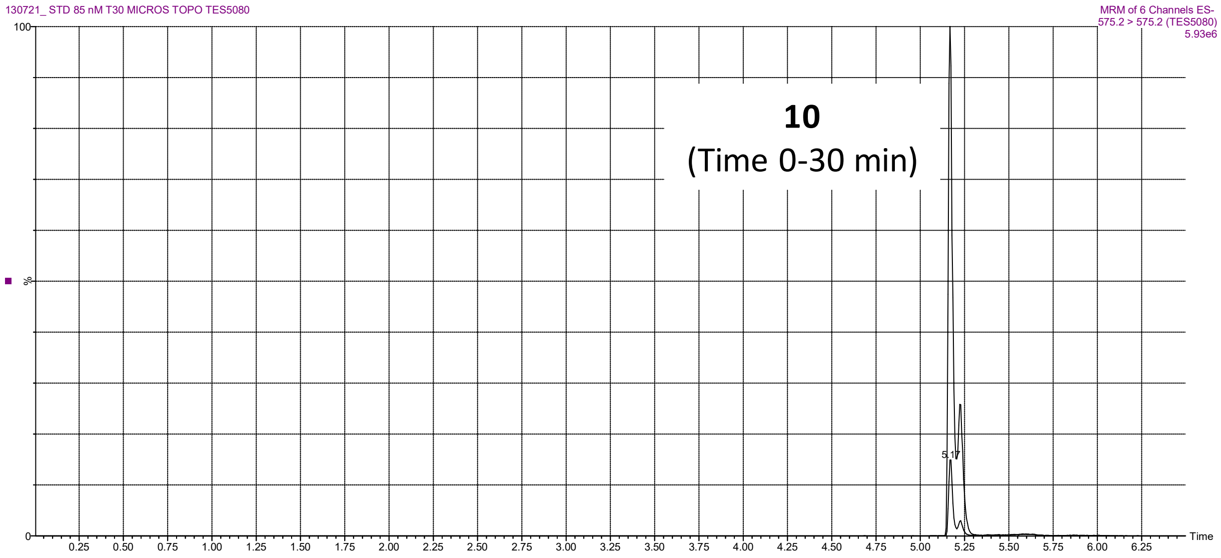

Figure S10. Chromatogram from time 0 minute to 30 minute (acquisition window, MRM) of **10** in mouse microsome sample.

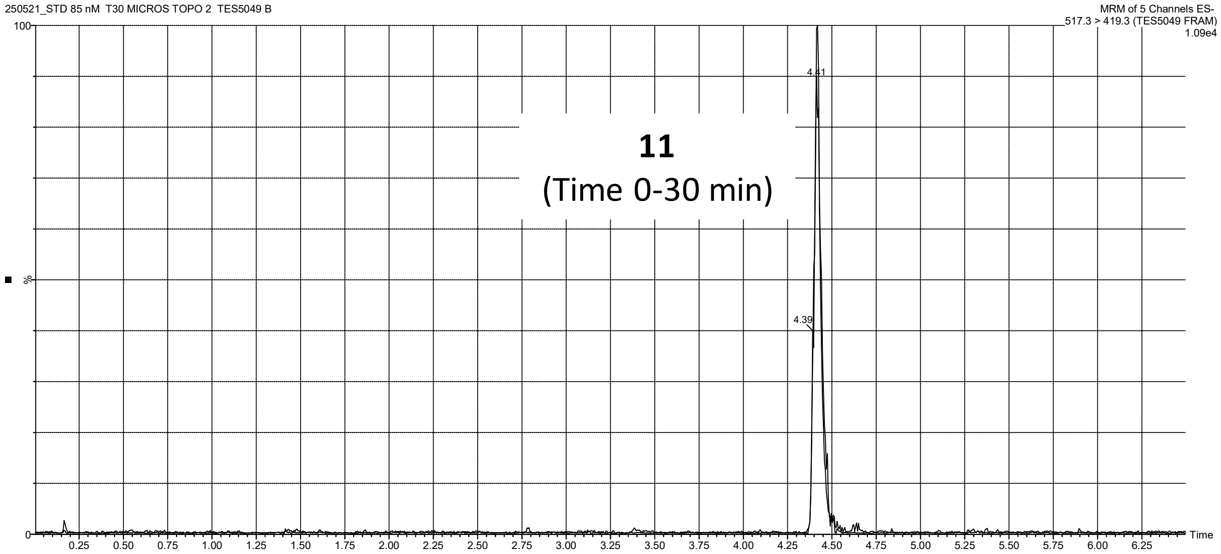

Figure S11. Chromatogram from time 0 minute to 30 minute (acquisition window, MRM) of **11** in mouse microsome sample.

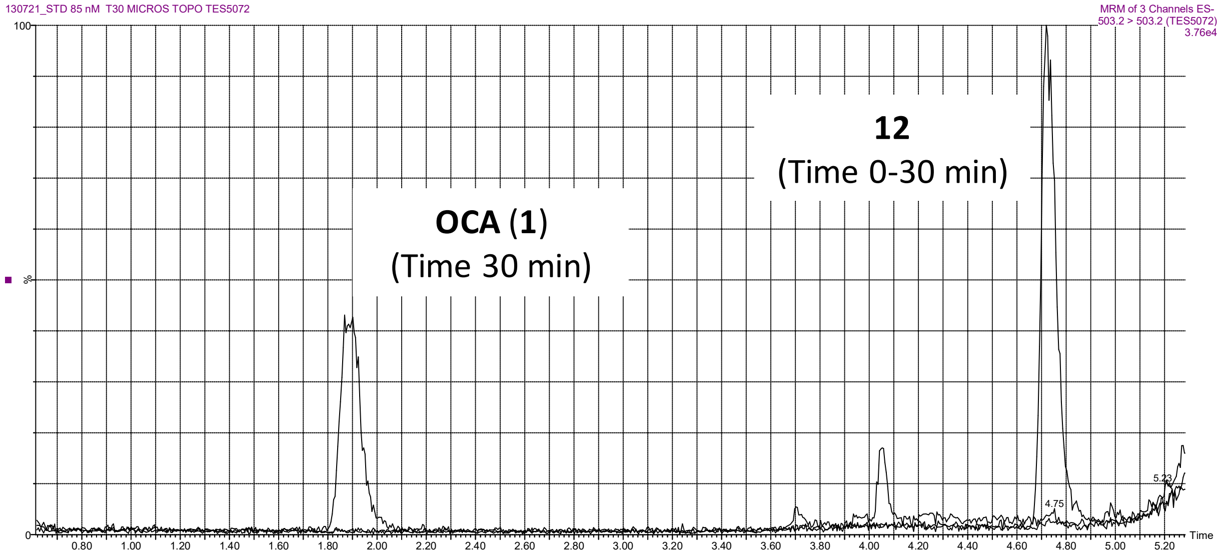

Figure S12. Chromatogram from time 0 minute to 30 minute (acquisition window, MRM) of **12** and OCA (**1**) in mouse microsome sample.

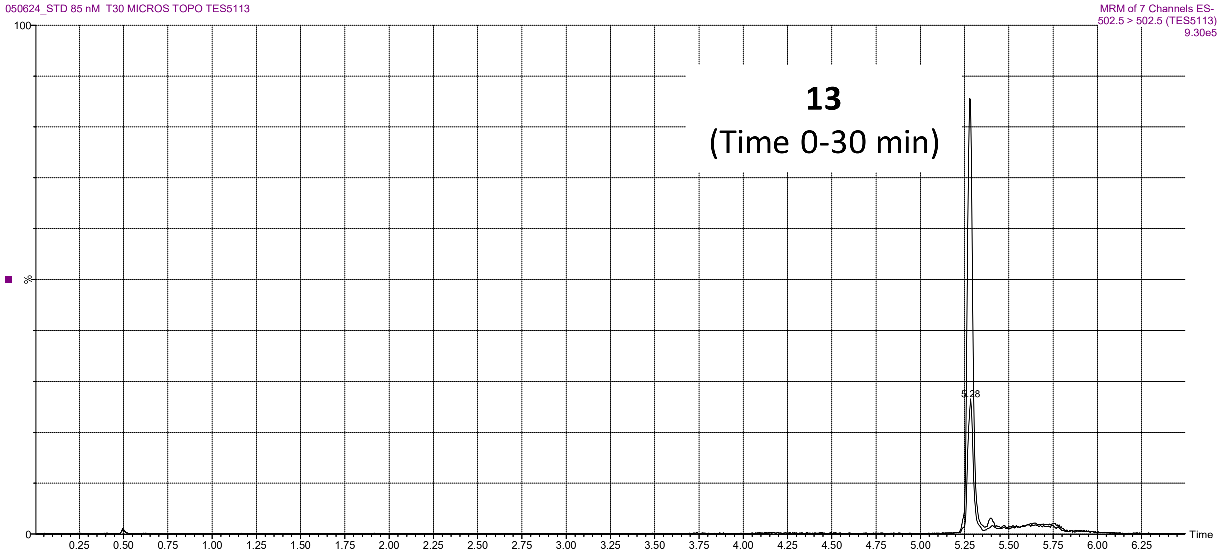

Figure S13. Chromatogram from time 0 minute to 30 minute (acquisition window, MRM) of **13** in mouse microsome sample.

Table S1. Metabolic stability in mouse and human microsomes of C3-modified obeticholic acid derivatives **2** and **16**.

**Cmpd**	**Microsomal stability (mouse)**			**Microsomal stability (human)**		
	**$t_{1/2}$ (min)[a]**	**CL_{int}[b]**	**Test item % remaining last time point**	**$t_{1/2}$ (min)[a]**	**CL_{int}[b]**	**Test item % remaining last time point**
OCA (**1**)	> 120	< 11.59	100	> 120	< 11.59	100
**2**	> 120	< 11.59	100	> 120	< 11.59	100
**16**	> 120	< 11.59	100	> 120	< 11.59	100

[a] Half-life in mouse and human microsomes expressed in minutes. Values are mean ± SD from duplicates by LC-MS/MS analysis. [b] Intrinsic clearance expressed in µL/min/mg protein. Values are mean ± SD from duplicates.

6. FXR Molecular dynamics analyses

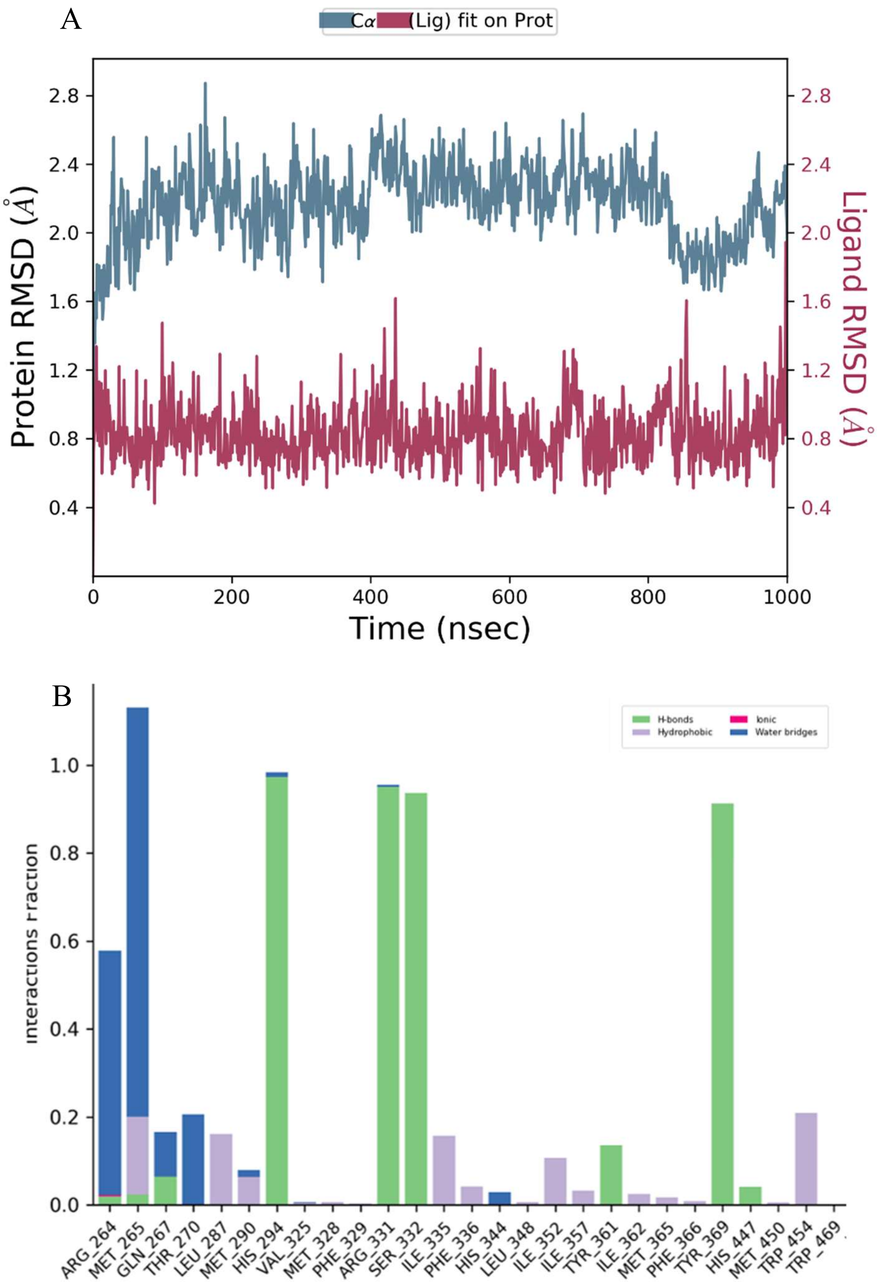

Figure S14. Protein-ligand RMSD (A) and protein-ligand interactions (B) registered for compound **1**.

A

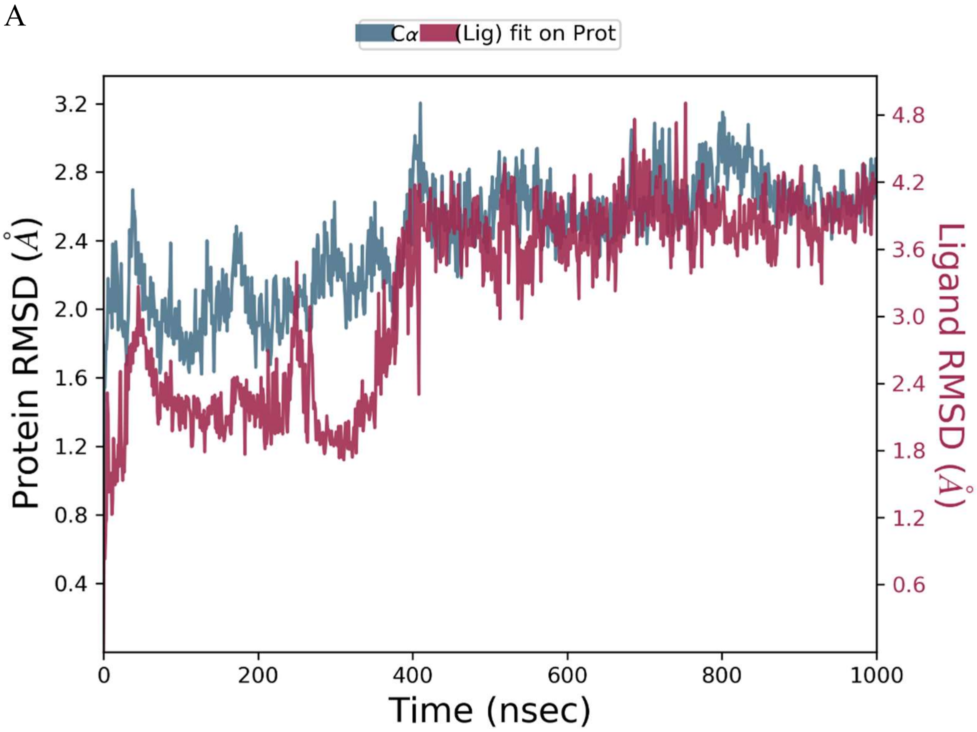

B

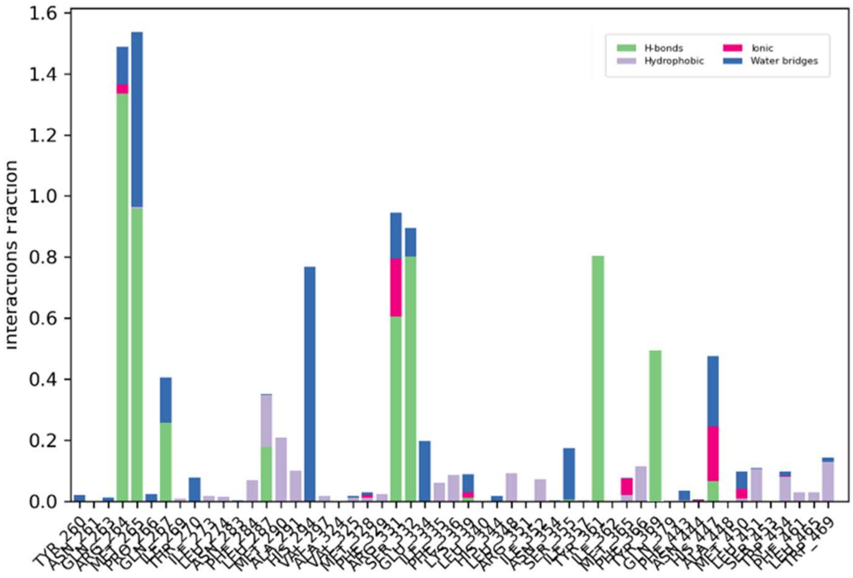

Figure S15. Protein-ligand RMSD (A) and protein-ligand interactions (B) registered for compound **2**.

A

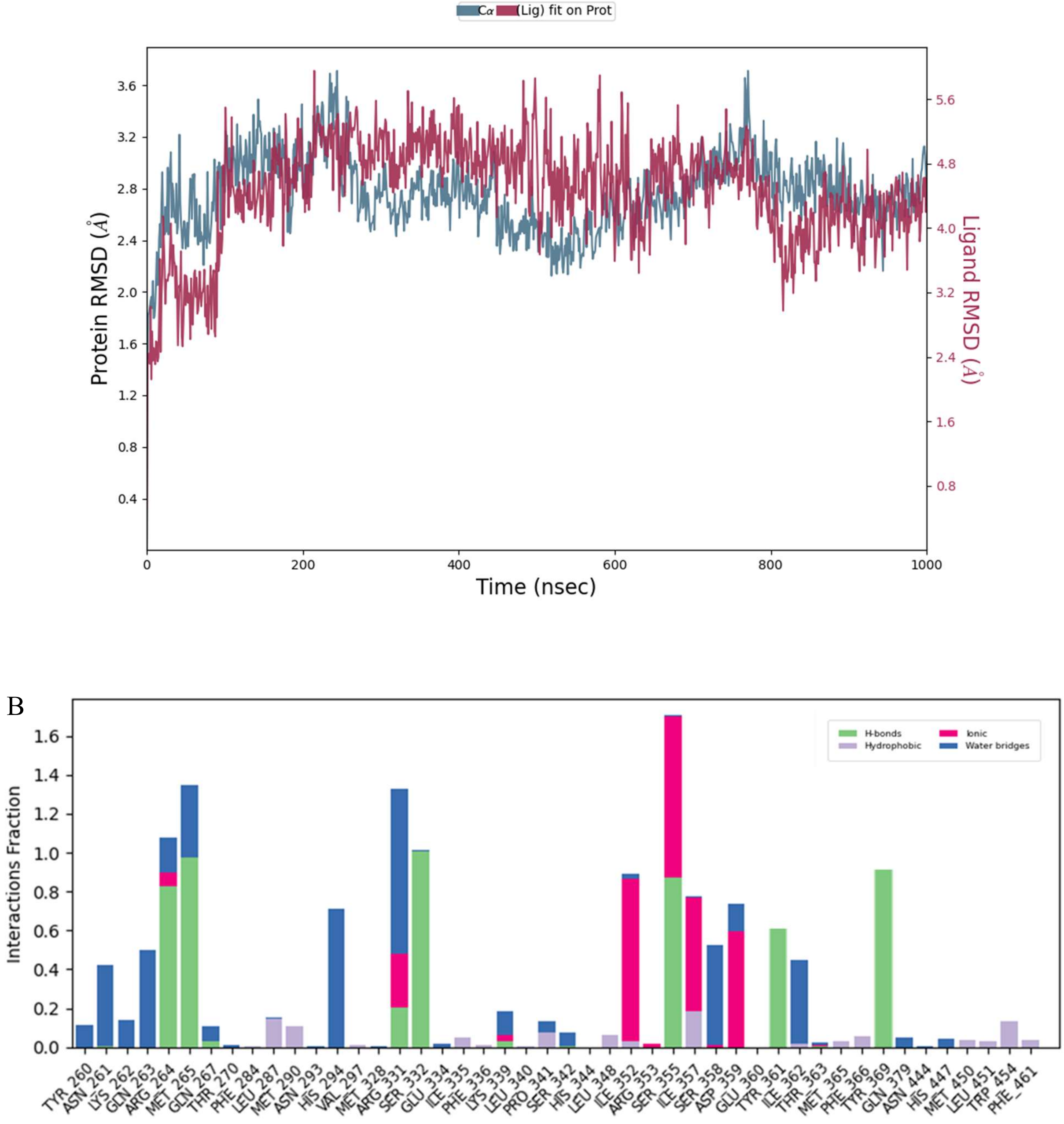

Figure S16. Protein-ligand RMSD (A) and protein-ligand interactions (B) registered for compound **16**.

A

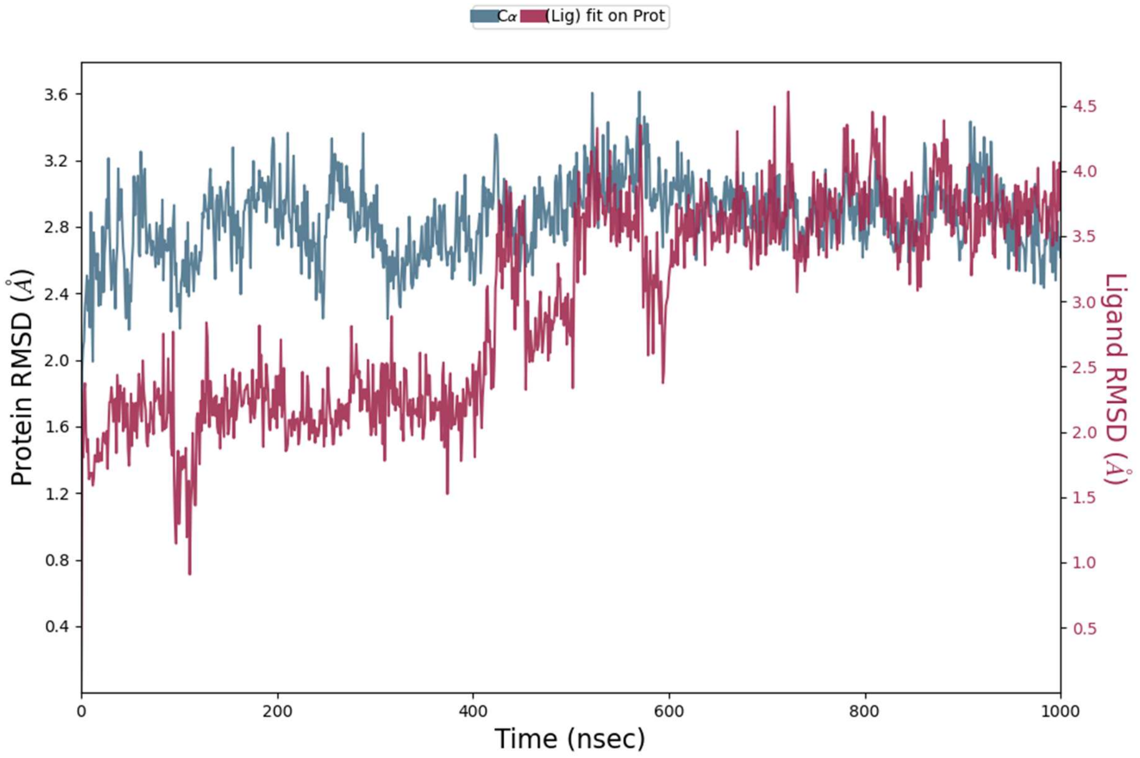

B

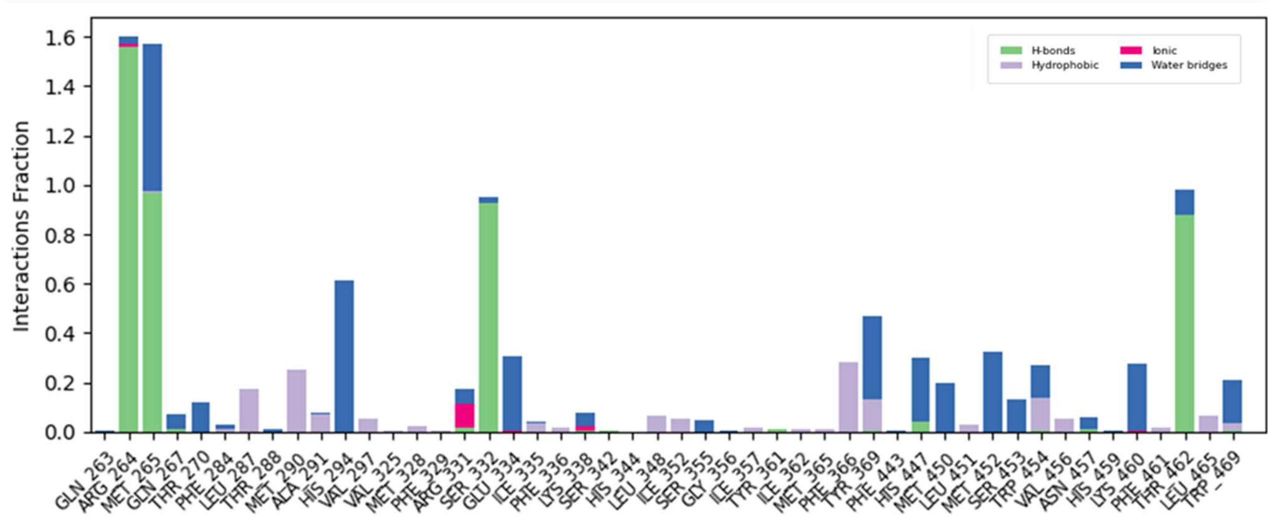

Figure S17. Protein-ligand RMSD (A) and protein-ligand interactions (B) registered for compound **14b**.

A

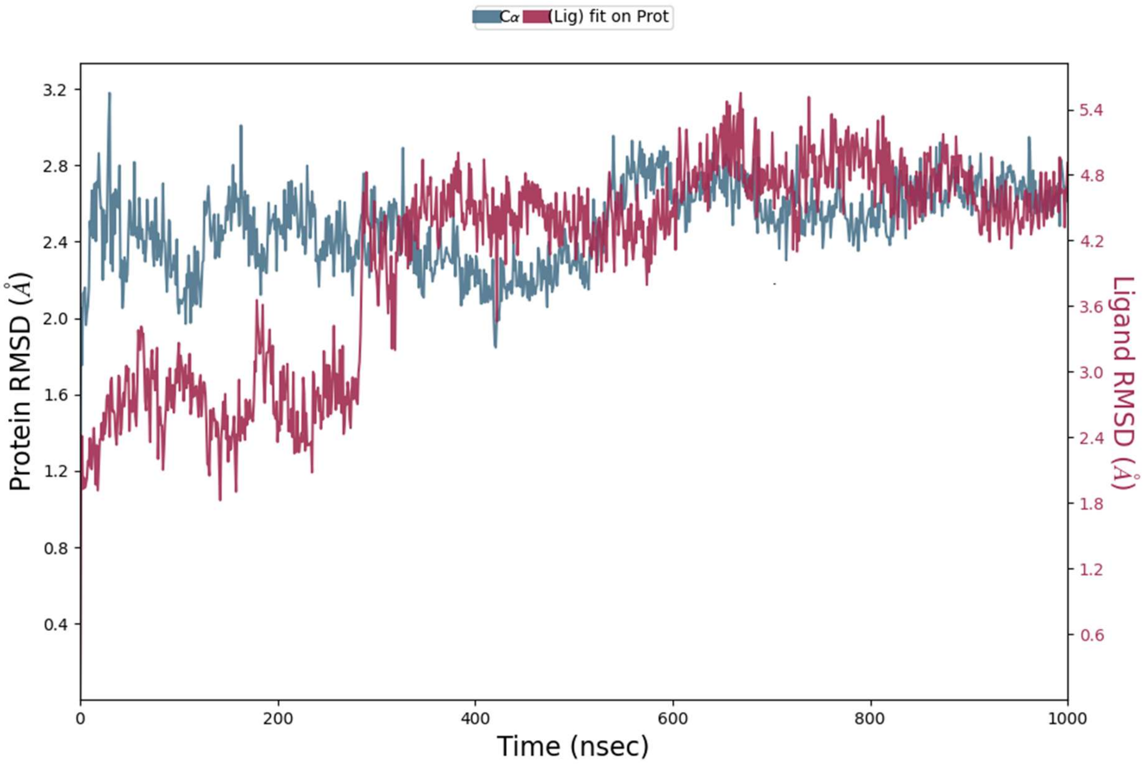

B

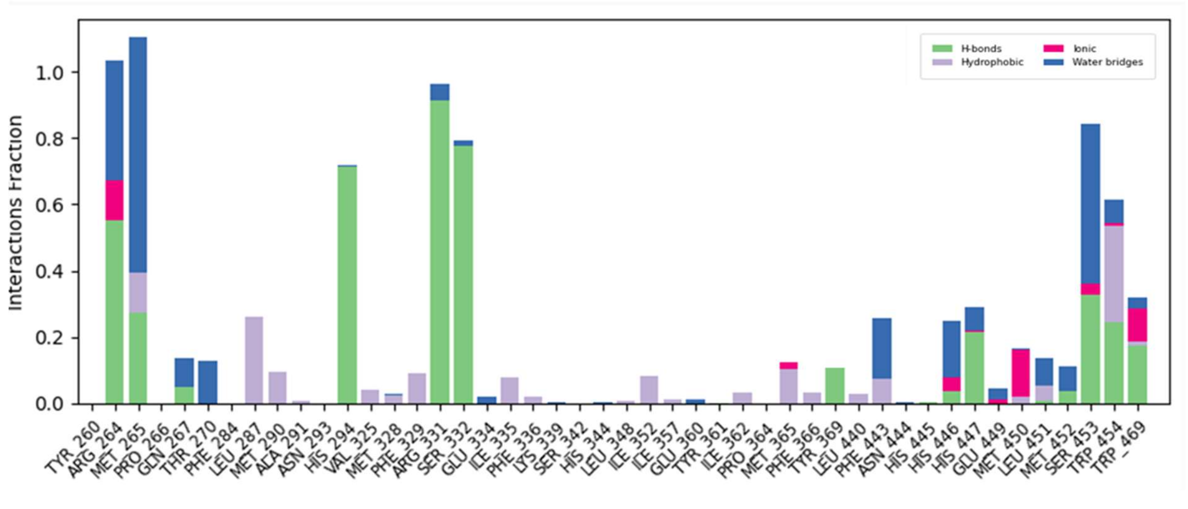

Figure S18. Protein-ligand RMSD (A) and protein-ligand interactions (B) registered for compound **14a**.

A

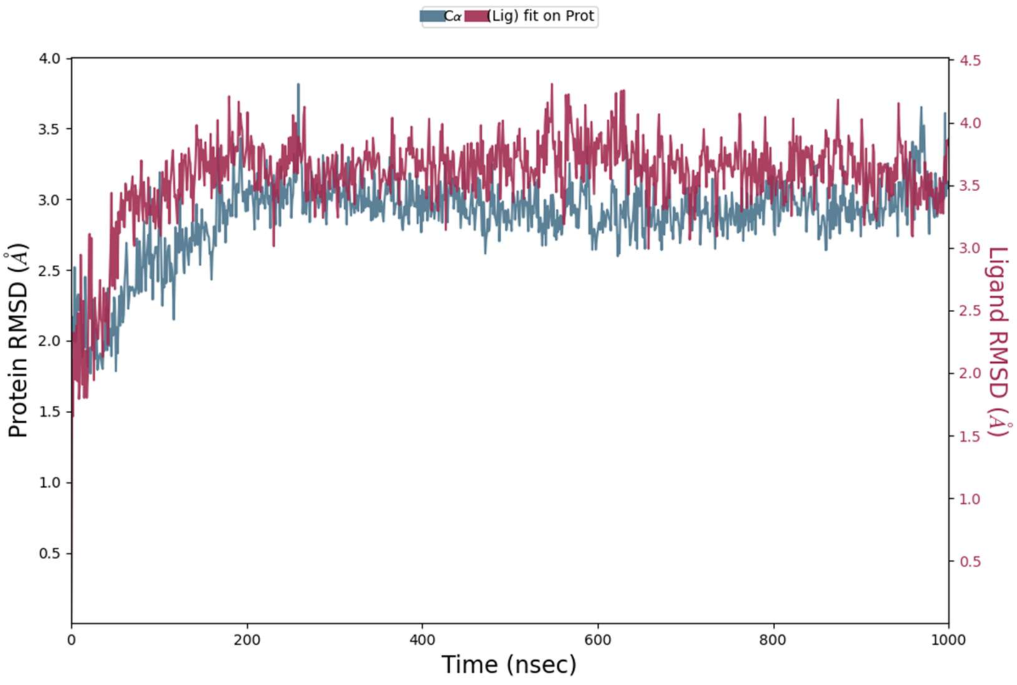

B

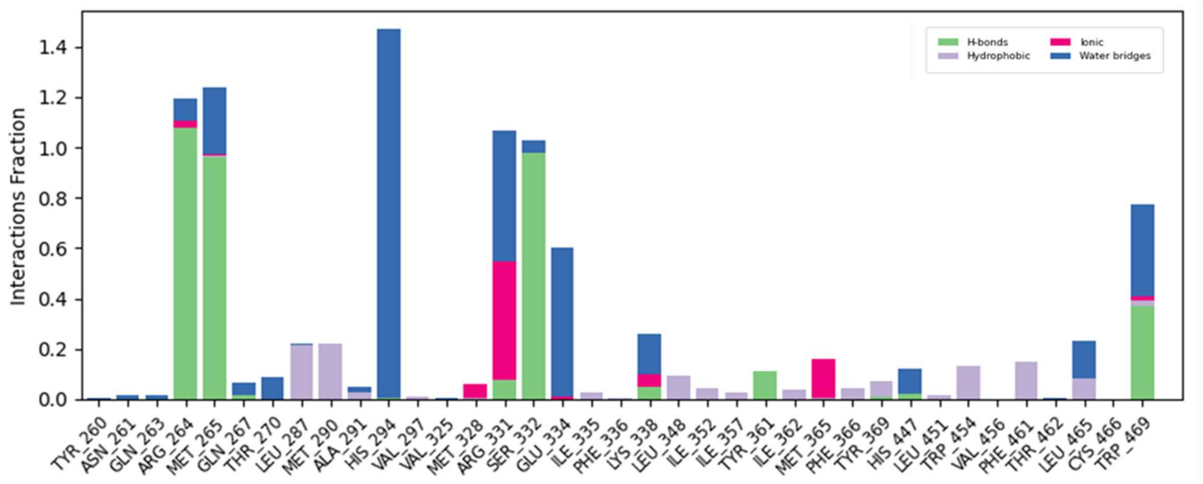

Figure S19. Protein-ligand RMSD (A) and protein-ligand interactions (B) registered for compound **15**.

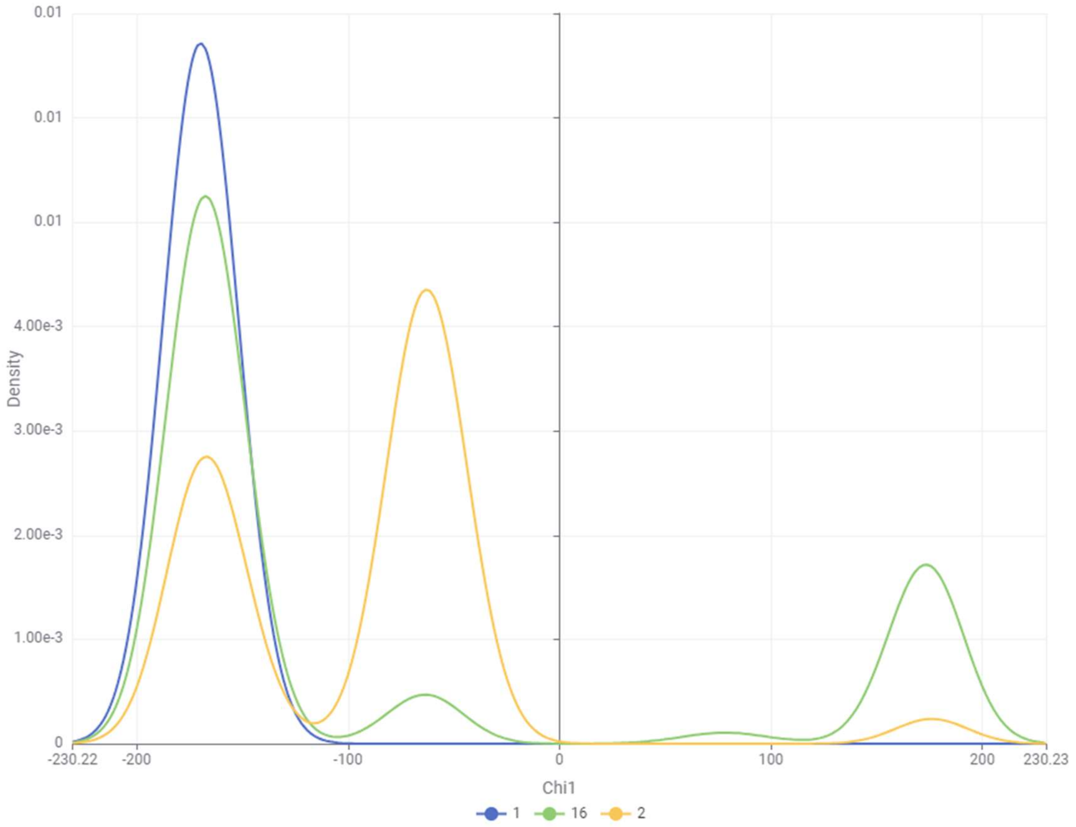

Figure S20. Density of the torsional angle chi1 (N-CA-CB-CG) values of Trp454 recorded in the MD simulations of compounds **1**, **2** and **16**. As observed in the paper of Asthana *et al.,*[5] the partial agonists represent a trimodal distribution of the chi1 values, while the agonist displays a unimodal one.

7. FXR coactivator (SRC-1) recruitment assay and transactivation assay on Hek293T cells of compounds 2 and 16

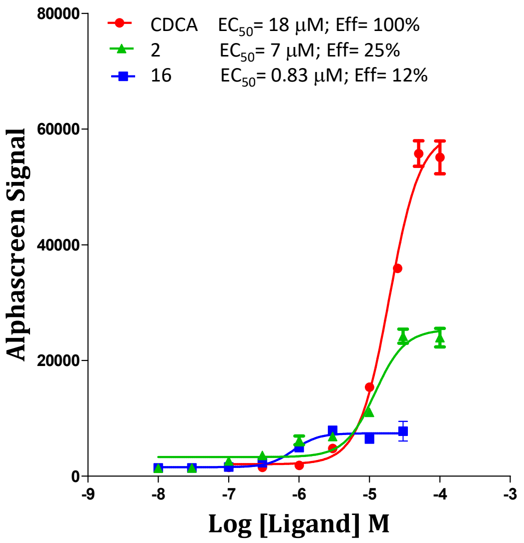

Figure S21. FXR coactivator (SRC-1) recruitment assay of compounds **2** and **16** with respect to CDCA as reference compound. The results show mean S.D. of triplicate samples from a representative experiment of two performed.

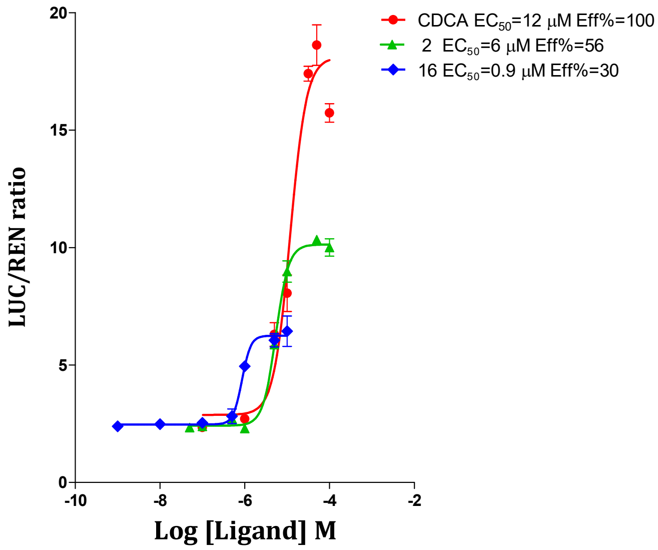

Figure S22. Transactivation assay on Hek293T cells performed by transfection of FXR–RXR CDS and the canonical FXRE(IR1) repeated three times upstream luciferase gene. The results show mean S.D. of triplicate samples from a representative experiment of two performed.

8. *In vitro* cytotoxicity and nuclear receptor selectivity of compounds 2 and 16

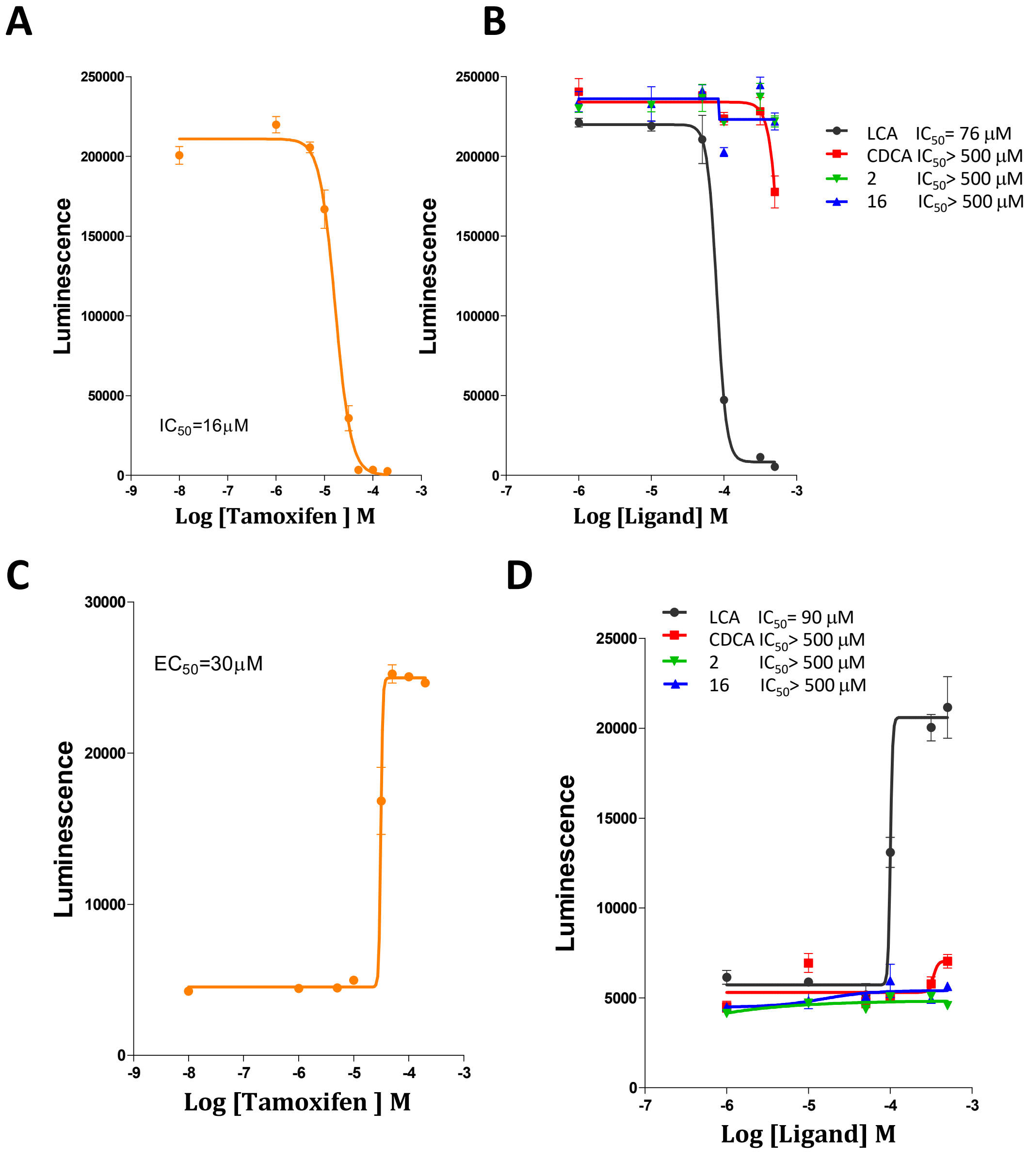

Figure S23. ATP measurement in HepG2 cells after treatment with tamoxifene (A), and LCA, CDCA, compound **2** and compound **16** (B). The results show mean S.D. of triplicate samples of two experiments performed. LDH release in HepG2 cells after treatment with tamoxifene (C), and LCA, CDCA, compound **2** and compound **16** (D). Tamoxifen and LCA were used as positive control. The results show mean S.D. of triplicate samples of two experiments performed.

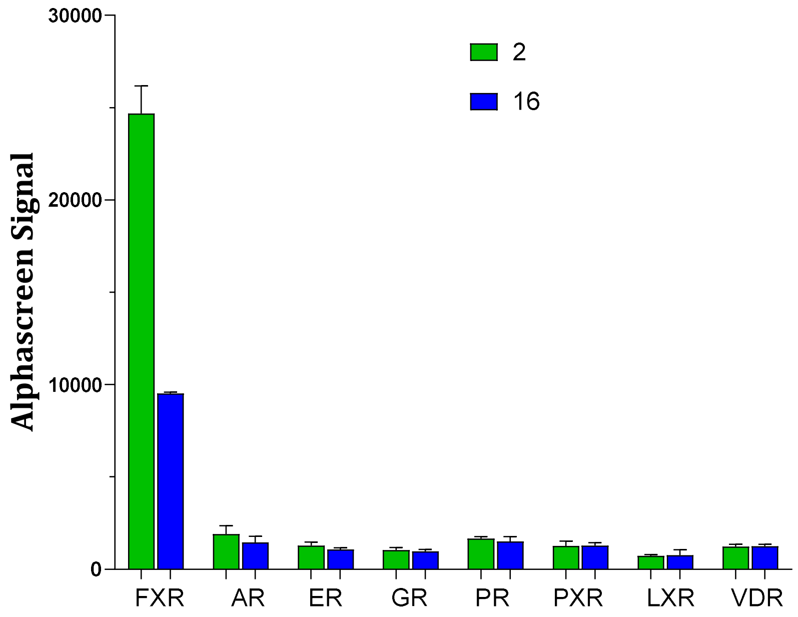

Figure S24. Nuclear receptor profiling (AlphaScreen) of compound **2** and **16** against a panel of nuclear receptors.

9. hX4 Molecular dynamics analyses

Table S2. Mean H-bond occupancy (%) calculated on the three replica MD simulations of hX4 in complex with agonist DCA-3P and compounds **2** and **16**.

Compound	hX4 activity	H-bond occupancy (%)							
		**Gln79**	**Arg82**	**Arg86**	**His92**	**Arg95**	**Lys96**	**Ser100**	**Val101**
**DCA-3P**	Agonist	7.3	99.5	97.7	1.9	14.4	65.5	0.0	0.0
**2**	Not active	0.0	10.2	3.7	0.4	2.3	1.6	2.3	14.5
**16**	Not Active	0.0	0.4	2.5	13.4	3.4	57.0	0.0	0.0

Compound	hX4 activity	H-bond occupancy (%)							
		Met102	Thr103	Ser154	Leu156	Glu157	Trp158	Arg159	Phe160
**DCA-3P**	Agonist	0.1	22.8	0.0	0.0	0.0	0.0	41.4	0.0
**2**	Not active	16.5	29.2	0.5	0.8	0.8	0.1	6.1	0.0
**16**	Not active	0.0	0.3	16.6	0.2	0.0	0.0	0.2	0.1

Compound	hX4 activity	H-bond occupancy (%)							
		Phe163	Ser166	Gly167	Ala168	Ser170	Ser171	Trp172	Cys173
**DCA-3P**	Agonist	0.0	0.0	0.0	0.0	0.0	0.0	0.0	0.0
**2**	Not active	0.0	0.0	0.0	0.0	0.0	0.0	0.0	0.0
**16**	Not active	0.4	0.1	0.2	0.2	17.3	0.1	0.1	12.9

Compound	hX4 activity	H-bond occupancy (%)							
		Glu174	Thr175	Ser176	Asp177	Val181	Gly236	Ile239	Tyr240
**DCA-3P**	Agonist	0.0	0.0	0.0	0.0	0.0	0.0	0.0	0.0
**2**	Not active	0.0	0.0	0.2	0.0	0.2	2.3	20.9	0.2
**16**	Not active	7.8	12.5	5.6	16.5	1.6	6.2	0.3	15.3

Compound	hX4 activity	H-bond occupancy (%)						
		Arg241	His243	Leu244	Asn245	Leu246	Glu247	Tyr254
**DCA-3P**	Agonist	0.0	0.0	0.0	14.9	0.3	0.0	62.3
**2**	Not active	33.3	23.2	22.4	36.1	15.9	2.6	23.6
**16**	Not active	76.8	1.4	0.1	15.0	0.0	0.0	0.8

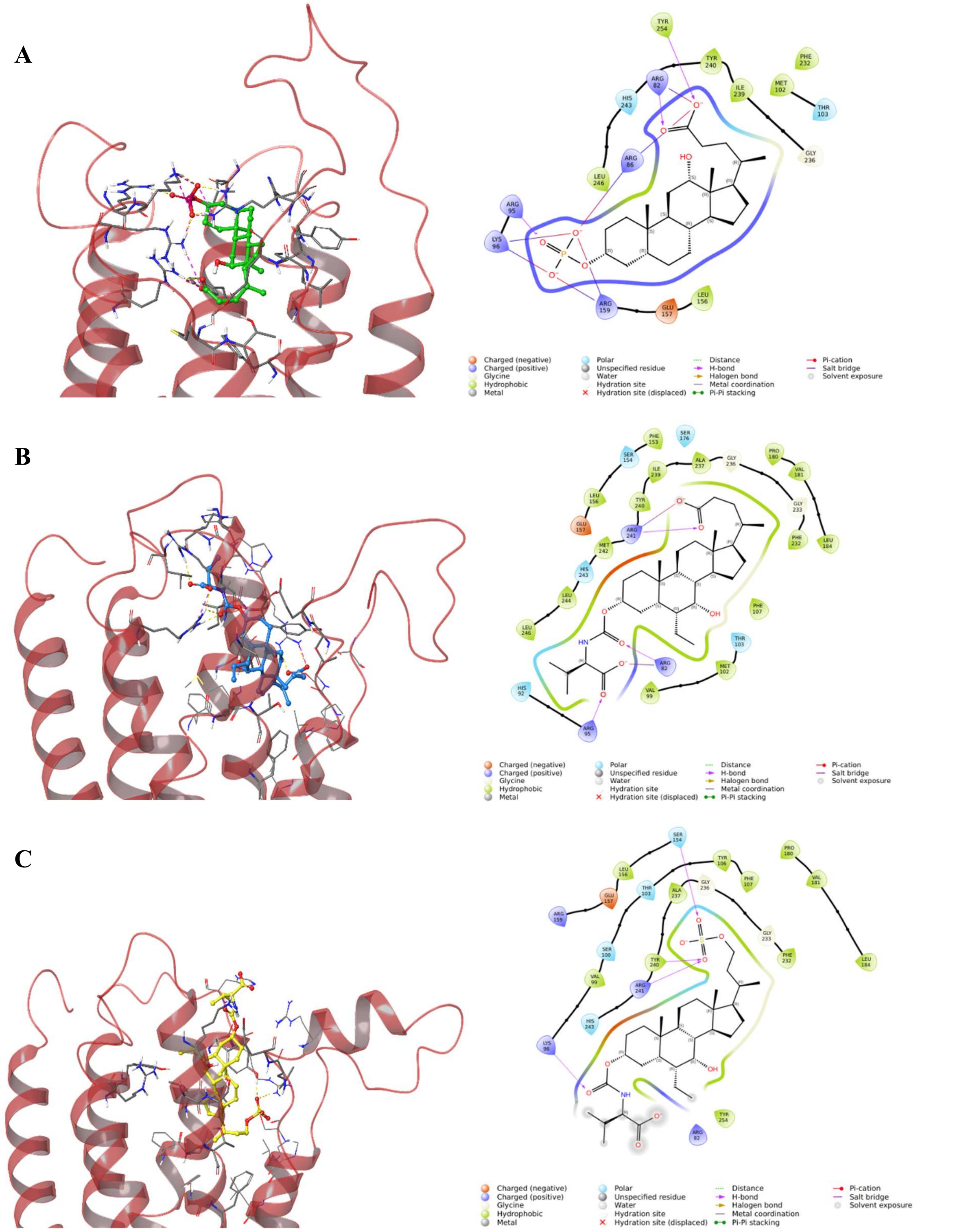

Figure S25. Images of the frames where the highest interaction energy between the ligand and the hX4 recorded among all the trajectories of the three replicas. The binding poses (left) adopted by DCA-3P, **2** and **16** (A, B and C, respectively) and the ligand interaction diagram (right) are reported.

10. Primers sequences for real-time PCR (Table S3)

Gene	Primer forward	Primer reverse
Bsep	GCAATGCCTCTCCTTGTTTGG	ACTTGTAACCATCTTCTGACATGC
CYP7α1	TTAGGAGAAGGCAAACGGGTG	AAATTGCAGAGCACAGCCCA
Ostβ	CTGTGGTGGTCATTATAAGCATGG	TCTGGTGGCTGCATCTTTTC
CD36	ATCACCTTCAACAACAACGACAC	CAGGGTCATGGGCTTATTCTCC
FASN	GCTGCCAGAGTCGGAGAAC	AGGGTCCATCGTGTGTGC
LDLR	GCCAAGTGGACTGCGACAAC	GACGAACTGCCGAGAGATGC
SREBP2	CCATTGACTCTGAGCCAGGAA	GTTAAAGGAGAGGCACAGGAA

11. References

[1] P. Franco, E. Porru, J. Fiori, A. Gioiello, B. Cerra, G. Roda, C. Caliceti, P. Simoni, A. Roda "Identification and quantification of oxo-bile acids in human faeces with liquid chromatography–mass spectrometry: a potent tool for human gut acidic sterolbiome studies", *J. Chromatogr. A* **2019**, *1585*, 70–81.

[2] E. Rosatelli, A. Carotti, B. Cerra, F. De Franco, D. Passeri, R. Pellicciari, A. Gioiello "Chemical exploration of TGR5 functional hot-spots: synthesis and structure-activity relationships of C7- and C23-Substituted cholic acid derivatives", *Eur. J. Med. Chem.* **2023**, *261*, 115851.

[3] B. Cerra, F. Venturoni, M. Souma, G. Ceccarelli, A. M. Lozza, D. Passeri, F. De Franco, I. R. Baxendale, R. Pellicciari, A. Macchiarulo, A. Gioiello "Development of 3α,7α-dihydroxy-6α-ethyl-24-nor-5β-cholan-23-sulfate sodium salt (INT-767): process optimization, synthesis and characterization of metabolites", *Eur. J. Med. Chem.* **2022**, *242*, 114652.

[4] R. Pellicciari, A. Gioiello, S. Macchiarulo, F. Perron-Sierra, S. Klaus (Intercept Pharmaceuticals, Les Laboratories Servier), "Tgr5 modulators and methods of use thereof", WO2016205475, **2016**.

[5] A. Kumari, L. Mittal, M. Srivastava, D. P. Pathak, S. Asthana "Deciphering the structural determinants critical in attaining the FXR partial agonism", *J. Phys. Chem. B* **2023**, *127*, 465−485.
